# Combined Non-Invasive Prediction and New Biomarkers of Oral and Fecal Microbiota in Patients With Gastric and Colorectal Cancer

**DOI:** 10.3389/fcimb.2022.830684

**Published:** 2022-05-19

**Authors:** Chaoyang Zhang, Asheng Hu, Jingxing Li, Fangfang Zhang, Pei Zhong, Yaxian Li, Yongxiang Li

**Affiliations:** ^1^ Department of General Surgery, The First Affiliated Hospital of Anhui Medical University, Hefei, China; ^2^ Department of Computer Science, Faculty of Science, University of Western Ontario, London, ON, Canada; ^3^ Department of Anesthesiology, Hefei BOE Hospital, Hefei, China; ^4^ Department of Clinical Laboratory, The First Affiliated Hospital of Anhui Medical University, Hefei, China

**Keywords:** gastric cancer, colorectal cancer, microbiota, oral cavity, stool, 16S rRNA

## Abstract

**Background:**

There is no information on the commonality and specificity of oral and fecal microbiota in patients with gastric cancer (GC) and colorectal cancer (CRC).

**Methods:**

The high-throughput 16S rRNA gene V4 region sequencing was used to perform bioinformatics analysis of oral, fecal, and tissue microbiota in GC (76 subjects), CRC (53), and healthy controls (HC, 70). Furthermore, we determined the microbial characteristics of each part, constructed and verified three classifiers for GC and CRC, and evaluated curves of receiver operating characteristic and precision–recall with probability of disease.

**Results:**

Compared to HC, the microbial richness and diversity of GC and CRC decreased in oral cavity and increased in stool; additionally, these indexes in GC tissue were higher than those in CRC tissue. In GC and CRC patients, *Haemophilus*, *Neisseria*, *Faecalibacterium*, and *Romboutsia* were significantly reduced compared to the relative abundance value of oral or fecal bacterial genera in the HC group, while the *Streptococcus*, *Gemella*, *Escherichia-Shigella*, and *Fusobacterium* were significantly increased. The oral and tissue microbiota have similar and abundant shared bacterial networks. The single and combined microbial detection have good AUC values based on POD indices for predicting GC, CRC, and gastrointestinal (GI) cancers (GC and CRC).

**Conclusion:**

This study is the first to examine the characteristics of oral, fecal, and tumor microbiota in GC and CRC patients, and the similarities and differences in their microbial changes are reported. These oral or fecal bacteria (*Haemophilus*, *Neisseria*, *Faecalibacterium*, *Romboutsia*, *Streptococcus*, *Gemella*, *Escherichia-Shigella*, and *Fusobacterium*) may be involved in tumor evolution as potentially characteristic genera. In addition, both oral and fecal microbial detection may provide a solid theoretical foundation for the non-invasive prediction of these cancers.

## Introduction

Gastric cancer (GC) is the fifth most common cancer and the fourth leading cause of cancer death worldwide, with half of all cases occurring in East Asia (primarily in China) (Smyth et al., 2020; Sung et al., 2021). *Helicobacter pylori* infection contributes significantly to non-cardiac GC development (Sugano et al., 2015; Du et al., 2020), and its eradication reduces the risk of GC (Graham, 2015; Engstrand and Graham, 2020). Only 1%–3% of people infected with *H. pylori* develop GC (Peek and Crabtree, 2006; LaCourse et al., 2021). Many studies have reported that other microbiota, such as *Peptostreptococcus*, *Desulfovibrio*, and *Fusobacterium*, play a role in GC occurrence and development (Coker et al., 2018; Liu et al., 2021). The endoscopy and biopsy techniques are effective in detecting patients with early-stage gastric cancer (EGC), and their 5-year survival rate is estimated up to 80% (Eusebi et al., 2020). Although new endoscopic techniques such as narrowband imaging (NBI) (Pimentel-Nunes et al., 2012), magnetic controlled capsule gastroscopy (MCCG) (Zhao et al., 2018), and serum markers such as pepsinogen, gastrin, and tumor markers can detect GC (Huang et al., 2015; Zagari et al., 2017; Grady et al., 2021), there is still a lack of effective, convenient, low-cost, and non-invasive tests.

The diversity and richness of microbiota change significantly during the evolution of superficial gastritis (SG), atrophic gastritis (AG), intestinal metaplasia (IM), and GC (Coker et al., 2018). With the increasing research on the relationship between microbiota and systemic tumors (especially digestive tract tumors), many studies have reported that oral microbiota is related to colorectal cancer (CRC) (Flemer et al., 2018; Zhang et al., 2020), esophageal cancer (Chen et al., 2015), pancreatic cancer (Farrell et al., 2012; Torres et al., 2015), and oral cancer (Pushalkar et al., 2012; Schmidt et al., 2014), while fecal microbiota is related to CRC (Flemer et al., 2017), liver cancer (Ren et al., 2019), and breast cancer (Terrisse et al., 2021). Due to microbial differences between tumor patients and healthy controls (HC), microbial detection has the potential to be a new non-invasive screen test (Flemer et al., 2018; Ren et al., 2019; Zhang et al., 2020; Yonekura et al., 2021). In the meantime, the role of gastrointestinal (GI) microbiota in the oral cavity and stool in GC remains unknown.

CRC is now the third most common cancer and the second leading cause of cancer death worldwide with an increase in morbidity and mortality rates in China over the last decade (Sung et al., 2021). Colonoscopy remains the gold standard for CRC detection, while imaging (CT: computed tomography), stool (FOBT: fecal occult blood test; FIT: fecal immune test), serum, and genetic material screening all have varying detection rates and limitations (Issa and Noureddine, 2017). It has been reported that oral and fecal microbiota play a role in developing polyps, adenomas, and CRC; however, the relationship between microbiota and CRC remains unknown (Flemer et al., 2017; Flemer et al., 2018; Zhang et al., 2020). In addition, the microbial relationship between GC and CRC requires further investigation.

The body produces approximately 1,000 ml of saliva (containing 10^11^ types of bacteria) each day, almost all of which enters the GI tract, forming a loop between the subject’s oral, fecal, and GI microbiota (Andersson et al., 2008; Pfaffe et al., 2011; Segata et al., 2012). This study investigated the microbial relationship among oral cavity, stool, tumor, and paracancerous tissue in GC and CRC patients. Some novel oral and fecal potential microbial markers (genera) (*Haemophilus*, *Neisseria*, *Faecalibacterium*, *Romboutsia*, *Streptococcus*, *Gemella*, *Escherichia-Shigella*, *Fusobacterium*, etc.) were reported in this study. Furthermore, we evaluated the potential of oral and fecal microbiota as non-invasive biomarkers for GC and CRC through the validation cohort and diagnostic cohort.

## Materials and Methods

### Participant Information

The study protocol was approved by the Committee on Medical Ethics of the First Affiliated Hospital of Anhui Medical University (Quick-PJ 2021-13-23) adopting prospective specimen collection and retrospective blind evaluation (PRoBE) methods (Pepe et al., 2008). Before sampling, all participants were notified, and their written consent was obtained before any procedure. A total of 353 oral, stool, tumor, and paracancerous tissue samples from GC (88) and CRC (61) inpatients were prospectively collected, eventually including 311 samples for the study. The collected samples consisted of samples from GC (76) [oral (70), stool (49), tumor (33), and paracancerous tissue (36)] and CRC (53) [oral (42), stool (33), tumor (24), and paracancerous tissue (24)], followed by 16S rRNA Miseq sequencing. The demographic information, relevant clinical data, pathological diagnosis, and tumor staging for inpatients were obtained from hospital electronic medical records and questionnaires (**Table 1**) (Yaghoobi et al., 2017; Ren et al., 2019; Shao et al., 2019; Zhang et al., 2020). Furthermore, paired oral and fecal samples from 70 healthy people (Qin et al., 2014) were collected with a detailed description of the participant’s information shown in **Table 1**. Meanwhile, dietary habits were collected and screened by referring to the food frequency questionnaire (FFQ) (Claesson et al., 2012) and relevant articles (**Table 1** and **Table S1**) (Tsugane and Sasazuki, 2007; Shao et al., 2019). The exclusion criteria for the GC and CRC cohorts were as follows: (1) taking antibiotics or probiotics within 1 month of inclusion; (2) cancer treatment within 3 months of inclusion; (3) presence of other diseases, such as irritable bowel syndrome (IBS), inflammatory bowel disease (IBD), and metabolic diseases; and (4) participants who lacked the clinical information (**Table S1**).

**Table 1:** Clinical characteristics and diet information of the enrolled participants.

Characteristics	HC (*n* = 70)	GC (*n* = 76)	CRC (*n* = 53)	*p*-value
**Age (years)**	60.99 ± 9.06	63.63 ± 9.56	59.21 ± 14.40	0.091 ^a^
**BMI (kg/m^2^)**	23.61 ± 3.04	22.54 ± 3.34	23.64 ± 3.02	0.064*
**Sex**				0.063^#^
Male	36 (51.4%)	51 (67.1%)	37 (69.8%)	
Female	34 (48.6%)	25 (32.9%)	16 (30.2%)	
**Education, *n* (%)**				0.003^#^
No education	7 (10.0%)	21 (27.6%)	14 (26.4%)	
Primary education	10 (14.3%)	21 (27.6%)	9 (17.0%)	
Secondary education or more	53 (75.7%)	34 (44.7%)	30 (56.6%)	
**Smoking status**				<0.001^#^
Never smoker	50 (71.4%)	39 (51.3%)	28 (52.8%)	
Former smoker	7 (10.0%)	30 (39.5%)	21 (39.6%)	
Current smoker	13 (18.6%)	7 (9.2%)	4 (7.5%)	
**Alcohol consumption**				0.138^#^
Never drink	38 (54.3%)	44 (57.9%)	34 (64.2%)	
<1 standard drink per day	28 (40.0%)	20 (26.3%)	13 (24.5%)	
≥1standard drink per day	4 (5.7%)	12 (15.8%)	6 (11.3%)	
**Household income, RMB**				0.002^#^
≤5,000	44 (62.9%)	68 (89.5%)	45 (84.9%)	
5,000–10,000	22 (31.4%)	7 (9.2%)	7 (13.2%)	
≥10,000	4 (5.7%)	1 (1.3%)	1 (1.9%)	
**Family history of cancer, *n* (%)**	15 (21.4%)	18 (23.7%)	16 (30.2%)	0.521^#^
**Diabetes**				
Yes	0 (0%)	8 (10.5%)	5 (9.4%)	
No	70 (100%)	68 (89.5%)	48 (90.6%)	
**Tumor location**				
Upper third of stomach	–	38 (50%)	–	
Middle third of stomach	–	18 (24%)	–	
Lower third of stomach	–	20 (26%)	–	
Proximal colon	–	–	7 (13%)	
Distal colon	–	–	12 (23%)	
Rectum	–	–	34 (64%)	
**Tumor size (cm)**	–	3.89 ± 1.88	4.14 ± 1.92	0.596 ^b^
**TNM stage**				0.460^#^
I–II	–	38 (50%)	23 (43.4%)	
III–IV	–	38 (50%)	30 (56.6%)	
**Fresh vegetables**				0.921^#^
≥5 days/per week	64 (91.4%)	71 (93.4%)	47 (88.7%)	
2–4 days/per week	5 (7.1%)	4 (5.3%)	5 (9.4%)	
≤1 day/per week	1 (1.4%)	1 (1.3%)	1 (1.9%)	
**Fresh fruits**				<0.001^#^
≥5 days/per week	35 (50.0%)	9 (11.8%)	7 (13.2%)	
2–4 days/per week	22 (31.4%)	25 (32.9%)	16 (30.2%)	
≤1 day/per week	13 (18.6%)	42 (55.3%)	30 (56.6%)	
**High-fat food**				0.016^#^
≥5 days/per week	3 (4.3%)	10 (13.2%)	6 (11.3%)	
2–4 days/per week	19 (27.1%)	25 (32.9%)	26 (49.1%)	
≤1 day/per week	48 (68.6%)	41 (53.9%)	21 (39.6%)	
**High-sugar food**				0.195^#^
≥5 days/per week	6 (8.6%)	3 (3.9%)	1 (1.9%)	
2–4 days/per week	20 (28.6%)	15 (19.7%)	10 (18.9%)	
≤1 day/per week	44 (62.9%)	58 (76.3%)	42 (79.2%)	
**Pickled food**				0.319^#^
≥5 days/per week	20 (28.6%)	18 (23.7%)	12 (22.6%)	
2–4 days/per week	23 (32.9%)	25 (32.9%)	11 (20.8%)	
≤1 day/per week	27 (38.6%)	33 (43.4%)	30 (56.6%)	
**Moldy food**				0.166^#^
≥5 days/per week	0 (0%)	0 (0%)	0 (0%)	
2–4 days/per week	2 (2.9%)	7 (9.2%)	6 (11.3%)	
≤1 day/per week	68 (97.1%)	69 (90.8%)	47 (88.7%)	
**FOBT**				
Yes	0/70 (0%)	12/58 (20.7%)	34/51 (66.7%)	
No	70/70 (100%)	46/58 (79.3%)	17/51 (33.3%)	

^*^One-way analysis of variance (ANOVA); ^#^Pearson chi-square test; ^a^Kruskal–Wallis H test; ^b^Wilcoxon. BM, body mass index; RMB, renminbi; FOBT, fecal occult blood test; 1 standard drink = 10 g alcohol.

### Tissue Sample Collection for GC and CRC Patients

Following isolation of lesions surgically, the samples comprising cancer lesions and paracancerous tissues (with no abnormality on the mucosal surface, 5–10 cm from the tumor boundary) were transferred into a 3-ml RNAlater sterile cryotubes (Qiagen, Hilden, Germany) and transported to the laboratory in an ice pack and stored it at −80°C until further use.

### Oral Sample Collection of Subjects

The oral samples were collected by swabbing the medial sides of both cheeks of the subjects using a cotton swab (Flemer et al., 2018), followed by transferring the swabs into a sterile test tube and stored them at −80°C until further use. It was ensured that none of the subjects had any oral disease and received any drug treatment before sampling as per the NIH Human Microbiome Project-Core microbiome sampling protocol (Gevers et al., 2012).

### Stool Sample Collection of Subjects

The subject’s fresh stool samples were collected in a special stool tube sterilized internally and were packed using ice packs and delivered to the laboratory for further processing. The samples were divided into 200-mg portions and kept at −80°C until further use. The oral and fecal samples were collected prior to surgery in GC and CRC inpatients.

### Extraction of Genome DNA, Library Preparation, and High-Throughput 16S Ribosomal RNA Gene Sequencing

Total genome DNA from samples was extracted using the CTAB method. DNA concentration and purity was monitored on 1% agarose gels. According to the concentration, DNA was diluted to 1 ng/µl using sterile water. The high-throughput 16S ribosomal RNA gene sequencing was carried out using specific primers of 16S rRNA gene V4 region 515F: -GTGCCAGCMGCCGCGGTAA- and 806R: -GGACTACHVGGGTWTCTAAT-. The sequencing libraries were created using a TruSeq^®^ DNA PCR-Free Sample Preparation Kit (Illumina, USA) according to manufacturer’s instructions and index codes were added. The Qubit@ 2.0 Fluorometer (Thermo Scientific) and Bioanalyzer 2100 system (Agilent) were used to evaluate the library’s quality. The library was sequenced on an Illumina NovaSeq platform (Novogene, Beijing, China), resulting in 250-bp paired-end reads.

### Data Processing

Paired terminal readings were assigned to each sample based on its unique barcode and truncated by excising the barcode and primer sequences. FLASH was used to perform sequence assembly (Magoc and Salzberg, 2011). The data filtering was done following QIIME quality control process (Caporaso et al., 2010). The database was consulted, using UCHIME algorithm for chimeric removal, and eventually obtained an effective label (Edgar et al., 2011).

### Operation Classification Unit Clustering and Classification Annotation

Sequence analysis was performed using Uparse software. Sequences with ≥97% similarity were assigned to the same OTU. The Silva database was used to annotate classification data using Mothur algorithm (Quast et al., 2013). Multiple sequence alignments were performed using MUSCLE software to study the phylogenetic relationship of different OTUs and the differences in the dominant species (Edgar, 2004). The abundance of OTUs was then normalized using the samples with the least sequence as the standard.

### Bacterial Diversity and Taxonomic Analysis

The raw data were normalized by the QIMME1/normalize module [cumulative-sum scaling (CSS)]. The α-diversity of each sample was evaluated by richness (Chao 1) and biodiversity (Shannon) (Zhang et al., 2021). These indicators were calculated using QIIME (version 1.9.1) and displayed using R package ggplot2 (version 2.15.3). To evaluate the microbial diversity among samples, the β-diversity among samples was evaluated using principal coordinate analysis (PCoA) and cluster analysis. The PCoA of this study was built on a distance (dissimilarity) matrix of Bray–Curtis indexes.

### Identification of Microbial OTU Markers

The optimal OTU marker described above was determined using fivefold cross-validation. The POD index was defined as the ratio between the number of randomly generated decision trees with predicted samples such as GC, CRC, or GI to the number of healthy controls. The optimal OTU set was used to calculate POD indices for the training and testing cohorts. The receiver operating characteristic (ROC) (R version 3.6.0, pROC package version 1.18.0) area under the curve (AUC) was used to represent the ROC effect. Characteristic biomarker genera were found by Random Forest (Version 3.6.0, randomForest package version 4.6-14) (Ren et al., 2019).

### Statistical Analysis

The clinical data were analyzed using one-way ANOVA, the Kruskal–Wallis *H* test, chi-square test, or Fisher’s exact test (**Table 1**). The difference in microbial diversity between two groups was estimated by using the Mann–Whitney *U* test, and two-stage FDR corrections were applied to adjust *p*-values. SPSS V.23.0 for Windows (SPSS, USA), GraphPad Prism V.9.0 (Graph Pad Software, USA), and Microsoft Excel (Microsoft, USA) were used for statistical analysis. The statistical significance was set at *p* < 0.05.

## Results

**Figure 1:**
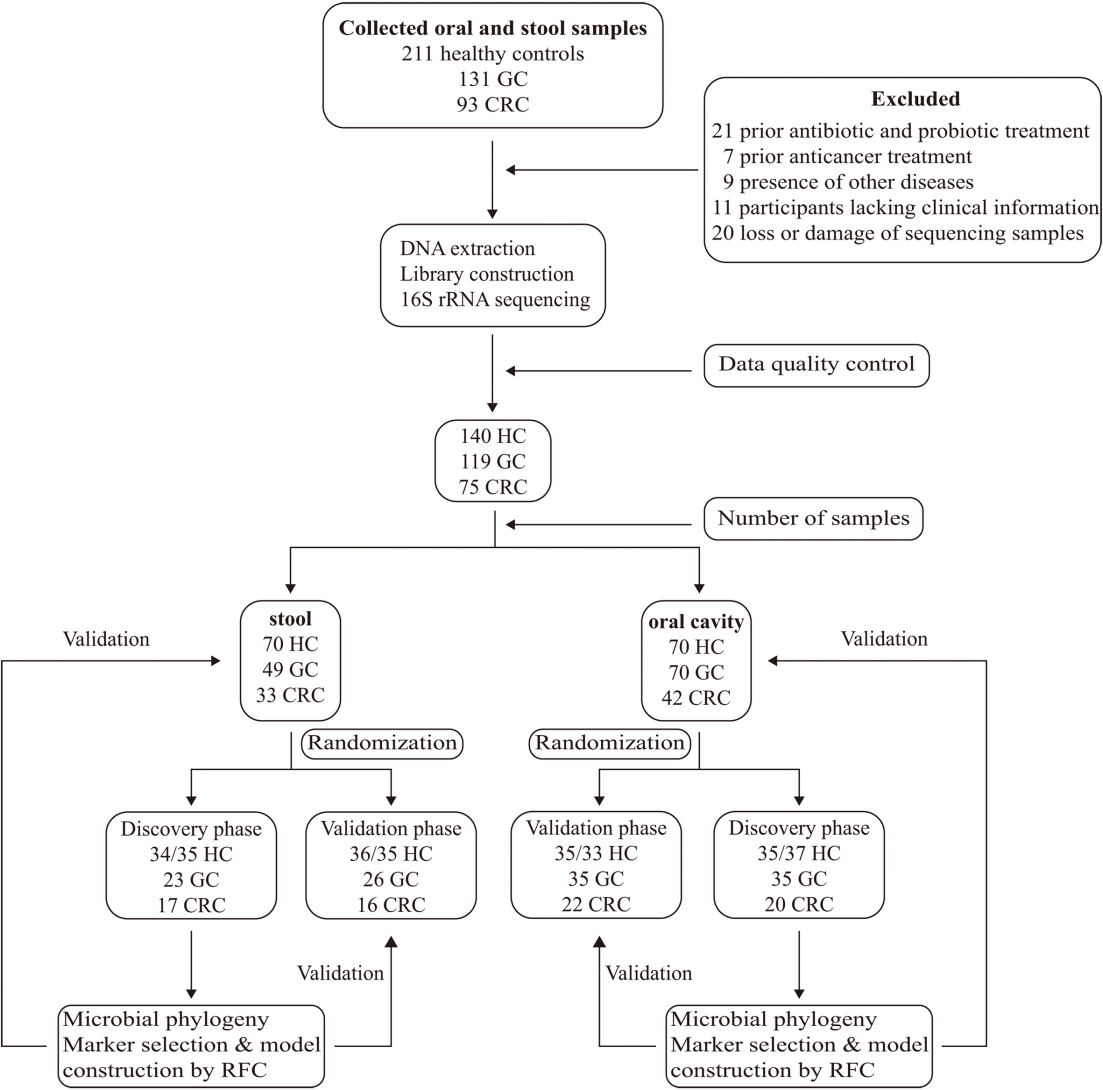
Study design and flow diagram. A total of 435 oral and fecal samples from the same area were prospectively collected and 334 samples (140 HC, 119 GC, and 75 CRC) were included after systematic elimination and pathological diagnosis. The above samples came from 199 qualified participants (including 70 healthy controls, 76 GC patients, and 53 CRC patients) and were randomly divided into a discovery phase and a verification phase. In the discovery phase, we characterized microbiota of 72 HC (37/35 oral, 35/37 stool), 60 GC (35 oral, 25 stool), and 38 CRC (21 oral, 17 stool), and identified microbial markers. The GC and CRC classifiers were constructed through the random forest model among GC, CRC cohort, and HC cohort. In the validation phase, we used 68 HC (33/35 oral, 35/33 stool), 59 GC (35 oral, 24 stool), and 37 CRC (21 oral, 16 stool) to validate diagnosis efficacy of the GC and CRC classifiers. Furthermore, all oral samples (70 HC, 70 GC, and 42 CRC) and fecal samples (70 HC, 49 GC, and 33 CRC) were used as another independent diagnostic stage to verify the potential of the GC and CRC classifiers. HC, healthy controls; GC, gastric cancer; CRC, colorectal cancer.

A total of 435 oral and fecal samples from the same area were prospectively collected. Of these, 334 samples (140 HC, 119 GC, and 75 CRC) were included after systematic elimination and pathological diagnosis. Meanwhile, 117 tumors and paracancerous tissues were collected from some participants (69 GC, 48 CRC). The above samples were acquired from 199 qualified participants (including 70 HC, 76 GC, and 53 CRC) and were randomly divided into a discovery phase and a verification phase. In the discovery phase, we characterized microbiota of 72/71 HC (37/35 oral, 36/35 stool), 68 GC (35 oral, 23 stool), and 37 CRC (20 oral, 17 stool), and identified microbial markers. The GC and CRC classifiers were constructed by using the random forest model between the GC/CRC cohort and the HC cohort. In the validation phase, we used 68/69 HC (33/35 oral, 34/35 stool), 61 GC (35 oral, 26 stool), and 38 CRC (22 oral, 16 stool) to validate diagnosis efficacy of the GC and CRC classifiers. Furthermore, all oral samples (70 HC, 70 GC, and 42 CRC) and fecal samples (70 HC, 49 GC, and 33 CRC) were used as another independent diagnostic stage to verify the potential of the GC and CRC classifiers (**Figure 1**).

### Clinical and Dietary Characteristics of Participants

The analysis of clinical data revealed that, compared to GC and CRC, the number of people in HC who met these criteria (secondary education or higher, never smoked, household income >5,000 RMB) increased significantly. In dietary surveys, healthy people had a higher frequency of intake of fresh fruits and a lower frequency of intake of high-fat foods than GC and CRC groups (**Table 1**). The detailed clinical and dietary information of each participant is presented in **Table S1**.

### Microbial Diversity and Phylogenetic Profiles of GC and CRC in Oral Cavity, Stool, and Tissue

**Figure 2:**
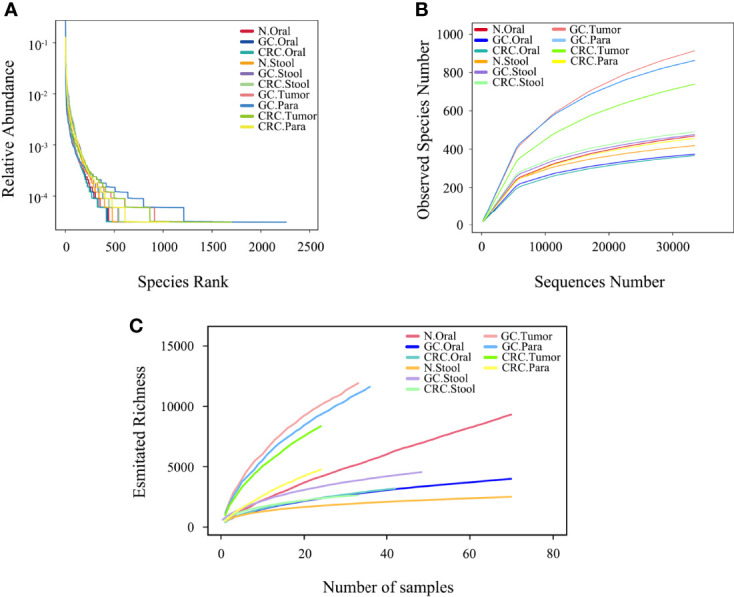
Microbial diversity in each group. **(A)** The evenness of the ten microbiota groups was assessed by graded abundance curve. **(B)** The accumulation curve between the sequencing depth of ten samples and the number of observed species. **(C)** The accumulation curve between ten samples and estimated richness.

The rank abundance curve showed that, compared to oral and fecal samples, the microbiota in GC and CRC tissue had greater richness and evenness (**Figure 2A**). The number of observed species in each group approached saturation as the sequencing depth increased (**Figure 2B**); as the number of samples increased, the richness of each group tended to stabilize (**Figure 2C**). The microbial richness and species number in GC and CRC oral samples decreased compared to HC but increased in fecal samples, while the index in the tumor was higher than that in the paracancerous tissue.

**Figure 3:**
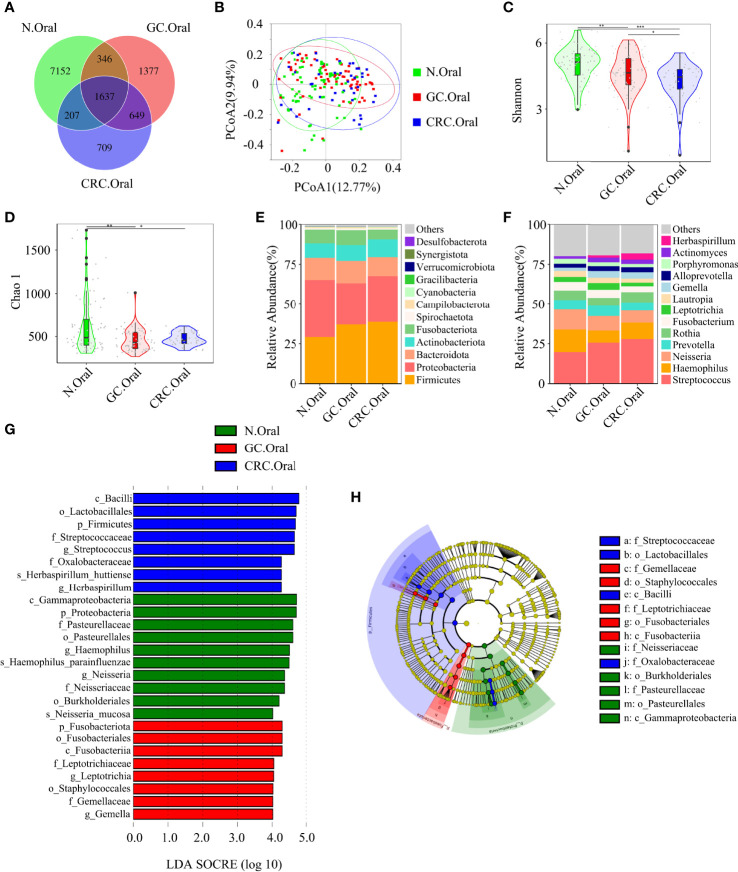
Compared with the HC (*n* = 70), the oral microbial diversity of patients with GC (*n* = 70) and CRC (*n* = 42) was reduced. **(A)** The Venn diagram displayed the overlaps among groups, showing a total of 12,077 OTUs. **(B)** PCoA calculated β-diversity on a distance (dissimilarity) matrix of Bray–Curtis indexes. The Shannon index **(C)** and Chao1 index **(D)** were used to evaluate the oral microbial diversity of patients with HC, GC, and CRC. Composition of oral microbiota at the phylum level **(E)** and genus level **(F)** among the three groups. The linear discriminant analysis (LDA) effect size (LEfSe) method was used to analyze the specific characterization of the oral microbiota of patients with HC, GC, and CRC. **(G, H)** The LEfSe method identified the most divergent taxa in GC and CRC and scored the two groups of oral samples by LDA. Only the taxa that reach the effective threshold of LDA >4 were displayed. The brightness of each point was proportional to the size of its effect. *p < 0.05, **p < 0.01, ***p < 0.001; OTUs, operational taxonomy units; PCoA, principal coordinate analysis.

**Figure 4:**
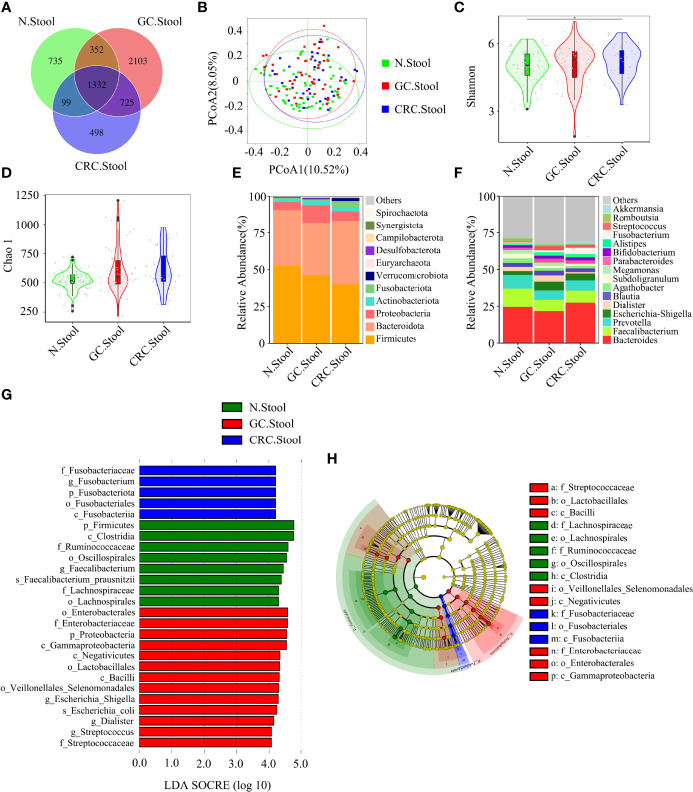
There were differences in fecal microbial diversity among HC (*n* = 70), GC (*n* = 49), and CRC (*n* = 33). **(A)** The Venn diagram displayed the overlaps among groups, showing a total of 5844 OTUs. **(B)** PCoA calculated β-diversity on a distance (dissimilarity) matrix of Bray–Curtis indexes. The Shannon index **(C)** and Chao1 index **(D)** were used to evaluate the fecal microbial diversity of patients with HC, GC, and CRC. Composition of fecal microbiota at the phylum level **(E)** and genus level **(F)** among the three groups. LDA and LEfSe methods were used to analyze the specific characterization of the fecal microbiota of patients with HC, GC, and CRC. **(G, H)** The LEfSe method identified the most divergent taxa in GC and CRC and scored the two groups of fecal samples by LDA. Only the taxa that reach the effective threshold of LDA >4 were displayed. The brightness of each point was proportional to the size of its effect. *p < 0.05.

**Figure 5:**
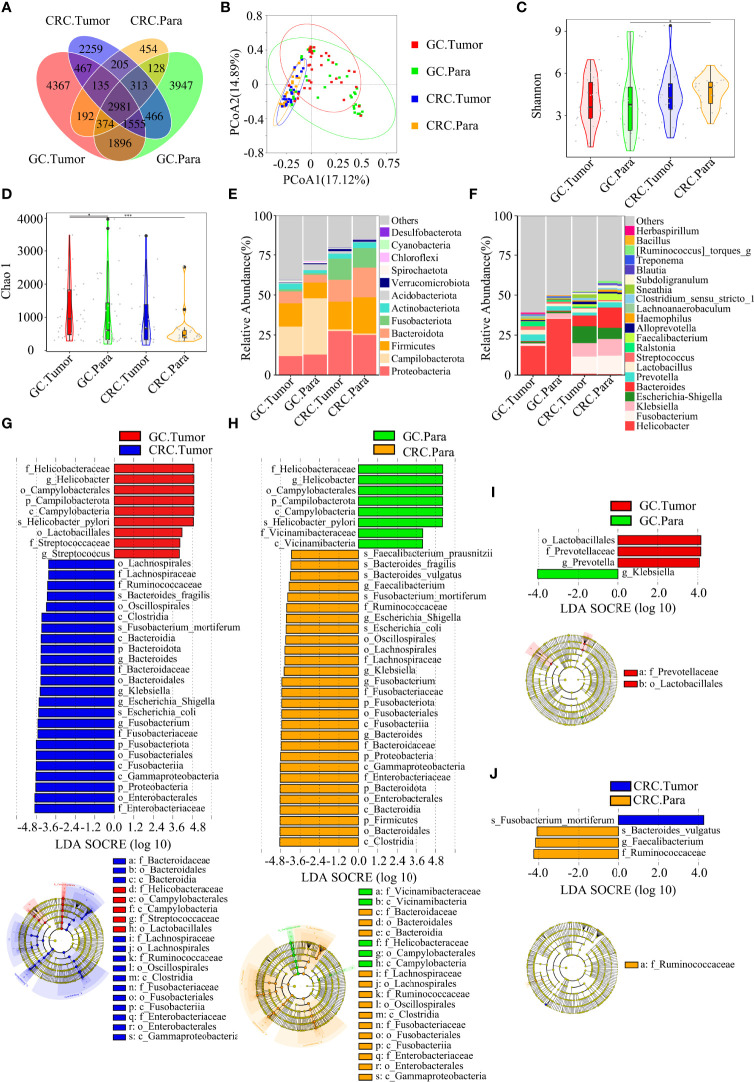
There were many similarities and differences in the microbial diversity of tissue between GC and CRC. GC.Tumor (*n* = 33), GC.Para (*n* = 36), CRC.Tumor (*n* = 24), and CRC.Para (*n* = 24). **(A)** The Venn diagram displayed the overlaps among groups, showing a total of 19,739 OTUs. **(B)** PCoA calculated β-diversity on a distance (dissimilarity) matrix of Bray–Curtis indexes. The Shannon index **(C)** and Chao1 index **(D)** were used to evaluate the microbial diversity of tissues in patients with GC and CRC. Composition of tissue microbiota at the phylum level **(E)** and genus level **(F)** among the four groups. LDA and LEfSe methods were used to analyze the specific characterization of the tissue microbiota of patients with GC and CRC. **(G)** The LEfSe method identified the most divergent taxa in GC and CRC and scored both groups of tumor samples by LDA. **(H)** The LEfSe method identified the most divergent taxa in GC and CRC, and scored both groups of paracancerous samples by LDA. **(I)** The LEfSe method identified the most divergent taxa in tumor and paracancerous tissues of GC and scored both groups of samples by LDA. **(J)** The LEfSe method identified the most divergent taxa in tumor and paracancerous tissues of CRC and scored both groups of samples by LDA. Only the taxa that reach the effective threshold of LDA >4 were displayed. The brightness of each point was proportional to the size of its effect. Para, paracancerous tissues. *p < 0.05, ***p < 0.001.

The Venn diagram showed that, compared to HC, the oral OTUs in GC and CRC were significantly reduced, while the fecal OTUs were increased. Interestingly, the oral, fecal, and tissue OTUs in GC were significantly higher than those in CRC (**Figures 3A**, **4A**, **5A**). PCoA based on Bray–Curtis distance and ANOSIM confirmed differences in the oral and fecal microbial structure of participants in the three groups, as well as in the tissues of GC/CRC patients (**Figures 3B**, **4B**, **5B**, and **Figures S1A–C**). We calculated two α-diversity indices to assess bacterial α-diversity across all groups (**Table S2**). The oral microbial diversity (Shannon index, Chao 1) of the GC and CRC groups was significantly lower than that of the HC group. On Shannon index, CRC was lower than GC (**Figures 3C, D**). Compared to HC, Shannon index of fecal microbiota in the CRC group was significantly higher (**Figures 4C, D**). There were differences in Shannon indexes between GC and CRC paracancerous tissue and in Chao 1 between tumor and paracancerous tissue of GC (**Figures 5C, D**).

Firmicutes, Proteobacteria, and Bacteroidete*s* were the three dominant phyla in oral samples, accounting for more than 75% of each group (**Figure 3E**). Compared to HC, the proportion of Firmicutes of GC and CRC increased, and the proportion of Proteobacteria decreased. The proportion of Fusobacteriota of HC and GC was higher than that of CRC (**Figure 3E**). GC and CRC had a lower proportion of *Haemophilus* and *Neisseria* at the genus level than HC, but a higher proportion of *Streptococcus* and *Herbaspirillum* (**Figure 3F**). The pairwise comparison of linear discriminant analysis (LDA) values (LDA > 4) of oral groups also showed the previously mentioned changes in phylum and genus level (**Figures S2A–C**). The three dominant phyla of fecal samples were consistent with the oral cavity and accounted for more than 85% of each group’s sequence (**Figure 4E**). At the phylum level, compared to HC and CRC, the proportion of Proteobacteria of GC increased; compared to HC and GC, the proportion of Fusobacteriota of CRC increased (**Figure 4E**). At the genus level, the proportion of *Escherichia-Shigella* in GC and CRC increased compared to HC; the proportion of *Fusobacterium* in CRC increased compared to HC and GC, and the proportion of *Streptococcus* in GC was higher than that of HC (**Figure 4F**). These changes were also observed in a pairwise comparison of LDA values (LDA > 4) of fecal groups (**Figures S3A–C**). We also observed that *Campilobacterota* and *Helicobacter* increased significantly in GC tissues, in accordance with the previous studies. Surprisingly, the abundance of *Helicobacter* was higher in paracancerous tissue of GC than in tumor tissue (**Figures 5E, F**). There was little difference in the proportion of phylum and genus level between tumor and paracancerous tissue in GC and CRC patients. Nevertheless, there were significant differences between GC and CRC groups. Compared to GC, the proportion of Proteobacteria, Fusobacteriota, and Bacteroidota in CRC bacteria phylum was found to increase, while the proportion of *Fusobacterium*, *Klebsiella*, *Escherichia-Shigella*, and *Bacteroides* in the genus increased (**Figures 5G–J**).

LEfSe analysis was applied to identify the taxa most associated with each of the three groups of participants. The rich genera of GC oral samples were *Leptotrichia* and *Gemella*, the rich genera of CRC were *Streptococcus* and *Herbaspirillum*, and the rich genera of HC were *Haemophilus* and *Neisseria* (**Figures 3G, H**). *Escherichia-Shigella*, *Dialister*, and *Streptococcus* were enriched in GC stool samples; *Fusobacterium* was enriched in CRC; and *Faecalibacterium* was enriched in HC (**Figures 4G, H**). The characteristic bacterial genera of oral samples of Lefse, RF, and PLSDA-VIP were compared, and the results showed that *Gemella* of GC, *Streptococcus* of CRC, and *Neisseria* of HC were all suggested as important genera in the three algorithms. In the comparison of characteristic bacterial genera of the three groups of fecal samples, *Lactobacillus* of GC, *Peptostreptococcus* of CRC, and *Faecalibacterium* of HC showed differences in the two algorithms (**Figures S2D–G**, **S3D–G**). Concurrently, pairwise MetaStat analysis comparisons were performed on all samples at the phylum and genus levels (**Figures S4A–F**, **S5A–F**, **S6A–H** and **Tables S3–5**).

**Figure 6:**
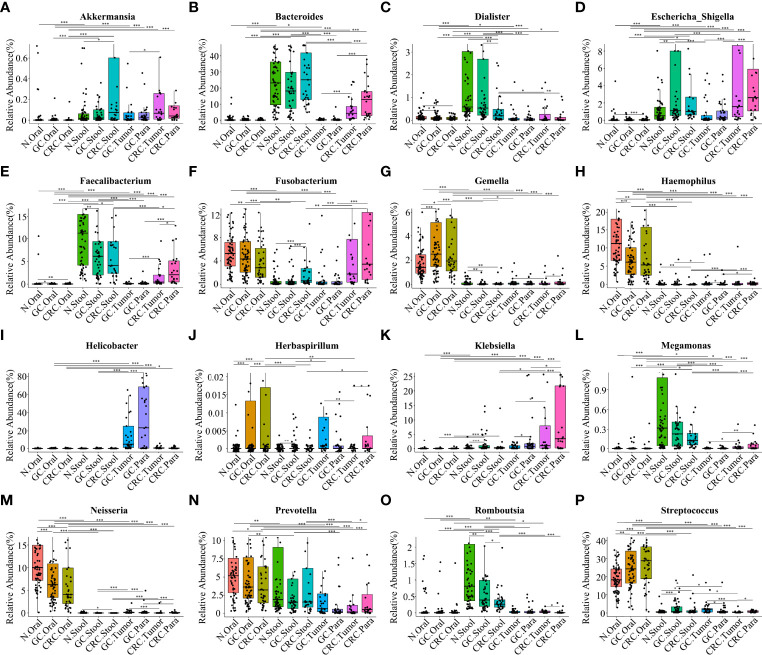
Comparison of the relative abundance of different bacterial genera (LDA > 4) among multiple groups. The results showed the different bacterial genera (LDA > 4) in the oral cavity, feces, and tissues of the HC, GC, and CRC groups: *Akkermansia* **(A)**, *Bacteroides* **(B)**, *Dialister* **(C)**, *Escherichia-Shigella* **(D)**, *Faecalibacterium* **(E)**, *Fusobacterium* **(F)**, *Gemella* ***(*G*)***, *Haemophilus* **(H)**, *Helicobacter* **(I)**, *Herbaspirillum* ***(*J*)***, *Klebsiella* ***(*K*)***, *Megamonas* **(L)**, *Neisseria* **(M)**, *Prevotella* **(N)**, *Romboutsia* **(O)**, and *Streptococcus* **(P)**. Compared with the HC oral group, the relative abundance values of *Haemophilus* **(H)** and *Neisseria* **(M)** in the oral cavity of GC and CRC patients were significantly downregulated; the relative abundance values of *Gemella* **(G)**, *Streptococcus* **(P)**, and *Herbaspirillum* **(J)** were significantly upregulated. Compared with the HC oral stool group, the relative abundance values of *Dialister* **(C)**, *Faecalibacterium* **(E)**, *Megamonas* **(L)**, and *Romboutsia* **(O)** in the stool of GC or CRC patients were significantly downregulated; the relative abundance values of *Streptococcus*, *Escherichia-Shigella* **(D)**, *Fusobacterium* **(F)**, and *Akkermansia* **(A)** were significantly upregulated. *Helicobacter* **(I)**, *Bacteroides* **(B)**, *Klebsiella* **(K)**, and *Prevotella* **(N)** are also different genera in GC and CRC tissues, and their relative abundance values have also changed. *p < 0.05, **p < 0.01, ***p < 0.001.

Based on the linear discriminant analysis effect size (LEfSe) and MetaStat analysis, we further analyzed the relative abundance values of the different bacterial genera (LDA > 4) among the groups. In comparison to oral flora of the HC group, *Streptococcus*, *Gemella*, and *Herbaspirillum* in the oral cavity of GC and CRC patients were more abundant (*p* < 0.05), while that of *Haemophilus* and *Neisseria* were less abundant (*p* < 0.05). In comparison to fecal flora of the HC group, *Escherichia*-*Shigella* and *Streptococcus* were more abundant (*p* < 0.05) in the stools of GC and CRC patients, while *Faecalibacterium*, *Dialister*, and *Romboutsia* were less abundant (*p* < 0.05). Meanwhile, *Fusobacterium* and *Akkermansia* were more abundant (*p* < 0.05) in the stool of CRC patients, and *Megamonas* was less abundant (*p* < 0.05). Interestingly, *Fusobacterium* was less abundant (*p* < 0.05) in the CRC oral cavity samples compared to the HC oral group. The different bacteria (*Bacteroides*, *Helicobacter*, *Klebsiella*, *Prevotella*, etc.) in GC and CRC tissues were also evaluated and they differed in oral, fecal, and tissue flora (**Figures 6A–P**). The majority of these diverse bacterial genera were a part of non-invasive tumor marker screening, shared microbial regulation, and network interaction. In conclusion, these differential bacteria in the oral cavity, stool, and tissue may play an active role in the GC/CRC evolution.

### Differences and Network Analysis of Co-Abundant Microbiota of Oral Cavity, Stool, and Tissue of GC and CRC

**Figure 7:**
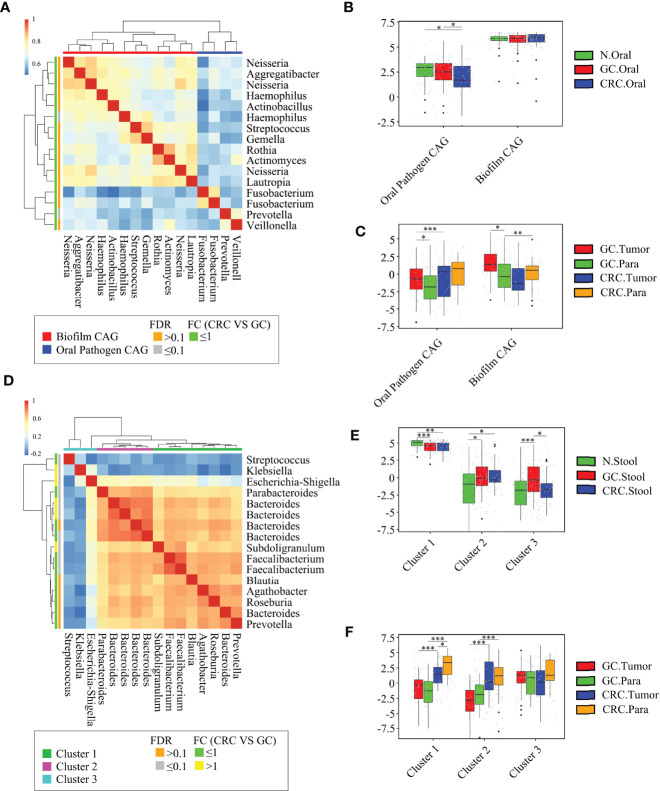
Abundant oral and fecal microbial networks were detected in GC and CRC mucosa. Analyze the shared microbiota of the oral cavity and tissue of HC, GC, and CRC participants: **(A)** 16 oral microbial OTUs associated with GC and CRC tissue were aggregated into two coabundance groups (CAGs). CAGs were defined in terms of clusters in a vertical or horizontal dendrogram and were named after their most prominent features. **(B)** Relative abundance of Oral pathogen CAG and Biofilm CAG in three groups of oral samples (HC = 70, GC = 70, and CRC = 42). **(C)** The relative abundance of Oral pathogen CAG and Biofilm CAG in GC and CRC tissue samples (GC.Tumor = 33, GC.Para = 36, CRC.Tumor = 24, and CRC.Para = 24). Analyze the shared microbiota of the stool and tissue of HC, GC, and CRC participants: **(D)** 16 fecal microbial OTUs associated with GC and CRC tissue were aggregated into three CAGs. CAGs were defined in terms of clusters in a vertical or horizontal dendrogram and were named after their most prominent features. **(E)** The relative abundance of Cluster 1, Cluster 2, and Cluster 3 in fecal samples of the three groups (HC = 70, GC = 49, and CRC = 33). **(F)** The relative abundance of Cluster 1, Cluster 2, and Cluster 3 in GC and CRC tissue samples (GC.Tumor = 33, GC.Para = 36, CRC.Tumor = 24, and CRC.Para = 24). *p < 0.05, **p < 0.01, ***p < 0.001.

**Figure 8:**
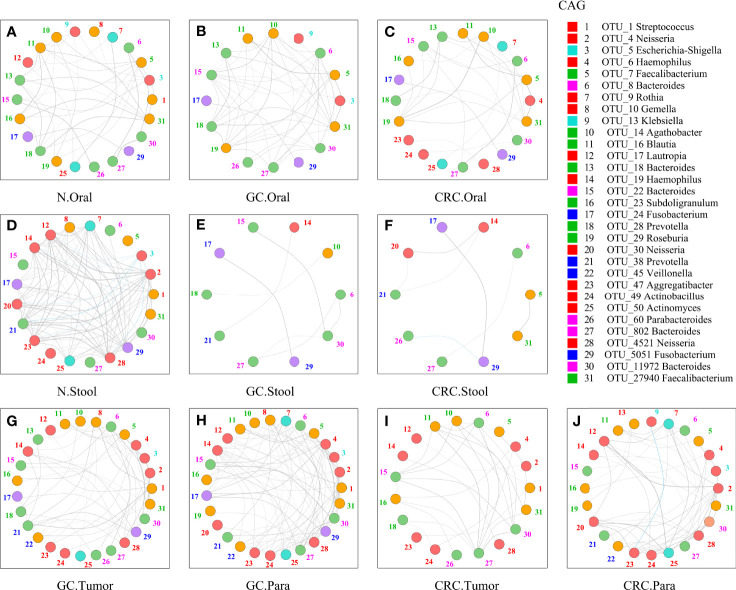
Network analysis of microbiota shared by tumor, paracancerous tissue, oral cavity, and stool in HC, GC, and CRC. The figure showed the 31-OTU (**Figure 6**) network map of bacteria found in the oral, fecal, tumor, and paracancerous mucosal microbiota of HC, GC, and CRC participants: oral samples of 70 HC **(A)**, 70 GC patients **(B)**, and 42 CRC patients **(C)**; fecal samples of 70 HC **(D)**, 49 GC patients **(E)**, and 33 CRC patients **(F)**; tumors from 33 GC patients **(G)** and 24 CRC patients **(I)**; and paracancerous tissue of 36 GC patients **(H)** and 24 CRC patients **(J)**. For each group of samples, OTUs shared with oral and fecal samples were determined respectively. The color of each nodule was divided according to its phylum, and the color of the number (the corresponding OTU) beside the nodule was only calculated using the corresponding CAG in **Figure 6** (**Figures 6A, D**). The colors of the lines between nodes (Spearman correlation value: *r* > 0.6 or <−0.6, *p* < 0.01) were divided according to correlation (gray: positive correlation, blue: negative correlation). As the Spearman correlation value between each corresponding node increased, the width of each edge increased. Legend on the right: the Mothur method and the SSUrRNA database of SILVA138 were used to perform species annotation analysis on OTUs sequences. OTU_1 was shared by Biofilm CAG and Proteobacteria CAG and shown in red.

Although some OTUs were more abundant across samples from GC/CRC patients, there was considerable heterogeneity. We analyzed the microbiota by assaying CAGs (or clusters), as community structure may provide more valuable information than differences in the abundance of individual genera. To investigate the microbiota correlation between HC, GC, and CRC in oral, fecal and tissue samples, 16 OTUs shared between oral and tissue samples of participants from the three groups were screened. These 16 OTUs were expressed in at least 30% of all oral and tissue samples from the three groups and had more than 300 reads in a single sample. In addition, 16 OTUs were shared between fecal and tissue samples from the three groups of participants, which were expressed in at least 30% of all fecal and tissue samples across all three groups, with >300 reads in a single sample (**Table S6**). The bacteria in oral and tissue samples were divided into two co-abundance groups (CAGs) based on abundance profiles (16 OTUs): oral pathogens and biofilms (**Figure 7A**). Oral pathogens (e.g., *Fusobacterium*) were pathogenic and were linked to the late colonization of oral biofilms and several human diseases (including CRC and juvenile periodontitis). *Actinomyces*, *Haemophilus*, *Rothia*, *Streptococcus*, and other genera existed in the early stage of dental biofilm formation and were associated with relatively healthy dental pockets, which were defined as Biofilm CAG (Flemer et al., 2018; Zhang et al., 2020). The oral pathogen CAG of GC and CRC was lower in oral samples than in HC (**Figure 7B**). The two groups of CAGs in the GC tumor tissue samples were higher than those in the paracancerous tissue (**Figure 7C**). The bacteria in the stool and tissue samples were clustered by abundance profiles (16 OTUs) and divided into three clusters: Cluster 1, Cluster 2, and Cluster 3 (**Figure 7D**). Cluster 1 of GC and CRC was found to decrease in the stool samples in comparison to HC, while an increasing trend was observed for Cluster 2 with a significant increase in Cluster 3 of GC (**Figure 7E**). Cluster 1 and Cluster 2 of CRC were found to be higher than that of GC in tissue samples, whereas Cluster 1 of CRC tumor was found to be lower than that of paracancerous tissue (**Figure 7F**). Furthermore, the network of the aforementioned bacteria (31 OTUs) in all samples was also analyzed. The number of bacteria and lines in HC oral and fecal samples were found to be in abundance (**Figures 8A, D**). Compared to HC, the number of oral bacteria in GC and CRC decreased (**Figures 8B, C**), while fecal bacteria decreased significantly (**Figures 8E, F**). Moreover, deletion of some OTUs (and genera) in the network resulted in significant changes in phylum and CAGs in GC/CRC patients. For example, *Actinobacteriota* (OTU_9 and OTU_50) and Biofilm CAG (OTU_1, OTU_9, OTU_10, OTU_17, OTU_50) of GC oral samples vanished completely. The bacterial network in CRC tissue was sparser (number of OTUs and lines) compared to that in GC tissue. Similar bacterial networks in tumor and paracancerous tissue may indicate that the bacterial composition of diseased organs has changed before tumor formation (**Figures 8G–J**). To further analyze the correlation between GC and CRC in oral, stool, and tissue, Venn diagrams and PCoA analysis were performed on all samples (**Figures S7A–H**).

### Identification and Validation of GC and CRC Markers Based on Oral and Fecal Microbial OTU

**Figure 9:**
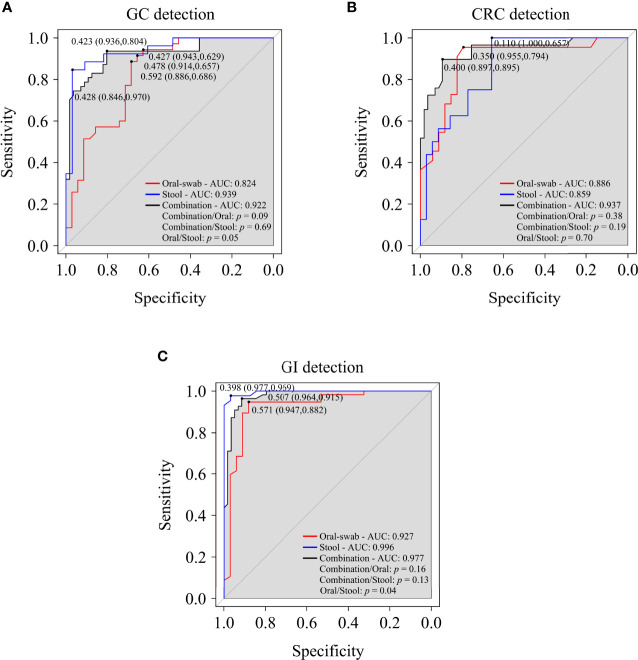
Oral and fecal microbiota spectrum may be a new non-invasive detection for GC, CRC, and gastrointestinal (GI) cancers. Through the analysis the AUC of POD index, and using microbiota profiles from oral swabs, stool, or a combination of both to predict GC **(A)**, CRC **(B)**, and GI cancers **(C)**. ROC, receiver operating characteristic curves; AUC, area under the curve.

A random forest classifier model to determine the diagnostic value of oral and fecal microbiota for GC, CRC, and GI cancers were generated. A 5-fold cross-validation was used to achieve specific cancer identification (**Table S7**). In the prediction of GC using oral and stool samples, in the discovery cohort, we found the optimal OTU marker set (oral: 13 OTUs, stool: 9 OTUs) and calculated their POD and area under the curve (AUC) (**Figures S8A–C, H–J**). In the validation cohort, the AUC of POD index between the GC and HC was found to be 82.40% (95% CI: 72.60–92.20%) for oral microbiota, 93.90% (95% CI: 87.70–100%) for fecal microbiota, and 92.20% (95% CI: 86.80–97.70%) for combined detection (**Figures 9A**, **S8D, E, K–L**). The diagnostic potential of the model was further confirmed by all GC samples, which showed an AUC of 94.90% (95% CI: 91.50–98.30%) for all oral samples and 97.70% (95% CI: 95.20–100%) for all fecal samples (**Figures S8F, G, M, N**). In the discovery cohort of CRC’s oral and fecal samples, we identified the optimal OTU marker set (oral: 9 OTUs, stool: 6 OTUs) and calculated their POD and AUCs (**Figures S9A–C, H–J**). In the validation cohort, the AUC was found to be 88.60% (95% CI: 79.10–98.20%) for oral samples, 85.90% (95% CI: 75.80–96.00%) for fecal samples, and 93.70% (95% CI: 87.90–99.40%) for combined detection (**Figures 9B**, **S9D, E, K, L**). The AUC of all CRC oral samples was found to be 96.40% (95% CI: 92.90–99.90%), and the AUC of all fecal samples was determined to be 96.30% (95% CI: 93.30–99.30%) (**Figures S9F, G, M, N**). The GC and CRC samples were integrated, and respective POD and AUC were calculated for the predictive potential of oral and fecal microbiota for GI cancers. In the validation cohort, using the optimal OTU marker set (oral: 20 OTUs, stool: 20 OTUs) for GI samples, the AUC was 92.70% (95% CI: 96.30–99.20%) for oral samples, 99.60% (95% CI: 98.80–100%) for fecal samples, and 97.70% (95% CI: 95.40–100%) for combined detection (**Figure 9C**, **S10A–E, H–L**). The AUC of all GI oral samples was found to be 97.50% (95% CI: 95.00–100%), and the AUC of all stool samples was 99.90% (95% CI: 99.70–100%) (**Figures S10F, G, M, N**). According to these findings, POD based on microbial OTU markers in oral and fecal samples had the high predictive potential for GC, CRC, and GI cancers. In addition, combined detection outperformed oral or fecal detection alone in terms of CRC prediction.

## Discussion

In this study, a combined analysis of GC and CRC was performed, demonstrating the commonality and specificity of microbiota in the oral cavity, stool, tumor, and paracancerous tissue. Compared to HC, GC and CRC showed the same changes in the richness, evenness, and number of bacterial species, revealing that the indicators decreased in oral samples and increased in fecal samples. Similar changes were observed in the α-diversity. Microbial dysbiosis may be caused by a decrease in low-abundance bacteria and a change in their composition in GC and CRC oral samples and an increase in microbial diversity in fecal and tumor samples. Meanwhile, compared to HC, the GC *Proteobacteria* and CRC *Fusobacteriota* content decreased in oral samples (LDA > 4), while they increased in fecal samples (LDA > 4). At the genus level, compared to HC, the content of *Haemophilus* and *Neisseria* of GC and CRC decreased, while the content of *Streptococcus* and *Herbaspirillum* increased in oral samples (LDA > 4); in addition, the content of GC’s *Streptococcus* and *Escherichia-Shigella* and CRC’s *Fusobacterium* and *Escherichia-Shigella* increased in stool samples (LDA > 4). Through the comparison of the three algorithms of Lefse, RF, and PLSDA-VIP, these shared differential genera (GC: *Gemella* and *Lactobacillus* and CRC: *Streptococcus*, *Peptostreptococcus*, etc.) were found, and may play a role in GC/CRC. We also further compared the relative abundance of different bacterial genera (LDA > 4) (*Haemophilus*, *Neisseria*, *Faecalibacterium*, *Romboutsia*, *Streptococcus*, *Gemella*, *Escherichia-Shigella*, *Fusobacterium*, etc.) in the oral cavity, stool, and tissue among multiple groups. The results suggested that they could be used as potential biomarkers to participate in the evolution of tumors as passengers or drivers. However, the significance of the synergistic or antagonistic relationship between microbiota must be investigated further. These oral and fecal bacterial changes appear to be regular, and further research into their interactions may provide new insights into tumorigenesis. Furthermore, significant differences from HC bacteria served as a foundation for GC and CRC-specific prediction.

The most prevalent species in the human stomach is *H. pylori*, which accounts for 72%–99% of sequencing reads (Hunt et al., 2015). This study showed that the sequencing reads of *Helicobacter* genus in tumor (<25%) and paracancerous tissue (<50%) implies that other bacteria may play more important biological functions in GC. Although the acidic environment of a healthy stomach is not conducive to bacterial growth, the bacterial richness and several GC α indexes were higher in the tissue samples than those of CRC, indicating a change in GC tissue environment and rich microbial diversity. *Streptococcus* was found to be more abundant in GC tumors than in paracancerous tissue, and *Fusobacteriota* and *Fusobacterium* were found to be more prevalent in CRC tissue (Flemer et al., 2018). These potential rules of bacterial changes in the oral cavity, stool, and tissue may be linked to the evolution of GI tumors, but the role of passenger or driver needs to be further explored (Sepich-Poore et al., 2021). The *Fusobacterium nucleatum* is involved in the occurrence and development mechanism of CRC at the moment (Yang et al., 2017; Brennan and Garrett, 2019).

We observed that the bacteria on tumor mucosa of GC and CRC were more closely related to oral bacteria by analyzing the shared bacteria and networks between oral cavity, stool, and tissue of all samples and that distant colonization of oral microbiota may promote this close relationship (Flemer et al., 2018; Mascitti et al., 2019; Koliarakis et al., 2019; Stasiewicz and Karpiński, 2021). The GC and CRC differ from their precancerous lesions in microbial diversity, and bacteria can influence the risk of inflammation and cancer in the host by participating in purine metabolism, carbohydrate metabolism, peptidoglycan biosynthesis (Grivennikov et al., 2012; Coker et al., 2018), and interaction with the immune system (such as Treg cells) (Furusawa et al., 2013). The network map also showed significant changes in the phylum and CAGs within each group. These results suggested that the composition and abundance of microbiota constantly change in different disease states and sites and may actively participate in disease evolution (Abreu and Peek, 2014; Engstrand and Graham, 2020; LaCourse et al., 2021; Sepich-Poore et al., 2021).

The GC is an inflammatory-related cancer that is commonly classified into cardia and non-cardia; factors that increase the risk of non-cardia GC include chronic *H. pylori* infection, smoking, alcohol consumption, pickled food, barbecue, low fruit intake, etc. (Engstrand and Graham, 2020; Sung et al., 2021). A high-fat diet and a sedentary lifestyle increase the CRC risk (Sung et al., 2021). Primary prevention remains an important strategy for reducing the global burden of GC and CRC (Song et al., 2020; Sung et al., 2021). According to clinical data, education, smoking, and family income may also be associated with the prevalence of GC and CRC. Our results and those of several other studies suggested that improving dietary intake may be beneficial in the prevention of GC and CRC (Tsugane and Sasazuki, 2007; Claesson et al., 2012; Etemadi et al., 2020). In this study, a random forest model and POD calculation were used to classify oral and fecal microbiota of GC, CRC, and GI cancers, which indicated that the microbial markers could be used as a non-invasive predictive tool for them. A more effective, non-invasive, convenient, and cost-effective screening program may be implemented as a primary preventive measure.

Since this study was primarily based on a high-risk elderly cohort, appropriately lowering the screening age (down to 45 years old) could help reduce the burden of GC and CRC (Wolf et al., 2018). Microbiota research in the pathogenesis of systemic diseases, metabolomics analysis, and multi-center large-sample verification is in the early stages. Biomarker analysis failed to resolve to the species level and better identify species (e.g., *Shigella* spp. and *Escherichia coli*) (Devanga Ragupathi et al., 2018). Various sequencing analyses and algorithms (such as DADA2) also need to be updated and compared in time to better present the results. Longitudinal studies remain required to determine the role of oral, fecal, and GI biomarkers in tumor development. We hope to provide a new idea for the difficulties of targeted therapy in GI cancer’s intratumoral, intra-patient, and inter-patient heterogeneity by further studying the relationship between microbiota and GI cancers and applying it to clinical diagnosis and treatment (Smyth et al., 2020).

## Data Availability statement

The original contributions presented in the study are publicly available. These data can be found here: https://www.ncbi.nlm.nih.gov/sra/PRJNA778008.

## Ethics Statement

This study was approved by the Committee on Medical Ethics of the First Affiliated Hospital of Anhui Medical University (Quick-PJ 2021-13-23). The patients/participants provided their written informed consent to participate in this study.

## Author Contributions

Study concept and design: CZ and YoL. Specimen provider: CZ, AH, and YaL. Analysis and interpretation of data: CZ, AH, and JL. Technical and material support: YoL, CZ, and PZ. Drafting of the manuscript: CZ. All authors contributed to the article and approved the submitted version.

## Funding

This work was supported by grants from the National Natural Science Foundation of China (No. 81874063) and the Natural Science Foundation of Anhui Province (No. 2008085QH408).

## Conflict of Interest

The authors declare that the research was conducted in the absence of any commercial or financial relationships that could be construed as a potential conflict of interest.

## Publisher’s Note

All claims expressed in this article are solely those of the authors and do not necessarily represent those of their affiliated organizations, or those of the publisher, the editors and the reviewers. Any product that may be evaluated in this article, or claim that may be made by its manufacturer, is not guaranteed or endorsed by the publisher.

## Abbreviations

CRC, colorectal cancer; GC, gastric cancer; GI, gastrointestinal; EGC, early gastric cancer; NBI, narrowband imaging; MCCG, magnetic controlled capsule gastroscopy; SG, superficial gastritis; AG, atrophic gastritis; IM, intestinal metaplasia; HC, healthy controls; CT, computed tomography; FOBT, fecal occult blood test; FIT, fecal immune test; PRoBE, prospective specimen collection and retrospective blind evaluation; FFQ, food frequency questionnaire; IBS, irritable bowel syndrome; IBD, inflammatory bowel disease; PCoA, principal coordinate analysis; POD, probability of disease; ANOVA, analysis of variance; FDR, false discovery rate; BMI, body mass index; RMB, renminbi; CSS, cumulative-sum scaling; LDA, linear discriminant analysis; LEfSe, linear discriminant analysis effect size; RF, random forest; ROC, receiver operating characteristic; AUC, area under the curve; OTUs, operational taxonomic units; CAGs, co-abundance groups.

## References

[B1] Abreu M. T. Peek R. M. Jr. (2014). Gastrointestinal Malignancy and the Microbiome. Gastroenterology 146, 1534–1546.e3. doi: 10.1053/j.gastro.2014.01.001 24406471 PMC3995897

[B2] Andersson A. F. Lindberg M. Jakobsson H. Bäckhed F. Nyrén P. Engstrand L. (2008). Comparative Analysis of Human Gut Microbiota by Barcoded Pyrosequencing. PLoS One 3, e2836. doi: 10.1371/journal.pone.0002836 18665274 PMC2475661

[B3] Brennan C. A. Garrett W. S. (2019). Fusobacterium Nucleatum - Symbiont, Opportunist and Oncobacterium. Nat. Rev. Microbiol. 17, 156–166. doi: 10.1038/s41579-018-0129-6 30546113 PMC6589823

[B4] Caporaso J. G. Kuczynski J. Stombaugh J. Bittinger K. Bushman F. D. Costello E. K.. (2010). QIIME Allows Analysis of High-Throughput Community Sequencing Data. Nat. Methods 7, 335–336. doi: 10.1038/nmeth.f.303 20383131 PMC3156573

[B5] Chen X. Winckler B. Lu M. Cheng H. Yuan Z. Yang Y.. (2015). Oral Microbiota and Risk for Esophageal Squamous Cell Carcinoma in a High-Risk Area of China. PLoS One 10, e0143603. doi: 10.1371/journal.pone.0143603 26641451 PMC4671675

[B6] Claesson M. J. Jeffery I. B. Conde S. Power S. E. O'Connor E. M. Cusack S.. (2012). Gut Microbiota Composition Correlates With Diet and Health in the Elderly. Nature 488, 84–178. doi: 10.1038/nature11319 22797518

[B7] Coker O. O. Dai Z. Nie Y. Zhao G. Cao L. Nakatsu G.. (2018). Mucosal Microbiome Dysbiosis in Gastric Carcinogenesis. Gut 67, 1024–1032. doi: 10.1136/gutjnl-2017-314281 28765474 PMC5969346

[B8] Devanga Ragupathi N. K. Muthuirulandi Sethuvel D. P. Inbanathan F. Y. Veeraraghavan B. (2018). Accurate Differentiation of Escherichia Coli and Shigella Serogroups: Challenges and Strategies. New Microbes New Infect. 21, 58–62. doi: 10.1016/j.nmni.2017.09.003 29204286 PMC5711669

[B9] Du Y. Zhu H. Liu J. Li J. Chang X. Zhou L.. (2020). Consensus on Eradication of Helicobacter Pylori and Prevention and Control of Gastric Cancer in China (2019, Shanghai). J. Gastroenterol. Hepatol. 35, 624–629. doi: 10.1111/jgh.14947 31788864

[B10] Edgar R. C. (2004). MUSCLE: Multiple Sequence Alignment With High Accuracy and High Throughput. Nucleic Acids Res. 32, 1792–1797. doi: 10.1093/nar/gkh340 15034147 PMC390337

[B11] Edgar R. C. Haas B. J. Clemente J. C. Quince C. Knight R. (2011). UCHIME Improves Sensitivity and Speed of Chimera Detection. Bioinformatics 27, 2194–2200. doi: 10.1093/bioinformatics/btr381 21700674 PMC3150044

[B12] Engstrand L. Graham D. Y. (2020). Microbiome and Gastric Cancer. Digest. Dis. Sci. 65, 865–873. doi: 10.1007/s10620-020-06101-z 32040665 PMC8697197

[B13] Etemadi A Safiri S Sepanlou SG Ikuta K Bisignano C Shakeri R. (2020). The Global, Regional, and National Burden of Stomach Cancer in 195 Countries, 1990-2017: A Systematic Analysis for the Global Burden of Disease Study 2017. Lancet Gastroenterol. Hepatol. 5, 42–54. doi: 10.1016/S2468-1253(19)30328-0 31648970 PMC7033564

[B14] Eusebi L. H. Telese A. Marasco G. Bazzoli F. Zagari R. M. (2020). Gastric Cancer Prevention Strategies: A Global Perspective. J. Gastroenterol. Hepatol. 35, 1495–1502. doi: 10.1111/jgh.15037 32181516

[B15] Farrell J. J. Zhang L. Zhou H. Chia D. Elashoff D. Akin D.. (2012). Variations of Oral Microbiota are Associated With Pancreatic Diseases Including Pancreatic Cancer. Gut 61, 582–588. doi: 10.1136/gutjnl-2011-300784 21994333 PMC3705763

[B16] Flemer B. Lynch D. B. Brown J. M. Jeffery I. B. Ryan F. J. Claesson M. J.. (2017). Tumour-Associated and non-Tumour-Associated Microbiota in Colorectal Cancer. Gut 66, 633–643. doi: 10.1136/gutjnl-2015-309595 26992426 PMC5529966

[B17] Flemer B. Warren R. D. Barrett M. P. Cisek K. Das A. Jeffery I. B.. (2018). The Oral Microbiota in Colorectal Cancer is Distinctive and Predictive. Gut 67, 1454–1463. doi: 10.1136/gutjnl-2017-314814 28988196 PMC6204958

[B18] Furusawa Y. Obata Y. Fukuda S. Endo T. A. Nakato G. Takahashi D.. (2013). Commensal Microbe-Derived Butyrate Induces the Differentiation of Colonic Regulatory T Cells. Nature 504, 446–450. doi: 10.1038/nature12721 24226770

[B19] Gevers D. Knight R. Petrosino J. F. Huang K. McGuire A. L. Birren B. W.. (2012). The Human Microbiome Project: A Community Resource for the Healthy Human Microbiome. PLoS Biol. 10 (8), e1001377. doi: 10.1371/journal.pbio.1001377 22904687 PMC3419203

[B20] Grady W. M. Yu M. Markowitz S. D. (2021). Epigenetic Alterations in the Gastrointestinal Tract: Current and Emerging Use for Biomarkers of Cancer. Gastroenterology 160, 690–709. doi: 10.1053/j.gastro.2020.09.058 33279516 PMC7878343

[B21] Graham D. Y. (2015). Helicobacter Pylori Update: Gastric Cancer, Reliable Therapy, and Possible Benefits. Gastroenterology 148, 719–31.e3. doi: 10.1053/j.gastro.2015.01.040 25655557 PMC4375058

[B22] Grivennikov S. I. Wang K. Mucida D. Stewart C. A. Schnabl B. Jauch D.. (2012). Adenoma-Linked Barrier Defects and Microbial Products Drive IL-23/IL-17-Mediated Tumour Growth. Nature 491, 254–258. doi: 10.1038/nature11465 23034650 PMC3601659

[B23] Huang Y. K. Yu J. C. Kang W. M. Ma Z. Q. Ye X. Tian S. B.. (2015). Significance of Serum Pepsinogens as a Biomarker for Gastric Cancer and Atrophic Gastritis Screening: A Systematic Review and Meta-Analysis. PLoS One 10, e0142080. doi: 10.1371/journal.pone.0142080 26556485 PMC4640555

[B24] Hunt R. H. Camilleri M. Crowe S. E. El-Omar E. M. Fox J. G. Kuipers E. J.. (2015). The Stomach in Health and Disease. Gut 64, 1650–1668. doi: 10.1136/gutjnl-2014-307595 26342014 PMC4835810

[B25] Issa I. A. Noureddine M. (2017). Colorectal Cancer Screening: An Updated Review of the Available Options. World J. Gastroenterol. 23, 5086–5096. doi: 10.3748/wjg.v23.i28.5086 28811705 PMC5537177

[B26] Koliarakis I. Messaritakis I. Nikolouzakis T. K. Hamilos G. Souglakos J. Tsiaoussis J. (2019). Oral Bacteria and Intestinal Dysbiosis in Colorectal Cancer. Int. J. Mol. Sci. 20(17), 4146. doi: 10.3390/ijms20174146 PMC6747549 31450675

[B27] LaCourse K. D. Johnston C. D. Bullman S. (2021). The Relationship Between Gastrointestinal Cancers and the Microbiota. Lancet Gastroenterol. Hepatol. 6, 498–509. doi: 10.1016/S2468-1253(20)30362-9 33743198 PMC10773981

[B28] Liu S. Dai J. Lan X. Fan B. Dong T. Zhang Y.. (2021). Intestinal Bacteria are Potential Biomarkers and Therapeutic Targets for Gastric Cancer. Microb. Pathog. 151, 104747. doi: 10.1016/j.micpath.2021.104747 33484807

[B29] Magoc T. Salzberg S. L. (2011). FLASH: Fast Length Adjustment of Short Reads to Improve Genome Assemblies. Bioinformatics 27, 2957–2963. doi: 10.1093/bioinformatics/btr507 21903629 PMC3198573

[B30] Mascitti M. Togni L. Troiano G. Caponio V. C. A. Gissi D. B. Montebugnoli L.. (2019). Beyond Head and Neck Cancer: The Relationship Between Oral Microbiota and Tumour Development in Distant Organs. Front. Cell. Infect. Microbiol. 9, 232. doi: 10.3389/fcimb.2019.00232 31297343 PMC6607058

[B31] Peek R. M. Jr. Crabtree J. E. (2006). Helicobacter Infection and Gastric Neoplasia. J. Pathol. 208, 233–248. doi: 10.1002/path.1868 16362989

[B32] Pepe M. S. Feng Z. Janes H. Bossuyt P. M. Potter J. D. (2008). Pivotal Evaluation of the Accuracy of a Biomarker Used for Classification or Prediction: Standards for Study Design. J. Natl. Cancer Inst. 100, 1432–1438. doi: 10.1093/jnci/djn326 18840817 PMC2567415

[B33] Pfaffe T. Cooper-White J. Beyerlein P. Kostner K. Punyadeera C. (2011). Diagnostic Potential of Saliva: Current State and Future Applications. Clin. Chem. 57, 675–687. doi: 10.1373/clinchem.2010.153767 21383043

[B34] Pimentel-Nunes P. Dinis-Ribeiro M. Soares J. B. Marcos-Pinto R. Santos C. Rolanda C.. (2012). A Multicenter Validation of an Endoscopic Classification With Narrow Band Imaging for Gastric Precancerous and Cancerous Lesions. Endoscopy 44, 236–246. doi: 10.1055/s-0031-1291537 22294194

[B35] Pushalkar S. Ji X. Li Y. Estilo C. Yegnanarayana R. Singh B.. (2012). Comparison of Oral Microbiota in Tumor and non-Tumor Tissues of Patients With Oral Squamous Cell Carcinoma. BMC Microbiol. 12, 144. doi: 10.1186/1471-2180-12-144 22817758 PMC3507910

[B36] Qin N. Yang F. L. Li A. Prifti E. Chen Y. F. Shao L.. (2014). Alterations of the Human Gut Microbiome in Liver Cirrhosis. Nature 513, 59–64. doi: 10.1038/nature13568 25079328

[B37] Quast C. Pruesse E. Yilmaz P. Gerken J. Schweer T. Yarza P.. (2013). The SILVA Ribosomal RNA Gene Database Project: Improved Data Processing and Web-Based Tools. Nucleic Acids Res. 41, D590–D596. doi: 10.1093/nar/gks1219 23193283 PMC3531112

[B38] Ren Z. G. Li A. Jiang J. W. Zhou L. Yu Z. J. Lu H. F.. (2019). Gut Microbiome Analysis as a Tool Towards Targeted non-Invasive Biomarkers for Early Hepatocellular Carcinoma. Gut 68, 1014–1023. doi: 10.1136/gutjnl-2017-315084 30045880 PMC6580753

[B39] Schmidt B. L. Kuczynski J. Bhattacharya A. Huey B. Corby P. M. Queiroz E. L.. (2014). Changes in Abundance of Oral Microbiota Associated With Oral Cancer. PLoS One 9, e98741. doi: 10.1371/journal.pone.0098741 24887397 PMC4041887

[B40] Segata N. Haake S. K. Mannon P. Lemon K. P. Waldron L. Gevers D.. (2012). Composition of the Adult Digestive Tract Bacterial Microbiome Based on Seven Mouth Surfaces, Tonsils, Throat and Stool Samples. Genome Biol. 13, R42. doi: 10.1186/gb-2012-13-6-r42 22698087 PMC3446314

[B41] Sepich-Poore G. D. Zitvogel L. Straussman R. Hasty J. Wargo J. A. Knight R. (2021). The Microbiome and Human Cancer. Science (New York N. Y.) 371(6536), eabc4552. doi: 10.1126/science.abc4552 PMC8767999 33766858

[B42] Shao D. T. Vogtmann E. Liu A. Q. Qin J. J. Chen W. Abnet C. C.. (2019). Microbial Characterization of Esophageal Squamous Cell Carcinoma and Gastric Cardia Adenocarcinoma From a High-Risk Region of China. Cancer 125, 3993–4002. doi: 10.1002/cncr.32403 31355925 PMC7285383

[B43] Smyth E. C. Nilsson M. Grabsch H. I. van Grieken N. C. Lordick F. (2020). Gastric Cancer. Lancet (Lond. England) 396, 635–648. doi: 10.1016/S0140-6736(20)31288-5 32861308

[B44] Song M. Chan A. T. Sun J. (2020). Influence of the Gut Microbiome, Diet, and Environment on Risk of Colorectal Cancer. Gastroenterology 158, 322–340. doi: 10.1053/j.gastro.2019.06.048 31586566 PMC6957737

[B45] Stasiewicz M. Karpiński T. M. (2021). The Oral Microbiota and its Role in Carcinogenesis. Semin. Cancer Biol. S1044-579X(21), 00269–8. doi: 10.1016/j.semcancer.2021.11.002 34743032

[B46] Sugano K. Tack J. Kuipers E. J. Graham D. Y. El-Omar E. M. Miura S.. (2015). Kyoto Global Consensus Report on Helicobacter Pylori Gastritis. Gut 64, 1353–1367. doi: 10.1136/gutjnl-2015-309252 26187502 PMC4552923

[B47] Sung H. Ferlay J. Siegel R. L. Laversanne M. Soerjomataram I. Jemal A.. (2021). Global Cancer Statistics 2020: GLOBOCAN Estimates of Incidence and Mortality Worldwide for 36 Cancers in 185 Countries. CA: Cancer J. Clin. 71, 209–249. doi: 10.3322/caac.21660 33538338

[B48] Terrisse S. Derosa L. Iebba V. Ghiringhelli F. Vaz-Luis I. Kroemer G.. (2021). Intestinal Microbiota Influences Clinical Outcome and Side Effects of Early Breast Cancer Treatment. Cell Death Differ. 28, 2778–2796. doi: 10.1038/s41418-021-00784-1 33963313 PMC8408230

[B49] Torres P. J. Fletcher E. M. Gibbons S. M. Bouvet M. Doran K. S. Kelley S. T. (2015). Characterization of the Salivary Microbiome in Patients With Pancreatic Cancer. PeerJ 3, e1373. doi: 10.7717/peerj.1373 26587342 PMC4647550

[B50] Tsugane S. Sasazuki S. (2007). Diet and the Risk of Gastric Cancer: Review of Epidemiological Evidence. Gastric Cancer 10, 75–83. doi: 10.1007/s10120-007-0420-0 17577615

[B51] Wolf A. M. D. Fontham E. T. H. Church T. R. Flowers C. R. Guerra C. E. LaMonte S. J.. (2018). Colorectal Cancer Screening for Average-Risk Adults: 2018 Guideline Update From the American Cancer Society. CA: Cancer J. Clin. 68, 250–281. doi: 10.3322/caac.21457 29846947

[B52] Yaghoobi M. McNabb-Baltar J. Bijarchi R. Hunt R. H. (2017). What is the Quantitative Risk of Gastric Cancer in the First-Degree Relatives of Patients? A Meta-Analysis. World J. Gastroenterol. 23, 2435–2442. doi: 10.3748/wjg.v23.i13.2435 28428723 PMC5385410

[B53] Yang Y. Weng W. Peng J. Hong L. Yang L. Toiyama Y.. (2017). Fusobacterium Nucleatum Increases Proliferation of Colorectal Cancer Cells and Tumor Development in Mice by Activating Toll-Like Receptor 4 Signaling to Nuclear Factor-κb, and Up-Regulating Expression of MicroRNA-21. Gastroenterology 152, 851–866.e24. doi: 10.1053/j.gastro.2016.11.018 27876571 PMC5555435

[B54] Yonekura S. Terrisse S. Alves Costa Silva C. Lafarge A. Iebba V. Ferrere G.. (2021). Cancer Induces a Stress Ileopathy Depending on B-Adrenergic Receptors and Promoting Dysbiosis That Contribute to Carcinogenesis. Cancer Discov 12 (4), 1128–1151. doi: 10.1158/2159-8290.CD-21-0999 34930787

[B55] Zagari R. M. Rabitti S. Greenwood D. C. Eusebi L. H. Vestito A. Bazzoli F. (2017). Systematic Review With Meta-Analysis: Diagnostic Performance of the Combination of Pepsinogen, Gastrin-17 and Anti-Helicobacter Pylori Antibodies Serum Assays for the Diagnosis of Atrophic Gastritis. Aliment Pharmacol. Ther. 46, 657–667. doi: 10.1111/apt.14248 28782119

[B56] Zhang S. Kong C. Yang Y. Cai S. Li X. Cai G.. (2020). Human Oral Microbiome Dysbiosis as a Novel non-Invasive Biomarker in Detection of Colorectal Cancer. Theranostics 10, 11595–11606. doi: 10.7150/thno.49515 33052235 PMC7545992

[B57] Zhang X. Li C. Cao W. Zhang Z. (2021). Alterations of Gastric Microbiota in Gastric Cancer and Precancerous Stages. Front. Cell. Infect. Microbiol. 11, 559148. doi: 10.3389/fcimb.2021.559148 33747975 PMC7966516

[B58] Zhao A. J. Qian Y. Y. Sun H. Hou X. Pan J. Liu X.. (2018). Screening for Gastric Cancer With Magnetically Controlled Capsule Gastroscopy in Asymptomatic Individuals. Gastrointest. Endosc. 88, 466–474.e1. doi: 10.1016/j.gie.2018.05.003 29753039

