# Supplementary material for: Combined Non-Invasive Prediction and New Biomarkers of Oral and Fecal Microbiota in Patients With Gastric and Colorectal Cancer
Source: Front Cell Infect Microbiol. 2022 May 19;12:830684. doi: 10.3389/fcimb.2022.830684 (PMC9161364; doi:10.3389/fcimb.2022.830684)
Supplement: Supplementary file 1 [file DataSheet_1.zip › Supplementary Figures.pdf]

Supplementary Figure 1

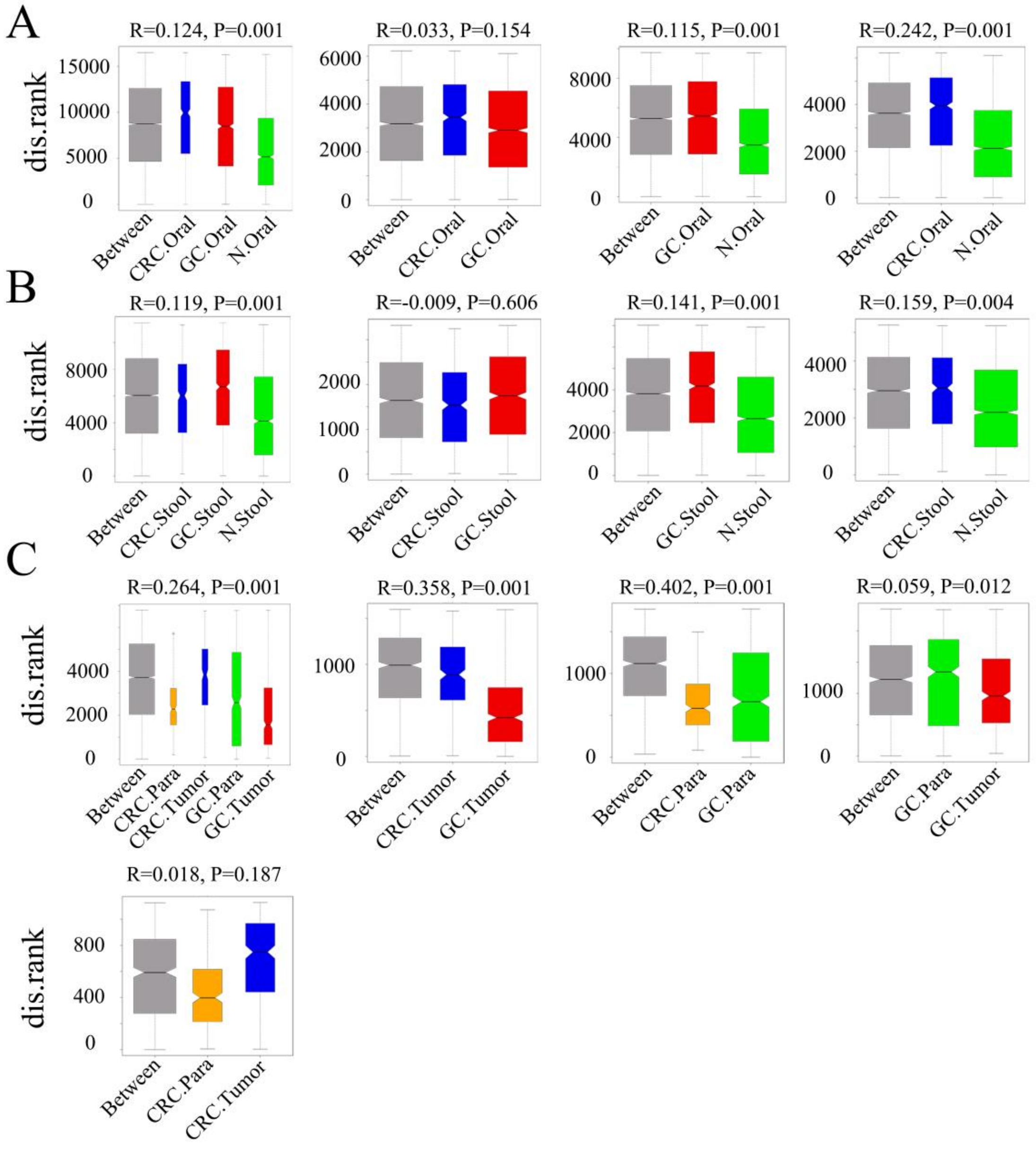

Figure S1. Group separation comparison of three groups of participants (HC, GC, CRC). (A) The cohort separation among the three groups of oral samples was compared pairwise by the ANOSIM algorithm. (B) The cohort separation among the three groups of fecal samples was compared pairwise by the ANOSIM algorithm. (C) The cohort separation between GC/CRC tissue samples was compared pairwise by the ANOSIM algorithm.

Supplementary Figure 2

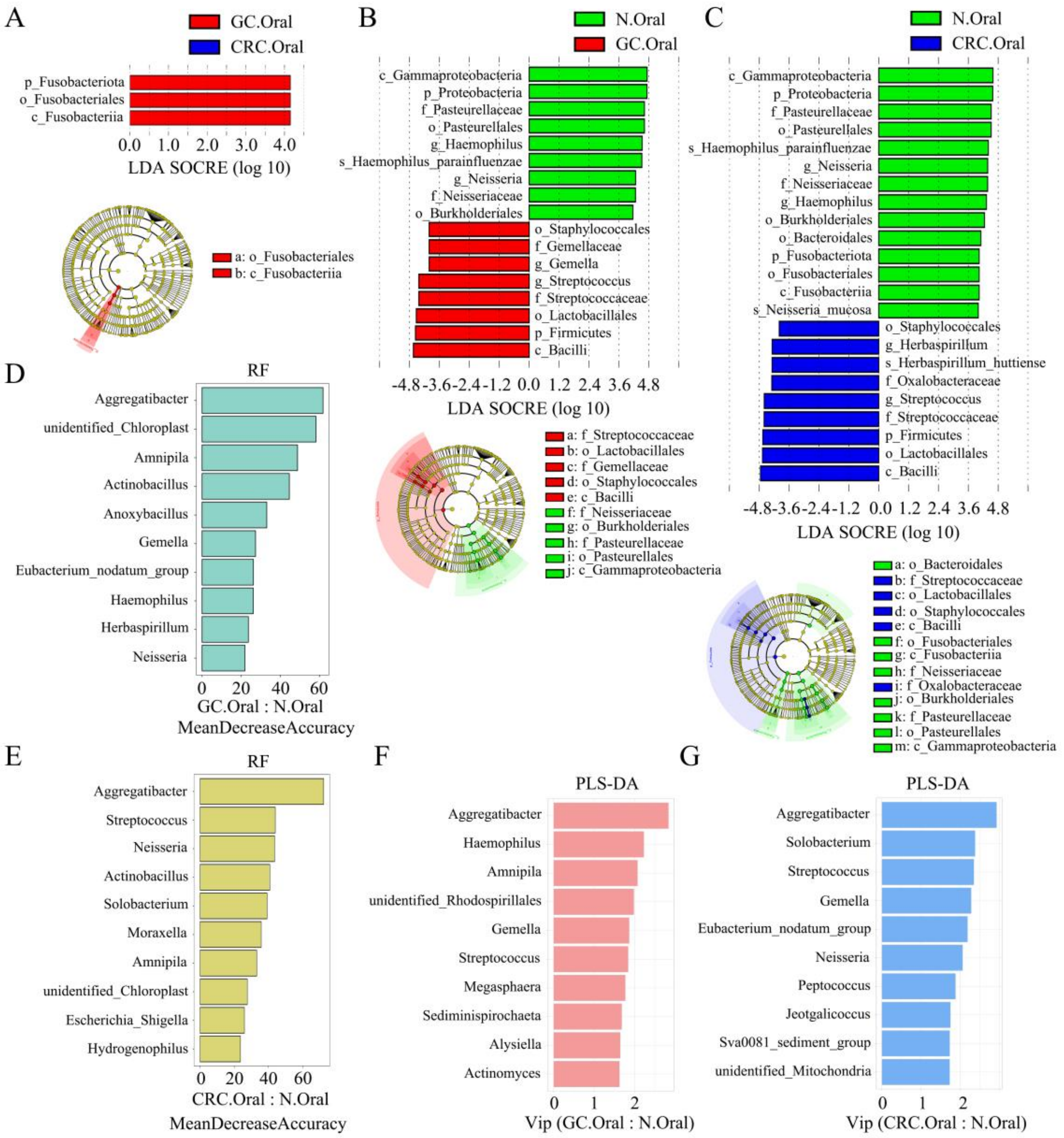

Figure S2. Comparison of the differential oral microbiota between the two groups of participants in the three groups (HC, GC, CRC). (A) The LEfSe method identified the most divergent taxa in GC and CRC and scored the two groups of oral samples by LDA. (B) The LEfSe method identified the most divergent taxa in HC and GC and scored both groups of oral samples by LDA. (C) The LEfSe method identified the most divergent taxa in HC and CRC and scored both groups of oral samples by LDA. Only the taxa that reach the effective threshold of LDA >4 were displayed. The brightness of each point was proportional to the size of its effect. (D) The top ten important differential bacterial genera in GC and HC oral samples were calculated by random forest (RF) model. (E) The top ten important differential bacterial genera in CRC and HC oral samples were calculated by random forest model. (F) The top ten important differential bacterial genera in GC and HC oral samples were calculated by PLSDA-VIP. (G) The top ten important differential bacterial genera in CRC and HC oral samples were calculated by PLSDA-VIP.

Supplementary Figure 3

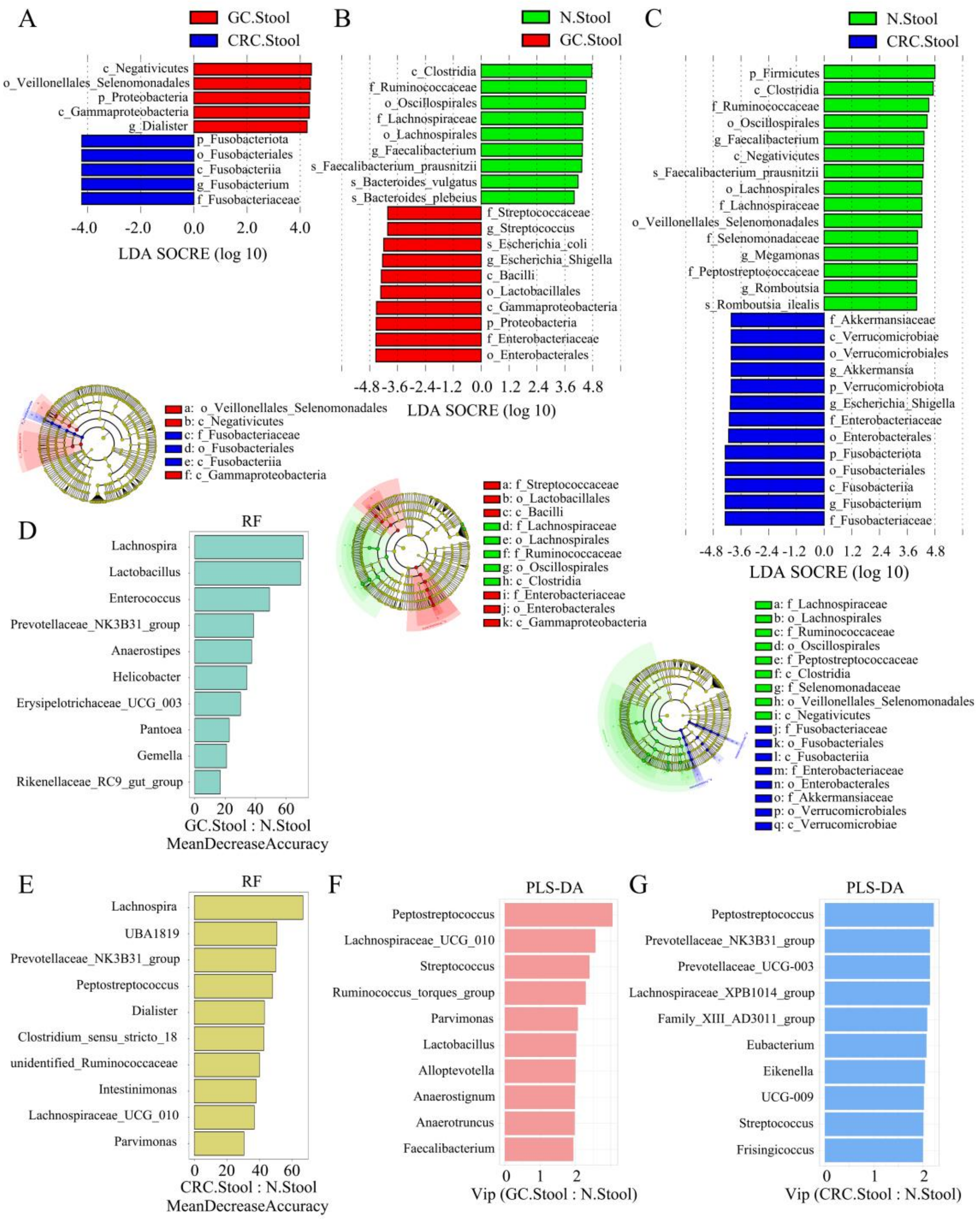

Figure S3. Comparison of the differential fecal microbiota between the two groups of participants in the three groups (HC, GC, CRC). (A) The LEfSe method identified the most divergent taxa in GC and CRC and scored the two groups of fecal samples by LDA. (B) The LEfSe method identified the most divergent taxa in HC and GC and scored both groups of fecal samples by LDA. (C) The LEfSe method identified the most divergent taxa in HC and CRC and scored both groups of fecal samples by LDA. Only the taxa that reach the effective threshold of LDA >4 were displayed. The brightness of each point was proportional to the size of its effect. (D) The top ten important differential bacterial genera in GC and HC oral samples were calculated by random

forest (RF) model. (E) The top ten important differential bacterial genera in CRC and HC oral samples were calculated by random forest model. (F) The top ten important differential bacterial genera in GC and HC oral samples were calculated by PLSDA-VIP. (G) The top ten important differential bacterial genera in CRC and HC oral samples were calculated by PLSDA-VIP.

Supplementary Figure 4

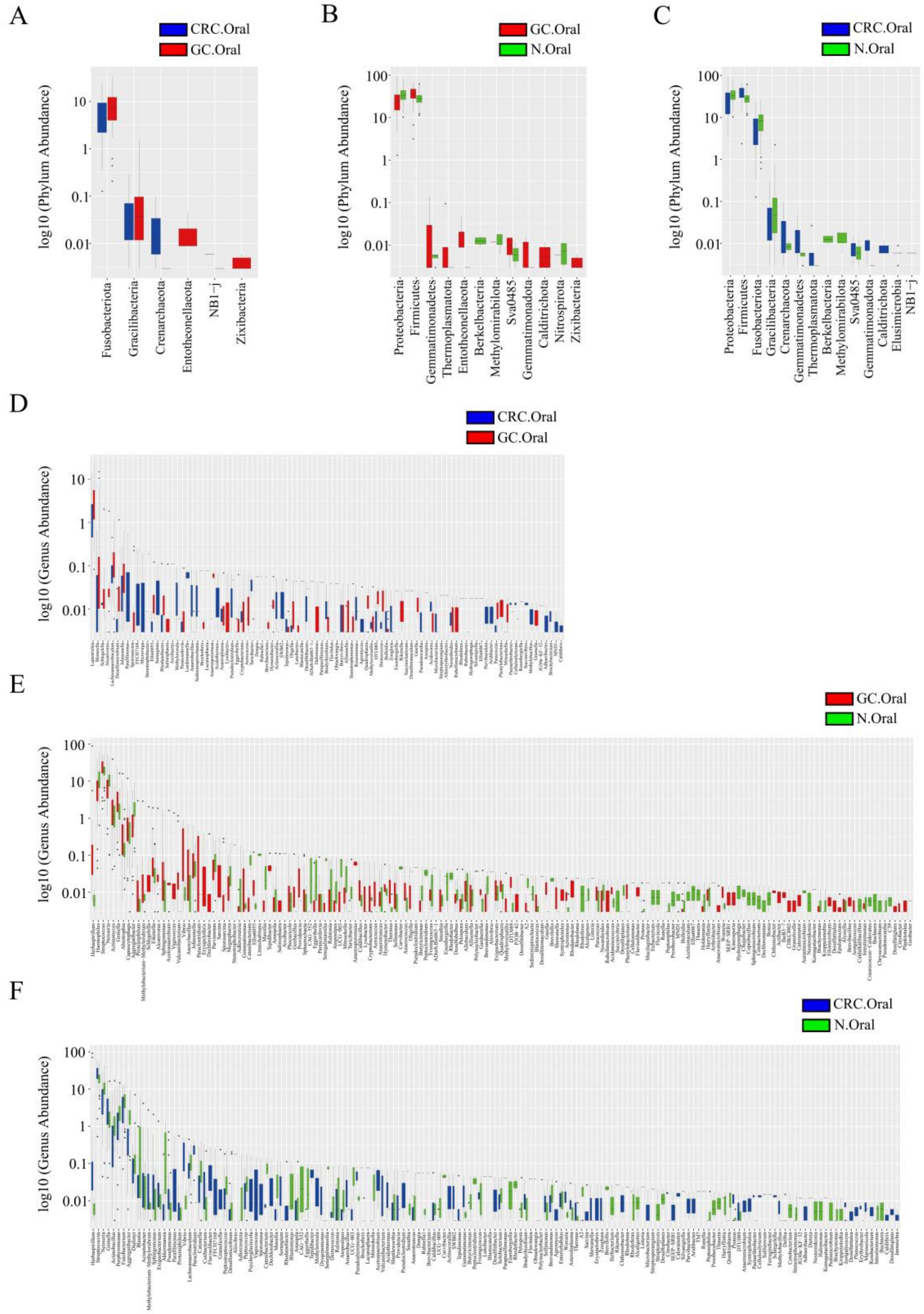

Figure S4. Comparison of oral microbiota diversity among participants in GC (n = 70), CRC (n = 42) and HC (n = 70) (all p <0.05). Microbiota at phylum (A) and genus (D) levels in GC and CRC were represented. The microbial communities in GC and CRC were compared respectively with those in HC at phylum (B, C) and genus (E, F) levels.

Supplementary Figure 5

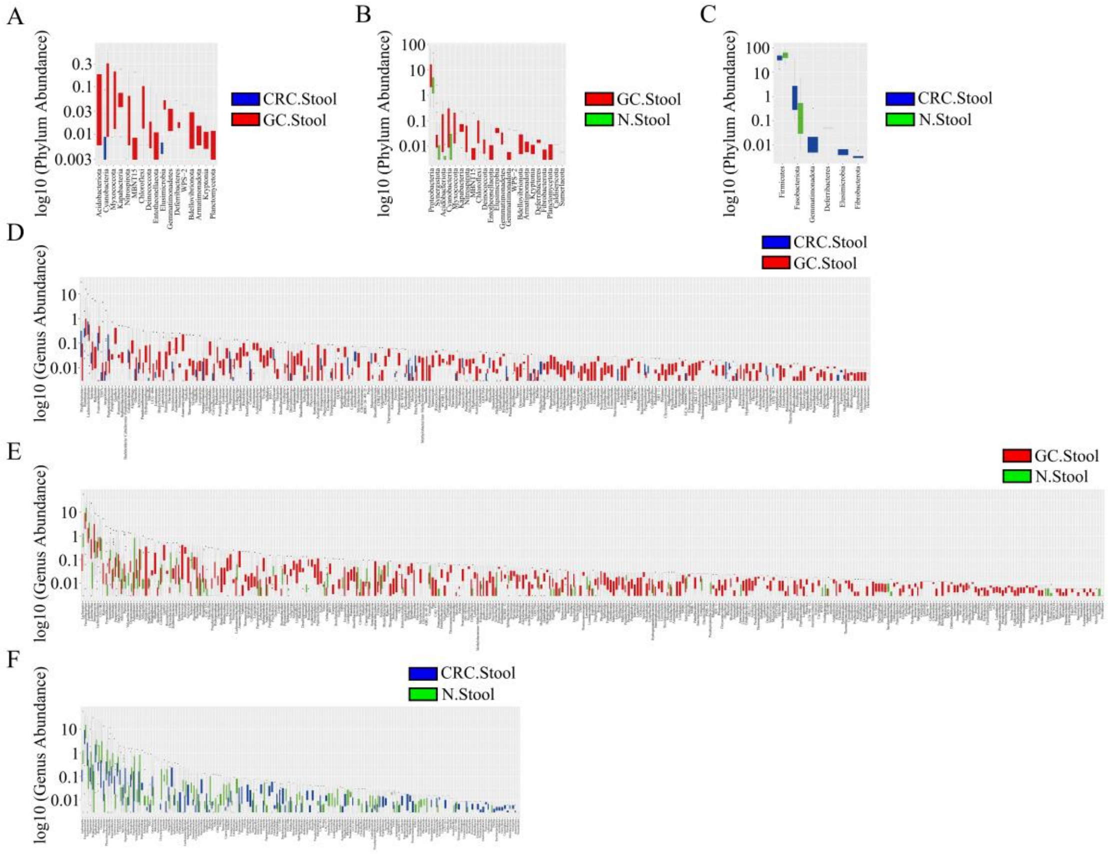

Figure S5. Comparison of fecal microbiota diversity among participants in GC (n = 49), CRC (n = 33) and HC (n = 70) (all p <0.05). Microbiota at phylum (A) and genus (D) levels in GC and CRC were represented. The microbial communities in GC and CRC were compared respectively with those in HC at phylum (B, C) and genus (E, F) levels.

Supplementary Figure 6

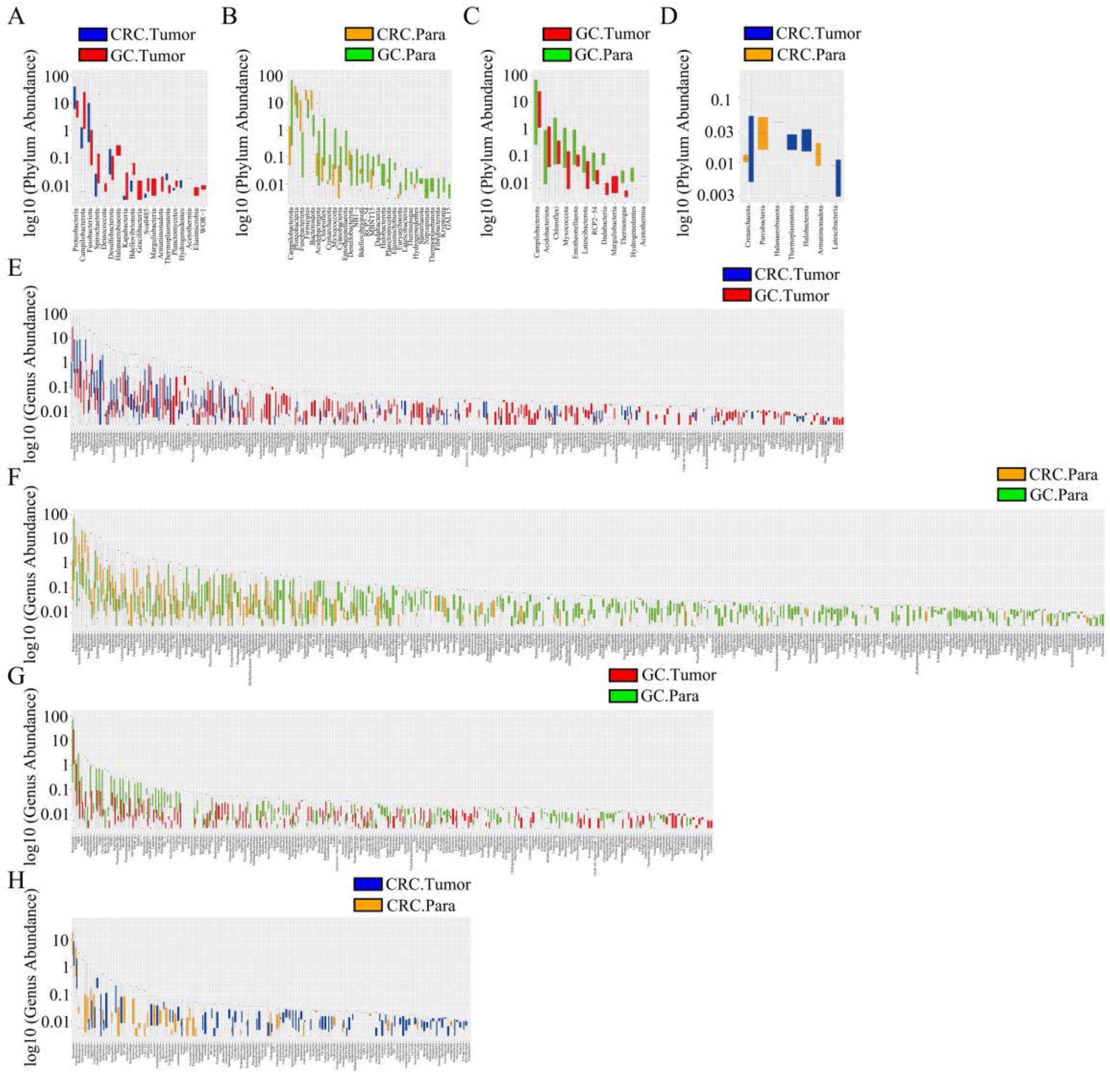

Figure S6. Comparison of fecal microbiota diversity among participants in four groups of GC.Tumor (33), GC.Para (36), CRC.Tumor (24), and CRC.Para (24) (all p <0.05). Microbiota at phylum (A, B) and genus (E, F) levels in GC, CRC and corresponding paracancerous tissue were compared respectively. Microbiota at phylum (C, D) and genus (G, H) levels in GC, CRC tumor and paracancerous tissue were also compared.

Supplementary Figure 7

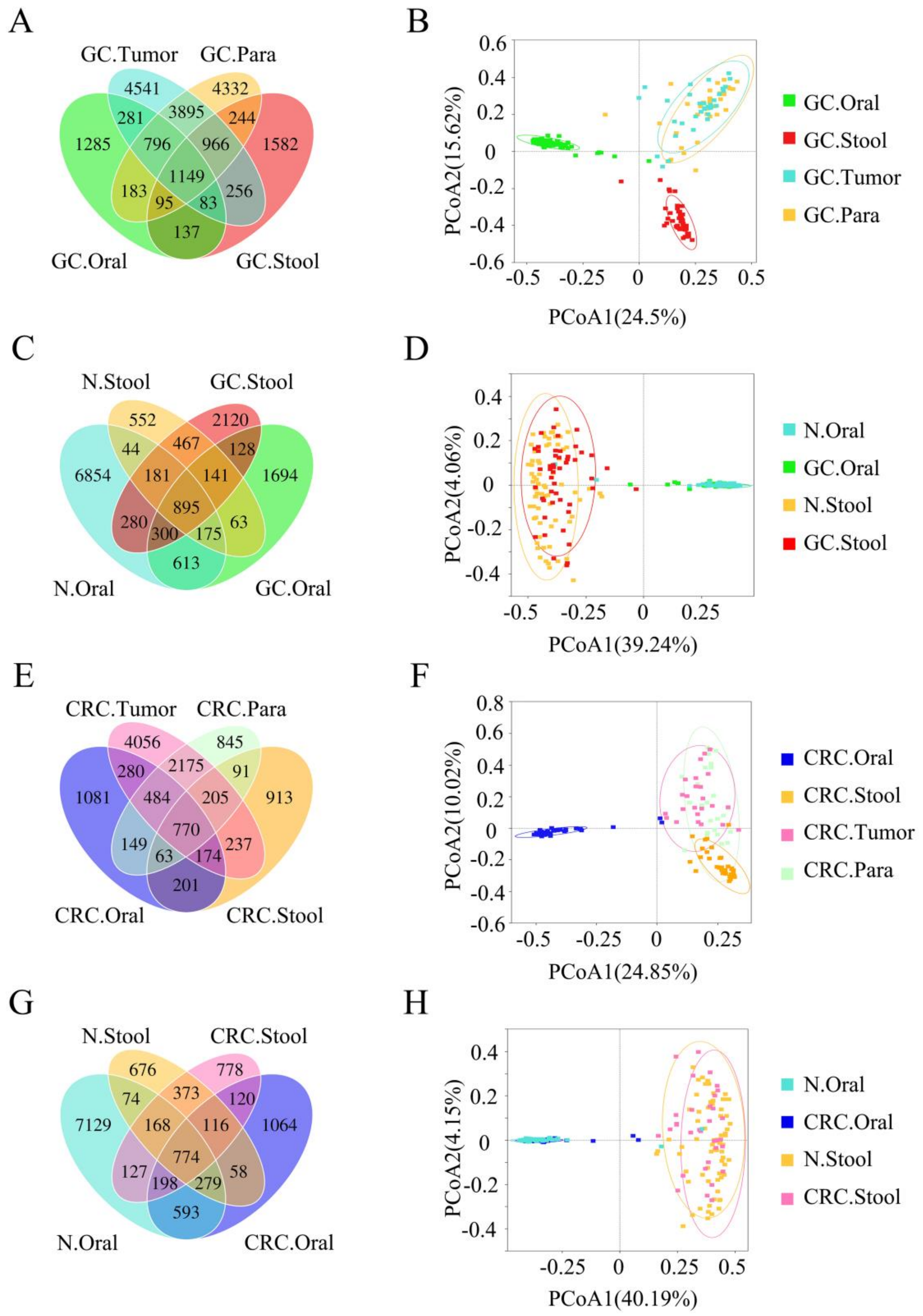

Figure S7: Analysis of microbial diversity among groups. The Venn diagram of oral, fecal, tumor, and paracancerous samples with GC (A) and CRC (E) patients. The Venn diagram oral and fecal samples from GC (C) and CRC (G) patients paired with HC. β-diversity analysis was performed on oral, fecal, tumor, and paracancerous samples from

GC (B) and CRC (D) patients using PCoA on a distance (dissimilarity) matrix of bray-Curtis indexes. β-diversity analysis was performed on oral and fecal samples from GC (F), CRC (H) and HC using PCoA on a distance (dissimilarity) matrix of bray-Curtis indexes.

Supplementary Figure 8

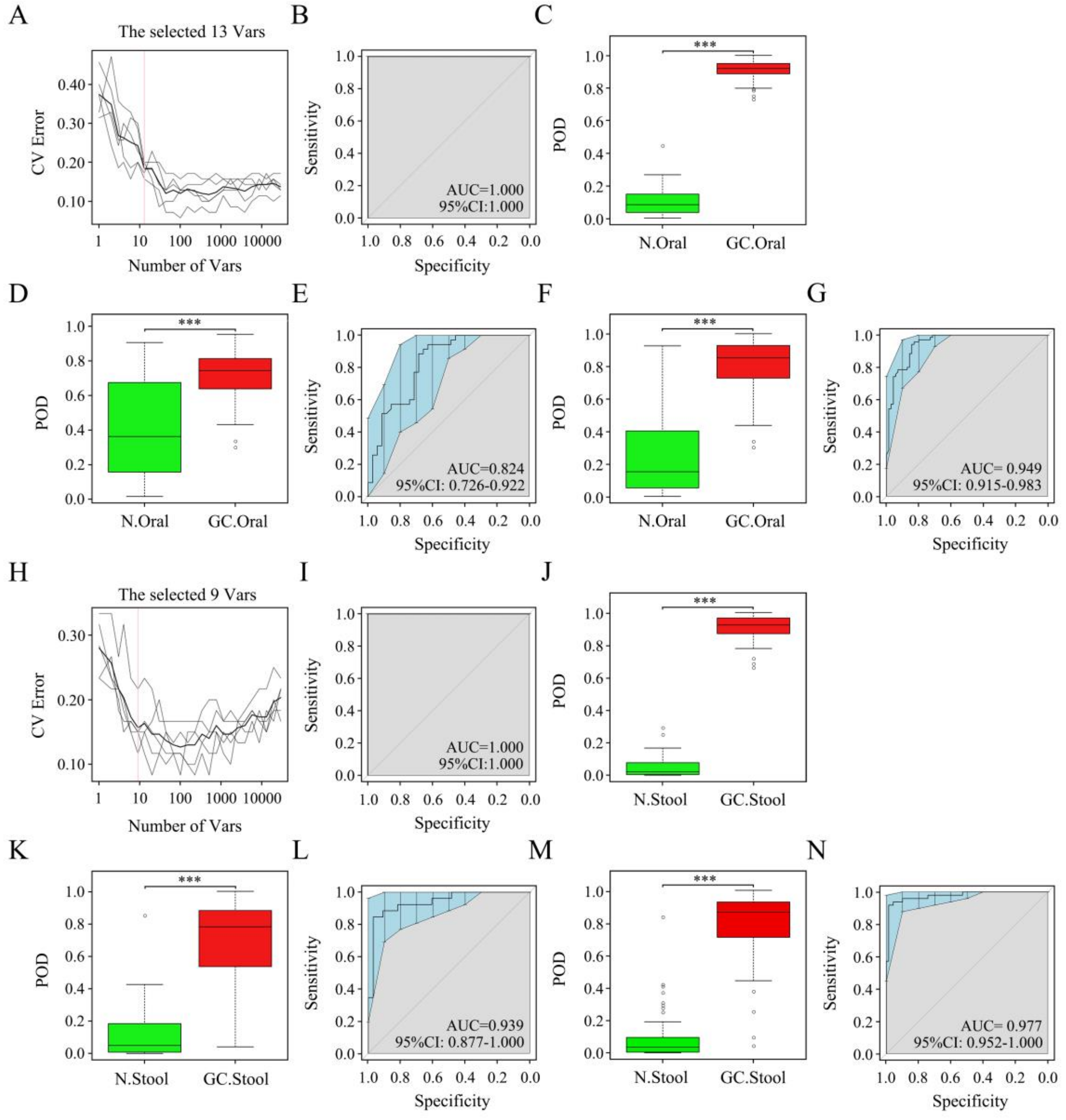

Figure S8: Identification and validation of GC markers based on oral and fecal microbial OTU. (A) In the discovery set of oral samples, five-fold cross-validation was performed on a random forest model between 35 GC samples and 35 controls. The random forest model selected 13 OTUs as the best marker set. (B) The AUC value of the POD index was 100% (95% CI: 100%). (C) The POD value of GC oral samples was significantly higher than that of the control (p <0.001). In the validation phase, (D) the average POD value of 35 GC patients was significantly higher than that of the 35 controls (p <0.001), and (E) the AUC value of the POD index reached 82.40% (95% CI: 72.60%-92.20%). All samples (including 70 GC and 70 controls) were used to

verify the reliability of POD. (F) The average POD value in GC samples was significantly higher than the control (p <0.001), and (G) the AUC value of POD index was 100% (95% CI: 100%). (H) In the discovery set of fecal samples, five-fold cross-validation was performed on a random forest model between 23 GC samples and 34 controls. The random forest model chose 9 OTUs as the best label set. (I) The AUC value of the POD index was 93.9% (95% CI: 87.7%-100%). (J) The POD value of GC oral samples was significantly higher than that of the control (p <0.001). In the validation phase, (K) the average POD value of 26 GC patients was significantly higher than that of 36 controls (p <0.001), and (L) the AUC value of the POD index reached 79.2% (95% CI: 66.9%-91.5%). All samples (including 49 GC and 70 controls) were used to verify the reliability of POD. (M) The average POD value in GC samples was significantly higher than the control (p <0.001), and (N) the AUC value of POD index was 97.7% (95% CI: 95.2%-100%). AUC, area under the curve; CV error, cross-validation error; POD, possibility of disease. *p <0.05, **p <0.01, ***p <0.001

Supplementary Figure 9

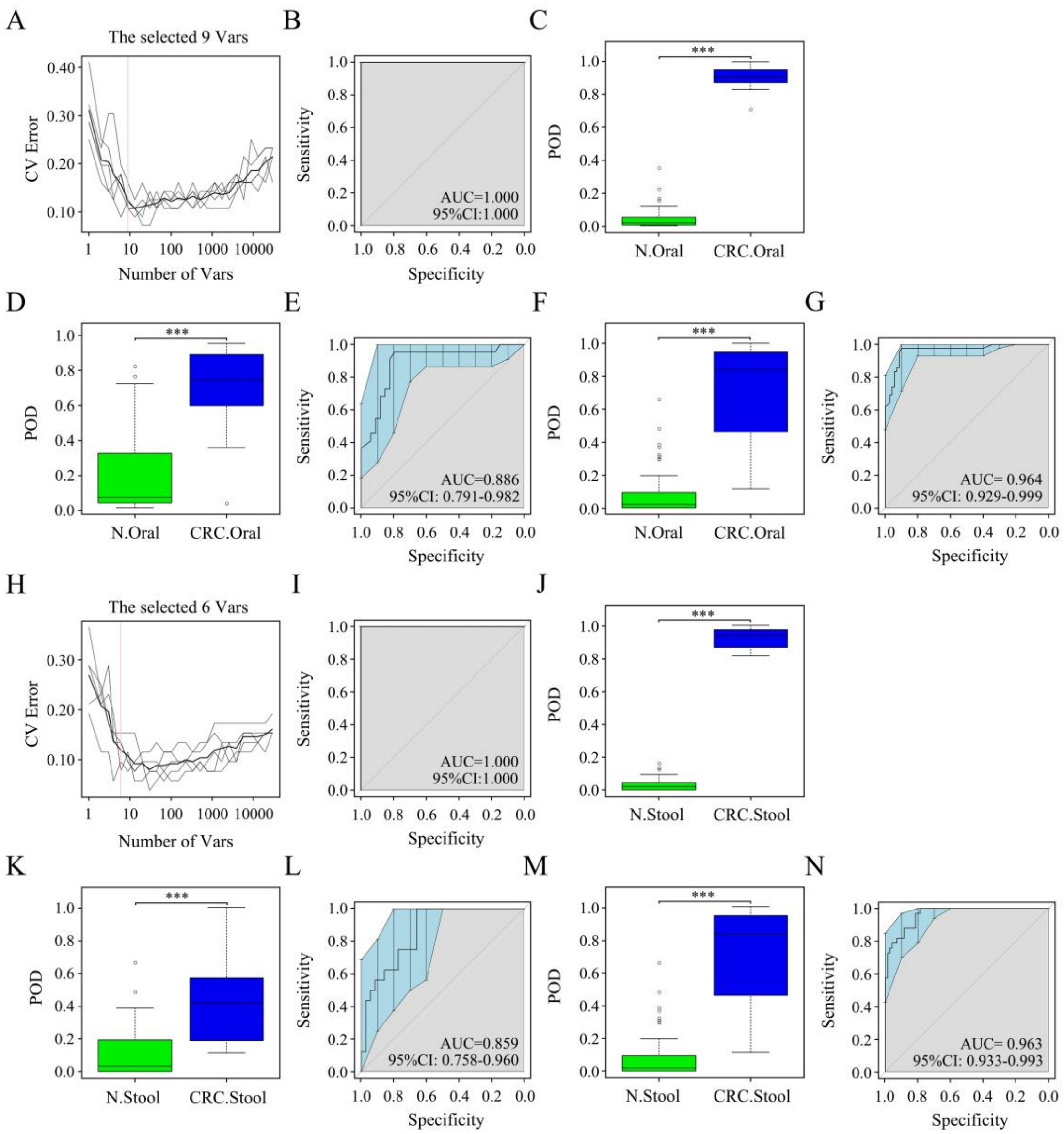

Figure S9: Identification and validation of CRC markers based on oral and fecal microbial OTU. (A) In the discovery set of oral samples, five-fold cross-validation was performed on a random forest model between 20 CRC samples and 37 controls. The random forest model selected 9 OTUs as the best marker set. (B) The AUC value of the POD index was 100% (95% CI: 100%). (C) The POD value of the CRC oral sample was significantly higher than that of the control (p <0.001). In the validation phase, (D) the average POD value of 22 CRC patients was significantly higher than that of 33 controls (p <0.001), and (E) the AUC value of the POD index reached 88.60% (95% CI: 79.10%-98.20%). All samples (including 42 CRC and 70 controls) were used to verify the reliability of POD. (F) The average POD value in the CRC sample was significantly higher than that in the control (p <0.001), and (G) the AUC value of POD index was 96.40% (95% CI: 92.90%-99.90%). (H) In the discovery set of fecal samples, five-fold cross-validation was performed on the random forest model between 17 CRC samples and 35 controls. The random forest model selects 6 OTUs as the best label set.

(I) The AUC value of the POD index is 100% (95% CI: 100%). (J) The POD value of the CRC fecal sample was significantly higher than that of the control (*p* <0.001). In the validation phase, (K) the average POD value of 16 CRC patients was significantly higher than that of 35 controls (*p* <0.001), and (L) the AUC value of POD index reached 85.90% (95% CI: 75.80%-96.00%). All samples (including 33 CRCs and 70 controls) were used to verify the reliability of POD. (M) The average POD value in the CRC sample was significantly higher than that in the control (*p* <0.001), and (N) the AUC value of POD index was 96.30% (95% CI: 93.30%-99.30%). **p* <0.05, ***p* <0.01, ****p* <0.001

Supplementary Figure 10

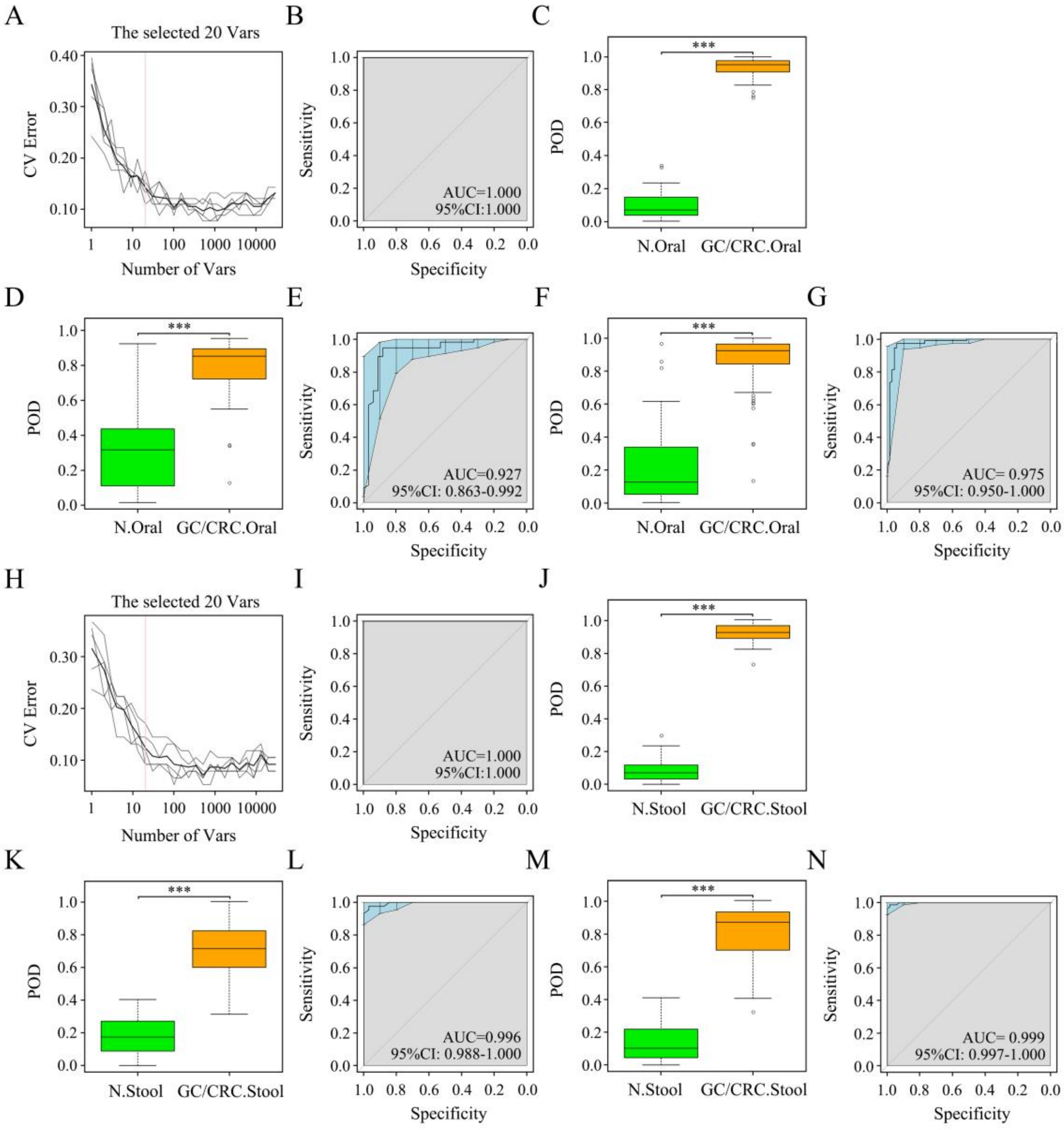

Figure S10: Identification and validation of GI cancers (GC and CRC) markers based on oral and fecal microbial OTU. (A) In the discovery set of oral samples, five-fold cross-validation was performed on a random forest model between 55 GI samples and 36 controls. The random forest model selected 20 OTUs as the best label set. (B) The AUC value of the POD index was 100% (95% CI: 100%). (C) The POD value of GI oral samples was significantly higher than that of the control (*p* <0.001). In the validation phase, (D) the average POD value of 57 GI patients was significantly higher than that of 34 controls (*p* <0.001), and (E) the AUC value of the POD index reached 92.70% (95% CI: 86.30%-99.20%). All samples (including 112 GC/CRC and 70 controls) are used to verify the reliability of POD. (F) The average POD value in GC/CRC samples was significantly higher than the control (*p* <0.001), and (G) the AUC value of POD index was 97.50% (95% CI: 95.00%-100%). (H) In the discovery set of fecal samples, five-fold cross-validation was performed on the random forest model between 38 GI samples and 38 controls. The random forest model selected 20 OTUs as

the best marker set. (I) The AUC value of the POD index was 100% (95% CI: 100%). (J) The POD value of GI fecal samples was significantly higher than that of the control (p <0.001). In the verification phase, (K) the average POD value of 44 GI patients was significantly higher than that of 32 controls (p <0.001), and (L) the AUC value of the POD index reached 99.60% (95% CI: 98.80%-100%). All samples (including 82 GI and 70 controls) were used to verify the reliability of POD. (M) The average POD value in GI samples was significantly higher than the control (p <0.001), and (N) the AUC value of POD index was 99.90% (95% CI: 99.70%-100%). *p <0.05, **p <0.01, ***p <0.001
