# Supplementary material for: Combined Non-Invasive Prediction and New Biomarkers of Oral and Fecal Microbiota in Patients With Gastric and Colorectal Cancer
Source: Front Cell Infect Microbiol. 2022 May 19;12:830684. doi: 10.3389/fcimb.2022.830684 (PMC9161364; doi:10.3389/fcimb.2022.830684)
Supplement: Supplementary file 1 [file DataSheet_1.zip › Supplementary Table 1.pdf]

Table S1. Clinical characteristics and diet information of all enrolled individuals

Sample	Group	Sex	Age (year)	Height (m)	Weight (kg)	BMI (kg/m^2)	Education	Smoking status	Alcohol consumption	Sample collection			
										Oral cavity	stool	tumor	Para cancer
N-4	HC	F	53	1.55	48	19.98	2	1	1	1	1	-	-
N-7	HC	M	63	1.75	68	22.20	3	3	2	1	1	-	-
N-8	HC	M	54	1.7	78	26.99	3	1	2	1	1	-	-
N-11	HC	M	48	1.67	75	26.89	3	1	2	1	1	-	-
N-13	HC	M	63	1.75	73	23.84	3	3	1	1	1	-	-
N-17	HC	M	54	1.76	78	25.18	3	3	2	1	1	-	-
N-21	HC	M	50	1.75	88	28.73	3	1	1	1	1	-	-
N-24	HC	M	63	1.6	64	25.00	2	1	1	1	1	-	-
N-25	HC	M	57	1.65	68	24.98	3	1	2	1	1	-	-
N-26	HC	M	63	1.6	50	19.53	3	3	1	1	1	-	-
N-29	HC	M	68	1.6	68	26.56	3	3	2	1	1	-	-
N-33	HC	M	62	1.65	71	26.08	3	1	1	1	1	-	-
N-34	HC	M	51	1.7	68	23.53	3	3	3	1	1	-	-
N-39	HC	M	73	1.8	98	30.25	1	2	2	1	1	-	-
N-40	HC	M	62	1.72	58	19.61	3	3	2	1	1	-	-
N-41	HC	F	49	1.57	53	21.50	3	1	2	1	1	-	-
N-44	HC	M	69	1.6	58	22.66	1	2	2	1	1	-	-
N-46	HC	F	51	1.58	58	23.23	2	1	1	1	1	-	-
N-47	HC	F	53	1.59	66	26.11	1	1	1	1	1	-	-
N-48	HC	F	50	1.59	50	19.78	3	1	1	1	1	-	-
N-49	HC	M	63	1.8	74	22.84	3	3	2	1	1	-	-
N-50	HC	M	55	1.7	51	17.65	2	1	1	1	1	-	-
N-51	HC	F	47	1.59	59	23.34	3	1	1	1	1	-	-
N-53	HC	M	74	1.72	68	22.99	3	1	1	1	1	-	-
N-54	HC	M	86	1.7	51	17.65	3	1	1	1	1	-	-
N-55	HC	F	80	1.65	72	26.45	3	1	1	1	1	-	-
N-56	HC	F	67	1.55	44	18.31	3	1	1	1	1	-	-
N-59	HC	F	79	1.53	38	16.23	3	1	1	1	1	-	-
N-60	HC	F	73	1.55	57	27.73	3	1	1	1	1	-	-
N-61	HC	F	77	1.7	57	19.72	3	3	3	1	1	-	-
N-62	HC	F	73	1.5	59	26.22	3	1	2	1	1	-	-
N-63	HC	F	72	1.58	63	25.24	3	1	1	1	1	-	-
N-64	HC	F	71	1.62	57	21.72	3	1	2	1	1	-	-
N-65	HC	F	75	1.6	60	23.44	3	1	1	1	1	-	-
N-66	HC	F	71	1.63	68	25.59	3	1	2	1	1	-	-
N-67	HC	M	68	1.79	82	25.59	3	1	2	1	1	-	-
N-69	HC	M	78	1.68	61	21.61	3	2	2	1	1	-	-

N-70	HC	F	73	1.58	64	25.64	3	1	1	1	1	-	-
N-71	HC	F	64	1.38	43	22.58	3	1	2	1	1	-	-
N-74	HC	F	55	1.57	55	22.31	2	1	1	1	1	-	-
N-75	HC	M	63	1.74	75	24.77	3	1	2	1	1	-	-
N-76	HC	M	64	1.64	58	21.56	3	3	2	1	1	-	-
N-80	HC	F	64	1.55	75	31.22	1	1	1	1	1	-	-
N-81	HC	F	55	1.5	60	26.67	1	1	2	1	1	-	-
N-82	HC	M	57	1.65	71	26.08	3	1	1	1	1	-	-
N-83	HC	M	53	1.72	73	24.68	3	1	1	1	1	-	-
N-85	HC	M	51	1.68	76	26.93	3	2	2	1	1	-	-
N-86	HC	F	55	1.66	50	18.14	2	1	1	1	1	-	-
N-89	HC	M	59	1.62	57	21.72	2	3	2	1	1	-	-
N-90	HC	M	53	1.66	66	23.95	3	1	1	1	1	-	-
N-91	HC	F	63	1.55	61	25.39	3	2	2	1	1	-	-
N-93	HC	F	52	1.58	63	25.24	3	1	1	1	1	-	-
N-94	HC	F	55	1.58	53	21.23	3	1	2	1	1	-	-
N-95	HC	M	51	1.65	73	26.81	3	2	3	1	1	-	-
N-97	HC	M	56	1.76	77	24.86	3	1	2	1	1	-	-
N-98	HC	F	60	1.58	60	24.03	3	1	1	1	1	-	-
N-99	HC	F	55	1.6	52	20.31	2	1	1	1	1	-	-
N-101	HC	F	58	1.56	63	25.89	3	1	1	1	1	-	-
N-103	HC	F	54	1.7	68	23.53	3	1	1	1	1	-	-
N-104	HC	M	56	1.72	76	25.69	3	3	3	1	1	-	-
N-105	HC	F	53	1.61	51	19.68	1	1	1	1	1	-	-
N-106	HC	F	60	1.6	64	25.00	1	1	1	1	1	-	-
N-107	HC	M	70	1.69	53	18.56	3	1	1	1	1	-	-
N-109	HC	F	59	1.54	58	24.46	2	1	1	1	1	-	-
N-110	HC	F	56	1.7	61	21.11	2	1	2	1	1	-	-
N-111	HC	M	57	1.75	84	27.43	3	2	2	1	1	-	-
N-112	HC	M	60	1.72	74	25.01	3	3	2	1	1	-	-
N-113	HC	M	51	1.74	78	25.76	3	2	2	1	1	-	-
N-115	HC	M	57	1.7	63	21.80	3	1	1	1	1	-	-
N-116	HC	F	55	1.56	58	23.83	3	1	1	1	1	-	-
G-1	GC	M	68	1.73	66	22.05	3	2	2	1	1	2	2
G-2	GC	F	69	1.51	54	23.68	2	1	1	1	2	1	1
G-3	GC	M	76	1.68	68	24.09	3	1	3	1	1	1	1
G-4	GC	M	73	1.7	54	18.69	3	2	1	1	1	1	1
G-5	GC	M	74	1.62	71	27.05	1	2	3	1	1	1	1
G-6	GC	M	59	1.7	71	24.57	3	2	2	1	1	1	1
G-7	GC	F	51	1.48	46	21.00	1	1	1	1	2	2	2
G-8	GC	M	80	1.68	53	18.78	2	2	2	1	1	1	1
G-9	GC	M	72	1.65	53	19.47	2	2	1	1	1	2	2
G-10	GC	F	57	1.67	44	15.78	3	1	1	1	1	2	2
G-11	GC	M	64	1.61	58	22.38	3	1	2	1	1	1	1

G-13	GC	M	69	1.65	62	22.77	1	2	1	1	2	1	1
G-14	GC	M	67	1.69	86	30.11	3	2	3	1	1	1	1
G-15	GC	M	58	1.75	71	23.18	3	1	2	1	1	2	2
G-16	GC	M	53	1.68	60	21.26	3	1	2	1	2	2	2
G-18	GC	M	66	1.7	50	17.30	3	2	2	1	2	2	2
G-19	GC	M	52	1.65	51	18.73	3	3	3	1	1	2	2
G-20	GC	M	43	1.7	55	19.03	2	2	3	1	1	2	2
G-21	GC	M	56	1.62	66	25.15	3	2	2	1	1	2	2
G-22	GC	F	64	1.65	52	19.10	1	2	1	1	1	1	1
G-23	GC	M	72	1.83	71	21.20	2	1	2	1	1	2	2
G-24	GC	F	71	1.6	46	17.97	2	3	1	1	2	2	2
G-25	GC	F	77	1.47	49	22.68	3	1	1	1	1	1	1
G-26	GC	M	62	1.65	66	24.24	3	2	2	1	1	1	1
G-27	GC	F	69	1.65	81	29.75	2	1	1	1	1	1	1
G-28	GC	F	72	1.55	63	26.22	2	1	1	1	1	2	1
G-29	GC	M	51	1.69	61	21.36	3	1	1	2	1	2	1
G-30	GC	F	78	1.5	53	23.56	1	1	1	2	1	1	1
G-32	GC	F	71	1.52	51	22.07	1	1	1	2	2	1	1
G-33	GC	F	64	1.52	46	19.91	1	1	1	2	1	2	2
G-34	GC	M	71	1.6	47	18.36	3	3	2	1	2	1	1
G-35	GC	F	68	1.69	57	19.96	1	1	1	1	1	2	2
G-38	GC	M	61	1.7	79	27.34	3	2	2	2	1	2	2
G-39	GC	M	68	1.56	50	20.55	1	1	2	1	2	1	1
G-40	GC	M	70	1.7	54	18.69	2	2	2	1	2	1	1
G-41	GC	F	57	1.5	45	20.00	1	1	1	1	2	2	2
G-42	GC	M	52	1.69	53	18.56	3	2	2	1	2	2	2
G-43	GC	F	66	1.5	57	25.33	2	1	1	1	1	2	2
G-44	GC	M	55	1.54	70	29.52	2	1	1	1	1	2	2
G-45	GC	M	68	1.67	74	26.53	3	1	1	1	2	2	2
G-46	GC	M	55	1.65	71	26.08	1	1	2	1	1	2	2
G-47	GC	F	66	1.6	63	24.61	3	1	1	1	2	2	2
G-49	GC	F	58	1.58	43	17.22	1	1	1	1	2	2	2
G-51	GC	M	55	1.7	66	22.84	2	1	1	1	2	1	1
G-53	GC	M	64	1.68	76	26.93	3	1	3	1	2	1	1
G-54	GC	M	58	1.72	73	24.68	2	1	1	1	1	1	1
G-55	GC	F	55	1.58	66	26.44	1	1	1	1	1	1	1
G-56	GC	M	58	1.7	62	21.45	3	2	1	1	2	2	2
G-57	GC	F	70	1.65	56	20.57	1	1	1	1	1	1	1
G-58	GC	M	63	1.65	70	25.71	3	2	2	1	1	2	2
G-59	GC	F	53	1.6	61	23.83	1	1	1	1	2	2	2
G-60	GC	M	79	1.67	76	27.25	3	3	1	1	2	1	1
G-62	GC	M	54	1.67	56	20.08	1	3	3	1	1	1	1
G-63	GC	M	72	1.65	60	22.04	3	1	1	1	2	1	1
G-64	GC	F	70	1.56	66	27.12	2	1	1	1	1	1	1

G-65	GC	M	51	1.69	65	22.76	3	2	3	1	1	2	2
G-66	GC	M	78	1.75	81	26.45	2	2	1	1	1	2	2
G-67	GC	F	64	1.55	56	23.31	1	1	1	1	1	1	1
G-68	GC	M	56	1.65	41	15.06	1	2	1	1	1	1	1
G-70	GC	F	66	1.62	51	19.43	2	1	1	1	1	2	2
G-71	GC	F	73	1.58	52	20.83	3	1	1	1	2	2	2
G-72	GC	M	58	1.75	61	19.92	2	2	1	1	1	2	2
G-73	GC	F	69	1.53	51	21.79	1	1	1	1	1	2	2
G-74	GC	M	48	1.59	60	23.73	2	1	1	1	2	2	2
G-75	GC	M	34	1.7	66	22.84	1	2	2	1	1	2	2
G-76	GC	M	76	1.67	64	22.95	3	2	1	1	1	2	2
G-77	GC	M	56	1.67	56	20.08	3	2	1	1	1	1	1
G-78	GC	M	49	1.70	65	22.49	3	2	1	2	2	1	1
G-79	GC	F	66	1.55	63	26.22	2	1	1	1	1	2	1
G-80	GC	M	73	1.75	71	23.18	2	2	3	1	1	1	1
G-81	GC	M	58	1.7	54	18.69	3	2	3	1	1	2	2
G-83	GC	M	53	1.65	59	21.67	3	3	3	1	2	2	2
G-84	GC	M	68	1.67	76	27.25	3	2	2	1	2	2	2
G-86	GC	M	80	1.6	51	19.92	3	3	3	1	2	2	2
G-87	GC	M	79	1.67	75	26.89	1	2	1	1	1	1	1
G-88	GC	M	57	1.78	71	22.41	2	1	2	1	1	2	2
B-1	CRC	M	31	1.72	90	30.42	3	2	2	1	1	2	2
B-2	CRC	M	63	1.7	72	24.91	3	1	2	1	1	1	1
B-3	CRC	M	52	1.75	64	20.90	3	1	1	1	1	1	1
B-4	CRC	M	72	1.62	71	27.05	3	2	1	1	1	1	1
B-5	CRC	M	88	1.56	54	22.19	2	3	2	1	2	2	2
B-6	CRC	M	73	1.68	65	23.03	1	2	3	1	1	2	2
B-7	CRC	F	30	1.61	50	19.29	3	1	1	1	1	1	1
B-9	CRC	M	73	1.5	55	24.44	1	2	3	1	1	2	2
B-10	CRC	M	43	1.7	76	26.30	3	1	1	1	1	2	2
B-11	CRC	M	66	1.68	70	24.80	3	1	1	2	2	1	1
B-12	CRC	M	35	1.75	73	23.84	3	2	3	1	1	1	1
B-13	CRC	M	62	1.59	56	22.15	1	2	2	1	1	2	2
B-14	CRC	F	81	1.6	59	23.05	2	1	1	1	1	2	2
B-15	CRC	M	48	1.78	85	26.83	3	1	3	1	1	2	2
B-16	CRC	M	35	1.74	82	27.08	3	1	1	1	2	1	1
B-17	CRC	M	65	1.76	83	26.79	3	2	3	1	2	1	1
B-19	CRC	M	57	1.68	60	21.26	2	2	2	1	1	1	1
B-20	CRC	M	71	1.7	70	24.22	2	2	2	1	1	1	1
B-21	CRC	M	75	1.6	60	23.44	3	2	1	1	2	1	1
B-22	CRC	M	77	1.65	55	20.20	3	2	2	2	1	2	2
B-23	CRC	F	36	1.66	54	19.60	3	1	1	2	1	1	1
B-24	CRC	M	57	1.65	72	26.45	3	1	1	1	2	1	1
B-25	CRC	M	71	1.57	45	18.26	2	3	1	2	2	1	1

B-26	CRC	F	65	1.51	55	24.12	3	1	1	2	1	1	1
B-27	CRC	M	55	1.7	55	19.03	3	2	2	2	1	1	1
B-28	CRC	M	63	1.58	65	26.04	2	2	2	1	1	2	2
B-29	CRC	F	79	1.52	53	22.94	2	1	1	2	2	1	1
B-31	CRC	M	76	1.72	73	24.68	3	2	1	2	1	2	2
B-33	CRC	F	77	1.57	62	25.15	1	1	1	2	1	1	1
B-35	CRC	F	64	1.55	63	26.22	1	1	1	1	2	1	1
B-36	CRC	M	55	1.7	70	24.22	3	2	2	1	1	2	2
B-37	CRC	M	55	1.78	82	25.88	2	2	1	2	2	1	1
B-38	CRC	M	61	1.68	68	24.09	3	2	1	1	1	2	2
B-39	CRC	M	56	1.54	44	18.55	3	2	2	1	2	2	2
B-40	CRC	F	62	1.52	69	29.86	1	1	1	1	1	2	2
B-41	CRC	M	56	1.75	78	25.47	2	1	1	1	2	1	1
B-42	CRC	F	55	1.58	51	20.43	1	1	1	1	2	2	2
B-43	CRC	F	50	1.63	65	24.46	1	1	1	1	1	1	1
B-44	CRC	M	51	1.68	71	25.16	3	3	1	1	1	1	1
B-45	CRC	F	56	1.55	42	17.48	1	1	1	1	2	2	2
B-46	CRC	M	67	1.63	60	22.58	1	1	1	1	1	2	2
B-47	CRC	M	32	1.74	59	19.49	3	1	1	1	2	2	2
B-48	CRC	F	57	1.6	59	23.05	1	1	1	2	2	1	1
B-49	CRC	F	62	1.62	55	20.96	1	1	1	1	2	2	2
B-50	CRC	M	24	1.75	55	17.96	3	1	1	1	1	2	2
B-52	CRC	M	66	1.65	60	22.04	1	1	2	1	1	2	2
B-53	CRC	F	67	1.63	72	27.10	2	1	1	1	2	2	2
B-54	CRC	M	57	1.68	70	24.80	3	2	3	1	2	2	2
B-55	CRC	M	73	1.55	65	27.06	3	2	2	1	2	2	2
B-57	CRC	F	49	1.56	56	23.01	3	1	1	1	1	2	2
B-59	CRC	M	67	1.74	82	27.08	3	3	1	1	1	2	2
B-60	CRC	M	47	1.7	75	25.95	2	2	1	1	1	2	2
B-61	CRC	F	73	1.6	55	21.48	1	1	1	1	1	2	2

**Household income (RMB)**	**Family history of cancer**	**Diabetes**	**Tumor location**	**Tumor size (cm)**	**TNM stage**	**Fresh vegetables**	**Fresh fruits**	**High-fat food**	**High-sugar food**	**Pickled food**	**Moldy food**	**FOBT**
1	2	2	-	-	-	1	3	3	3	2	3	-
1	2	2	-	-	-	1	2	2	2	2	3	-
1	2	2	-	-	-	1	3	2	3	2	3	-
1	2	2	-	-	-	1	3	3	2	3	3	-
3	1	2	-	-	-	1	3	2	2	1	3	-
2	1	2	-	-	-	1	2	3	3	3	3	-
1	2	2	-	-	-	1	1	2	3	2	3	-
1	2	2	-	-	-	1	2	3	3	3	3	-

1	2	2	-	-	-	1	2	3	3	3	3	-
1	2	2	-	-	-	1	3	2	3	1	3	-
1	2	2	-	-	-	1	1	3	3	3	3	-
2	2	2	-	-	-	1	2	3	2	2	3	-
3	1	2	-	-	-	1	2	3	3	1	3	-
1	2	2	-	-	-	1	3	1	3	1	3	-
1	2	2	-	-	-	1	1	3	1	1	3	-
2	1	2	-	-	-	1	2	3	3	2	3	-
1	2	2	-	-	-	2	2	2	2	2	3	-
1	1	2	-	-	-	1	2	3	3	2	3	-
1	2	2	-	-	-	1	2	2	1	3	3	-
1	2	2	-	-	-	1	2	3	2	3	3	-
1	2	2	-	-	-	1	2	2	2	2	3	-
1	2	2	-	-	-	2	3	3	3	2	3	-
2	2	2	-	-	-	1	2	3	2	1	3	-
2	1	2	-	-	-	1	1	3	3	3	3	-
2	2	2	-	-	-	1	1	3	2	3	2	-
2	2	2	-	-	-	1	1	3	2	3	3	-
1	2	2	-	-	-	1	1	3	3	1	3	-
1	2	2	-	-	-	1	1	3	2	3	2	-
1	2	2	-	-	-	1	2	3	3	1	3	-
1	2	2	-	-	-	2	3	3	3	1	3	-
1	2	2	-	-	-	1	2	2	3	2	3	-
1	1	2	-	-	-	1	1	3	3	2	3	-
1	1	2	-	-	-	1	1	3	3	1	3	-
1	2	2	-	-	-	1	1	2	3	3	3	-
1	1	2	-	-	-	1	1	3	3	3	3	-
1	2	2	-	-	-	1	1	3	1	3	3	-
1	2	2	-	-	-	1	1	3	3	2	3	-
1	2	2	-	-	-	1	1	3	2	3	3	-
1	2	2	-	-	-	1	1	3	3	1	3	-
2	2	2	-	-	-	1	1	3	3	2	3	-
2	2	2	-	-	-	1	3	2	2	1	3	-
1	2	2	-	-	-	2	2	1	1	1	3	-
1	2	2	-	-	-	1	1	3	3	2	3	-
1	2	2	-	-	-	1	1	3	3	2	3	-
1	2	2	-	-	-	1	1	3	2	2	3	-
2	2	2	-	-	-	1	1	2	3	3	3	-
1	2	2	-	-	-	1	1	2	2	2	3	-
1	2	2	-	-	-	1	1	2	2	2	3	-
2	2	2	-	-	-	3	3	1	3	3	3	-
3	2	2	-	-	-	1	1	3	3	3	3	-
3	2	2	-	-	-	1	1	3	2	1	3	-
2	1	2	-	-	-	1	1	3	3	2	3	-

1	2	2	-	-	-	1	1	2	2	1	3	-
2	2	2	-	-	-	1	3	3	3	2	3	-
2	1	2	-	-	-	1	2	3	2	1	3	-
2	1	2	-	-	-	1	1	3	3	3	3	-
1	2	2	-	-	-	1	1	3	3	3	3	-
2	2	2	-	-	-	1	2	2	3	1	3	-
2	2	2	-	-	-	1	1	3	1	1	3	-
2	2	2	-	-	-	1	2	3	3	1	3	-
1	1	2	-	-	-	1	3	3	2	3	3	-
1	1	2	-	-	-	2	3	3	3	3	3	-
1	2	2	-	-	-	1	1	3	1	3	3	-
2	2	2	-	-	-	1	1	3	3	3	3	-
1	2	2	-	-	-	1	2	2	3	1	3	-
2	2	2	-	-	-	1	2	3	3	3	3	-
1	2	2	-	-	-	1	2	2	3	2	3	-
1	1	2	-	-	-	1	1	3	3	2	3	-
2	2	2	-	-	-	1	1	3	3	3	3	-
2	2	2	-	-	-	1	1	2	3	3	3	-
1	2	2	1	2	1	1	3	2	2	3	3	1
1	2	2	1	6	2	1	3	3	3	2	3	1
1	2	1	1	5	3	1	2	1	3	1	3	-
1	2	2	1	5	2	1	2	3	3	2	3	2
1	2	2	1	5	3	1	1	3	3	1	2	1
1	1	2	1	4	3	1	1	1	2	2	3	-
1	1	2	3	2	1	1	3	3	2	2	3	1
1	2	2	2	4	3	1	3	1	2	3	3	2
1	2	2	1	2	1	2	3	2	3	3	3	1
1	2	2	1	1	1	1	1	3	3	2	3	1
1	2	1	2	8	2	1	2	2	3	3	3	1
1	2	2	1	4	2	1	3	2	2	1	2	-
1	2	2	1	6	2	3	3	2	3	3	3	-
1	2	2	1	4	2	2	1	2	3	2	3	1
1	1	2	3	2	1	1	3	1	3	1	3	-
1	2	2	1	5	4	1	2	3	3	1	3	-
1	2	2	2	3	1	2	1	1	2	3	3	2
1	2	2	3	1	1	1	1	3	3	2	3	-
1	1	2	3	4.5	3	1	2	2	2	3	3	1
1	2	2	3	5.5	3	1	3	2	3	2	3	-
1	2	2	1	5	3	1	1	3	3	1	3	2
1	2	2	3	3	3	2	3	3	2	2	3	1
1	2	2	3	8	3	1	2	1	3	3	2	1
2	2	2	1	3.5	3	1	3	3	3	3	3	1
1	2	2	2	4.3	2	1	2	2	3	1	3	2
2	1	2	2	1.5	2	1	2	3	2	2	3	1

2	1	2	3	3	1	1	2	2	3	2	3	1
1	2	2	3	3.5	1	1	3	2	3	2	3	1
1	1	2	1	8	3	1	2	2	3	2	3	1
2	2	2	3	2	1	1	3	3	3	3	3	1
1	2	2	3	6.5	3	1	3	2	3	1	2	1
1	1	1	3	0.5	1	1	3	3	3	3	3	1
1	1	2	1	3	1	1	3	2	3	1	3	1
1	2	2	1	4	3	1	3	2	2	3	3	1
1	2	2	1	3	2	1	2	3	3	2	3	1
1	2	2	1	6	3	1	3	3	3	2	3	-
1	1	2	3	3	3	1	3	3	3	3	3	-
1	1	2	1	0.2	1	1	3	3	3	1	3	-
1	2	1	1	2.5	2	1	2	3	3	3	3	2
1	1	1	1	3	2	1	3	2	3	3	3	2
3	2	1	1	5	3	1	1	1	3	2	3	1
1	1	2	2	2.5	1	1	3	2	1	1	3	1
1	2	2	2	4	3	1	2	2	1	3	3	1
1	1	2	1	5	2	1	2	2	2	2	3	1
1	1	2	1	6	3	1	3	1	3	2	3	1
1	2	2	3	8.5	3	1	3	3	3	2	2	1
1	2	2	3	3.5	2	1	3	3	3	1	3	1
1	2	2	1	4	3	1	3	3	3	2	3	1
1	2	2	1	5	3	1	3	3	3	3	3	-
1	2	2	2	5	4	1	3	3	2	3	3	1
1	2	2	2	4	3	1	2	3	3	2	3	1
2	2	2	1	4	3	1	3	2	3	1	3	1
1	2	2	3	1	1	1	3	3	2	1	3	1
1	2	2	1	6.5	2	1	2	3	3	3	3	-
1	2	2	3	2	1	1	2	3	3	3	3	1
2	2	2	2	5	4	1	3	3	3	3	2	2
1	2	2	1	5	4	1	3	3	3	3	3	1
1	2	2	2	2	2	1	2	3	3	3	3	1
1	2	1	1	6.5	2	1	2	3	3	2	3	-
1	2	2	1	5	4	1	2	3	3	2	3	-
1	2	2	3	2.5	3	1	2	2	3	2	3	1
1	2	2	1	5	4	1	2	3	2	1	3	2
1	2	2	2	5	2	1	3	3	3	3	3	1
1	2	2	2	1.5	1	1	3	2	3	1	2	1
1	2	2	1	4	3	1	3	3	3	3	3	-
1	2	2	1	1.5	3	1	1	3	3	3	3	1
2	2	2	1	4.5	3	1	3	3	3	3	3	2
1	2	2	3	3	4	1	2	3	3	3	3	1
1	1	2	2	1.1	1	1	3	1	2	1	3	-
1	1	2	2	6.5	3	1	3	1	3	3	3	1

1	1	2	1	3	2	1	3	3	3	2	3	-
1	2	2	3	4	3	1	2	3	3	3	3	1
1	2	2	1	4	2	1	2	2	3	3	3	2
1	2	2	2	0.9	1	1	3	2	3	1	3	2
1	2	2	2	5	3	1	3	2	1	3	3	1
1	2	1	2	2.3	4	1	3	3	3	3	3	1
1	1	2	6	8.3	3	2	3	1	3	2	3	2
2	2	2	6	3	2	2	2	2	2	2	3	2
1	2	2	5	5	3	1	1	2	2	3	3	2
3	2	2	6	4	3	1	2	3	3	3	3	2
1	2	2	6	6.3	3	3	3	2	3	3	2	2
1	2	2	6	3.3	3	1	3	2	3	3	3	1
1	2	2	4	8	2	1	2	2	2	2	3	2
1	2	2	6	4.3	3	1	3	2	3	3	3	2
1	2	2	5	4.5	1	1	2	2	2	3	3	1
1	2	2	4	4	3	1	3	2	3	1	3	-
1	2	2	6	2	2	2	3	2	2	3	3	1
1	2	2	6	1	1	1	3	2	3	3	3	1
1	2	2	6	4	3	1	3	3	3	1	3	2
2	1	1	5	1.2	1	1	3	3	3	1	3	1
1	1	2	6	3	3	1	2	1	3	2	3	1
2	2	1	5	3	3	1	2	1	3	2	3	2
1	2	2	4	4	4	1	3	2	3	1	3	2
1	2	2	5	3	3	2	3	2	3	3	3	2
1	2	2	6	3	1	1	3	3	2	3	2	2
1	2	2	6	1	4	1	3	2	3	3	3	1
1	2	2	6	3	1	1	2	3	3	3	3	2
2	2	1	5	4	3	2	2	2	3	3	3	1
1	2	2	6	6.5	2	1	3	3	3	3	3	2
2	2	2	6	2	1	1	1	3	3	3	3	2
1	2	2	6	5.5	3	1	2	2	3	2	3	1
1	2	2	6	6.5	1	1	3	2	3	3	3	1
1	2	2	6	4	2	1	2	2	2	2	3	2
1	2	2	6	6	3	1	3	3	3	3	3	2
1	2	1	4	3	3	1	3	3	3	2	3	1
1	2	2	6	2	1	1	2	1	2	2	3	2
2	1	2	6	2.5	1	1	1	1	2	1	3	1
1	1	2	6	1.1	1	1	2	3	3	2	3	2
1	1	2	6	4.4	3	1	3	2	3	3	3	2
1	1	2	6	6.9	3	1	3	1	3	1	3	2
1	1	1	4	5	3	1	3	3	3	1	3	2
1	1	2	5	4.5	3	1	1	2	3	2	3	2
1	1	2	6	8	2	1	3	3	3	3	2	2
1	2	2	5	3.5	1	1	3	3	3	1	3	2

1	1	2	4	6.3	2	1	1	2	3	1	3	-
1	2	2	6	3.8	3	1	2	3	3	3	3	2
2	2	2	6	4	3	1	3	3	3	3	3	1
1	2	2	5	5	4	1	3	3	3	3	3	1
1	1	2	6	7.3	1	1	3	3	3	3	3	2
1	2	2	6	1	4	1	2	3	3	3	3	2
1	2	2	6	5	3	1	3	3	3	3	3	2
1	2	2	6	7.5	2	1	3	2	3	1	2	1
1	1	2	5	5.5	2	1	1	3	3	1	3	2
1	2	2	6	3	2	1	2	2	3	3	3	2
1	1	2	6	1.5	3	1	3	2	3	3	3	2
1	2	2	6	4	3	1	3	2	2	3	3	2
1	1	2	5	4	4	1	2	2	3	3	3	1
1	1	2	5	3	1	1	1	3	3	3	2	2
1	2	2	4	4	3	1	3	2	1	1	2	1

Abbreviations: HC (Healthy controls), GC (Gastric cancer), CRC (Colorectal cancer), M (Male), F (Female). Education (1 No education, 2 Primary education, 3 Secondary education or more), Smoking status (1 Never smoker, 2 Former smoker, 3 Current smoker), Alcohol consumption (1 Never drink, 2 <1 standard drink per day, 3 ≥1standard drink per day), Sample collection (Oral cavity (1 Yes, 2 No), stool (1 Yes, 2 No), tumor (1 Yes, 2 No), Paracancer (1 Yes, 2 No)), Household income, RMB (1 ≤5000, 2 5000-10000, 3 ≥10000), Family history of cancer (1 Yes, 2 No), Diabetes (1 Yes, 2 No), Tumor location (1 Upper third of stomach, 2 Middle third of stomach, 3 Lower third of stomach, 4 Proximal colon, 5 Distal colon, 6 Rectum), Fresh vegetables (1 ≥5 days/per week, 2 2-4 days/per week, 3 ≤1 day/per week), Fresh fruits (1 ≥5 days/per week, 2 2-4 days/per week, 3 ≤1 day/per week), High-fat food (1 ≥5 days/per week, 2 2-4 days/per week, 3 ≤1 day/per week), High-sugar food (1 ≥5 days/per week, 2 2-4 days/per week, 3 ≤1 day/per week), Pickled food (1 ≥5 days/per week, 2 2-4 days/per week, 3 ≤1 day/per week), Moldy food (1 ≥5 days/per week, 2 2-4 days/per week, 3 ≤1 day/per week), FOBT (1 Yes, 2 No).

Fresh vegetables: carrots, spinach, broccoli, spring greens, kale, brussels, sprouts, cabbage, peas, green beans, broad beans, runner, beans, courgettes, cauliflower parsnips, turnips, leeks, onions, garlic, mushrooms, sweet peppers, green salad, lettuce cucumber, celery tomatoes, sweet-corn, beetroot, coleslaw, baked beans, dried lentils, beans, peas

Fresh fruits: apples, pears, oranges, satsumas, mandarins, grapefruit, bananas, grapes, melon, peaches, plums, apricots, strawberries, raspberries, kiwi fruit, blueberries, guava, red dates, persimmon, litchi, fig, pomegranate

High-fat food: pot-stewed meat, barbecue, salted duck egg, beef jerky, pork fat, cheese, animal oil, condensed milk, cream, full-fat yoghurt or greek style yoghurt (125g carton), wholemeal pasta, breaded chicken, chicken nuggets, chicken burger, Bacon/Ham

High-sugar food: chocolate coated sweet biscuits e.g., digestive, buns, pastries e.g., croissants, doughnuts, fruit pies, tarts, crumbles, sponge puddings, ice cream, frozen desserts, chocolates, sweets, toffees, mints, sugar added to tea coffee, packet snacks, honey, syrup (teaspoon)

Pickled food: sausage, salted duck egg, salted pork, cured fish, laba garlic, pickles strawberry, pickled cucumber

Moldy food: moldy fruit, germination potatoes, mildew nuts, red sugarcane
