# Supplementary material for: Combined Non-Invasive Prediction and New Biomarkers of Oral and Fecal Microbiota in Patients With Gastric and Colorectal Cancer
Source: Front Cell Infect Microbiol. 2022 May 19;12:830684. doi: 10.3389/fcimb.2022.830684 (PMC9161364; doi:10.3389/fcimb.2022.830684)
Supplement: Supplementary file 1 [file DataSheet_1.zip › Supplementary Table 2.pdf]

Table S2. Microbial α diversity index of all samples

sample	group	observed_species	shannon	simpson	Chao1	ACE	goods_coverage	PD_whole_tree
N.O.1	N.Oral	451	3.139	0.376	933	1018	0.933	173
N.O.1	N.Oral	451	3.139	0.763	933	1018	0.993	173
N.O.2	N.Oral	647	4.491	0.896	1160	1241	0.99	259
N.O.3	N.Oral	504	4.008	0.881	607	679	0.995	222
N.O.4	N.Oral	331	3.764	0.813	477	452	0.997	100
N.O.5	N.Oral	854	6.461	0.968	1174	1219	0.991	196
N.O.6	N.Oral	567	4.111	0.851	639	689	0.995	215
N.O.7	N.Oral	245	3.156	0.737	307	330	0.998	69
N.O.8	N.Oral	333	4.482	0.903	461	497	0.997	97
N.O.9	N.Oral	1251	6.465	0.955	1728	1707	0.987	585
N.O.10	N.Oral	465	2.928	0.721	834	1018	0.993	141
N.O.11	N.Oral	1004	6.544	0.975	1411	1564	0.988	281
N.O.12	N.Oral	514	4.733	0.907	1029	1034	0.993	157
N.O.13	N.Oral	592	5.479	0.947	1033	1196	0.992	203
N.O.14	N.Oral	769	4.195	0.834	1338	1447	0.988	247
N.O.15	N.Oral	532	5.133	0.93	977	1119	0.992	178
N.O.16	N.Oral	325	3.52	0.771	479	474	0.997	94
N.O.17	N.Oral	449	5.133	0.927	869	865	0.994	128
N.O.18	N.Oral	522	4.32	0.881	842	933	0.993	171
N.O.19	N.Oral	723	5.391	0.942	864	999	0.993	269
N.O.20	N.Oral	722	4.376	0.854	910	993	0.992	235
N.O.21	N.Oral	779	3.959	0.753	938	1061	0.992	243
N.O.22	N.Oral	1394	5.773	0.918	1634	1770	0.987	483
N.O.23	N.Oral	815	5.939	0.958	945	1011	0.993	310
N.O.24	N.Oral	344	4.971	0.917	416	419	0.998	61
N.O.25	N.Oral	483	5.339	0.924	647	618	0.996	81
N.O.26	N.Oral	540	5.305	0.909	640	643	0.996	82
N.O.27	N.Oral	519	5.64	0.933	631	628	0.996	127
N.O.28	N.Oral	418	4.82	0.922	492	477	0.997	76
N.O.29	N.Oral	482	4.211	0.874	589	620	0.996	161
N.O.30	N.Oral	479	5.014	0.887	600	595	0.996	92
N.O.31	N.Oral	471	5.045	0.916	598	596	0.996	86
N.O.32	N.Oral	513	6.005	0.953	618	617	0.997	79
N.O.33	N.Oral	513	5.583	0.936	614	622	0.997	95
N.O.34	N.Oral	353	4.47	0.82	430	425	0.998	75
N.O.35	N.Oral	386	5.302	0.915	475	457	0.998	99
N.O.36	N.Oral	391	5.842	0.96	458	437	0.998	77
N.O.37	N.Oral	366	5.286	0.926	410	417	0.998	66
N.O.38	N.Oral	499	5.606	0.927	602	591	0.997	87
N.O.39	N.Oral	561	5.693	0.926	715	696	0.996	92

N.O.40	N.Oral	472	5.608	0.955	560	582	0.997	190
N.O.41	N.Oral	508	5.449	0.941	640	641	0.996	166
N.O.42	N.Oral	476	5.36	0.893	570	570	0.997	89
N.O.43	N.Oral	472	5.317	0.923	549	556	0.997	86
N.O.44	N.Oral	491	6.122	0.96	584	564	0.997	75
N.O.45	N.Oral	297	4.942	0.94	343	359	0.998	68
N.O.46	N.Oral	386	5.681	0.943	435	434	0.998	143
N.O.47	N.Oral	304	5.074	0.926	345	358	0.998	61
N.O.48	N.Oral	318	5.159	0.933	389	381	0.998	81
N.O.49	N.Oral	372	5.802	0.961	426	431	0.998	91
N.O.50	N.Oral	344	5.353	0.949	397	397	0.998	71
N.O.51	N.Oral	330	5.063	0.914	364	375	0.998	75
N.O.52	N.Oral	326	5.104	0.933	385	386	0.998	85
N.O.53	N.Oral	344	5.25	0.933	397	415	0.998	82
N.O.54	N.Oral	351	5.491	0.948	428	418	0.998	94
N.O.55	N.Oral	331	5.185	0.931	365	375	0.998	91
N.O.56	N.Oral	277	4.725	0.908	328	333	0.998	69
N.O.57	N.Oral	280	3.73	0.799	339	335	0.998	68
N.O.58	N.Oral	354	5.076	0.917	419	429	0.998	129
N.O.59	N.Oral	324	5.356	0.947	426	426	0.997	70
N.O.60	N.Oral	314	4.573	0.89	372	391	0.998	83
N.O.61	N.Oral	355	5.655	0.955	413	424	0.998	105
N.O.62	N.Oral	307	5.1	0.941	378	362	0.998	81
N.O.63	N.Oral	297	4.953	0.912	362	351	0.998	63
N.O.64	N.Oral	340	4.891	0.889	407	393	0.998	113
N.O.65	N.Oral	342	5.049	0.922	433	420	0.998	90
N.O.66	N.Oral	304	4.232	0.845	360	365	0.998	80
N.O.67	N.Oral	295	4.895	0.92	366	357	0.998	71
N.O.68	N.Oral	285	4.357	0.9	324	336	0.998	77
N.O.69	N.Oral	341	4.782	0.908	418	404	0.998	101
N.O.70	N.Oral	298	4.518	0.87	359	358	0.998	88
G.O.1	GC.Oral	538	5.737	0.932	635	639	0.997	110
G.O.2	GC.Oral	502	5.816	0.943	596	600	0.997	95
G.O.3	GC.Oral	277	4.164	0.86	335	335	0.998	64
G.O.4	GC.Oral	442	4.81	0.911	535	547	0.997	92
G.O.5	GC.Oral	474	5.355	0.902	569	571	0.997	110
G.O.6	GC.Oral	475	5.168	0.917	559	574	0.997	115
G.O.7	GC.Oral	263	2.929	0.777	332	346	0.998	59
G.O.8	GC.Oral	517	5.805	0.946	640	627	0.996	92
G.O.9	GC.Oral	258	3.421	0.722	315	310	0.998	73
G.O.10	GC.Oral	352	4.136	0.836	400	417	0.998	83
G.O.11	GC.Oral	249	3.006	0.768	321	302	0.998	62
G.O.12	GC.Oral	493	5.854	0.961	615	629	0.996	210
G.O.13	GC.Oral	475	5.906	0.959	586	607	0.996	211

G.O.14	GC.Oral	286	4.512	0.862	353	344	0.998	67
G.O.15	GC.Oral	380	4.457	0.887	442	451	0.997	160
G.O.16	GC.Oral	445	3.577	0.701	544	583	0.996	210
G.O.17	GC.Oral	500	4.254	0.872	592	616	0.996	315
G.O.18	GC.Oral	325	4.645	0.909	382	405	0.998	190
G.O.19	GC.Oral	307	3.781	0.799	358	395	0.998	74
G.O.20	GC.Oral	360	3.979	0.812	453	464	0.997	270
G.O.21	GC.Oral	718	5.281	0.894	1009	1074	0.992	859
G.O.22	GC.Oral	414	3.503	0.702	529	567	0.996	208
G.O.23	GC.Oral	329	4.536	0.899	395	401	0.998	179
G.O.24	GC.Oral	406	3.785	0.854	558	564	0.996	171
G.O.25	GC.Oral	409	4.589	0.888	549	546	0.996	159
G.O.26	GC.Oral	395	4.602	0.884	518	543	0.996	179
G.O.27	GC.Oral	320	5.544	0.957	367	385	0.998	93
G.O.28	GC.Oral	287	4.629	0.899	322	319	0.999	67
G.O.29	GC.Oral	331	4.245	0.883	433	424	0.997	229
G.O.30	GC.Oral	425	4.613	0.851	565	563	0.996	72
G.O.31	GC.Oral	501	5.645	0.95	660	638	0.996	271
G.O.32	GC.Oral	331	4.763	0.906	418	419	0.997	77
G.O.33	GC.Oral	309	4.033	0.853	374	385	0.998	107
G.O.34	GC.Oral	324	4.607	0.903	395	411	0.998	116
G.O.35	GC.Oral	371	5.165	0.927	464	460	0.997	187
G.O.36	GC.Oral	338	4.306	0.838	426	415	0.998	127
G.O.37	GC.Oral	341	5.686	0.955	389	400	0.998	110
G.O.38	GC.Oral	325	4.363	0.891	368	371	0.998	189
G.O.39	GC.Oral	271	2.119	0.609	345	377	0.997	113
G.O.40	GC.Oral	310	4.8	0.916	391	391	0.998	89
G.O.41	GC.Oral	324	4.697	0.854	380	374	0.998	83
G.O.42	GC.Oral	364	3.869	0.794	429	440	0.997	122
G.O.43	GC.Oral	280	5.118	0.933	337	329	0.998	58
G.O.44	GC.Oral	275	3.911	0.873	319	317	0.998	72
G.O.45	GC.Oral	285	4.941	0.92	346	330	0.998	66
G.O.46	GC.Oral	271	3.48	0.75	338	329	0.998	77
G.O.47	GC.Oral	264	3.425	0.786	313	316	0.998	74
G.O.48	GC.Oral	287	4.302	0.844	334	341	0.998	63
G.O.49	GC.Oral	449	6.098	0.964	531	521	0.997	104
G.O.50	GC.Oral	468	5.045	0.91	585	587	0.996	150
G.O.51	GC.Oral	428	5.344	0.944	497	510	0.997	100
G.O.52	GC.Oral	390	4.149	0.82	468	474	0.997	96
G.O.53	GC.Oral	408	5.421	0.929	507	518	0.997	167
G.O.54	GC.Oral	483	5.364	0.907	616	643	0.996	189
G.O.55	GC.Oral	239	3.847	0.816	269	280	0.999	79
G.O.56	GC.Oral	367	4.731	0.889	416	424	0.998	183
G.O.57	GC.Oral	438	3.906	0.765	540	550	0.997	146

G.O.58	GC.Oral	437	4.204	0.835	562	557	0.996	160
G.O.59	GC.Oral	484	5.894	0.953	589	610	0.997	159
G.O.60	GC.Oral	434	4.437	0.844	547	557	0.997	144
G.O.61	GC.Oral	445	4.844	0.881	548	543	0.997	102
G.O.62	GC.Oral	389	5.877	0.963	457	475	0.997	79
G.O.63	GC.Oral	361	4.093	0.892	478	471	0.997	70
G.O.64	GC.Oral	331	5.492	0.938	386	383	0.998	66
G.O.65	GC.Oral	320	4.591	0.887	353	366	0.998	82
G.O.66	GC.Oral	327	5.307	0.926	394	401	0.998	71
G.O.67	GC.Oral	323	4.461	0.873	353	364	0.998	82
G.O.68	GC.Oral	348	3.943	0.806	412	432	0.997	69
G.O.69	GC.Oral	387	4.721	0.896	473	496	0.997	100
G.O.70	GC.Oral	233	1.027	0.212	328	340	0.997	98
B.O.1	CRC.Oral	480	4.7	0.842	564	561	0.997	86
B.O.2	CRC.Oral	466	4.773	0.862	574	584	0.996	94
B.O.3	CRC.Oral	363	4.202	0.863	435	443	0.997	64
B.O.4	CRC.Oral	409	4.223	0.789	541	512	0.997	93
B.O.5	CRC.Oral	306	4.166	0.879	419	410	0.997	61
B.O.6	CRC.Oral	399	4.697	0.869	475	488	0.997	85
B.O.7	CRC.Oral	299	3.444	0.795	406	432	0.997	93
B.O.8	CRC.Oral	371	4.979	0.932	456	483	0.997	120
B.O.9	CRC.Oral	385	5.489	0.939	426	436	0.998	90
B.O.10	CRC.Oral	440	4.409	0.877	573	582	0.996	230
B.O.11	CRC.Oral	423	3.836	0.759	535	559	0.996	287
B.O.12	CRC.Oral	348	3.492	0.803	452	460	0.997	213
B.O.13	CRC.Oral	341	4.018	0.876	481	449	0.997	79
B.O.14	CRC.Oral	362	3.671	0.818	512	523	0.996	111
B.O.15	CRC.Oral	275	0.834	0.166	366	401	0.997	96
B.O.16	CRC.Oral	267	3.278	0.787	346	360	0.998	218
B.O.17	CRC.Oral	437	4.785	0.895	568	605	0.996	96
B.O.18	CRC.Oral	297	5.161	0.931	339	339	0.998	97
B.O.19	CRC.Oral	370	4.564	0.888	434	435	0.998	94
B.O.20	CRC.Oral	337	3.853	0.848	444	468	0.997	125
B.O.21	CRC.Oral	441	4.416	0.855	556	577	0.996	207
B.O.22	CRC.Oral	423	4.587	0.893	534	544	0.997	183
B.O.23	CRC.Oral	460	5.506	0.932	589	601	0.996	256
B.O.24	CRC.Oral	488	4.612	0.845	610	645	0.996	170
B.O.25	CRC.Oral	361	4.033	0.881	519	510	0.996	64
B.O.26	CRC.Oral	412	4.855	0.898	554	580	0.996	305
B.O.27	CRC.Oral	342	2.698	0.618	621	541	0.996	149
B.O.28	CRC.Oral	311	3.964	0.857	467	413	0.997	113
B.O.29	CRC.Oral	305	3.021	0.708	409	405	0.997	98
B.O.30	CRC.Oral	424	4.526	0.883	503	526	0.997	182
B.O.31	CRC.Oral	325	3.554	0.812	426	427	0.997	105

B.O.32	CRC.Oral	325	3.916	0.83	468	438	0.997	105
B.O.33	CRC.Oral	332	2.323	0.484	451	459	0.997	150
B.O.34	CRC.Oral	290	4.393	0.877	350	346	0.998	87
B.O.35	CRC.Oral	344	4.649	0.906	414	417	0.998	99
B.O.36	CRC.Oral	326	3.861	0.848	439	460	0.997	65
B.O.37	CRC.Oral	368	4.798	0.922	416	430	0.998	81
B.O.38	CRC.Oral	315	3.931	0.845	388	386	0.998	87
B.O.39	CRC.Oral	351	5.321	0.927	421	425	0.998	76
B.O.40	CRC.Oral	338	4.833	0.899	416	425	0.997	81
B.O.41	CRC.Oral	345	4.449	0.907	452	455	0.997	74
B.O.42	CRC.Oral	412	4.787	0.831	480	494	0.997	108
N.S.1	N.Stool	394	4.254	0.859	483	494	0.997	63
N.S.2	N.Stool	417	3.854	0.744	508	515	0.997	62
N.S.3	N.Stool	402	3.496	0.754	506	531	0.997	63
N.S.4	N.Stool	460	4.837	0.865	577	573	0.996	66
N.S.5	N.Stool	468	5.195	0.888	515	525	0.998	70
N.S.6	N.Stool	392	4.156	0.832	513	514	0.997	73
N.S.7	N.Stool	230	4.121	0.881	305	324	0.998	48
N.S.8	N.Stool	421	3.078	0.697	551	523	0.997	73
N.S.9	N.Stool	424	4.749	0.883	467	481	0.998	65
N.S.10	N.Stool	385	4.621	0.88	457	465	0.997	54
N.S.11	N.Stool	395	3.715	0.801	522	544	0.996	88
N.S.12	N.Stool	334	3.214	0.703	412	433	0.997	102
N.S.13	N.Stool	385	4.379	0.852	461	474	0.997	60
N.S.14	N.Stool	476	5.246	0.912	570	573	0.997	68
N.S.15	N.Stool	556	5.863	0.93	721	702	0.996	73
N.S.16	N.Stool	536	4.973	0.884	693	662	0.996	76
N.S.17	N.Stool	470	4.927	0.891	594	623	0.996	69
N.S.18	N.Stool	472	5.578	0.921	555	555	0.997	66
N.S.19	N.Stool	475	4.465	0.821	570	578	0.996	103
N.S.20	N.Stool	471	3.079	0.69	570	564	0.997	86
N.S.21	N.Stool	500	4.213	0.768	641	666	0.996	69
N.S.22	N.Stool	327	4.414	0.869	387	384	0.998	52
N.S.23	N.Stool	387	4.578	0.856	455	465	0.997	60
N.S.24	N.Stool	211	2.788	0.683	258	273	0.998	39
N.S.25	N.Stool	379	4.047	0.835	495	483	0.997	58
N.S.26	N.Stool	406	4.426	0.868	549	533	0.997	63
N.S.27	N.Stool	437	4.692	0.84	524	539	0.997	61
N.S.28	N.Stool	458	5.341	0.915	603	611	0.996	59
N.S.29	N.Stool	398	4.513	0.856	504	479	0.997	65
N.S.30	N.Stool	444	4.843	0.869	515	535	0.997	59
N.S.31	N.Stool	458	5.56	0.916	558	565	0.997	57
N.S.32	N.Stool	437	5.221	0.898	518	517	0.997	63
N.S.33	N.Stool	423	5.297	0.916	509	525	0.997	62

N.S.34	N.Stool	441	5.612	0.923	505	521	0.997	58
N.S.35	N.Stool	408	4.768	0.883	505	520	0.997	58
N.S.36	N.Stool	454	5.644	0.924	554	557	0.997	57
N.S.37	N.Stool	442	4.961	0.875	550	536	0.997	66
N.S.38	N.Stool	346	4.131	0.844	405	419	0.998	53
N.S.39	N.Stool	492	5.887	0.932	573	586	0.997	65
N.S.40	N.Stool	335	3.872	0.838	432	462	0.997	49
N.S.41	N.Stool	436	4.436	0.83	544	563	0.997	56
N.S.42	N.Stool	421	4.666	0.881	519	528	0.997	53
N.S.43	N.Stool	417	5.317	0.912	506	501	0.997	59
N.S.44	N.Stool	436	5.229	0.916	509	531	0.997	58
N.S.45	N.Stool	430	4.717	0.877	509	520	0.997	56
N.S.46	N.Stool	440	5.304	0.907	524	519	0.997	58
N.S.47	N.Stool	367	4.741	0.878	448	441	0.998	53
N.S.48	N.Stool	387	3.871	0.781	513	520	0.997	54
N.S.49	N.Stool	399	4.551	0.875	497	511	0.997	65
N.S.50	N.Stool	349	4.221	0.856	504	477	0.997	51
N.S.51	N.Stool	405	4.65	0.863	455	466	0.998	54
N.S.52	N.Stool	335	3.47	0.733	403	398	0.998	48
N.S.53	N.Stool	426	4.849	0.883	540	518	0.997	61
N.S.54	N.Stool	349	4.383	0.863	406	406	0.998	50
N.S.55	N.Stool	416	5.047	0.892	499	510	0.997	57
N.S.56	N.Stool	414	4.683	0.851	491	501	0.997	55
N.S.57	N.Stool	429	4.739	0.843	571	569	0.997	59
N.S.58	N.Stool	338	4.095	0.847	388	399	0.998	50
N.S.59	N.Stool	361	4.73	0.89	480	457	0.997	49
N.S.60	N.Stool	506	5.729	0.922	632	603	0.997	65
N.S.61	N.Stool	465	5.384	0.909	608	588	0.996	67
N.S.62	N.Stool	463	5.027	0.897	543	561	0.997	78
N.S.63	N.Stool	479	5.747	0.923	563	570	0.997	61
N.S.64	N.Stool	478	5.337	0.904	565	587	0.997	65
N.S.65	N.Stool	438	4.97	0.899	667	577	0.996	67
N.S.66	N.Stool	431	4.375	0.858	570	595	0.996	55
N.S.67	N.Stool	487	5.655	0.924	578	590	0.997	61
N.S.68	N.Stool	469	5.227	0.905	626	629	0.996	59
N.S.69	N.Stool	439	4.577	0.878	587	573	0.996	59
N.S.70	N.Stool	398	4.923	0.894	550	533	0.997	61
G.S.1	GC.Stool	357	4.895	0.896	479	439	0.997	50
G.S.2	GC.Stool	896	5.789	0.943	1207	1198	0.991	132
G.S.3	GC.Stool	311	3.75	0.741	361	384	0.998	44
G.S.4	GC.Stool	425	5.437	0.948	495	514	0.997	80
G.S.5	GC.Stool	162	1.876	0.444	215	232	0.998	40
G.S.6	GC.Stool	521	5.771	0.953	710	677	0.996	77
G.S.7	GC.Stool	471	6.535	0.979	511	528	0.998	63

G.S.8	GC.Stool	383	4.391	0.868	484	475	0.997	53
G.S.9	GC.Stool	781	5.556	0.943	1061	1049	0.993	129
G.S.10	GC.Stool	340	3.651	0.822	437	439	0.997	70
G.S.11	GC.Stool	458	5.923	0.968	575	564	0.997	77
G.S.12	GC.Stool	445	4.35	0.881	623	569	0.996	98
G.S.13	GC.Stool	410	5.448	0.948	545	526	0.997	59
G.S.14	GC.Stool	454	5.747	0.956	577	580	0.996	73
G.S.15	GC.Stool	401	4.261	0.785	510	527	0.997	66
G.S.16	GC.Stool	454	5.478	0.951	636	602	0.996	63
G.S.17	GC.Stool	369	5.103	0.923	458	457	0.997	65
G.S.18	GC.Stool	392	5.528	0.958	495	492	0.997	55
G.S.19	GC.Stool	405	5.119	0.928	498	504	0.997	69
G.S.20	GC.Stool	343	4.727	0.891	428	431	0.997	56
G.S.21	GC.Stool	391	5.281	0.942	470	475	0.997	63
G.S.22	GC.Stool	447	5.238	0.899	549	556	0.997	72
G.S.23	GC.Stool	424	5.308	0.945	627	554	0.996	73
G.S.24	GC.Stool	771	5.267	0.94	1038	1068	0.992	122
G.S.25	GC.Stool	415	5.598	0.956	502	499	0.997	70
G.S.26	GC.Stool	693	5.167	0.927	902	906	0.994	189
G.S.27	GC.Stool	814	5.806	0.95	956	1013	0.994	161
G.S.28	GC.Stool	454	4.301	0.791	545	575	0.997	61
G.S.29	GC.Stool	453	5.555	0.95	514	521	0.997	56
G.S.30	GC.Stool	473	5.985	0.963	579	550	0.997	59
G.S.31	GC.Stool	329	3.759	0.831	430	440	0.997	54
G.S.32	GC.Stool	452	5.102	0.939	630	596	0.996	62
G.S.33	GC.Stool	431	5.787	0.957	490	500	0.998	64
G.S.34	GC.Stool	387	3.492	0.691	473	476	0.997	63
G.S.35	GC.Stool	450	5.966	0.965	525	542	0.997	62
G.S.36	GC.Stool	441	5.637	0.955	530	532	0.997	57
G.S.37	GC.Stool	392	3.766	0.791	482	482	0.997	58
G.S.38	GC.Stool	587	5.623	0.945	689	718	0.996	73
G.S.39	GC.Stool	560	5.386	0.906	705	691	0.996	87
G.S.40	GC.Stool	495	6.076	0.965	610	597	0.997	68
G.S.41	GC.Stool	452	4.081	0.76	565	571	0.996	61
G.S.42	GC.Stool	419	4.861	0.925	569	586	0.996	174
G.S.43	GC.Stool	526	5.183	0.943	766	794	0.994	114
G.S.44	GC.Stool	524	3.772	0.753	783	826	0.994	101
G.S.45	GC.Stool	554	5.263	0.919	742	752	0.995	112
G.S.46	GC.Stool	513	5.744	0.956	627	644	0.996	92
G.S.47	GC.Stool	352	4.487	0.876	477	505	0.996	65
G.S.48	GC.Stool	799	6.962	0.981	934	955	0.995	107
G.S.49	GC.Stool	609	4.694	0.837	800	817	0.994	101
B.S.1	CRC.Stool	409	5.512	0.948	508	505	0.997	55
B.S.2	CRC.Stool	389	4.553	0.873	542	496	0.997	79

B.S.3	CRC.Stool	424	5.378	0.928	530	510	0.997	60
B.S.4	CRC.Stool	411	4.59	0.864	563	557	0.996	58
B.S.5	CRC.Stool	473	5.188	0.911	554	577	0.997	70
B.S.6	CRC.Stool	513	5.683	0.933	638	634	0.996	102
B.S.7	CRC.Stool	427	4.935	0.911	493	501	0.997	79
B.S.8	CRC.Stool	481	5.632	0.942	624	614	0.996	86
B.S.9	CRC.Stool	267	4.161	0.892	344	352	0.998	65
B.S.10	CRC.Stool	497	5.956	0.959	639	631	0.996	93
B.S.11	CRC.Stool	458	6.039	0.968	536	554	0.997	63
B.S.12	CRC.Stool	634	5.751	0.954	821	817	0.995	83
B.S.13	CRC.Stool	423	4.462	0.858	560	530	0.997	71
B.S.14	CRC.Stool	469	5.847	0.962	564	570	0.997	71
B.S.15	CRC.Stool	530	6.371	0.974	655	638	0.996	81
B.S.16	CRC.Stool	274	4.005	0.853	315	323	0.998	51
B.S.17	CRC.Stool	367	5.491	0.952	406	412	0.998	55
B.S.18	CRC.Stool	431	5.472	0.949	511	520	0.997	81
B.S.19	CRC.Stool	246	3.288	0.807	319	347	0.998	56
B.S.20	CRC.Stool	339	4.659	0.907	404	393	0.998	67
B.S.21	CRC.Stool	696	4.63	0.856	973	961	0.993	111
B.S.22	CRC.Stool	440	4.219	0.807	571	584	0.996	64
B.S.23	CRC.Stool	469	4.954	0.865	627	646	0.996	142
B.S.24	CRC.Stool	513	5.325	0.928	674	688	0.995	67
B.S.25	CRC.Stool	629	5.516	0.922	729	765	0.996	76
B.S.26	CRC.Stool	434	4.876	0.929	531	556	0.996	84
B.S.27	CRC.Stool	380	4.813	0.923	475	474	0.997	53
B.S.28	CRC.Stool	636	5.4	0.944	827	827	0.994	93
B.S.29	CRC.Stool	731	6.057	0.956	918	944	0.994	89
B.S.30	CRC.Stool	702	5.519	0.938	879	899	0.994	100
B.S.31	CRC.Stool	790	6.465	0.972	976	1002	0.994	122
B.S.32	CRC.Stool	686	5.811	0.956	866	870	0.994	89
B.S.33	CRC.Stool	661	4.804	0.848	827	829	0.995	101
G.T.1	GC.Tumor	361	4.125	0.842	420	442	0.997	318
G.T.2	GC.Tumor	262	4.628	0.929	306	309	0.998	200
G.T.3	GC.Tumor	2095	5.649	0.851	2773	2746	0.979	1029
G.T.4	GC.Tumor	225	2.219	0.605	305	301	0.998	172
G.T.5	GC.Tumor	368	2.759	0.706	475	472	0.997	367
G.T.6	GC.Tumor	399	2.154	0.615	568	565	0.996	469
G.T.7	GC.Tumor	932	4.609	0.846	1246	1305	0.99	332
G.T.8	GC.Tumor	742	2.77	0.498	992	1051	0.992	1404
G.T.9	GC.Tumor	289	2.555	0.723	380	412	0.997	301
G.T.10	GC.Tumor	539	4.063	0.808	658	670	0.996	916
G.T.11	GC.Tumor	593	3.508	0.742	864	857	0.993	947
G.T.12	GC.Tumor	1620	6.973	0.964	1936	1944	0.988	199
G.T.13	GC.Tumor	3087	6.396	0.835	3476	3625	0.975	1163

G.T.14	GC.Tumor	667	3.309	0.587	901	911	0.994	1193
G.T.15	GC.Tumor	606	3.345	0.72	746	736	0.996	628
G.T.16	GC.Tumor	383	0.75	0.117	523	561	0.995	149
G.T.17	GC.Tumor	1944	6.202	0.902	2754	2934	0.977	453
G.T.18	GC.Tumor	220	2.863	0.693	314	344	0.997	72
G.T.19	GC.Tumor	1461	6.278	0.918	1794	1844	0.987	237
G.T.20	GC.Tumor	1303	5.355	0.852	1683	1769	0.987	170
G.T.21	GC.Tumor	886	4.478	0.718	957	966	0.996	270
G.T.22	GC.Tumor	1866	5.184	0.802	2507	2609	0.98	842
G.T.23	GC.Tumor	1601	6.065	0.898	2008	2029	0.986	269
G.T.24	GC.Tumor	1528	3.281	0.56	2420	2533	0.979	1586
G.T.25	GC.Tumor	252	3.583	0.798	310	322	0.998	94
G.T.26	GC.Tumor	1356	4.44	0.662	1827	1840	0.986	817
G.T.27	GC.Tumor	741	3.376	0.683	1020	1031	0.992	614
G.T.28	GC.Tumor	419	1.16	0.272	681	831	0.993	298
G.T.29	GC.Tumor	1369	5.71	0.9	1859	1954	0.985	213
G.T.30	GC.Tumor	239	1.012	0.192	288	295	0.998	65
G.T.31	GC.Tumor	719	2.306	0.406	1078	1094	0.991	1122
G.T.32	GC.Tumor	329	3.513	0.758	403	407	0.998	85
G.T.33	GC.Tumor	919	5.531	0.927	1208	1247	0.991	309
G.P.1	GC.Para	2112	8.977	0.994	2322	2387	0.988	334
G.P.2	GC.Para	848	5.142	0.929	1185	1311	0.99	218
G.P.3	GC.Para	236	1.878	0.501	304	307	0.998	66
G.P.4	GC.Para	154	0.469	0.089	236	281	0.998	50
G.P.5	GC.Para	260	2.371	0.662	312	320	0.998	189
G.P.6	GC.Para	384	1.935	0.502	513	508	0.996	170
G.P.7	GC.Para	290	3.19	0.698	348	365	0.998	87
G.P.8	GC.Para	293	3.024	0.759	382	385	0.997	377
G.P.9	GC.Para	152	1.732	0.524	194	201	0.999	67
G.P.10	GC.Para	2102	8.958	0.994	2319	2354	0.988	379
G.P.11	GC.Para	197	1.579	0.474	274	279	0.998	81
G.P.12	GC.Para	260	4.059	0.817	321	326	0.998	208
G.P.13	GC.Para	3499	8.674	0.971	3978	4188	0.973	1045
G.P.14	GC.Para	2832	8.617	0.977	3681	3647	0.974	653
G.P.15	GC.Para	239	4.057	0.829	279	285	0.999	87
G.P.16	GC.Para	220	3.895	0.817	262	269	0.999	101
G.P.17	GC.Para	1976	8.611	0.989	2166	2246	0.989	501
G.P.18	GC.Para	2069	8.813	0.993	2342	2419	0.987	432
G.P.19	GC.Para	212	1.057	0.213	283	297	0.998	90
G.P.20	GC.Para	308	4.97	0.94	366	378	0.998	299
G.P.21	GC.Para	1209	4.872	0.874	1682	1761	0.986	160
G.P.22	GC.Para	812	4.227	0.812	1045	1083	0.992	979
G.P.23	GC.Para	821	4.04	0.765	1201	1210	0.99	902
G.P.24	GC.Para	589	1.379	0.297	835	916	0.992	647

G.P.25	GC.Para	1048	3.458	0.59	1400	1510	0.988	189
G.P.26	GC.Para	1148	7.02	0.974	1398	1402	0.991	150
G.P.27	GC.Para	440	1.249	0.254	602	632	0.995	997
G.P.28	GC.Para	363	4.888	0.922	415	416	0.998	130
G.P.29	GC.Para	623	3.649	0.642	799	808	0.995	754
G.P.30	GC.Para	522	2.36	0.466	624	644	0.996	187
G.P.31	GC.Para	2026	5.285	0.701	2707	2614	0.981	349
G.P.32	GC.Para	1177	3.945	0.673	1547	1588	0.988	263
G.P.33	GC.Para	970	2.753	0.445	1341	1445	0.989	1442
G.P.34	GC.Para	312	1.687	0.378	364	370	0.998	186
G.P.35	GC.Para	271	1.159	0.218	333	347	0.998	66
G.P.36	GC.Para	255	3.101	0.706	330	349	0.998	74
B.T.1	CRC.Tumor	218	3.661	0.811	254	255	0.999	81
B.T.2	CRC.Tumor	230	4.213	0.888	278	278	0.998	60
B.T.3	CRC.Tumor	298	5.165	0.94	352	348	0.998	130
B.T.4	CRC.Tumor	944	3.463	0.708	1597	1745	0.985	465
B.T.5	CRC.Tumor	3072	9.409	0.991	3458	3564	0.98	794
B.T.6	CRC.Tumor	318	4.38	0.874	436	402	0.997	170
B.T.7	CRC.Tumor	223	3.775	0.844	286	285	0.998	119
B.T.8	CRC.Tumor	450	2.513	0.572	709	717	0.994	163
B.T.9	CRC.Tumor	240	2.374	0.576	332	335	0.997	96
B.T.10	CRC.Tumor	213	3.856	0.88	278	293	0.998	127
B.T.11	CRC.Tumor	1309	5.541	0.904	2000	2082	0.983	192
B.T.12	CRC.Tumor	391	1.396	0.322	694	777	0.993	195
B.T.13	CRC.Tumor	1483	5.877	0.906	2258	2390	0.981	1343
B.T.14	CRC.Tumor	287	3.33	0.712	366	360	0.998	87
B.T.15	CRC.Tumor	2220	6.661	0.897	2753	2726	0.982	437
B.T.16	CRC.Tumor	114	4.294	0.913	163	188	0.999	96
B.T.17	CRC.Tumor	1359	5.082	0.872	1946	2089	0.983	282
B.T.18	CRC.Tumor	257	2.12	0.436	314	316	0.998	61
B.T.19	CRC.Tumor	946	6.247	0.957	1269	1293	0.991	799
B.T.20	CRC.Tumor	379	2.107	0.467	625	653	0.995	222
B.T.21	CRC.Tumor	792	5.748	0.929	943	984	0.994	606
B.T.22	CRC.Tumor	629	4.158	0.727	788	780	0.995	99
B.T.23	CRC.Tumor	424	4.466	0.856	509	542	0.996	219
B.T.24	CRC.Tumor	1046	4.47	0.762	1316	1343	0.991	1336
B.P.1	CRC.Para	255	4.974	0.918	299	310	0.998	135
B.P.2	CRC.Para	443	4.783	0.915	594	585	0.996	101
B.P.3	CRC.Para	525	6.15	0.962	688	660	0.996	129
B.P.4	CRC.Para	362	5.33	0.953	463	491	0.997	83
B.P.5	CRC.Para	424	5.275	0.947	551	589	0.996	87
B.P.6	CRC.Para	315	4.399	0.907	411	407	0.997	118
B.P.7	CRC.Para	328	3.952	0.878	434	430	0.997	124
B.P.8	CRC.Para	913	5.497	0.941	1234	1290	0.99	410

B.P.9	CRC.Para	2134	6.564	0.838	2520	2482	0.985	386
B.P.10	CRC.Para	197	3.503	0.827	277	245	0.998	77
B.P.11	CRC.Para	453	5.469	0.943	668	655	0.995	170
B.P.12	CRC.Para	278	5.116	0.951	353	374	0.998	296
B.P.13	CRC.Para	344	5.525	0.954	415	408	0.998	77
B.P.14	CRC.Para	317	3.539	0.726	383	407	0.997	108
B.P.15	CRC.Para	292	4.434	0.888	375	399	0.997	122
B.P.16	CRC.Para	307	5.716	0.965	398	379	0.998	106
B.P.17	CRC.Para	195	3.176	0.789	302	279	0.998	90
B.P.18	CRC.Para	318	3.521	0.783	463	453	0.997	68
B.P.19	CRC.Para	390	4.395	0.883	450	459	0.997	172
B.P.20	CRC.Para	289	3.135	0.647	360	375	0.998	169
B.P.21	CRC.Para	509	5.024	0.911	701	688	0.995	474
B.P.22	CRC.Para	468	5.085	0.932	600	641	0.996	320
B.P.23	CRC.Para	420	5.273	0.936	496	508	0.997	83
B.P.24	CRC.Para	511	2.401	0.496	632	666	0.995	741
