# Supplementary material for: Combined Non-Invasive Prediction and New Biomarkers of Oral and Fecal Microbiota in Patients With Gastric and Colorectal Cancer
Source: Front Cell Infect Microbiol. 2022 May 19;12:830684. doi: 10.3389/fcimb.2022.830684 (PMC9161364; doi:10.3389/fcimb.2022.830684)
Supplement: Supplementary file 1 [file DataSheet_1.zip › Supplementary Table 3.pdf]

Table S3. Metastases analysis of phylum and genus levels of oral samples of HC, GC and CRC

Phylum	mean. group1.	variance. group1.	standard. error. group1.	mean. group2.
**GC.Oral-CRC.Oral-Phylum**				
p__Crenarchaeota	2.67E-05	2.27E-08	2.33E-05	4.21E-07
p__Entotheonellaeota	0	0	0	9.27E-06
p__Fusobacteriota	0.060154591	0.002507557	0.00772682	0.094422435
p__NB1-j	5.62E-06	3.07E-10	2.70E-06	1.26E-06
p__Gracilibacteria	0.000433399	3.97E-07	9.72E-05	0.001078931
p__Zixibacteria	0	0	0	3.37E-06
**GC.Oral-N.Oral-Phylum**				
p__Gemmatimonadetes	2.53E-05	2.85E-08	2.02E-05	2.95E-06
p__Thermoplasmatota	1.60E-05	1.28E-08	1.35E-05	1.26E-06
p__Entotheonellaeota	9.27E-06	3.37E-09	6.94E-06	8.43E-07
p__Sva0485	1.14E-05	1.29E-09	4.30E-06	2.11E-06
p__Calditrichota	5.48E-06	4.36E-10	2.50E-06	0
p__Gemmatimonadota	5.90E-06	6.21E-10	2.98E-06	4.21E-07
p__Proteobacteria	0.260414208	0.024120382	0.018562782	0.360015931
p__Firmicutes	0.370889949	0.01704537	0.015604656	0.292115378
p__Nitrospirota	8.43E-07	4.97E-11	8.43E-07	6.32E-06
p__Berkelbacteria	0	0	0	3.79E-06
p__Zixibacteria	3.37E-06	1.40E-10	1.41E-06	0
p__Methylomirabilota	1.69E-06	1.99E-10	1.69E-06	5.90E-06
**CRC.Oral-N.Oral-Phylum**				
p__Crenarchaeota	2.67E-05	2.27E-08	2.33E-05	2.53E-06
p__Gemmatimonadetes	2.04E-05	6.35E-09	1.23E-05	2.95E-06
p__Calditrichota	9.13E-06	7.00E-10	4.08E-06	0
p__Gemmatimonadota	8.43E-06	8.19E-10	4.42E-06	4.21E-07
p__Thermoplasmatota	1.05E-05	1.82E-09	6.58E-06	1.26E-06
p__NB1-j	5.62E-06	3.07E-10	2.70E-06	0
p__Firmicutes	0.388701575	0.02130765	0.022523876	0.292115378
p__Methylomirabilota	0	0	0	5.90E-06
p__Gracilibacteria	0.000433399	3.97E-07	9.72E-05	0.001226019
p__Fusobacteriota	0.060154591	0.002507557	0.00772682	0.085375856
p__Berkelbacteria	0	0	0	3.79E-06
p__Sva0485	7.73E-06	7.67E-10	4.27E-06	2.11E-06
p__Elusimicrobia	0	0	0	4.21E-06
p__Proteobacteria	0.287648774	0.039034005	0.030485752	0.360015931

variance. group2.	standard. error. group2.	p.value	q.value	FC	log10FC
**GC.Oral-CRC.Oral-Phylum**					
1.24E-11	4.21E-07	0.0000	0.0000	0.9744	-0.0113
3.37E-09	6.94E-06	0.0001	0.0026	1.0093	0.0040
0.004996137	0.008448277	0.0040	0.1172	1.5603	0.1932
3.62E-11	7.19E-07	0.0250	0.4237	0.9957	-0.0019
5.67E-06	0.000284514	0.0270	0.4237	1.4504	0.1615
**GC.Oral-N.Oral-Phylum**					
1.55E-10	1.49E-06	0.0000	0.0000	0.9782	-0.0096
3.62E-11	7.19E-07	0.0000	0.0000	0.9855	-0.0064
2.45E-11	5.92E-07	0.0000	0.0006	0.9916	-0.0036
2.10E-10	1.73E-06	0.0001	0.0017	0.9908	-0.0040
0	0	0.0002	0.0031	0.9946	-0.0024
1.24E-11	4.21E-07	0.0010	0.0095	0.9946	-0.0024
0.022835046	0.018061421	0.0020	0.0160	1.3810	0.1402
0.010900234	0.012478687	0.0020	0.0160	0.7882	-0.1034
5.52E-10	2.81E-06	0.0023	0.0172	1.0055	0.0024
5.53E-10	2.81E-06	0.0039	0.0246	1.0038	0.0016
0	0	0.0078	0.0458	0.9966	-0.0015
9.23E-10	3.63E-06	0.0309	0.1618	1.0042	0.0018
**CRC.Oral-N.Oral-Phylum**					
2.46E-10	1.87E-06	0.0000	0.0000	0.9765	-0.0103
1.55E-10	1.49E-06	0.0000	0.0000	0.9829	-0.0075
0	0	0.0000	0.0001	0.9910	-0.0039
1.24E-11	4.21E-07	0.0001	0.0010	0.9921	-0.0035
3.62E-11	7.19E-07	0.0001	0.0011	0.9908	-0.0040
0	0	0.0004	0.0043	0.9944	-0.0024
0.010900234	0.012478687	0.0010	0.0098	0.7522	-0.1237
9.23E-10	3.63E-06	0.0017	0.0149	1.0059	0.0026
8.42E-06	0.0003468	0.0050	0.0366	1.5530	0.1912
0.002469062	0.005939051	0.0130	0.0877	1.4124	0.1500
5.53E-10	2.81E-06	0.0169	0.0877	1.0038	0.0016
2.10E-10	1.73E-06	0.0169	0.0877	0.9944	-0.0024
2.59E-10	1.93E-06	0.0169	0.0877	1.0042	0.0018
0.022835046	0.018061421	0.0390	0.1805	1.2507	0.0972

Genus	mean. group1.	variance. group1.	standard. error. group1.	mean. group2.
**GC.Oral-CRC.Oral-Phylum**				
g__Sorangium	2.74E-05	2.99E-08	2.67E-05	0
g__Candidatus_Koribacter	2.88E-05	3.48E-08	2.88E-05	1.26E-06

g__Methylorosula	2.39E-05	2.26E-08	2.32E-05	8.43E-07
g__Lacunisphaera	1.83E-05	1.40E-08	1.83E-05	0
g__Turicibacter	2.74E-05	1.21E-08	1.70E-05	2.53E-06
g__[Eubacterium]_ruminantium_group	2.60E-05	1.14E-08	1.65E-05	2.11E-06
g__Acidothermus	1.69E-05	1.10E-08	1.62E-05	0
g__Stenotrophobacter	2.11E-06	1.87E-10	2.11E-06	2.70E-05
g__Salinisphaera	0	0	0	1.94E-05
g__Sediminispirochaeta	1.97E-05	1.51E-08	1.90E-05	1.26E-06
g__Brochothrix	1.83E-05	1.40E-08	1.83E-05	1.26E-06
g__Dongia	1.33E-05	7.48E-09	1.33E-05	0
g__SWB02	1.26E-05	5.99E-09	1.19E-05	0
g__Arcobacter	0	0	0	1.56E-05
g__Rhodanobacter	2.88E-05	2.99E-08	2.67E-05	6.32E-06
g__Rahnella1	1.33E-05	7.48E-09	1.33E-05	4.21E-07
g__Agromyces	1.12E-05	2.59E-09	7.85E-06	0
g__Anaerobacillus	2.04E-05	1.62E-08	1.97E-05	2.95E-06
g__Quadrisphaera	7.02E-07	2.07E-11	7.02E-07	1.60E-05
g__Blastocatella	1.05E-05	4.66E-09	1.05E-05	0
g__Anaerosporomusa	1.40E-06	8.29E-11	1.40E-06	1.73E-05
g__Deinococcus	2.11E-06	1.02E-10	1.56E-06	1.94E-05
g__Parapusillimonas	9.83E-06	4.06E-09	9.83E-06	0
g__Ohtaekwangia	9.13E-06	3.50E-09	9.13E-06	0
g__Prevotellaceae_UCG-003	7.02E-07	2.07E-11	7.02E-07	1.39E-05
g__Brevibacterium	1.33E-05	7.48E-09	1.33E-05	1.26E-06
g__Parascardovia	3.51E-06	2.21E-10	2.29E-06	2.07E-05
g__Ellin6055	3.51E-06	2.21E-10	2.29E-06	2.11E-05
g__Zoogloea	1.19E-05	2.04E-09	6.97E-06	8.43E-07
g__Actinocorallia	1.26E-05	5.99E-09	1.19E-05	1.26E-06
g__Lysobacter	0	0	0	1.05E-05
g__Hymenobacter	2.81E-06	3.32E-10	2.81E-06	1.77E-05
g__Flavitalea	9.13E-06	3.50E-09	9.13E-06	4.21E-07
g__Tepidimonas	1.48E-05	5.36E-09	1.13E-05	2.53E-06
g__Candidatus_Entotheonella	0	0	0	9.27E-06
g__Succinivibrio	6.32E-06	8.29E-10	4.44E-06	0
g__Candidatus_Solibacter	7.73E-06	1.74E-09	6.44E-06	4.21E-07
g__Adhaeribacter	5.62E-06	4.35E-10	3.22E-06	0
g__Pseudonocardia	7.02E-06	1.35E-09	5.67E-06	4.21E-07
g__Luteibacter	1.05E-05	4.66E-09	1.05E-05	1.69E-06
g__Roseomonas	9.13E-06	2.99E-09	8.44E-06	1.26E-06
g__Paracoccus	2.81E-06	1.19E-10	1.69E-06	1.39E-05
g__Halomonas	0	0	0	7.16E-06
g__Candidatus_Methanomethylophilus	4.21E-06	1.94E-10	2.15E-06	1.60E-05
g__Aerococcus	1.40E-06	8.29E-11	1.40E-06	1.01E-05
g__Microbacterium	6.32E-06	1.04E-09	4.98E-06	4.21E-07

g__Lachnoanaerobaculum	0.000842212	6.74E-07	0.000126716	0.001789507
g__Desulfomicrobium	2.46E-05	2.88E-09	8.29E-06	0.000294177
g__Microvirga	5.48E-05	9.65E-08	4.79E-05	0
g__Candidatus_Udaeobacter	6.32E-05	4.80E-08	3.38E-05	0
g__Parafilimonas	0.000137676	7.63E-07	0.000134816	0
g__FFCH7168	6.18E-05	1.50E-07	5.97E-05	0
g__Luedemannella	3.02E-05	2.05E-08	2.21E-05	0
g__unidentified_Muribaculaceae	4.92E-06	1.02E-09	4.92E-06	0
g__Parvibaculum	4.92E-06	7.61E-10	4.26E-06	0
g__Streptosporangium	4.92E-06	1.02E-09	4.92E-06	0
g__Altererythrobacter	4.92E-06	1.02E-09	4.92E-06	0
g__Aridibacter	4.92E-06	7.61E-10	4.26E-06	0
g__Bradyrhizobium	1.19E-05	3.61E-09	9.27E-06	2.95E-06
g__ADurb.Bin063-1	0	0	0	6.32E-06
g__Oligella	2.81E-06	1.19E-10	1.69E-06	1.22E-05
g__Alcanivorax	7.02E-07	2.07E-11	7.02E-07	0.000297549
g__Acidovorax	1.55E-05	2.05E-09	6.98E-06	5.06E-06
g__Rhodoferax	4.21E-06	7.46E-10	4.21E-06	0
g__Ellin6067	4.21E-06	7.46E-10	4.21E-06	0
g__Hydrogenophaga	4.21E-06	7.46E-10	4.21E-06	0
g__Silvanigrella	4.21E-06	7.46E-10	4.21E-06	0
g__Methylobacillus	4.21E-06	5.34E-10	3.56E-06	0
g__Coriobacteriaceae_UCG-002	0	0	0	5.48E-06
g__Sporosarcina	7.02E-07	2.07E-11	7.02E-07	7.80E-05
g__Desulfitibacter	1.33E-05	4.81E-09	1.07E-05	4.21E-06
g__Cellulosilyticum	6.32E-06	8.29E-10	4.44E-06	8.43E-07
g__Allisonella	7.02E-06	4.16E-10	3.15E-06	1.81E-05
g__Prevotellaceae_UCG-004	3.51E-06	1.78E-10	2.06E-06	3.16E-05
g__Phenylobacterium	0	0	0	5.06E-06
g__Rubellimicrobium	0	0	0	5.06E-06
g__Anaeroplasma	5.62E-06	5.20E-10	3.52E-06	1.56E-05
g__Erysipelotrichaceae_UCG-006	1.48E-05	7.75E-10	4.30E-06	2.91E-05
g__Stenotrophomonas	1.40E-06	4.04E-11	9.81E-07	8.43E-06
g__Leptotrichia	0.022063286	0.000810758	0.004393604	0.043821016
g__Schlegelella	6.32E-06	8.29E-10	4.44E-06	0.000334216
g__MND1	3.51E-06	2.21E-10	2.29E-06	0
g__Rosenbergiella	3.51E-06	5.18E-10	3.51E-06	0
g__Pseudoclostridium	1.62E-05	1.00E-08	1.55E-05	6.74E-06
g__Eggerthella	4.21E-06	1.52E-10	1.90E-06	1.26E-05
g__Nocardioides	3.51E-06	3.48E-10	2.88E-06	1.14E-05
g__Paenarthrobacter	6.32E-06	8.29E-10	4.44E-06	1.26E-06
g__Rubrobacter	4.21E-06	7.46E-10	4.21E-06	4.21E-07
g__Eisenbergiella	0	0	0	3.79E-06
g__Succiniclasticum	0	0	0	3.79E-06

g__Desulfotomaculum	0	0	0	3.79E-06
g__Rikenella	0	0	0	4.21E-06
g__JG30a-KF-32	2.81E-06	3.32E-10	2.81E-06	0
g__Caldithrix	2.81E-06	1.19E-10	1.69E-06	0
g__DTU089	7.73E-06	7.24E-10	4.15E-06	1.69E-05
g__Mitsuokella	7.02E-07	2.07E-11	7.02E-07	5.48E-06
g__Johnsonella	0.00046571	4.48E-07	0.000103234	0.000880424
g__Moraxella	0.000342785	1.29E-06	0.000175443	0.005340706
g__Lachnospiraceae_UCG-006	4.21E-06	2.79E-10	2.58E-06	1.10E-05
g__Azospira	0	0	0	3.37E-06
g__unidentified_Spirochaetaceae	0	0	0	3.37E-06
g__Gramella	0	0	0	3.37E-06
g__[Eubacterium]_yurii_group	3.58E-05	6.60E-09	1.25E-05	9.23E-05
g__Brachybacterium	4.92E-06	3.79E-10	3.00E-06	8.43E-07
g__Butyrivibrio	2.53E-05	3.38E-09	8.97E-06	5.39E-05
g__Bulleidia	1.76E-05	2.59E-09	7.86E-06	9.27E-06
g__Cryptobacterium	1.26E-05	8.55E-10	4.51E-06	2.23E-05
g__Gaiella	7.02E-07	2.07E-11	7.02E-07	4.64E-06
g__Methyloversatilis	2.11E-06	1.02E-10	1.56E-06	7.59E-06
**GC.Oral-N.Oral-Genus**				
g__Stenotrophobacter	2.70E-05	5.09E-08	2.70E-05	0
g__CAG-352	4.21E-07	1.24E-11	4.21E-07	2.70E-05
g__Gemmatimonas	2.49E-05	2.85E-08	2.02E-05	1.26E-06
g__Salinisphaera	1.94E-05	1.71E-08	1.56E-05	0
g__Anaerosporomusa	1.73E-05	1.08E-08	1.24E-05	0
g__unidentified_Clostridiaceae	1.69E-05	3.50E-09	7.07E-06	0
g__Ellin6055	2.11E-05	2.11E-08	1.74E-05	1.26E-06
g__Adlercreutzia	2.95E-06	2.31E-10	1.82E-06	2.57E-05
g__Massilia	4.21E-06	1.84E-10	1.62E-06	2.87E-05
g__Arcobacter	1.56E-05	1.70E-08	1.56E-05	0
g__[Eubacterium]_xylanophilum_group	4.21E-06	2.34E-10	1.83E-06	2.82E-05
g__Senegalimassilia	2.11E-06	1.34E-10	1.38E-06	2.19E-05
g__Catenibacterium	3.37E-06	2.66E-10	1.95E-06	2.49E-05
g__Sediminispirochaeta	1.26E-06	1.12E-10	1.26E-06	1.90E-05
g__Geobacillus	2.11E-05	1.66E-08	1.54E-05	2.11E-06
g__Curvibacter	0	0	0	1.31E-05
g__Providencia	1.56E-05	1.61E-08	1.52E-05	8.43E-07
g__Frisingicoccus	6.32E-06	7.29E-10	3.23E-06	2.82E-05
g__Candidatus_Methanomethylophilus	1.60E-05	1.28E-08	1.35E-05	1.26E-06
g__Rhodoferax	0	0	0	1.14E-05
g__Thermus	1.85E-05	5.93E-09	9.21E-06	2.53E-06
g__Polynucleobacter	0	0	0	1.05E-05
g__DTU089	1.69E-05	3.77E-09	7.34E-06	2.11E-06

g__Peptoclostridium	0	0	0	1.01E-05
g__Lachnospiraceae_FCS020_group	2.11E-06	8.38E-11	1.09E-06	1.60E-05
g__Parascardovia	2.07E-05	1.24E-08	1.33E-05	4.21E-06
g__A2	2.11E-06	1.34E-10	1.38E-06	1.56E-05
g__Sva0081_sediment_group	9.27E-06	1.28E-09	4.27E-06	0
g__[Clostridium]_innocuum_group	1.69E-06	7.28E-11	1.02E-06	1.39E-05
g__Rhodobacter	0	0	0	8.43E-06
g__Candidatus_Stoquefichus	7.59E-06	1.08E-09	3.92E-06	2.49E-05
g__Alcaligenes	8.43E-07	4.97E-11	8.43E-07	1.05E-05
g__Lysobacter	1.05E-05	6.59E-09	9.70E-06	8.43E-07
g__Exiguobacterium	2.23E-05	4.91E-09	8.37E-06	6.74E-06
g__Aerococcus	1.01E-05	5.52E-09	8.88E-06	8.43E-07
g__Thauera	0	0	0	7.16E-06
g__Anaerotruncus	1.10E-05	2.45E-09	5.92E-06	2.87E-05
g__UCG-003	8.01E-06	1.11E-09	3.98E-06	2.36E-05
g__Sva0996_marine_group	6.74E-06	5.59E-10	2.83E-06	0
g__Pseudoclostridium	6.74E-06	3.18E-09	6.74E-06	0
g__Rhodoluna	0	0	0	6.74E-06
g__Pelospora	6.74E-06	5.34E-10	2.76E-06	2.11E-05
g__Candidatus_Entotheonella	9.27E-06	3.37E-09	6.94E-06	8.43E-07
g__Caldibacillus	1.22E-05	7.83E-09	1.06E-05	2.11E-06
g__Family_XIII_AD3011_group	1.01E-05	1.11E-09	3.98E-06	2.61E-05
g__Rhodanobacter	6.32E-06	1.23E-09	4.20E-06	0
g__Erysipelotrichaceae_UCG-002	0	0	0	6.32E-06
g__Brevifollis	0	0	0	6.32E-06
g__Erysipelotrichaceae_UCG-006	2.91E-05	7.71E-09	1.05E-05	1.22E-05
g__Lachnospiraceae_UCG-006	1.10E-05	3.51E-09	7.08E-06	2.70E-05
g__Shewanella	8.43E-06	1.32E-09	4.34E-06	8.43E-07
g__Cetobacterium	0	0	0	5.90E-06
g__P3OB-42	5.90E-06	1.30E-09	4.31E-06	0
g__MND1	0	0	0	5.90E-06
g__Eggerthella	1.26E-05	5.67E-09	9.00E-06	2.87E-05
g__Eisenbergiella	3.79E-06	1.01E-09	3.79E-06	1.43E-05
g__Hymenobacter	1.77E-05	5.54E-09	8.89E-06	5.90E-06
g__Woeseia	8.85E-06	1.02E-09	3.81E-06	1.26E-06
g__Quadrisphaera	1.60E-05	3.17E-09	6.73E-06	5.06E-06
g__Ralstonia	1.39E-05	7.97E-09	1.07E-05	2.91E-05
g__Anaerococcus	1.26E-05	4.15E-09	7.70E-06	3.37E-06
g__Aliivibrio	5.06E-06	1.79E-09	5.06E-06	0
g__Succinivibrio	0	0	0	5.06E-06
g__Clostridium_sensu_stricto_7	0	0	0	5.06E-06
g__Rubellimicrobium	5.06E-06	7.31E-10	3.23E-06	0
g__Syntrophotalea	5.06E-06	8.82E-10	3.55E-06	0
g__Runella	0	0	0	5.06E-06

g__ADurb.Bin063-1	6.32E-06	2.80E-09	6.32E-06	4.21E-07
g__Mitsuokella	5.48E-06	6.89E-10	3.14E-06	1.60E-05
g__Sellimonas	2.07E-05	5.69E-09	9.01E-06	8.43E-06
g__Ellin6067	0	0	0	4.64E-06
g__Hydrogenophaga	0	0	0	4.64E-06
g__Dechloromonas	0	0	0	4.64E-06
g__Herbaspirillum	0.013847399	0.011255465	0.012680393	2.95E-06
g__Haemophilus	0.077064298	0.004751207	0.008238591	0.142701625
g__Gemella	0.041098402	0.002104126	0.005482603	0.020282208
g__Abiotrophia	0.008155197	0.000511417	0.002702953	0.00215744
g__Sarcina	4.76E-05	1.45E-07	4.55E-05	0
g__Aggregatibacter	0.008849759	8.10E-05	0.001075634	0.021785124
g__Megasphaera	0.000194713	6.16E-08	2.97E-05	0.000406285
g__hgcI_clade	0	0	0	0.000439158
g__Schlegelella	0.000334216	5.83E-06	0.000288471	0
g__Turicibacter	2.53E-06	9.44E-11	1.16E-06	2.95E-05
g__Acetoanaerobium	0.000238545	3.65E-06	0.000228333	0
g__Vagococcus	0.000203985	2.71E-06	0.000196749	0
g__Vulcaniibacterium	0.000330844	2.82E-06	0.000200682	4.21E-07
g__Limnohabitans	0	0	0	2.95E-05
g__Erysipelothrix	5.86E-05	2.30E-07	5.73E-05	0
g__Dechlorobacter	5.61E-05	2.00E-07	5.35E-05	0
g__Desulfobulbus	2.40E-05	1.72E-09	4.96E-06	5.82E-05
g__Tepidibacter	4.64E-05	2.97E-08	2.06E-05	0
g__Amnipila	1.35E-05	1.15E-09	4.06E-06	8.77E-05
g__Granulicella	4.21E-06	3.10E-10	2.10E-06	0
g__Acetitomaculum	0	0	0	4.21E-06
g__Lachnospiraceae_NC2004_group	0	0	0	4.21E-06
g__Buchnera	0	0	0	4.21E-06
g__Desulfitibacter	4.21E-06	1.24E-09	4.21E-06	0
g__Desulfatitalea	4.21E-06	2.34E-10	1.83E-06	0
g__Alcanivorax	0.000297549	4.83E-06	0.000262713	1.69E-06
g__Sphingomonas	0.000398699	4.36E-06	0.000249484	3.03E-05
g__Sphaerochaeta	6.74E-05	3.12E-08	2.11E-05	1.52E-05
g__Atopostipes	5.90E-06	4.69E-10	2.59E-06	1.52E-05
g__Proteiniphilum	0.000450959	1.25E-05	0.000422851	2.95E-06
g__[Eubacterium]_brachy_group	0.00014751	3.72E-08	2.31E-05	0.000253717
g__Peptoniphilus	4.21E-07	1.24E-11	4.21E-07	5.06E-06
g__Nesterenkonia	0	0	0	3.79E-06
g__Desulfotomaculum	3.79E-06	1.01E-09	3.79E-06	0
g__Prosthecobacter	0	0	0	3.79E-06
g__Desulfatiglans	3.79E-06	1.49E-10	1.46E-06	0
g__Streptococcus	0.256053812	0.015125187	0.014699459	0.197415624
g__Vibrio	0.000483833	2.70E-06	0.000196297	6.32E-06

g__Methyloversatilis	7.59E-06	1.63E-09	4.83E-06	1.69E-06
g__Candidatus_Soleaferrea	1.69E-06	7.28E-11	1.02E-06	7.59E-06
g__Brachymonas	1.69E-06	7.28E-11	1.02E-06	7.59E-06
g__Bulleidia	9.27E-06	3.92E-10	2.37E-06	1.94E-05
g__Actinomyces	0.029290013	0.002615265	0.006112359	0.014926751
g__Brevundimonas	1.22E-05	9.71E-10	3.72E-06	4.17E-05
g__Paraclostridium	0.000256667	9.03E-07	0.000113591	1.26E-06
g__Gaiella	4.64E-06	1.05E-09	3.87E-06	4.21E-07
g__Flavisolibacter	3.37E-06	4.93E-10	2.65E-06	0
g__Listeria	0	0	0	3.37E-06
g__Chryseomicrobium	0	0	0	3.37E-06
g__unidentified_Spirochaetaceae	3.37E-06	7.96E-10	3.37E-06	0
g__Anaeromicrobium	3.37E-06	3.42E-10	2.21E-06	0
g__Komagataeibacter	0	0	0	3.37E-06
g__Actibacter	3.37E-06	3.16E-10	2.13E-06	0
g__unidentified_Gastranaerophilales	5.90E-06	2.44E-09	5.90E-06	1.39E-05
g__Alysiella	2.95E-06	1.80E-10	1.61E-06	9.27E-06
g__Johnsonella	0.000880424	1.40E-06	0.000141346	0.000503641
g__Family_XIII_UCG-001	2.74E-05	1.90E-09	5.21E-06	4.80E-05
g__Oligella	1.22E-05	3.82E-09	7.39E-06	5.06E-06
g__Candidatus_Solibacter	4.21E-07	1.24E-11	4.21E-07	4.21E-06
g__Kroppenstedtia	4.21E-07	1.24E-11	4.21E-07	4.21E-06
g__Phocaeicola	9.99E-05	4.36E-08	2.50E-05	3.88E-05
g__Candidatus_Arthromitus	9.27E-06	2.76E-09	6.28E-06	1.81E-05
g__Sneathia	8.85E-06	2.63E-09	6.13E-06	2.95E-06
g__Phenylobacterium	5.06E-06	7.56E-10	3.29E-06	8.43E-07
g__Butyricimonas	5.48E-06	4.36E-10	2.50E-06	1.26E-05
g__Aeromonas	8.01E-06	1.11E-09	3.98E-06	2.53E-06
g__Fluviicola	0	0	0	2.95E-06
g__Chthoniobacter	2.95E-06	3.07E-10	2.09E-06	0
g__Eubacterium	0	0	0	2.95E-06
g__Chujaibacter	0	0	0	2.95E-06
g__Aurantimicrobium	0	0	0	2.95E-06
g__Caldalkalibacillus	0	0	0	2.95E-06
g__Caloramator	2.95E-06	2.56E-10	1.91E-06	0
g__Gardnerella	4.21E-06	4.36E-10	2.50E-06	1.05E-05
g__Methylobacterium-Methylorubrum	0.000440423	1.18E-05	0.000410316	5.06E-06
g__Anoxybacillus	0.000299235	9.89E-07	0.000118867	4.38E-05
g__[Eubacterium]_nodatum_group	0.000294599	2.21E-07	5.62E-05	0.000476247
g__Prevotellaceae_UCG-004	3.16E-05	1.08E-08	1.24E-05	8.01E-06
g__Allisonella	1.81E-05	4.07E-09	7.62E-06	9.69E-06
g__Eikenella	0.000958815	5.49E-06	0.000280051	0.000437473
g__Capnocytophaga	0.009470987	0.00027503	0.00198217	0.005125341
g__Lachnospiraceae_NK4A136_group	0.000177855	2.96E-07	6.51E-05	0.000864409

g__Harryflintia	3.79E-06	4.02E-10	2.40E-06	9.69E-06
g__Intestinimonas	1.26E-06	6.14E-11	9.37E-07	5.48E-06
g__Cryptobacterium	2.23E-05	7.02E-09	1.00E-05	1.31E-05
g__Pseudonocardia	4.21E-07	1.24E-11	4.21E-07	3.79E-06
g__Jeotgalicoccus	3.79E-06	2.25E-10	1.79E-06	4.21E-07
g__Desulfoplanes	4.21E-07	1.24E-11	4.21E-07	3.79E-06
g__Nocardioides	1.14E-05	1.50E-09	4.62E-06	5.06E-06
g__Parvimonas	0.000567281	3.42E-07	6.99E-05	0.000776324
g__Bosea	0	0	0	2.53E-06
g__Cellulomonas	2.53E-06	4.48E-10	2.53E-06	0
g__Brevibacillus	2.53E-06	1.70E-10	1.56E-06	0
g__Epulopiscium	2.53E-06	1.45E-10	1.44E-06	0
g__Coprobacillus	0	0	0	2.53E-06
g__Geobacter	0	0	0	2.53E-06
g__CHKCI002	2.53E-06	2.21E-10	1.78E-06	0
g__unidentified_Nitrospiraceae	0	0	0	2.53E-06
g__Mucilaginibacter	2.53E-06	4.48E-10	2.53E-06	0
g__C39	0	0	0	2.53E-06
g__Kineosporia	2.53E-06	3.21E-10	2.14E-06	0
g__Arthrobacter	0	0	0	2.53E-06
g__SEEP-SRB1	2.53E-06	3.21E-10	2.14E-06	0
g__Sphingomicrobium	0	0	0	2.53E-06
g__IheB3-7	2.53E-06	2.46E-10	1.87E-06	0
g__Planktotalea	2.53E-06	1.20E-10	1.31E-06	0
g__Filomicrobium	2.53E-06	1.70E-10	1.56E-06	0
g__Craurococcus-Caldovatus	0	0	0	2.53E-06
g__Allobaculum	5.90E-06	2.11E-09	5.49E-06	1.22E-05
g__Sporosarcina	7.80E-05	1.81E-07	5.08E-05	2.53E-06
g__Neisseria	0.091734802	0.008980845	0.011326861	0.125805826
g__Citrobacter	3.37E-06	1.40E-10	1.41E-06	4.21E-07
g__Proteus	6.32E-06	6.28E-10	3.00E-06	2.11E-06
g__Paracoccus	1.39E-05	1.53E-09	4.68E-06	7.59E-06
g__Acidaminococcus	5.48E-06	8.15E-10	3.41E-06	1.69E-06
g__Blastococcus	5.48E-06	1.07E-09	3.90E-06	1.69E-06
g__Holdemania	1.69E-06	7.28E-11	1.02E-06	5.48E-06
**CRC.Oral-N.Oral-Genus**				
g__Rhodanobacter	2.88E-05	2.99E-08	2.67E-05	0
g__Sorangium	2.74E-05	2.99E-08	2.67E-05	4.21E-07
g__Methylorosula	2.39E-05	2.26E-08	2.32E-05	0
g__Dysgonomonas	2.53E-05	2.26E-08	2.32E-05	4.21E-07
g__Candidatus_Koribacter	2.88E-05	3.48E-08	2.88E-05	1.26E-06
g__CAG-352	0	0	0	2.70E-05
g__Lacunisphaera	1.83E-05	1.40E-08	1.83E-05	0
g__Anaerobacillus	2.04E-05	1.62E-08	1.97E-05	8.43E-07

g__Pseudoclostridium	1.62E-05	1.00E-08	1.55E-05	0
g__Adlercreutzia	1.40E-06	4.04E-11	9.81E-07	2.57E-05
g__Sva0081_sediment_group	1.48E-05	1.79E-09	6.54E-06	0
g__Pseudoalteromonas	2.53E-05	1.65E-08	1.98E-05	3.37E-06
g__Gemmatimonas	1.90E-05	6.32E-09	1.23E-05	1.26E-06
g__Senegalimassilia	1.40E-06	4.04E-11	9.81E-07	2.19E-05
g__Acidothermus	1.69E-05	1.10E-08	1.62E-05	8.43E-07
g__Providencia	1.69E-05	1.00E-08	1.55E-05	8.43E-07
g__Dongia	1.33E-05	7.48E-09	1.33E-05	0
g__Desulfitibacter	1.33E-05	4.81E-09	1.07E-05	0
g__Eggerthella	4.21E-06	1.52E-10	1.90E-06	2.87E-05
g__Actinocorallia	1.26E-05	5.99E-09	1.19E-05	0
g__Lachnospiraceae_UCG-006	4.21E-06	2.79E-10	2.58E-06	2.70E-05
g__Catenibacterium	3.51E-06	2.63E-10	2.50E-06	2.49E-05
g__Brevibacterium	1.33E-05	7.48E-09	1.33E-05	4.21E-07
g__Tepidimonas	1.48E-05	5.36E-09	1.13E-05	8.43E-07
g__Agromyces	1.12E-05	2.59E-09	7.85E-06	0
g__Massilia	5.62E-06	3.07E-10	2.70E-06	2.87E-05
g__Eisenbergiella	0	0	0	1.43E-05
g__[Eubacterium]_xylanophilum_group	5.62E-06	3.92E-10	3.06E-06	2.82E-05
g__SWB02	1.26E-05	5.99E-09	1.19E-05	4.21E-07
g__Shewanella	1.40E-05	2.26E-09	7.34E-06	8.43E-07
g__Caldibacillus	1.76E-05	7.60E-09	1.35E-05	2.11E-06
g__Mitsuokella	7.02E-07	2.07E-11	7.02E-07	1.60E-05
g__Luteibacter	1.05E-05	4.66E-09	1.05E-05	0
g__Blastocatella	1.05E-05	4.66E-09	1.05E-05	0
g__Woeseia	1.48E-05	2.81E-09	8.18E-06	1.26E-06
g__Curvibacter	0	0	0	1.31E-05
g__A2	7.02E-07	2.07E-11	7.02E-07	1.56E-05
g__Zoogloea	1.19E-05	2.04E-09	6.97E-06	4.21E-07
g__Erysipelothrix	9.83E-06	1.85E-09	6.64E-06	0
g__Parapusillimonas	9.83E-06	4.06E-09	9.83E-06	0
g__Frisingicoccus	7.02E-06	1.18E-09	5.30E-06	2.82E-05
g__Flavitalea	9.13E-06	3.50E-09	9.13E-06	0
g__Brochothrix	1.83E-05	1.40E-08	1.83E-05	3.37E-06
g__Sarcina	8.43E-06	1.71E-09	6.38E-06	0
g__Alcaligenes	0	0	0	1.05E-05
g__Pelospora	4.21E-06	1.52E-10	1.90E-06	2.11E-05
g__Anaerotruncus	8.43E-06	7.34E-10	4.18E-06	2.87E-05
g__Peptoclostridium	0	0	0	1.01E-05
g__Anaeromicrobium	7.73E-06	7.67E-10	4.27E-06	0
g__Ohtaekwangia	9.13E-06	3.50E-09	9.13E-06	4.21E-07
g__Rahnella1	1.33E-05	7.48E-09	1.33E-05	2.11E-06

g__Caloramator	7.02E-06	1.05E-09	5.01E-06	0
g__SEEP-SRB1	7.02E-06	8.41E-10	4.47E-06	0
g__Sneathia	1.48E-05	9.14E-09	1.48E-05	2.95E-06
g__Rhodobacter	0	0	0	8.43E-06
g__Polynucleobacter	7.02E-07	2.07E-11	7.02E-07	1.05E-05
g__Schlegelella	6.32E-06	8.29E-10	4.44E-06	0
g__Cellulosilyticum	6.32E-06	8.29E-10	4.44E-06	0
g__Chthoniobacter	6.32E-06	1.34E-09	5.65E-06	0
g__Microbacterium	6.32E-06	1.04E-09	4.98E-06	0
g__Syntrophotalea	6.32E-06	7.02E-10	4.09E-06	0
g__Citrobacter	7.73E-06	1.23E-09	5.42E-06	4.21E-07
g__Coriobacteriaceae_UCG-002	0	0	0	8.01E-06
g__UCG-003	7.73E-06	5.12E-10	3.49E-06	2.36E-05
g__Adhaeribacter	5.62E-06	4.35E-10	3.22E-06	0
g__Jeotgalicoccus	7.02E-06	5.44E-10	3.60E-06	4.21E-07
g__[Clostridium]_innocuum_group	2.81E-06	1.19E-10	1.69E-06	1.39E-05
g__Thauera	0	0	0	7.16E-06
g__Candidatus_Stoquefichus	9.13E-06	1.46E-09	5.90E-06	2.49E-05
g__Proteus	1.05E-05	1.31E-09	5.58E-06	2.11E-06
g__Asteroleplasma	1.76E-05	2.72E-09	8.05E-06	5.90E-06
g__Lachnospiraceae_FCS020_group	4.21E-06	2.37E-10	2.37E-06	1.60E-05
g__Paenarthrobacter	6.32E-06	8.29E-10	4.44E-06	4.21E-07
g__Rhodoluna	0	0	0	6.74E-06
g__Herbaspirillum	0.038965126	0.03109845	0.027211014	2.95E-06
g__Aggregatibacter	0.006555767	4.31E-05	0.001012695	0.021785124
g__Acetoanaerobium	3.30E-05	2.07E-08	2.22E-05	0
g__Vagococcus	4.92E-05	5.75E-08	3.70E-05	0
g__Granulicella	6.11E-05	1.32E-07	5.61E-05	0
g__Peptococcus	0.000183334	3.92E-08	3.06E-05	0.000378469
g__[Eubacterium]_nodatum_group	0.000190358	4.24E-08	3.18E-05	0.000476247
g__Candidatus_Udaeobacter	6.32E-05	4.80E-08	3.38E-05	0
g__Parafilimonas	0.000137676	7.63E-07	0.000134816	0
g__Solobacterium	6.74E-05	7.57E-09	1.34E-05	0.000156361
g__Dechlorobacter	6.60E-05	6.49E-08	3.93E-05	0
g__Flavisolibacter	7.45E-05	1.76E-07	6.48E-05	0
g__FFCH7168	6.18E-05	1.50E-07	5.97E-05	0
g__Aliivibrio	4.28E-05	7.71E-08	4.28E-05	0
g__Psychroglaciecola	4.21E-05	6.50E-08	3.93E-05	0
g__Sporomusa	3.44E-05	4.38E-08	3.23E-05	0
g__Tepidibacter	6.88E-05	4.30E-08	3.20E-05	0
;g__Amnipila	1.76E-05	3.74E-09	9.43E-06	8.77E-05
g__Parvibaculum	4.92E-06	7.61E-10	4.26E-06	0
g__Streptosporangium	4.92E-06	1.02E-09	4.92E-06	0
g__Aridibacter	4.92E-06	7.61E-10	4.26E-06	0

g__Atopostipes	4.21E-06	1.94E-10	2.15E-06	1.52E-05
g__Thermus	1.12E-05	2.25E-09	7.32E-06	2.53E-06
g__Cetobacterium	0	0	0	5.90E-06
g__Erysipelotrichaceae_UCG-002	0	0	0	6.32E-06
g__Brevifollis	0	0	0	6.32E-06
g__Streptococcus	0.279039808	0.017509963	0.020418224	0.197415624
g__Lachnoanaerobaculum	0.000842212	6.74E-07	0.000126716	0.001297245
g__Paraclostridium	0.000262708	7.14E-07	0.000130399	1.26E-06
g__TM7	4.21E-06	7.46E-10	4.21E-06	0
g__Silvanigrella	4.21E-06	7.46E-10	4.21E-06	0
g__Candidatus_Soleaferrea	7.02E-07	2.07E-11	7.02E-07	7.59E-06
g__Brachymonas	7.02E-07	2.07E-11	7.02E-07	7.59E-06
g__Catonella	0.000457984	4.08E-07	9.86E-05	0.000849658
g__Blastomonas	0	0	0	3.29E-05
g__Shuttleworthia	3.30E-05	2.98E-09	8.42E-06	8.68E-05
g__Sellimonas	1.97E-05	2.58E-09	7.83E-06	8.43E-06
g__Ileibacterium	0	0	0	5.06E-06
g__Runella	0	0	0	5.06E-06
g__Neisseria	0.077510902	0.006825273	0.012747803	0.125805826
g__Ferruginibacter	3.51E-06	5.18E-10	3.51E-06	0
g__Sulfurovum	3.51E-06	5.18E-10	3.51E-06	0
g__Desulfatiglans	3.51E-06	1.78E-10	2.06E-06	0
g__Desulfatitalea	3.51E-06	2.21E-10	2.29E-06	0
g__Lachnospiraceae_NK3A20_group	9.13E-06	7.42E-10	4.20E-06	2.53E-06
g__Bradyrhizobium	1.19E-05	3.61E-09	9.27E-06	4.21E-06
g__Dialister	0.00093704	7.74E-07	0.000135732	0.002615142
g__Streptomyces	0	0	0	4.64E-06
g__Brevundimonas	6.32E-06	1.34E-09	5.65E-06	4.17E-05
g__Gemella	0.040336969	0.002027472	0.006947888	0.020282208
g__Fusobacterium	0.037870039	0.001051239	0.005002948	0.053858441
g__Ralstonia	1.62E-05	3.28E-09	8.83E-06	2.91E-05
g__Methylobacillus	4.21E-06	5.34E-10	3.56E-06	4.21E-07
g__Peptostreptococcus	0.000200192	9.92E-08	4.86E-05	0.000353181
g__Harryflintia	2.81E-06	3.32E-10	2.81E-06	9.69E-06
g__Delftia	0	0	0	3.79E-06
g__Halomonas	0	0	0	3.79E-06
g__Rikenella	0	0	0	3.79E-06
g__Nesterenkonia	0	0	0	3.79E-06
g__Desulfoplanes	0	0	0	3.79E-06
g__Prosthecobacter	0	0	0	3.79E-06
g__DTU089	7.73E-06	7.24E-10	4.15E-06	2.11E-06
g__Pedomicrobium	0	0	0	4.21E-06
g__Lachnospiraceae_NC2004_group	0	0	0	4.21E-06
g__Buchnera	0	0	0	4.21E-06

g__Kroppenstedtia	0	0	0	4.21E-06
g__Jannaschia	2.81E-06	1.19E-10	1.69E-06	0
g__Planococcus	2.81E-06	2.04E-10	2.20E-06	0
g__Renibacterium	2.81E-06	2.04E-10	2.20E-06	0
g__JG30a-KF-32	2.81E-06	3.32E-10	2.81E-06	0
g__Dietzia	2.81E-06	3.32E-10	2.81E-06	0
g__unidentified_Calditrichaceae	2.81E-06	1.19E-10	1.69E-06	0
g__Caldithrix	2.81E-06	1.19E-10	1.69E-06	0
g__Akkermansia	5.20E-05	7.49E-08	4.22E-05	0.000788968
g__Proteiniphilum	0.000155237	7.13E-07	0.00013033	2.95E-06
g__Sphingomonas	0.000778993	1.10E-05	0.000511226	3.03E-05
g__[Eubacterium]_saphenum_group	0.000205109	1.70E-07	6.36E-05	0.000467818
g__Intestinimonas	7.02E-07	2.07E-11	7.02E-07	5.48E-06
g__Cutibacterium	0.000325224	4.91E-07	0.000108098	6.87E-05
g__Stenotrophomonas	1.40E-06	4.04E-11	9.81E-07	6.74E-06
g__UCG-009	4.92E-06	1.66E-10	1.99E-06	1.26E-05
g__Pseudomonas	0.000369478	1.61E-06	0.000195731	2.99E-05
g__Listeria	0	0	0	3.37E-06
g__Komagataeibacter	0	0	0	3.37E-06
g__Porphyromonas	0.020541825	0.000587304	0.003739441	0.031575154
g__Rhodoferax	4.21E-06	7.46E-10	4.21E-06	1.14E-05
g__Methylobacterium-Methylorubrum	0.000874524	1.93E-05	0.000677067	5.06E-06
g__Vibrio	0.000330141	1.12E-06	0.000163644	6.32E-06
g__Marmoricola	4.92E-06	3.79E-10	3.00E-06	8.43E-07
g__Family_XIII_AD3011_group	1.55E-05	2.01E-09	6.91E-06	2.61E-05
g__Actinobacillus	0.009043067	0.000275636	0.00256179	0.017867258
g__Desulfomicrobium	2.46E-05	2.88E-09	8.29E-06	9.40E-05
g__Vulcaniibacterium	4.00E-05	2.12E-08	2.25E-05	4.21E-07
g__Exiguobacterium	0.000365263	4.48E-06	0.000326734	6.74E-06
g__Blautia	0.00044815	2.38E-06	0.000237866	0.002574682
g__Quadrisphaera	7.02E-07	2.07E-11	7.02E-07	5.06E-06
g__Clostridium_sensu_stricto_7	7.02E-07	2.07E-11	7.02E-07	5.06E-06
g__Peptoniphilus	7.02E-07	2.07E-11	7.02E-07	5.06E-06
g__Deinococcus	2.11E-06	1.02E-10	1.56E-06	3.03E-05
g__Butyricimonas	5.62E-06	2.65E-10	2.51E-06	1.26E-05
g__Acinetobacter	0.001487744	4.23E-05	0.001003574	0.000136131
g__Enterorhabdus	1.12E-05	1.65E-09	6.28E-06	3.33E-05
g__unidentified_Muribaculaceae	4.92E-06	1.02E-09	4.92E-06	1.26E-06
g__Erythrobacter	4.92E-06	3.36E-10	2.83E-06	1.26E-06

**variance. group2.**	**standard. error. group2.**	**p.value**	**q.value**	**FC**	**log10FC**
**GC.Oral-CRC.Oral-Genus**					
0	0	0.0000	0.0000	0.9733	-0.0117

6.14E-11	9.37E-07	0.0000	0.0000	0.9732	-0.0118
4.97E-11	8.43E-07	0.0000	0.0000	0.9775	-0.0099
0	0	0.0000	0.0000	0.9821	-0.0079
9.44E-11	1.16E-06	0.0000	0.0000	0.9758	-0.0106
1.34E-10	1.38E-06	0.0000	0.0000	0.9767	-0.0102
0	0	0.0000	0.0000	0.9834	-0.0073
5.09E-08	2.70E-05	0.0000	0.0000	1.0248	0.0106
1.71E-08	1.56E-05	0.0000	0.0000	1.0194	0.0083
1.12E-10	1.26E-06	0.0000	0.0000	0.9820	-0.0079
6.14E-11	9.37E-07	0.0000	0.0000	0.9833	-0.0073
0	0	0.0000	0.0000	0.9868	-0.0058
0	0	0.0000	0.0000	0.9875	-0.0055
1.70E-08	1.56E-05	0.0000	0.0000	1.0156	0.0067
1.23E-09	4.20E-06	0.0000	0.0000	0.9782	-0.0096
1.24E-11	4.21E-07	0.0000	0.0000	0.9872	-0.0056
0	0	0.0000	0.0000	0.9889	-0.0049
1.55E-10	1.49E-06	0.0000	0.0000	0.9829	-0.0075
3.17E-09	6.73E-06	0.0000	0.0000	1.0153	0.0066
0	0	0.0000	0.0000	0.9896	-0.0046
1.08E-08	1.24E-05	0.0000	0.0001	1.0159	0.0068
3.91E-09	7.47E-06	0.0000	0.0001	1.0172	0.0074
0	0	0.0000	0.0001	0.9903	-0.0042
0	0	0.0000	0.0002	0.9910	-0.0039
1.27E-08	1.35E-05	0.0000	0.0002	1.0132	0.0057
6.14E-11	9.37E-07	0.0000	0.0002	0.9881	-0.0052
1.24E-08	1.33E-05	0.0000	0.0002	1.0171	0.0074
2.11E-08	1.74E-05	0.0000	0.0002	1.0175	0.0075
2.45E-11	5.92E-07	0.0000	0.0002	0.9890	-0.0048
3.62E-11	7.19E-07	0.0000	0.0004	0.9888	-0.0049
6.59E-09	9.70E-06	0.0000	0.0005	1.0105	0.0046
5.54E-09	8.89E-06	0.0000	0.0009	1.0148	0.0064
1.24E-11	4.21E-07	0.0000	0.0011	0.9914	-0.0038
1.45E-10	1.44E-06	0.0000	0.0011	0.9880	-0.0053
3.37E-09	6.94E-06	0.0001	0.0024	1.0093	0.0040
0	0	0.0001	0.0056	0.9937	-0.0027
1.24E-11	4.21E-07	0.0002	0.0061	0.9928	-0.0032
0	0	0.0004	0.0142	0.9944	-0.0024
1.24E-11	4.21E-07	0.0004	0.0142	0.9934	-0.0029
7.28E-11	1.02E-06	0.0004	0.0144	0.9912	-0.0038
1.12E-10	1.26E-06	0.0005	0.0153	0.9922	-0.0034
1.53E-09	4.68E-06	0.0005	0.0164	1.0111	0.0048
2.81E-09	6.34E-06	0.0005	0.0176	1.0072	0.0031
1.28E-08	1.35E-05	0.0008	0.0252	1.0118	0.0051
5.52E-09	8.88E-06	0.0009	0.0252	1.0087	0.0038

1.24E-11	4.21E-07	0.0010	0.0252	0.9941	-0.0026
4.48E-06	0.000252937	0.0010	0.0252	1.5142	0.1802
1.90E-06	0.000164713	0.0010	0.0252	1.2631	0.1014
0	0	0.0010	0.0252	0.9481	-0.0232
0	0	0.0010	0.0252	0.9405	-0.0266
0	0	0.0010	0.0252	0.8790	-0.0560
0	0	0.0010	0.0252	0.9418	-0.0260
0	0	0.0010	0.0252	0.9707	-0.0129
0	0	0.0010	0.0252	0.9951	-0.0021
0	0	0.0010	0.0252	0.9951	-0.0021
0	0	0.0010	0.0252	0.9951	-0.0021
0	0	0.0010	0.0252	0.9951	-0.0021
0	0	0.0010	0.0252	0.9951	-0.0021
3.32E-10	2.18E-06	0.0011	0.0272	0.9911	-0.0039
2.80E-09	6.32E-06	0.0019	0.0424	1.0063	0.0027
3.82E-09	7.39E-06	0.0019	0.0424	1.0094	0.0041
4.83E-06	0.000262713	0.0020	0.0445	1.2966	0.1128
3.78E-10	2.32E-06	0.0020	0.0448	0.9898	-0.0045
0	0	0.0028	0.0566	0.9958	-0.0018
0	0	0.0028	0.0566	0.9958	-0.0018
0	0	0.0028	0.0566	0.9958	-0.0018
0	0	0.0028	0.0566	0.9958	-0.0018
0	0	0.0028	0.0566	0.9958	-0.0018
2.10E-09	5.48E-06	0.0029	0.0585	1.0055	0.0024
1.81E-07	5.08E-05	0.0030	0.0593	1.0772	0.0323
1.24E-09	4.21E-06	0.0032	0.0624	0.9910	-0.0039
4.97E-11	8.43E-07	0.0035	0.0683	0.9946	-0.0024
4.07E-09	7.62E-06	0.0043	0.0820	1.0110	0.0048
1.08E-08	1.24E-05	0.0050	0.0936	1.0280	0.0120
7.56E-10	3.29E-06	0.0051	0.0937	1.0051	0.0022
7.31E-10	3.23E-06	0.0051	0.0937	1.0051	0.0022
6.96E-09	9.97E-06	0.0053	0.0960	1.0099	0.0043
7.71E-09	1.05E-05	0.0061	0.1095	1.0141	0.0061
2.22E-09	5.64E-06	0.0066	0.1158	1.0070	0.0030
0.002672317	0.006178669	0.0070	0.1200	1.9434	0.2886
5.83E-06	0.000288471	0.0070	0.1200	1.3258	0.1225
0	0	0.0074	0.1242	0.9965	-0.0015
0	0	0.0074	0.1242	0.9965	-0.0015
3.18E-09	6.74E-06	0.0075	0.1249	0.9907	-0.0040
5.67E-09	9.00E-06	0.0092	0.1495	1.0084	0.0036
1.50E-09	4.62E-06	0.0099	0.1590	1.0078	0.0034
1.12E-10	1.26E-06	0.0130	0.2058	0.9950	-0.0022
1.24E-11	4.21E-07	0.0132	0.2067	0.9962	-0.0016
1.01E-09	3.79E-06	0.0169	0.2539	1.0038	0.0016

1.01E-09	3.79E-06	0.0169	0.2539	1.0038	0.0016
1.01E-09	3.79E-06	0.0169	0.2539	1.0038	0.0016
1.02E-09	3.81E-06	0.0169	0.2539	1.0042	0.0018
0	0	0.0198	0.2855	0.9972	-0.0012
0	0	0.0198	0.2855	0.9972	-0.0012
3.77E-09	7.34E-06	0.0200	0.2855	1.0091	0.0039
6.89E-10	3.14E-06	0.0236	0.3324	1.0048	0.0021
1.40E-06	0.000141346	0.0240	0.3347	1.2829	0.1082
0.000527185	0.002744307	0.0280	0.3809	4.7221	0.6741
3.51E-09	7.08E-06	0.0286	0.3809	1.0067	0.0029
7.96E-10	3.37E-06	0.0289	0.3809	1.0034	0.0015
7.96E-10	3.37E-06	0.0289	0.3809	1.0034	0.0015
3.67E-10	2.29E-06	0.0289	0.3809	1.0034	0.0015
3.65E-08	2.28E-05	0.0310	0.4046	1.0545	0.0231
2.45E-11	5.92E-07	0.0316	0.4086	0.9959	-0.0018
7.37E-09	1.03E-05	0.0330	0.4229	1.0280	0.0120
3.92E-10	2.37E-06	0.0339	0.4315	0.9919	-0.0036
7.02E-09	1.00E-05	0.0369	0.4652	1.0096	0.0041
1.05E-09	3.87E-06	0.0386	0.4820	1.0039	0.0017
1.63E-09	4.83E-06	0.0396	0.4902	1.0055	0.0024
**GC.Oral-N.Oral-Genus**					
0	0	0.0000	0.0000	0.9737	-0.0116
2.33E-08	1.82E-05	0.0000	0.0000	1.0265	0.0114
6.14E-11	9.37E-07	0.0000	0.0000	0.9770	-0.0101
0	0	0.0000	0.0000	0.9810	-0.0083
0	0	0.0000	0.0000	0.9830	-0.0074
0	0	0.0000	0.0000	0.9834	-0.0073
6.14E-11	9.37E-07	0.0000	0.0000	0.9806	-0.0085
4.33E-08	2.49E-05	0.0000	0.0000	1.0227	0.0097
2.28E-08	1.81E-05	0.0000	0.0000	1.0243	0.0104
0	0	0.0000	0.0000	0.9846	-0.0067
2.35E-08	1.83E-05	0.0000	0.0000	1.0239	0.0103
1.70E-08	1.56E-05	0.0000	0.0000	1.0198	0.0085
2.82E-08	2.01E-05	0.0000	0.0000	1.0214	0.0092
2.65E-09	6.15E-06	0.0000	0.0000	1.0177	0.0076
5.86E-11	9.15E-07	0.0000	0.0000	0.9814	-0.0081
6.05E-09	9.29E-06	0.0000	0.0000	1.0131	0.0056
4.97E-11	8.43E-07	0.0000	0.0000	0.9855	-0.0064
7.71E-09	1.05E-05	0.0000	0.0000	1.0218	0.0094
3.62E-11	7.19E-07	0.0000	0.0000	0.9855	-0.0064
1.57E-09	4.74E-06	0.0000	0.0000	1.0114	0.0049
1.45E-10	1.44E-06	0.0000	0.0000	0.9843	-0.0069
3.84E-09	7.40E-06	0.0000	0.0000	1.0105	0.0046
1.34E-10	1.38E-06	0.0000	0.0000	0.9855	-0.0063

3.93E-09	7.50E-06	0.0000	0.0000	1.0101	0.0044
5.82E-09	9.12E-06	0.0000	0.0000	1.0139	0.0060
3.35E-10	2.19E-06	0.0000	0.0000	0.9839	-0.0071
3.30E-09	6.86E-06	0.0000	0.0000	1.0135	0.0058
0	0	0.0000	0.0000	0.9908	-0.0040
8.19E-09	1.08E-05	0.0000	0.0001	1.0122	0.0053
2.85E-09	6.39E-06	0.0000	0.0001	1.0084	0.0036
4.63E-09	8.14E-06	0.0000	0.0001	1.0171	0.0074
1.49E-09	4.61E-06	0.0000	0.0002	1.0097	0.0042
2.45E-11	5.92E-07	0.0000	0.0002	0.9904	-0.0042
7.61E-10	3.30E-06	0.0000	0.0004	0.9847	-0.0067
2.45E-11	5.92E-07	0.0000	0.0004	0.9908	-0.0040
8.18E-10	3.42E-06	0.0000	0.0006	1.0072	0.0031
7.79E-09	1.06E-05	0.0000	0.0006	1.0175	0.0075
1.73E-08	1.57E-05	0.0000	0.0008	1.0155	0.0067
0	0	0.0000	0.0010	0.9933	-0.0029
0	0	0.0000	0.0010	0.9933	-0.0029
1.57E-09	4.73E-06	0.0000	0.0010	1.0067	0.0029
3.18E-09	6.74E-06	0.0000	0.0011	1.0142	0.0061
2.45E-11	5.92E-07	0.0000	0.0012	0.9916	-0.0036
2.10E-10	1.73E-06	0.0000	0.0012	0.9900	-0.0044
2.10E-08	1.73E-05	0.0001	0.0016	1.0159	0.0068
0	0	0.0001	0.0018	0.9937	-0.0027
9.31E-10	3.65E-06	0.0001	0.0018	1.0063	0.0027
1.44E-09	4.53E-06	0.0001	0.0018	1.0063	0.0027
5.67E-10	2.85E-06	0.0001	0.0019	0.9836	-0.0072
8.27E-09	1.09E-05	0.0001	0.0021	1.0158	0.0068
2.45E-11	5.92E-07	0.0001	0.0032	0.9925	-0.0033
5.20E-10	2.72E-06	0.0001	0.0032	1.0059	0.0026
0	0	0.0001	0.0032	0.9941	-0.0026
6.46E-10	3.04E-06	0.0001	0.0032	1.0059	0.0026
2.29E-08	1.81E-05	0.0002	0.0040	1.0158	0.0068
4.31E-09	7.84E-06	0.0002	0.0043	1.0105	0.0045
6.46E-10	3.04E-06	0.0002	0.0058	0.9884	-0.0051
6.14E-11	9.37E-07	0.0003	0.0067	0.9925	-0.0033
7.56E-10	3.29E-06	0.0003	0.0073	0.9892	-0.0047
1.58E-08	1.50E-05	0.0005	0.0102	1.0150	0.0065
2.66E-10	1.95E-06	0.0005	0.0102	0.9908	-0.0040
0	0	0.0005	0.0102	0.9950	-0.0022
7.31E-10	3.23E-06	0.0005	0.0102	1.0051	0.0022
9.07E-10	3.60E-06	0.0005	0.0102	1.0051	0.0022
0	0	0.0005	0.0102	0.9950	-0.0022
0	0	0.0005	0.0102	0.9950	-0.0022
8.82E-10	3.55E-06	0.0005	0.0102	1.0051	0.0022

1.24E-11	4.21E-07	0.0005	0.0107	0.9941	-0.0026
9.81E-09	1.18E-05	0.0006	0.0126	1.0105	0.0045
2.25E-09	5.67E-06	0.0006	0.0128	0.9880	-0.0052
6.47E-10	3.04E-06	0.0010	0.0151	1.0046	0.0020
6.22E-10	2.98E-06	0.0010	0.0151	1.0046	0.0020
4.20E-10	2.45E-06	0.0010	0.0151	1.0046	0.0020
2.56E-10	1.91E-06	0.0010	0.0151	0.0676	-1.1704
0.014031651	0.014158113	0.0010	0.0151	1.8408	0.2650
0.000469363	0.002589436	0.0010	0.0151	0.5055	-0.2962
7.00E-06	0.000316203	0.0010	0.0151	0.3449	-0.4623
0	0	0.0010	0.0151	0.9545	-0.0202
0.000302368	0.00207835	0.0010	0.0151	2.3133	0.3642
1.86E-07	5.15E-05	0.0010	0.0151	1.1771	0.0708
6.80E-06	0.000311579	0.0010	0.0151	1.4392	0.1581
0	0	0.0010	0.0151	0.7495	-0.1252
1.00E-08	1.20E-05	0.0010	0.0151	1.0269	0.0115
0	0	0.0010	0.0151	0.8074	-0.0929
0	0	0.0010	0.0151	0.8306	-0.0806
1.24E-11	4.21E-07	0.0010	0.0151	0.7517	-0.1239
3.04E-08	2.08E-05	0.0010	0.0151	1.0295	0.0126
0	0	0.0010	0.0151	0.9447	-0.0247
0	0	0.0010	0.0151	0.9469	-0.0237
5.63E-09	8.96E-06	0.0010	0.0151	1.0333	0.0142
0	0	0.0010	0.0151	0.9557	-0.0197
2.40E-08	1.85E-05	0.0010	0.0151	1.0732	0.0307
0	0	0.0020	0.0276	0.9958	-0.0018
5.37E-10	2.77E-06	0.0020	0.0276	1.0042	0.0018
7.14E-10	3.19E-06	0.0020	0.0276	1.0042	0.0018
2.85E-10	2.02E-06	0.0020	0.0276	1.0042	0.0018
0	0	0.0020	0.0276	0.9958	-0.0018
0	0	0.0020	0.0276	0.9958	-0.0018
1.99E-10	1.69E-06	0.0020	0.0276	0.7720	-0.1124
1.16E-08	1.29E-05	0.0020	0.0276	0.7366	-0.1327
9.77E-10	3.74E-06	0.0020	0.0276	0.9510	-0.0218
1.06E-08	1.23E-05	0.0026	0.0356	1.0092	0.0040
1.80E-10	1.61E-06	0.0030	0.0403	0.6912	-0.1604
6.52E-08	3.05E-05	0.0030	0.0403	1.0926	0.0384
5.29E-10	2.75E-06	0.0034	0.0455	1.0046	0.0020
3.26E-10	2.16E-06	0.0039	0.0501	1.0038	0.0016
0	0	0.0039	0.0501	0.9962	-0.0016
5.03E-10	2.68E-06	0.0039	0.0501	1.0038	0.0016
0	0	0.0039	0.0501	0.9962	-0.0016
0.008201321	0.010824127	0.0040	0.0504	0.7719	-0.1124
8.80E-10	3.55E-06	0.0040	0.0504	0.6782	-0.1686

4.76E-11	8.24E-07	0.0043	0.0533	0.9941	-0.0026
1.13E-09	4.01E-06	0.0043	0.0533	1.0059	0.0026
4.46E-10	2.52E-06	0.0043	0.0533	1.0059	0.0026
1.26E-09	4.24E-06	0.0049	0.0597	1.0100	0.0043
0.000158999	0.001507122	0.0060	0.0711	0.5258	-0.2792
9.02E-09	1.14E-05	0.0060	0.0711	1.0291	0.0125
1.12E-10	1.26E-06	0.0060	0.0711	0.7968	-0.0987
1.24E-11	4.21E-07	0.0063	0.0747	0.9958	-0.0018
0	0	0.0078	0.0869	0.9966	-0.0015
7.96E-10	3.37E-06	0.0078	0.0869	1.0034	0.0015
1.90E-10	1.65E-06	0.0078	0.0869	1.0034	0.0015
0	0	0.0078	0.0869	0.9966	-0.0015
0	0	0.0078	0.0869	0.9966	-0.0015
3.92E-10	2.37E-06	0.0078	0.0869	1.0034	0.0015
0	0	0.0078	0.0869	0.9966	-0.0015
6.68E-09	9.77E-06	0.0079	0.0877	1.0080	0.0034
4.17E-10	2.44E-06	0.0081	0.0891	1.0063	0.0027
1.47E-07	4.58E-05	0.0090	0.0970	0.7996	-0.0971
2.96E-09	6.50E-06	0.0090	0.0970	1.0201	0.0086
1.79E-09	5.06E-06	0.0115	0.1232	0.9929	-0.0031
5.12E-10	2.70E-06	0.0117	0.1236	1.0038	0.0016
3.10E-10	2.10E-06	0.0117	0.1236	1.0038	0.0016
3.95E-09	7.51E-06	0.0120	0.1255	0.9444	-0.0248
5.51E-09	8.87E-06	0.0125	0.1294	1.0088	0.0038
2.56E-10	1.91E-06	0.0125	0.1294	0.9942	-0.0025
2.45E-11	5.92E-07	0.0129	0.1326	0.9958	-0.0018
3.75E-09	7.32E-06	0.0137	0.1395	1.0071	0.0031
1.45E-10	1.44E-06	0.0146	0.1478	0.9946	-0.0024
4.58E-10	2.56E-06	0.0156	0.1503	1.0030	0.0013
0	0	0.0156	0.1503	0.9971	-0.0013
4.58E-10	2.56E-06	0.0156	0.1503	1.0030	0.0013
3.57E-10	2.26E-06	0.0156	0.1503	1.0030	0.0013
3.07E-10	2.09E-06	0.0156	0.1503	1.0030	0.0013
1.80E-10	1.61E-06	0.0156	0.1503	1.0030	0.0013
0	0	0.0156	0.1503	0.9971	-0.0013
2.32E-09	5.76E-06	0.0167	0.1583	1.0063	0.0027
4.03E-10	2.40E-06	0.0170	0.1591	0.6978	-0.1563
5.88E-08	2.90E-05	0.0170	0.1591	0.8034	-0.0951
1.72E-07	4.95E-05	0.0180	0.1663	1.1403	0.0570
3.01E-10	2.07E-06	0.0180	0.1663	0.9771	-0.0101
1.30E-09	4.32E-06	0.0187	0.1714	0.9917	-0.0036
1.84E-07	5.13E-05	0.0190	0.1733	0.7338	-0.1344
2.20E-05	0.000560497	0.0200	0.1797	0.5850	-0.2329
8.86E-06	0.000355681	0.0200	0.1797	1.5829	0.1994

1.15E-09	4.06E-06	0.0201	0.1797	1.0059	0.0025
3.35E-10	2.19E-06	0.0213	0.1843	1.0042	0.0018
7.22E-10	3.21E-06	0.0214	0.1843	0.9909	-0.0040
2.25E-10	1.79E-06	0.0215	0.1843	1.0034	0.0015
1.24E-11	4.21E-07	0.0215	0.1843	0.9966	-0.0015
6.54E-10	3.06E-06	0.0215	0.1843	1.0034	0.0015
8.07E-10	3.39E-06	0.0237	0.2021	0.9937	-0.0027
3.15E-07	6.71E-05	0.0290	0.2392	1.1334	0.0544
3.21E-10	2.14E-06	0.0312	0.2392	1.0025	0.0011
0	0	0.0312	0.2392	0.9975	-0.0011
0	0	0.0312	0.2392	0.9975	-0.0011
0	0	0.0312	0.2392	0.9975	-0.0011
3.21E-10	2.14E-06	0.0312	0.2392	1.0025	0.0011
1.45E-10	1.44E-06	0.0312	0.2392	1.0025	0.0011
0	0	0.0312	0.2392	0.9975	-0.0011
2.21E-10	1.78E-06	0.0312	0.2392	1.0025	0.0011
0	0	0.0312	0.2392	0.9975	-0.0011
1.70E-10	1.56E-06	0.0312	0.2392	1.0025	0.0011
0	0	0.0312	0.2392	0.9975	-0.0011
4.48E-10	2.53E-06	0.0312	0.2392	1.0025	0.0011
0	0	0.0312	0.2392	0.9975	-0.0011
3.21E-10	2.14E-06	0.0312	0.2392	1.0025	0.0011
0	0	0.0312	0.2392	0.9975	-0.0011
0	0	0.0312	0.2392	0.9975	-0.0011
0	0	0.0312	0.2392	0.9975	-0.0011
1.70E-10	1.56E-06	0.0312	0.2392	1.0025	0.0011
3.65E-09	7.22E-06	0.0315	0.2402	1.0063	0.0027
2.21E-10	1.78E-06	0.0380	0.2875	0.9300	-0.0315
0.007853606	0.010592184	0.0390	0.2928	1.3674	0.1359
1.24E-11	4.21E-07	0.0391	0.2928	0.9971	-0.0013
8.38E-11	1.09E-06	0.0414	0.3086	0.9958	-0.0018
1.10E-09	3.97E-06	0.0489	0.3581	0.9938	-0.0027
1.23E-10	1.33E-06	0.0490	0.3581	0.9962	-0.0016
7.28E-11	1.02E-06	0.0490	0.3581	0.9962	-0.0016
6.13E-10	2.96E-06	0.0490	0.3581	1.0038	0.0016
**CRC.Oral-N.Oral-Genus**					
0	0	0.0000	0.0000	0.9720	-0.0123
1.24E-11	4.21E-07	0.0000	0.0000	0.9737	-0.0116
0	0	0.0000	0.0000	0.9767	-0.0103
1.24E-11	4.21E-07	0.0000	0.0000	0.9757	-0.0107
3.62E-11	7.19E-07	0.0000	0.0000	0.9732	-0.0118
2.33E-08	1.82E-05	0.0000	0.0000	1.0270	0.0116
0	0	0.0000	0.0000	0.9821	-0.0079
4.97E-11	8.43E-07	0.0000	0.0000	0.9809	-0.0084

0	0	0.0000	0.0000	0.9841	-0.0070
4.33E-08	2.49E-05	0.0000	0.0000	1.0243	0.0104
0	0	0.0000	0.0000	0.9855	-0.0064
4.17E-10	2.44E-06	0.0000	0.0000	0.9786	-0.0094
6.14E-11	9.37E-07	0.0000	0.0000	0.9826	-0.0076
1.70E-08	1.56E-05	0.0000	0.0000	1.0205	0.0088
4.97E-11	8.43E-07	0.0000	0.0000	0.9843	-0.0069
4.97E-11	8.43E-07	0.0000	0.0000	0.9843	-0.0069
0	0	0.0000	0.0000	0.9868	-0.0058
0	0	0.0000	0.0000	0.9868	-0.0058
2.29E-08	1.81E-05	0.0000	0.0000	1.0243	0.0104
0	0	0.0000	0.0000	0.9875	-0.0055
8.27E-09	1.09E-05	0.0000	0.0000	1.0227	0.0097
2.82E-08	2.01E-05	0.0000	0.0000	1.0213	0.0091
1.24E-11	4.21E-07	0.0000	0.0000	0.9872	-0.0056
2.45E-11	5.92E-07	0.0000	0.0000	0.9863	-0.0060
0	0	0.0000	0.0000	0.9889	-0.0049
2.28E-08	1.81E-05	0.0000	0.0000	1.0229	0.0098
4.31E-09	7.84E-06	0.0000	0.0000	1.0143	0.0062
2.35E-08	1.83E-05	0.0000	0.0000	1.0225	0.0097
1.24E-11	4.21E-07	0.0000	0.0000	0.9879	-0.0053
2.45E-11	5.92E-07	0.0000	0.0000	0.9870	-0.0057
2.10E-10	1.73E-06	0.0000	0.0000	0.9848	-0.0066
9.81E-09	1.18E-05	0.0000	0.0000	1.0153	0.0066
0	0	0.0000	0.0000	0.9896	-0.0046
0	0	0.0000	0.0000	0.9896	-0.0046
6.14E-11	9.37E-07	0.0000	0.0000	0.9867	-0.0058
6.05E-09	9.29E-06	0.0000	0.0000	1.0131	0.0056
3.30E-09	6.86E-06	0.0000	0.0000	1.0149	0.0064
1.24E-11	4.21E-07	0.0000	0.0000	0.9886	-0.0050
0	0	0.0000	0.0000	0.9903	-0.0042
0	0	0.0000	0.0000	0.9903	-0.0042
7.71E-09	1.05E-05	0.0000	0.0001	1.0211	0.0091
0	0	0.0000	0.0001	0.9910	-0.0039
2.41E-10	1.85E-06	0.0000	0.0002	0.9854	-0.0064
0	0	0.0000	0.0002	0.9916	-0.0036
1.49E-09	4.61E-06	0.0000	0.0003	1.0105	0.0046
3.18E-09	6.74E-06	0.0000	0.0003	1.0168	0.0072
7.79E-09	1.06E-05	0.0000	0.0004	1.0201	0.0086
3.93E-09	7.50E-06	0.0000	0.0005	1.0101	0.0044
0	0	0.0000	0.0006	0.9923	-0.0033
1.24E-11	4.21E-07	0.0000	0.0007	0.9914	-0.0038
1.09E-10	1.25E-06	0.0001	0.0014	0.9889	-0.0048
0	0	0.0001	0.0014	0.9930	-0.0030

0	0	0.0001	0.0014	0.9930	-0.0030
2.56E-10	1.91E-06	0.0001	0.0024	0.9884	-0.0051
2.85E-09	6.39E-06	0.0001	0.0025	1.0084	0.0036
3.84E-09	7.40E-06	0.0001	0.0032	1.0098	0.0042
0	0	0.0001	0.0033	0.9937	-0.0027
0	0	0.0001	0.0033	0.9937	-0.0027
0	0	0.0001	0.0033	0.9937	-0.0027
0	0	0.0001	0.0033	0.9937	-0.0027
0	0	0.0001	0.0033	0.9937	-0.0027
1.24E-11	4.21E-07	0.0002	0.0036	0.9928	-0.0032
4.03E-09	7.59E-06	0.0002	0.0037	1.0080	0.0035
1.73E-08	1.57E-05	0.0002	0.0045	1.0158	0.0068
0	0	0.0004	0.0082	0.9944	-0.0024
1.24E-11	4.21E-07	0.0004	0.0082	0.9934	-0.0029
8.19E-09	1.08E-05	0.0005	0.0101	1.0111	0.0048
8.18E-10	3.42E-06	0.0005	0.0109	1.0072	0.0031
4.63E-09	8.14E-06	0.0006	0.0109	1.0156	0.0067
8.38E-11	1.09E-06	0.0008	0.0150	0.9917	-0.0036
3.43E-10	2.21E-06	0.0008	0.0150	0.9885	-0.0050
5.82E-09	9.12E-06	0.0008	0.0150	1.0118	0.0051
1.24E-11	4.21E-07	0.0010	0.0150	0.9941	-0.0026
1.57E-09	4.73E-06	0.0010	0.0150	1.0067	0.0029
2.56E-10	1.91E-06	0.0010	0.0150	0.0251	-1.6004
0.000302368	0.00207835	0.0010	0.0150	3.0156	0.4794
0	0	0.0010	0.0150	0.9680	-0.0141
0	0	0.0010	0.0150	0.9531	-0.0208
0	0	0.0010	0.0150	0.9424	-0.0258
9.82E-08	3.75E-05	0.0010	0.0150	1.1649	0.0663
1.72E-07	4.95E-05	0.0010	0.0150	1.2402	0.0935
0	0	0.0010	0.0150	0.9405	-0.0266
0	0	0.0010	0.0150	0.8790	-0.0560
1.10E-08	1.25E-05	0.0010	0.0150	1.0833	0.0348
0	0	0.0010	0.0150	0.9381	-0.0278
0	0	0.0010	0.0150	0.9307	-0.0312
0	0	0.0010	0.0150	0.9418	-0.0260
0	0	0.0010	0.0150	0.9589	-0.0182
0	0	0.0010	0.0150	0.9596	-0.0179
0	0	0.0010	0.0150	0.9667	-0.0147
0	0	0.0010	0.0150	0.9356	-0.0289
2.40E-08	1.85E-05	0.0010	0.0150	1.0689	0.0289
0	0	0.0010	0.0152	0.9951	-0.0021
0	0	0.0010	0.0152	0.9951	-0.0021
0	0	0.0010	0.0152	0.9951	-0.0021
1.06E-08	1.23E-05	0.0013	0.0182	1.0109	0.0047

1.45E-10	1.44E-06	0.0013	0.0187	0.9914	-0.0038
5.20E-10	2.72E-06	0.0017	0.0238	1.0059	0.0026
9.31E-10	3.65E-06	0.0019	0.0257	1.0063	0.0027
1.44E-09	4.53E-06	0.0019	0.0257	1.0063	0.0027
0.008201321	0.010824127	0.0020	0.0268	0.7085	-0.1496
6.42E-07	9.58E-05	0.0020	0.0268	1.2470	0.0959
1.12E-10	1.26E-06	0.0020	0.0268	0.7929	-0.1008
0	0	0.0028	0.0367	0.9958	-0.0018
0	0	0.0028	0.0367	0.9958	-0.0018
1.13E-09	4.01E-06	0.0031	0.0405	1.0069	0.0030
4.46E-10	2.52E-06	0.0031	0.0405	1.0069	0.0030
5.27E-07	8.68E-05	0.0040	0.0504	1.2686	0.1033
3.03E-08	2.08E-05	0.0040	0.0504	1.0329	0.0140
2.14E-08	1.75E-05	0.0040	0.0504	1.0521	0.0221
2.25E-09	5.67E-06	0.0042	0.0519	0.9890	-0.0048
1.06E-09	3.89E-06	0.0051	0.0630	1.0051	0.0022
8.82E-10	3.55E-06	0.0051	0.0630	1.0051	0.0022
0.007853606	0.010592184	0.0060	0.0730	1.6151	0.2082
0	0	0.0074	0.0873	0.9965	-0.0015
0	0	0.0074	0.0873	0.9965	-0.0015
0	0	0.0074	0.0873	0.9965	-0.0015
0	0	0.0074	0.0873	0.9965	-0.0015
1.70E-10	1.56E-06	0.0078	0.0913	0.9935	-0.0029
4.11E-10	2.42E-06	0.0088	0.1018	0.9924	-0.0033
7.16E-05	0.001011518	0.0090	0.1033	1.8663	0.2710
6.72E-10	3.10E-06	0.0092	0.1052	1.0046	0.0020
9.02E-09	1.14E-05	0.0110	0.1242	1.0352	0.0150
0.000469363	0.002589436	0.0120	0.1334	0.5148	-0.2883
0.001009362	0.003797297	0.0130	0.1421	1.4113	0.1496
1.58E-08	1.50E-05	0.0131	0.1421	1.0127	0.0055
1.24E-11	4.21E-07	0.0132	0.1425	0.9962	-0.0016
1.28E-07	4.28E-05	0.0150	0.1604	1.1275	0.0521
1.15E-09	4.06E-06	0.0158	0.1652	1.0069	0.0030
3.51E-10	2.24E-06	0.0169	0.1652	1.0038	0.0016
3.26E-10	2.16E-06	0.0169	0.1652	1.0038	0.0016
1.01E-09	3.79E-06	0.0169	0.1652	1.0038	0.0016
3.26E-10	2.16E-06	0.0169	0.1652	1.0038	0.0016
6.54E-10	3.06E-06	0.0169	0.1652	1.0038	0.0016
5.03E-10	2.68E-06	0.0169	0.1652	1.0038	0.0016
1.34E-10	1.38E-06	0.0169	0.1652	0.9944	-0.0024
3.35E-10	2.19E-06	0.0169	0.1652	1.0042	0.0018
7.14E-10	3.19E-06	0.0169	0.1652	1.0042	0.0018
2.85E-10	2.02E-06	0.0169	0.1652	1.0042	0.0018
3.10E-10	2.10E-06	0.0169	0.1652	1.0042	0.0018

0	0	0.0198	0.1812	0.9972	-0.0012
0	0	0.0198	0.1812	0.9972	-0.0012
0	0	0.0198	0.1812	0.9972	-0.0012
0	0	0.0198	0.1812	0.9972	-0.0012
0	0	0.0198	0.1812	0.9972	-0.0012
0	0	0.0198	0.1812	0.9972	-0.0012
0	0	0.0198	0.1812	0.9972	-0.0012
6.66E-06	0.000308369	0.0200	0.1812	1.7006	0.2306
1.80E-10	1.61E-06	0.0200	0.1812	0.8682	-0.0614
1.16E-08	1.29E-05	0.0210	0.1879	0.5792	-0.2372
7.00E-07	0.000100019	0.0210	0.1879	1.2180	0.0856
3.35E-10	2.19E-06	0.0236	0.2099	1.0048	0.0021
6.83E-08	3.12E-05	0.0240	0.2121	0.8064	-0.0934
5.34E-10	2.76E-06	0.0257	0.2250	1.0053	0.0023
4.83E-09	8.31E-06	0.0258	0.2250	1.0077	0.0033
5.59E-09	8.93E-06	0.0270	0.2342	0.7521	-0.1237
7.96E-10	3.37E-06	0.0289	0.2470	1.0034	0.0015
3.92E-10	2.37E-06	0.0289	0.2470	1.0034	0.0015
0.000875614	0.003536775	0.0290	0.2470	1.5122	0.1796
1.57E-09	4.74E-06	0.0292	0.2475	1.0071	0.0031
4.03E-10	2.40E-06	0.0300	0.2525	0.5362	-0.2707
8.80E-10	3.55E-06	0.0310	0.2594	0.7566	-0.1212
4.97E-11	8.43E-07	0.0316	0.2628	0.9959	-0.0018
2.10E-08	1.73E-05	0.0324	0.2682	1.0105	0.0045
0.000677653	0.003111392	0.0350	0.2862	1.8786	0.2738
1.04E-07	3.85E-05	0.0350	0.2862	1.0677	0.0285
1.24E-11	4.21E-07	0.0370	0.3008	0.9619	-0.0169
7.61E-10	3.30E-06	0.0390	0.3152	0.7374	-0.1323
8.90E-05	0.001127761	0.0400	0.3170	2.4684	0.3924
7.56E-10	3.29E-06	0.0401	0.3170	1.0044	0.0019
9.07E-10	3.60E-06	0.0401	0.3170	1.0044	0.0019
5.29E-10	2.75E-06	0.0401	0.3170	1.0044	0.0019
1.89E-08	1.64E-05	0.0410	0.3222	1.0282	0.0121
3.75E-09	7.32E-06	0.0432	0.3382	1.0070	0.0030
6.51E-08	3.05E-05	0.0470	0.3634	0.4567	-0.3404
5.25E-09	8.66E-06	0.0470	0.3634	1.0218	0.0094
3.62E-11	7.19E-07	0.0475	0.3636	0.9964	-0.0016
3.62E-11	7.19E-07	0.0475	0.3636	0.9964	-0.0016
