# Supplementary material for: Combined Non-Invasive Prediction and New Biomarkers of Oral and Fecal Microbiota in Patients With Gastric and Colorectal Cancer
Source: Front Cell Infect Microbiol. 2022 May 19;12:830684. doi: 10.3389/fcimb.2022.830684 (PMC9161364; doi:10.3389/fcimb.2022.830684)
Supplement: Supplementary file 1 [file DataSheet_1.zip › Supplementary Table 4.pdf]

Table S4. Metastases analysis of phylum and genus levels of fecal samples of HC, GC and CRC

Phylum	mean. group1.	variance. group1.	standard. error. group1.	mean. group2.
**GC.Stool-CRC.Stool-Phylum**				
p__Bdellovibrionota	0	0	0	2.83E-05
p__Entotheonellaeota	0	0	0	1.69E-05
p__Armatimonadota	0	0	0	1.63E-05
p__Elusimicrobia	3.58E-06	2.59E-10	2.80E-06	1.87E-05
p__Acidobacteriota	0	0	0	0.000377505
p__Gemmatimonadetes	0	0	0	3.43E-05
p__WPS-2	0	0	0	8.43E-06
p__Kryptonia	0	0	0	8.43E-06
p__Chloroflexi	1.79E-06	1.05E-10	1.79E-06	0.000145704
p__Planctomycetota	0	0	0	7.22E-06
p__Kapabacteria	0	0	0	9.51E-05
p__Myxococcota	2.68E-06	2.37E-10	2.68E-06	0.000189656
p__Cyanobacteria	5.81E-05	2.61E-08	2.81E-05	0.000583417
p__Nitrospirota	8.94E-07	2.64E-11	8.94E-07	7.53E-05
p__MBNT15	0	0	0	4.27E-05
p__Deferribacteres	1.52E-05	7.62E-09	1.52E-05	6.62E-06
p__Deinococcota	1.79E-06	1.05E-10	1.79E-06	3.79E-05
**GC.Stool-N.Stool-Phylum**				
p__Bdellovibrionota	2.83E-05	7.90E-09	1.27E-05	0
p__Elusimicrobia	1.87E-05	9.89E-09	1.42E-05	0
p__Entotheonellaeota	1.69E-05	1.03E-08	1.45E-05	0
p__Armatimonadota	1.63E-05	3.92E-09	8.94E-06	0
p__Gemmatimonadota	1.26E-05	4.61E-09	9.70E-06	0
p__WPS-2	8.43E-06	3.48E-09	8.43E-06	0
p__Kryptonia	8.43E-06	1.34E-09	5.23E-06	0
p__Planctomycetota	7.22E-06	6.36E-10	3.60E-06	0
p__Deferribacteres	6.62E-06	1.13E-09	4.81E-06	0
p__Fibrobacterota	5.42E-06	5.32E-10	3.30E-06	0
p__Acidobacteriota	0.000377505	1.79E-06	0.000191195	2.95E-06
p__Chloroflexi	0.000145704	1.52E-07	5.57E-05	8.43E-07
p__Kapabacteria	9.51E-05	1.30E-07	5.16E-05	0
p__MBNT15	4.27E-05	8.20E-08	4.09E-05	0
p__Sumerlaeota	3.61E-06	2.04E-10	2.04E-06	0
p__Proteobacteria	0.119517444	0.026301708	0.023168288	0.054477983
p__Cyanobacteria	0.000583417	2.21E-06	0.000212542	5.10E-05
p__Gemmatimonadetes	3.43E-05	1.13E-08	1.52E-05	2.11E-06
p__Synergistota	0.000771869	1.34E-05	0.000523812	7.00E-05
p__Deinococcota	3.79E-05	2.97E-08	2.46E-05	1.26E-06

p__Nitrospirota	7.53E-05	1.25E-07	5.05E-05	4.21E-07
p__Caldisericota	2.41E-06	2.84E-10	2.41E-06	0
p__Myxococcota	0.000189656	3.56E-07	8.52E-05	4.64E-06
**CRC.Stool-N.Stool-Phylum**				
p__Deferribacteres	1.52E-05	7.62E-09	1.52E-05	0
p__Firmicutes	0.400814255	0.016560391	0.022401563	0.525976095
p__Gemmatimonadota	0.00010281	3.13E-07	9.73E-05	0
p__Fibrobacterota	4.47E-06	1.70E-10	2.27E-06	0
p__Elusimicrobia	3.58E-06	2.59E-10	2.80E-06	0
p__Fusobacteriota	0.043100643	0.008511082	0.016059626	0.006194157

variance. group2.	standard. error. group2.	p.value	q.value	FC	log10FC
**GC.Stool-CRC.Stool-Phylum**					
7.90E-09	1.27E-05	0.0000	0.0000	1.0283	0.0121
1.03E-08	1.45E-05	0.0000	0.0000	1.0169	0.0073
3.92E-09	8.94E-06	0.0000	0.0000	1.0163	0.0070
9.89E-09	1.42E-05	0.0004	0.0084	1.0150	0.0065
1.79E-06	0.000191195	0.0010	0.0136	1.3775	0.1391
1.13E-08	1.52E-05	0.0010	0.0136	1.0343	0.0147
3.48E-09	8.43E-06	0.0014	0.0136	1.0084	0.0036
1.34E-09	5.23E-06	0.0014	0.0136	1.0084	0.0036
1.52E-07	5.57E-05	0.0020	0.0176	1.1437	0.0583
6.36E-10	3.60E-06	0.0024	0.0177	1.0072	0.0031
1.30E-07	5.16E-05	0.0040	0.0270	1.0951	0.0395
3.56E-07	8.52E-05	0.0100	0.0628	1.1865	0.0743
2.21E-06	0.000212542	0.0180	0.0989	1.4965	0.1751
1.25E-07	5.05E-05	0.0220	0.1138	1.0743	0.0311
8.20E-08	4.09E-05	0.0270	0.1319	1.0427	0.0182
1.13E-09	4.81E-06	0.0334	0.1545	0.9916	-0.0037
2.97E-08	2.46E-05	0.0440	0.1934	1.0361	0.0154
**GC.Stool-N.Stool-Phylum**					
0	0	0.0000	0.0000	0.9725	-0.0121
0	0	0.0000	0.0000	0.9817	-0.0080
0	0	0.0000	0.0000	0.9834	-0.0073
0	0	0.0000	0.0000	0.9840	-0.0070
0	0	0.0000	0.0000	0.9875	-0.0055
0	0	0.0000	0.0000	0.9916	-0.0036
0	0	0.0000	0.0000	0.9916	-0.0036
0	0	0.0000	0.0002	0.9928	-0.0031
0	0	0.0001	0.0005	0.9934	-0.0029
0	0	0.0003	0.0023	0.9946	-0.0023
2.31E-10	1.82E-06	0.0010	0.0052	0.7281	-0.1378
4.97E-11	8.43E-07	0.0010	0.0052	0.8736	-0.0587

0	0	0.0010	0.0052	0.9131	-0.0395
0	0	0.0010	0.0052	0.9590	-0.0182
0	0	0.0049	0.0226	0.9964	-0.0016
0.006966326	0.009975918	0.0110	0.0484	0.4603	-0.3369
1.49E-08	1.46E-05	0.0150	0.0628	0.6638	-0.1780
3.11E-10	2.11E-06	0.0180	0.0719	0.9689	-0.0137
2.00E-08	1.69E-05	0.0240	0.0916	0.6039	-0.2191
3.62E-11	7.19E-07	0.0250	0.0916	0.9647	-0.0156
1.24E-11	4.21E-07	0.0270	0.0949	0.9304	-0.0313
0	0	0.0287	0.0973	0.9976	-0.0010
1.50E-09	4.64E-06	0.0320	0.1042	0.8445	-0.0734
**CRC.Stool-N.Stool-Phylum**					
0	0	0.0000	0.0000	0.9850	-0.0066
0.033025928	0.021720934	0.0010	0.0293	1.3115	0.1178
0	0	0.0010	0.0293	0.9068	-0.0425
0	0	0.0034	0.0743	0.9955	-0.0019
0	0	0.0105	0.1854	0.9964	-0.0016
0.000148756	0.001457766	0.0140	0.2051	0.1631	-0.7875

**Genus**	**mean. group1.**	**variance. group1.**	**standard. error. group1.**	**mean. group2.**
**GC.Stool-CRC.Stool-Genus**				
g__Aminobacter	2.50E-05	1.55E-08	2.16E-05	0
g__Truepera	0	0	0	2.83E-05
g__Proteiniphilum	0	0	0	2.65E-05
g__GKS98_freshwater_group	0	0	0	2.65E-05
g__Desulfitobacterium	0	0	0	2.65E-05
g__BSV13	0	0	0	2.59E-05
g__Wandonia	0	0	0	2.59E-05
g__Fusibacter	8.94E-07	2.64E-11	8.94E-07	2.83E-05
g__Taibaiella	0	0	0	2.47E-05
g__Terribacillus	2.68E-05	1.79E-08	2.33E-05	1.81E-06
g__Clostridium_sensu_stricto_7	0	0	0	2.41E-05
g__Eikenella	2.41E-05	2.85E-09	9.30E-06	1.20E-06
g__Luedemannella	0	0	0	2.29E-05
g__Thermincola	0	0	0	2.29E-05
g__[Desulfobacterium]_catecholicum_group	0	0	0	2.35E-05
g__Porphyrobacter	0	0	0	2.23E-05
g__Lacibacter	0	0	0	2.23E-05
g__Desulfonatronum	0	0	0	2.17E-05
g__Macellibacteroides	0	0	0	2.17E-05
f__Nitrosomonadaceae;g__GOUTA6	0	0	0	2.17E-05

g__Hyphomonas	0	0	0	2.11E-05
g__Ilumatobacter	0	0	0	2.05E-05
g__WCHB1-32	0	0	0	2.05E-05
g__Quadrisphaera	0	0	0	1.99E-05
g__Leptothrix	0	0	0	1.99E-05
g__Puia	0	0	0	1.99E-05
g__Devosia	1.79E-06	5.11E-11	1.24E-06	2.53E-05
g__Marmoricola	2.24E-05	1.29E-08	1.98E-05	1.81E-06
g__UCG-001	2.59E-05	3.74E-09	1.06E-05	3.01E-06
g__Syntrophococcus	0	0	0	1.93E-05
g__Phocaeicola	8.94E-07	2.64E-11	8.94E-07	2.17E-05
g__Phreatobacter	0	0	0	1.87E-05
g__Proteiniclasticum	0	0	0	1.87E-05
g__Hypnocyclicus	0	0	0	1.87E-05
g__Silanimonas	0	0	0	1.87E-05
g__Candidatus_Koribacter	0	0	0	1.75E-05
g__Angelakisella	0	0	0	1.75E-05
g__Cytophaga	0	0	0	1.75E-05
g__Vulgatibacter	0	0	0	1.81E-05
g__Erysipelotrichaceae_UCG-006	2.68E-06	2.37E-10	2.68E-06	2.53E-05
g__Thiopseudomonas	3.58E-06	2.59E-10	2.80E-06	2.65E-05
g__Clostridium_sensu_stricto_9	0	0	0	1.63E-05
g__Carnobacterium	2.86E-05	5.68E-09	1.31E-05	5.42E-06
g__Blastomonas	0	0	0	1.57E-05
g__Sulfuritalea	0	0	0	1.51E-05
g__Tannerella	8.94E-07	2.64E-11	8.94E-07	1.75E-05
g__Caulobacter	0	0	0	1.44E-05
g__RBG-16-49-21	0	0	0	1.44E-05
g__Citrifermentans	0	0	0	1.44E-05
g__Sporobacter	0	0	0	1.44E-05
g__Pseudoclostridium	1.16E-05	4.46E-09	1.16E-05	0
g__Hydrogenispora	0	0	0	1.38E-05
g__Christensenella	1.97E-05	1.67E-09	7.11E-06	3.01E-06
g__Pediococcus	4.47E-06	6.59E-10	4.47E-06	2.53E-05
g__Pelagibacterium	1.07E-05	3.20E-09	9.85E-06	0
g__Clostridioides	2.59E-05	9.62E-09	1.71E-05	6.02E-06
g__Polaromonas	0	0	0	1.26E-05
g__Actinomadura	0	0	0	1.26E-05
g__Nannocystis	0	0	0	1.26E-05
g__SWB02	0	0	0	1.32E-05
g__Paludibaculum	0	0	0	1.32E-05
g__Methyloglobulus	0	0	0	1.32E-05
g__Dinghuibacter	0	0	0	1.20E-05
g__HN-HF0106	0	0	0	1.20E-05

g__Mesorhizobium	1.97E-05	2.70E-09	9.05E-06	3.61E-06
g__Hirschia	0	0	0	1.14E-05
g__Pseudarcicella	0	0	0	1.14E-05
g__Nesterenkonia	0	0	0	1.14E-05
g__Limnobacter	0	0	0	1.14E-05
g__Pseudolabrys	0	0	0	1.08E-05
g__Pseudohongiella	0	0	0	1.08E-05
g__Tabrizicola	0	0	0	1.08E-05
g__Hyphomicrobium	0	0	0	1.02E-05
g__Luteolibacter	0	0	0	1.02E-05
g__Edaphobaculum	0	0	0	1.02E-05
g__Candidatus_Planktoluna	0	0	0	1.02E-05
g__Persicitalea	0	0	0	1.02E-05
g__Acetobacteroides	0	0	0	1.02E-05
g__Soehngenia	0	0	0	1.02E-05
g__Oxobacter	0	0	0	1.02E-05
g__Lysobacter	8.94E-07	2.64E-11	8.94E-07	1.26E-05
g__Bryobacter	0	0	0	9.63E-06
g__Methylorosula	0	0	0	9.63E-06
g__Alicyclobacillus	0	0	0	9.63E-06
g__Amphiplicatus	0	0	0	9.63E-06
g__Enorma	4.47E-06	2.24E-10	2.61E-06	1.93E-05
g__Tuzzerella	1.07E-05	1.84E-09	7.47E-06	1.20E-06
g__Caldibacillus	7.15E-06	1.69E-09	7.15E-06	0
g__Deinococcus	0	0	0	9.03E-06
g__Tagaea	0	0	0	9.03E-06
g__Fonticella	0	0	0	9.03E-06
g__UKL13-1	0	0	0	9.03E-06
g__Candidatus_Stoquefichus	2.50E-05	1.19E-08	1.90E-05	8.43E-06
g__Corynebacterium	1.70E-05	1.04E-09	5.60E-06	0.000138479
g__[Eubacterium]_siraeum_group	0.003078043	0.000156764	0.002179544	0.000204106
g__Cetobacterium	3.04E-05	2.36E-08	2.68E-05	0
g__Pedomicrobium	0	0	0	8.19E-05
g__Mycobacterium	0	0	0	4.15E-05
g__Parapedobacter	0	0	0	0.000178818
g__Ruminofilibacter	0	0	0	0.000139683
g__Burkholderia-Caballeronia-Paraburkholderia	0	0	0	9.63E-05
g__Gemmatimonas	0	0	0	3.43E-05
g__Nonomuraea	0	0	0	6.62E-05
g__Niabella	0	0	0	5.12E-05
g__Thermopolyspora	0	0	0	6.38E-05
g__Longispora	0	0	0	5.48E-05
g__Sphingorhabdus	0	0	0	0.000114396

g__Limnochorda	0	0	0	7.83E-06
g__Desulfurispora	0	0	0	7.83E-06
g__Sideroxydans	0	0	0	7.83E-06
g__Lysinibacillus	0	0	0	8.43E-06
g__SH3-11	0	0	0	8.43E-06
g__Desulfocapsa	0	0	0	8.43E-06
g__Rhodoluna	0	0	0	8.43E-06
g__Candidatus_Megaira	0	0	0	8.43E-06
g__Lachnospiraceae_UCG-004	1.25E-05	8.18E-10	4.98E-06	2.41E-06
g__[Bacteroides]_pectinophilus_group	6.26E-06	6.40E-10	4.40E-06	0
g__hgcI_clade	0	0	0	0.000135468
g__Methylobacterium-Methylorubrum	0	0	0	3.07E-05
g__Olsenella	3.22E-05	3.45E-09	1.02E-05	0.000272141
g__GCA-900066755	1.79E-05	1.68E-09	7.14E-06	5.42E-06
g__Acholeplasma	0	0	0	7.22E-06
g__Dongia	0	0	0	7.22E-06
g__Candidatus_Solibacter	0	0	0	7.22E-06
g__Rheinheimera	0	0	0	7.22E-06
g__Lachnospiraceae_NK3A20_group	0	0	0	7.22E-06
g__Blvii28_wastewater-sludge_group	0	0	0	7.22E-06
g__Friedmanniella	0	0	0	7.22E-06
g__Methylotenera	0	0	0	7.22E-06
g__Oligoflexus	0	0	0	7.22E-06
g__Thermoactinomyces	0	0	0	7.22E-06
g__Lachnospiraceae_UCG-006	9.83E-06	9.07E-10	5.24E-06	1.20E-06
g__Atopobium	2.95E-05	2.07E-09	7.91E-06	0.000564151
g__Bradyrhizobium	0	0	0	7.47E-05
g__Phenylobacterium	0	0	0	4.70E-05
g__Herbinix	2.15E-05	5.29E-09	1.27E-05	7.83E-06
g__CHKCI002	8.05E-06	7.22E-10	4.68E-06	2.23E-05
g__Ralstonia	0	0	0	0.001109035
g__TM7x	2.95E-05	6.31E-09	1.38E-05	0.001207776
g__Salinispira	0	0	0	6.62E-06
g__Anseongella	0	0	0	6.62E-06
g__Arthrobacter	0	0	0	6.62E-06
g__Actinomyces	0.000118008	2.12E-08	2.53E-05	0.000405803
g__CL500-29_marine_group	0	0	0	9.03E-05
g__Polynucleobacter	0	0	0	6.62E-05
g__Candidatus_Methylopumilus	0	0	0	3.61E-05
g__Algoriphagus	0	0	0	0.000152327

g__Cyanobium_PCC-6307	0	0	0	4.27E-05
g__Limnohabitans	0	0	0	5.78E-05
g__Sediminibacterium	0	0	0	0.000125835
g__Roseomonas	0	0	0	8.97E-05
g__Arenimonas	0	0	0	3.01E-05
g__Hydrogenophaga	0	0	0	0.000104762
g__Fluviicola	0	0	0	0.000131254
g__Candidatus_Limnoluna	8.94E-07	2.64E-11	8.94E-07	7.41E-05
g__Pseudanabaena_PCC-7429	0	0	0	0.00010416
g__Sanguibacter	1.43E-05	1.91E-09	7.61E-06	4.21E-06
g__Romboutsia	0.003540241	1.29E-05	0.000624259	0.011807425
g__Pseudofulvimonas	0	0	0	4.40E-05
g__Aestuariimicrobium	0	0	0	4.33E-05
g__Rhodococcus	8.94E-07	2.64E-11	8.94E-07	8.43E-06
g__Porphyromonas	0.015957903	0.003153966	0.009776232	0.000609909
g__Leptotrichia	1.79E-06	5.11E-11	1.24E-06	9.69E-05
g__Microcystis_PCC-7914	0	0	0	0.000221566
g__UCG-004	8.94E-07	2.64E-11	8.94E-07	7.10E-05
g__OLB12	0	0	0	5.48E-05
g__Acidibacter	0	0	0	6.02E-06
g__Tychonema_CCAP_1459-11B	0	0	0	6.02E-06
g__Clostridium_sensu_stricto_12	0	0	0	6.02E-06
g__Parvibacter	0	0	0	6.02E-06
g__Thermomonospora	0	0	0	6.02E-06
g__Chryseomicrobium	0	0	0	6.02E-06
g__Pelotomaculum	0	0	0	6.02E-06
g__Clostridium_sensu_stricto_18	0.000267306	1.41E-06	0.000207024	7.83E-06
g__Pseudoxanthomonas	8.94E-07	2.64E-11	8.94E-07	7.95E-05
g__Desulfosporosinus	0	0	0	5.06E-05
g__Fusicatenibacter	0.001845216	3.42E-06	0.000322121	0.004555351
g__Ureaplasma	4.47E-06	6.59E-10	4.47E-06	0
g__Candidatus_Arthromitus	4.47E-06	6.59E-10	4.47E-06	0
g__Egicoccus	4.47E-06	3.33E-10	3.18E-06	0
g__Anaerosporobacter	4.47E-06	2.79E-10	2.91E-06	0
g__Rikenella	1.43E-05	5.23E-09	1.26E-05	4.82E-06
g__Rhodoferax	0	0	0	6.86E-05
g__Candidatus_Aquirestis	0	0	0	3.13E-05
g__Catabacter	0.000109068	7.51E-08	4.77E-05	8.43E-06
g__Selenomonas	7.15E-06	3.28E-10	3.15E-06	1.87E-05
g__Bosea	0	0	0	5.42E-06
g__Gaiella	0	0	0	5.42E-06
g__Proteus	0	0	0	5.42E-06
g__Bdellovibrio	0	0	0	5.42E-06
g__Succiniclasticum	0	0	0	5.42E-06

g__MSBL7	0	0	0	5.42E-06
g__Pelosinus	0	0	0	5.42E-06
g__Propionivibrio	0	0	0	5.42E-06
g__Chelativorans	0	0	0	5.42E-06
g__Haliangium	2.68E-06	2.37E-10	2.68E-06	1.08E-05
g__Sarcina	0.00012516	5.48E-08	4.07E-05	0.001632846
g__Mailhella	4.47E-06	4.42E-10	3.66E-06	7.22E-05
g__Candidatus_Soleaferrea	0.000126948	3.01E-08	3.02E-05	4.40E-05
g__Peptostreptococcus	0.001432188	9.66E-06	0.000541078	0.000330543
g__UCG-012	5.36E-06	6.78E-10	4.53E-06	6.02E-07
g__Solitalea	0	0	0	3.55E-05
g__Frisingicoccus	9.92E-05	3.48E-08	3.25E-05	2.11E-05
g__Delftia	3.58E-06	1.50E-10	2.13E-06	1.20E-05
g__Hymenobacter	0	0	0	3.91E-05
g__Syntrophus	0	0	0	4.70E-05
g__Phocea	1.07E-05	4.25E-10	3.59E-06	3.07E-05
g__Anaeromyxobacter	0	0	0	0.000117406
g__Geothrix	0	0	0	0.00021976
g__Geobacter	0	0	0	3.43E-05
g__Longivirga	0	0	0	4.09E-05
g__Ercella	0	0	0	3.19E-05
g__Thermoanaerobaculum	0	0	0	3.49E-05
g__Anaerovibrio	4.92E-05	1.26E-08	1.95E-05	4.82E-06
g__Herbaspirillum	2.68E-06	1.29E-10	1.97E-06	1.02E-05
g__Pedobacter	1.79E-06	5.11E-11	1.24E-06	7.53E-05
g__Papillibacter	8.05E-06	2.32E-10	2.65E-06	1.87E-05
g__Moryella	3.49E-05	1.06E-08	1.79E-05	2.41E-06
g__Intestinimonas	0.000207408	2.61E-07	8.89E-05	4.33E-05
g__Brevundimonas	7.15E-06	3.82E-10	3.40E-06	6.98E-05
g__Blastococcus	0	0	0	4.82E-06
g__Sphingopyxis	0	0	0	4.82E-06
g__Dyadobacter	0	0	0	4.82E-06
g__Legionella	0	0	0	4.82E-06
g__Peredibacter	0	0	0	4.82E-06
g__Sulfuricurvum	0	0	0	4.82E-06
g__UBA6140	0	0	0	4.82E-06
g__Chroococcopsis	0	0	0	4.82E-06
g__Prosthecobacter	0	0	0	4.82E-06
g__Thermoflavimicrobium	0	0	0	4.82E-06
g__Lachnospiraceae_XPB1014_group	0.000225288	1.92E-07	7.62E-05	4.15E-05
g__Synergistes	8.94E-06	2.64E-09	8.94E-06	2.41E-06
g__Streptobacillus	3.58E-06	4.22E-10	3.58E-06	0
g__Helcococcus	3.58E-06	2.04E-10	2.49E-06	0

g__Treponema	0.000122478	8.12E-08	4.96E-05	1.81E-05
g__Acetobacterium	0	0	0	9.63E-05
g__Acidaminobacter	0	0	0	6.32E-05
g__Cellulosilyticum	0.000117114	7.47E-08	4.76E-05	1.32E-05
g__Coprobacillus	9.83E-06	3.08E-10	3.06E-06	3.91E-05
g__Lachnoclostridium	0.00900884	0.000300005	0.003015141	0.003643799
g__Sphingomonas	6.26E-06	4.76E-10	3.80E-06	5.36E-05
g__Peptoniphilus	8.67E-05	2.37E-08	2.68E-05	2.47E-05
g__Mucispirillum	1.52E-05	7.62E-09	1.52E-05	6.62E-06
g__Massilia	5.36E-06	2.97E-10	3.00E-06	0.000113191
g__Senegalimassilia	6.17E-05	9.97E-09	1.74E-05	0.000165572
g__Fenollaria	1.07E-05	7.52E-10	4.77E-06	2.17E-05
g__Family_XIII_AD3011_group	0.000774204	1.56E-06	0.000217114	0.000311276
g__Brachybacterium	5.90E-05	1.88E-08	2.38E-05	7.22E-06
g__Erysipelothrix	7.15E-06	8.72E-10	5.14E-06	7.41E-05
g__Arcticibacter	4.47E-06	6.59E-10	4.47E-06	6.02E-07
g__Mitsuokella	2.68E-06	1.29E-10	1.97E-06	4.46E-05
g__Prevotellaceae_UCG-003	0.000361176	4.76E-07	0.000120133	8.37E-05
g__Colidextribacter	0.000592722	8.69E-07	0.000162319	0.000250466
g__Pygmaiobacter	8.05E-06	1.70E-09	7.18E-06	2.41E-06
g__Howardella	1.43E-05	6.59E-10	4.47E-06	2.59E-05
g__OM60(NOR5)_clade	0	0	0	4.21E-06
g__Chryseolinea	0	0	0	4.21E-06
g__Erythrobacter	0	0	0	4.21E-06
g__FFCH7168	0	0	0	4.21E-06
g__Dechloromonas	0	0	0	4.21E-06
g__Planktothrix_NIVA-CYA_15	0	0	0	4.21E-06
g__Pajaroellobacter	0	0	0	4.21E-06
g__Alkaliphilus	0	0	0	4.21E-06
g__Ureibacillus	0	0	0	4.21E-06
g__Faecalicoccus	0	0	0	4.21E-06
g__Dethiosulfatibacter	0	0	0	4.21E-06
g__Luteimonas	3.58E-06	2.59E-10	2.80E-06	7.29E-05
g__Paludibacter	0	0	0	3.13E-05
**GC.Stool-N.Stool-Genus**				
g__Fusibacter	2.83E-05	2.34E-08	2.18E-05	0
g__Succinivibrio	2.83E-05	1.02E-08	1.44E-05	0
g__Truepera	2.83E-05	2.44E-08	2.23E-05	0
g__Proteiniphilum	2.65E-05	2.99E-08	2.47E-05	0
g__GKS98_freshwater_group	2.65E-05	2.86E-08	2.42E-05	0
g__Thiopseudomonas	2.65E-05	1.53E-08	1.77E-05	0
g__Desulfitobacterium	2.65E-05	1.14E-08	1.52E-05	0
g__BSV13	2.59E-05	2.60E-08	2.30E-05	0
g__Wandonia	2.59E-05	2.10E-08	2.07E-05	0

g__Pediococcus	2.53E-05	1.46E-08	1.73E-05	0
g__Taibaiella	2.47E-05	1.95E-08	2.00E-05	0
g__[Desulfobacterium]_catecholicum_group	2.35E-05	2.70E-08	2.35E-05	0
g__Luedemannella	2.29E-05	2.06E-08	2.05E-05	0
g__Thermincola	2.29E-05	7.37E-09	1.23E-05	0
g__Lacibacter	2.23E-05	8.14E-09	1.29E-05	0
g__Rahnella1	6.02E-07	1.78E-11	6.02E-07	2.91E-05
g__Desulfonatronum	2.17E-05	2.30E-08	2.17E-05	0
g__Macellibacteroides	2.17E-05	2.06E-08	2.05E-05	0
g__GOUTA6	2.17E-05	7.28E-09	1.22E-05	0
g__Hyphomonas	2.11E-05	5.91E-09	1.10E-05	0
g__WCHB1-32	2.05E-05	7.04E-09	1.20E-05	0
g__Porphyrobacter	2.23E-05	5.93E-09	1.10E-05	4.21E-07
g__Quadrisphaera	1.99E-05	5.38E-09	1.05E-05	0
g__Leptothrix	1.99E-05	3.71E-09	8.71E-06	0
g__Puia	1.99E-05	9.95E-09	1.43E-05	0
g__Allobaculum	1.93E-05	1.33E-08	1.65E-05	0
g__Nocardiopsis	1.93E-05	9.49E-09	1.39E-05	0
g__Syntrophococcus	1.93E-05	1.82E-08	1.93E-05	0
g__dgA-11_gut_group	1.93E-05	1.07E-08	1.48E-05	0
g__Proteiniclasticum	1.87E-05	8.44E-09	1.31E-05	0
g__Hypnocyclicus	1.87E-05	8.58E-09	1.32E-05	0
g__Silanimonas	1.87E-05	3.98E-09	9.01E-06	0
g__Vulgatibacter	1.81E-05	8.73E-09	1.34E-05	0
g__Peptoanaerobacter	0	0	0	2.11E-05
g__Candidatus_Koribacter	1.75E-05	1.39E-08	1.69E-05	0
g__Angelakisella	1.75E-05	1.29E-08	1.63E-05	0
g__Cytophaga	1.75E-05	8.30E-09	1.30E-05	0
g__Anaerostignum	1.81E-06	5.11E-11	1.02E-06	2.57E-05
g__Clostridium_sensu_stricto_9	1.63E-05	5.48E-09	1.06E-05	0
g__Erysipelotrichaceae_UCG-006	2.53E-05	1.22E-08	1.58E-05	2.95E-06
g__CAG-873	1.51E-05	9.40E-09	1.38E-05	0
g__Sulfuritalea	1.51E-05	3.74E-09	8.74E-06	0
g__Caulobacter	1.44E-05	4.28E-09	9.35E-06	0
g__RBG-16-49-21	1.44E-05	1.02E-08	1.44E-05	0
g__Citrifermentans	1.44E-05	1.02E-08	1.44E-05	0
g__Sporobacter	1.44E-05	3.70E-09	8.69E-06	0
g__Enorma	1.93E-05	7.35E-09	1.22E-05	1.26E-06
g__Clostridium_sensu_stricto_7	2.41E-05	1.80E-08	1.92E-05	2.95E-06
g__Hydrogenispora	1.38E-05	4.83E-09	9.93E-06	0
g__SWB02	1.32E-05	2.54E-09	7.20E-06	0
g__Paludibaculum	1.32E-05	2.32E-09	6.89E-06	0
g__Methyloglobulus	1.32E-05	3.70E-09	8.69E-06	0

g__Devosia	2.53E-05	6.89E-09	1.19E-05	4.21E-06
g__Phreatobacter	1.87E-05	4.67E-09	9.76E-06	1.69E-06
g__Polaromonas	1.26E-05	6.42E-09	1.14E-05	0
g__Actinomadura	1.26E-05	7.83E-09	1.26E-05	0
g__Nannocystis	1.26E-05	6.46E-09	1.15E-05	0
g__Delftia	1.20E-05	2.75E-09	7.50E-06	0
g__Dinghuibacter	1.20E-05	2.64E-09	7.35E-06	0
g__HN-HF0106	1.20E-05	7.11E-09	1.20E-05	0
g__Hirschia	1.14E-05	1.44E-09	5.43E-06	0
g__Pseudarcicella	1.14E-05	2.17E-09	6.65E-06	0
g__Nesterenkonia	1.14E-05	2.10E-09	6.54E-06	0
g__Limnobacter	1.14E-05	1.95E-09	6.31E-06	0
g__Blastomonas	1.57E-05	2.29E-09	6.83E-06	1.26E-06
g__Lysobacter	1.26E-05	1.99E-09	6.38E-06	4.21E-07
g__Haliangium	1.08E-05	1.26E-09	5.07E-06	0
g__Pseudolabrys	1.08E-05	2.13E-09	6.59E-06	0
g__Pseudohongiella	1.08E-05	1.66E-09	5.82E-06	0
g__Promicromonospora	1.08E-05	3.14E-09	8.01E-06	0
g__Tabrizicola	1.08E-05	2.06E-09	6.48E-06	0
g__Hyphomicrobium	1.02E-05	1.25E-09	5.06E-06	0
g__Luteolibacter	1.02E-05	2.01E-09	6.41E-06	0
g__Edaphobaculum	1.02E-05	1.43E-09	5.41E-06	0
g__Candidatus_Planktoluna	1.02E-05	2.27E-09	6.80E-06	0
g__Persicitalea	1.02E-05	4.55E-09	9.64E-06	0
g__Acetobacteroides	1.02E-05	4.01E-09	9.05E-06	0
g__Oxobacter	1.02E-05	2.20E-09	6.69E-06	0
g__Bryobacter	9.63E-06	1.72E-09	5.92E-06	0
g__Methylorosula	9.63E-06	2.30E-09	6.85E-06	0
g__Prevotellaceae_UCG-004	9.63E-06	4.00E-09	9.04E-06	0
g__Alicyclobacillus	9.63E-06	4.00E-09	9.04E-06	0
g__Amphiplicatus	9.63E-06	1.17E-09	4.90E-06	0
g__Deinococcus	9.03E-06	2.62E-09	7.31E-06	0
g__Tagaea	9.03E-06	3.02E-09	7.85E-06	0
g__Fonticella	9.03E-06	2.04E-09	6.45E-06	0
g__UKL13-1	9.03E-06	1.35E-09	5.25E-06	0
g__Clostridium_sensu_stricto_2	1.20E-05	2.57E-09	7.24E-06	8.43E-07
g__Soehngenia	1.02E-05	2.45E-09	7.07E-06	4.21E-07
g__Helcococcus	0	0	0	1.05E-05
g__SH3-11	8.43E-06	1.27E-09	5.09E-06	0
g__Desulfocapsa	8.43E-06	2.21E-09	6.72E-06	0
g__Rhodoluna	8.43E-06	1.31E-09	5.16E-06	0
g__Candidatus_Megaira	8.43E-06	9.07E-10	4.30E-06	0
g__unidentified_Prevotellaceae	3.61E-06	2.41E-10	2.22E-06	1.90E-05
g__[Eubacterium]_saphenum_group	2.41E-06	1.75E-10	1.89E-06	1.64E-05

g__Clostridium_sensu_stricto_18	7.83E-06	2.57E-09	7.24E-06	0
g__Limnochorda	7.83E-06	1.84E-09	6.13E-06	0
g__Herbinix	7.83E-06	6.45E-10	3.63E-06	0
g__Desulfurispora	7.83E-06	1.04E-09	4.61E-06	0
g__Sideroxydans	7.83E-06	7.90E-10	4.01E-06	0
g__Vibrio	4.82E-06	3.75E-10	2.77E-06	2.02E-05
g__Mycoplasma	2.41E-06	1.03E-10	1.45E-06	1.56E-05
g__Ilumatobacter	2.05E-05	4.98E-09	1.01E-05	5.48E-06
g__Epulopiscium	2.41E-05	3.58E-09	8.55E-06	7.59E-06
g__Candidatus_Solibacter	7.22E-06	1.29E-09	5.13E-06	0
g__Blvii28_wastewater-sludge_group	7.22E-06	9.98E-10	4.51E-06	0
g__Brachybacterium	7.22E-06	6.36E-10	3.60E-06	0
g__Friedmanniella	7.22E-06	2.16E-09	6.64E-06	0
g__Leucobacter	7.22E-06	2.16E-09	6.64E-06	0
g__Methylotenera	7.22E-06	2.16E-09	6.64E-06	0
g__Oligoflexus	7.22E-06	1.29E-09	5.13E-06	0
g__Thermoactinomyces	7.22E-06	1.29E-09	5.13E-06	0
g__Rhodococcus	8.43E-06	9.43E-10	4.39E-06	4.21E-07
g__Pseudopropionibacterium	1.81E-06	5.11E-11	1.02E-06	1.35E-05
g__Mucispirillum	6.62E-06	1.13E-09	4.81E-06	0
g__Salinispira	6.62E-06	1.28E-09	5.11E-06	0
g__Anseongella	6.62E-06	1.17E-09	4.89E-06	0
g__Stenotrophomonas	1.14E-05	1.99E-09	6.37E-06	1.69E-06
g__Papillibacter	1.87E-05	4.09E-09	9.13E-06	5.48E-06
g__Acidibacter	6.02E-06	4.34E-10	2.98E-06	0
g__Tychonema_CCAP_1459-11B	6.02E-06	1.01E-09	4.55E-06	0
g__Clostridioides	6.02E-06	7.25E-10	3.85E-06	0
g__Clostridium_sensu_stricto_12	6.02E-06	7.97E-10	4.03E-06	0
g__Thermomonospora	6.02E-06	1.78E-09	6.02E-06	0
g__Chryseomicrobium	6.02E-06	1.16E-09	4.87E-06	0
g__Pelotomaculum	6.02E-06	8.70E-10	4.21E-06	0
g__Lysinibacillus	8.43E-06	1.92E-09	6.26E-06	8.43E-07
g__Dongia	7.22E-06	2.16E-09	6.64E-06	4.21E-07
g__Ileibacterium	7.22E-06	2.56E-09	7.22E-06	4.21E-07
g__Fenollaria	2.17E-05	3.47E-09	8.42E-06	8.01E-06
g__Georgenia	5.42E-06	5.68E-10	3.41E-06	0
g__Gaiella	5.42E-06	6.77E-10	3.72E-06	0
g__Aurantimicrobium	5.42E-06	7.13E-10	3.82E-06	0
g__Bdellovibrio	5.42E-06	3.15E-10	2.53E-06	0
g__Succiniclasticum	5.42E-06	1.44E-09	5.42E-06	0
g__Elusimicrobium	5.42E-06	1.44E-09	5.42E-06	0
g__MSBL7	5.42E-06	1.44E-09	5.42E-06	0
g__Pelosinus	5.42E-06	1.15E-09	4.84E-06	0

g__Propionivibrio	5.42E-06	5.32E-10	3.30E-06	0
g__Chelativorans	5.42E-06	4.96E-10	3.18E-06	0
g__Asteroleplasma	0	0	0	6.32E-06
g__Arthrobacter	6.62E-06	7.35E-10	3.87E-06	4.21E-07
g__Fretibacterium	6.02E-07	1.78E-11	6.02E-07	8.01E-06
g__Paenibacillus	1.75E-05	4.68E-09	9.77E-06	5.90E-06
g__Curvibacter	4.82E-06	7.02E-10	3.78E-06	0
g__Rikenella	4.82E-06	1.14E-09	4.82E-06	0
g__Blastococcus	4.82E-06	3.03E-10	2.49E-06	0
g__Saccharopolyspora	4.82E-06	8.83E-10	4.24E-06	0
g__Arcobacter	4.82E-06	8.83E-10	4.24E-06	0
g__Fibrobacter	4.82E-06	5.20E-10	3.26E-06	0
g__Dyadobacter	4.82E-06	5.20E-10	3.26E-06	0
g__Legionella	4.82E-06	6.65E-10	3.68E-06	0
g__Anaerovibrio	4.82E-06	4.48E-10	3.02E-06	0
g__Peredibacter	4.82E-06	4.84E-10	3.14E-06	0
g__Sulfuricurvum	4.82E-06	8.83E-10	4.24E-06	0
g__UBA6140	4.82E-06	8.83E-10	4.24E-06	0
g__Chroococcopsis	4.82E-06	8.83E-10	4.24E-06	0
g__Prosthecobacter	4.82E-06	4.84E-10	3.14E-06	0
g__Thermoflavimicrobium	4.82E-06	5.93E-10	3.48E-06	0
g__Micrococcus	6.02E-06	7.25E-10	3.85E-06	4.21E-07
g__Lachnospira	0.001803235	7.17E-06	0.000382434	0.018155535
g__Lactobacillus	0.018017297	0.002414722	0.007019974	0.000955865
g__[Eubacterium]_siraeum_group	0.000204106	2.03E-07	6.43E-05	0.002840622
g__Lachnoclostridium	0.003643799	1.16E-05	0.000485575	0.007831518
g__CL500-29_marine_group	9.03E-05	9.69E-08	4.45E-05	0
g__Peptostreptococcus	0.000330543	2.64E-07	7.34E-05	5.69E-05
g__Aerococcus	9.03E-05	3.94E-07	8.97E-05	0
g__hgcI_clade	0.000135468	2.51E-07	7.16E-05	0
g__Polynucleobacter	6.62E-05	5.76E-08	3.43E-05	0
g__Schlegelella	0	0	0	0.000191763
g__Prevotellaceae_Ga6A1_group	0.000226985	7.62E-07	0.000124723	0
g__Cellulomonas	6.56E-05	8.86E-08	4.25E-05	0
g__Vulcaniibacterium	6.02E-07	1.78E-11	6.02E-07	0.000225058
g__Dysgonomonas	0.000416641	3.49E-06	0.000267013	2.53E-06
g__Peptoclostridium	5.42E-05	5.33E-08	3.30E-05	0
g__Pedomicrobium	8.19E-05	1.47E-07	5.48E-05	0
g__Candidatus_Methylopumilus	3.61E-05	1.48E-08	1.74E-05	0
g__Dubosiella	0.000121018	4.67E-07	9.76E-05	0
g__Nocardioides	3.85E-05	2.05E-08	2.04E-05	0
g__Streptomyces	0.000240231	1.82E-06	0.000192773	0
g__Algoriphagus	0.000152327	3.91E-07	8.94E-05	0
g__Mycobacterium	4.15E-05	1.76E-08	1.89E-05	0

g__Bradyrhizobium	7.47E-05	1.53E-07	5.60E-05	0
g__Parapedobacter	0.000178818	1.04E-06	0.000145638	0
g__Cyanobium_PCC-6307	4.27E-05	1.93E-08	1.98E-05	0
g__Alcaligenes	3.31E-05	4.01E-08	2.86E-05	0
g__Anaeromyxobacter	0.000117406	2.22E-07	6.73E-05	0
g__Alkalibacter	0.000114396	6.21E-07	0.000112566	0
g__Limnohabitans	5.78E-05	5.54E-08	3.36E-05	0
g__Geothrix	0.00021976	8.70E-07	0.000133252	0
g__Ruminofilibacter	0.000139683	5.46E-07	0.000105528	0
g__Trichococcus	0.000192064	8.19E-07	0.000129265	0
g__Sediminibacterium	0.000125835	5.16E-07	0.000102595	0
g__Anaerotruncus	0.00011861	5.70E-08	3.41E-05	2.40E-05
g__Phenylobacterium	4.70E-05	2.85E-08	2.41E-05	0
g__Rhodoferax	6.86E-05	6.19E-08	3.55E-05	0
g__Erysipelothrix	7.41E-05	4.96E-08	3.18E-05	4.21E-07
g__Arenimonas	3.01E-05	9.16E-09	1.37E-05	0
g__Microcystis_PCC-7914	0.000221566	4.90E-07	1.00E-04	0
g__Prevotellaceae_UCG-003	8.37E-05	1.83E-07	6.10E-05	0
g__Fluviicola	0.000131254	2.00E-07	6.39E-05	0
g__Acetobacterium	9.63E-05	1.77E-07	6.00E-05	0
g__Nonomuraea	6.62E-05	1.29E-07	5.12E-05	0
g__Niabella	5.12E-05	9.71E-08	4.45E-05	0
g__Thermopolyspora	6.38E-05	9.87E-08	4.49E-05	0
g__Syntrophus	4.70E-05	9.20E-08	4.33E-05	0
g__Candidatus_Limnoluna	7.41E-05	5.82E-08	3.45E-05	0
g__Pseudanabaena_PCC-7429	0.00010416	1.26E-07	5.07E-05	0
g__Longispora	5.48E-05	8.29E-08	4.11E-05	0
g__Sphingorhabdus	0.000114396	1.21E-07	4.96E-05	0
g__Pseudofulvimonas	4.40E-05	4.89E-08	3.16E-05	0
g__Lachnospiraceae_XPB1014_group	4.15E-05	4.16E-08	2.91E-05	0
g__Geobacter	3.43E-05	4.45E-08	3.01E-05	0
g__Listeria	3.01E-05	4.44E-08	3.01E-05	0
g__Longivirga	4.09E-05	4.14E-08	2.91E-05	0
g__Desulfosporosinus	5.06E-05	4.27E-08	2.95E-05	0
g__unidentified_P5D1-392	3.01E-05	3.44E-08	2.65E-05	0
g__Acidaminobacter	6.32E-05	5.56E-08	3.37E-05	0
g__Ercella	3.19E-05	2.93E-08	2.45E-05	0
g__Aestuariimicrobium	4.33E-05	3.34E-08	2.61E-05	0
g__Candidatus_Aquirestis	3.13E-05	1.11E-08	1.50E-05	0
g__Paludibacter	3.13E-05	2.05E-08	2.05E-05	0
g__OLB12	5.48E-05	3.06E-08	2.50E-05	0
g__Thermoanaerobaculum	3.49E-05	1.69E-08	1.85E-05	0
g__Frisingicoccus	2.11E-05	7.07E-09	1.20E-05	8.85E-06

g__Streptococcus	0.027971514	0.001860949	0.006162674	0.005643734
g__Neisseria	3.49E-05	1.99E-08	2.01E-05	0.000441687
g__Odoribacter	0.000887469	2.66E-06	0.000233176	0.000268468
g__Lachnospiraceae_UCG-010	0.002304167	7.56E-06	0.000392911	0.000920463
g__Anaerostipes	0.00118008	3.20E-06	0.000255447	0.002491655
g__F0058	1.20E-06	3.48E-11	8.43E-07	3.67E-05
g__Roseomonas	8.97E-05	7.77E-08	3.98E-05	4.21E-07
g__Candidatus_Stoquefichus	8.43E-06	9.79E-10	4.47E-06	0.000109157
g__Eubacterium	8.25E-05	6.96E-08	3.77E-05	8.01E-06
g__Morganella	1.81E-06	5.11E-11	1.02E-06	3.08E-05
g__OM60(NOR5)_clade	4.21E-06	4.71E-10	3.10E-06	0
g__Chryseolinea	4.21E-06	4.35E-10	2.98E-06	0
g__Erythrobacter	4.21E-06	2.54E-10	2.28E-06	0
g__FFCH7168	4.21E-06	8.70E-10	4.21E-06	0
g__Dechloromonas	4.21E-06	2.54E-10	2.28E-06	0
g__Planktothrix_NIVA-CYA_15	4.21E-06	8.70E-10	4.21E-06	0
g__Sanguibacter	4.21E-06	4.35E-10	2.98E-06	0
g__Pajaroellobacter	4.21E-06	4.35E-10	2.98E-06	0
g__Alkaliphilus	4.21E-06	8.70E-10	4.21E-06	0
g__Ureibacillus	4.21E-06	3.63E-10	2.72E-06	0
g__Dethiosulfatibacter	4.21E-06	4.35E-10	2.98E-06	0
g__W5053	6.02E-07	1.78E-11	6.02E-07	6.74E-06
g__Sphingobacterium	1.20E-05	2.14E-09	6.60E-06	2.61E-05
g__Capnocytophaga	4.21E-06	5.08E-10	3.22E-06	6.07E-05
g__Colidextribacter	0.000250466	6.82E-08	3.73E-05	0.000553373
g__Paraprevotella	0.000880846	1.49E-06	0.000174646	0.002366904
g__[Ruminococcus]_gauvreauii_group	0.000242037	1.35E-07	5.24E-05	0.000521343
g__Demequina	4.82E-05	2.59E-08	2.30E-05	4.21E-07
g__Microbacterium	4.82E-06	4.12E-10	2.90E-06	4.21E-07
g__Vagococcus	3.61E-06	1.68E-10	1.85E-06	0
g__Steroidobacter	3.61E-06	4.58E-10	3.06E-06	0
g__Clade_Ia	3.61E-06	6.39E-10	3.61E-06	0
g__Planomicrobium	3.61E-06	1.68E-10	1.85E-06	0
g__Xanthobacter	3.61E-06	6.39E-10	3.61E-06	0
g__Bauldia	3.61E-06	2.41E-10	2.22E-06	0
g__Mesorhizobium	3.61E-06	1.32E-10	1.64E-06	0
g__Tumebacillus	3.61E-06	2.41E-10	2.22E-06	0
g__Parascardovia	3.61E-06	3.13E-10	2.53E-06	0
g__SM1A02	3.61E-06	3.49E-10	2.67E-06	0
g__Sumerlaea	3.61E-06	2.04E-10	2.04E-06	0
g__[Anaerorhabdus]_furcosa_group	3.61E-06	3.13E-10	2.53E-06	0
g__Gracilibacter	3.61E-06	4.58E-10	3.06E-06	0
g__Roseiarcus	3.61E-06	3.49E-10	2.67E-06	0

g__Lacihabitans	3.61E-06	2.41E-10	2.22E-06	0
g__Azoarcus	3.61E-06	6.39E-10	3.61E-06	0
g__Pigmentiphaga	3.61E-06	4.58E-10	3.06E-06	0
g__C1-B045	3.61E-06	4.58E-10	3.06E-06	0
g__Psychrobacillus	3.61E-06	3.13E-10	2.53E-06	0
g__IMCC26134	3.61E-06	2.04E-10	2.04E-06	0
g__Dolichospermum_NIES41	3.61E-06	3.49E-10	2.67E-06	0
g__Clostridium_sensu_stricto_3	3.61E-06	2.41E-10	2.22E-06	0
g__Faecalibacterium	0.075790052	0.004414175	0.009491322	0.123499191
g__Anaerovorax	2.95E-05	1.12E-08	1.51E-05	4.21E-07
g__Psychrobacter	5.42E-06	3.51E-10	2.68E-06	7.84E-05
g__Filifactor	6.62E-06	1.21E-09	4.96E-06	7.63E-05
g__Family_XIII_AD3011_group	0.000311276	2.20E-07	6.69E-05	0.000126437
g__Enterorhabdus	1.14E-05	3.15E-09	8.02E-06	3.79E-06
g__Shuttleworthia	4.21E-06	2.54E-10	2.28E-06	9.99E-05
g__Pseudoxanthomonas	7.95E-05	2.03E-07	6.44E-05	4.21E-07
g__Lachnospiraceae_UCG-001	0.000174002	7.01E-08	3.78E-05	0.000427779
g__Family_XIII_UCG-001	7.16E-05	1.04E-08	1.45E-05	0.000132759
g__Eikenella	1.20E-06	7.11E-11	1.20E-06	6.74E-06
g__Acinetobacter	0.000137275	3.47E-07	8.42E-05	0.001223912
g__Pantoea	0.00126678	1.08E-05	0.000469859	0.000155518
g__Methylobacterium-Methylorubrum	3.07E-05	1.20E-08	1.57E-05	4.21E-07
g__Chryseobacterium	2.41E-06	1.75E-10	1.89E-06	0.000227587
g__Lachnospiraceae_NC2004_group	6.14E-05	5.25E-09	1.04E-05	0.000121801
**CRC.Stool-N.Stool-Genus**				
g__Terribacillus	2.68E-05	1.79E-08	2.33E-05	0
g__Clostridioides	2.59E-05	9.62E-09	1.71E-05	0
g__Listeria	2.50E-05	2.07E-08	2.50E-05	0
g__Aminobacter	2.50E-05	1.55E-08	2.16E-05	0
g__Demequina	2.68E-05	2.22E-08	2.59E-05	4.21E-07
g__Marmoricola	2.24E-05	1.29E-08	1.98E-05	0
g__UCG-001	2.59E-05	3.74E-09	1.06E-05	8.43E-07
g__Herbinix	2.15E-05	5.29E-09	1.27E-05	0
g__Rahnella1	0	0	0	2.91E-05
g__Mesorhizobium	1.97E-05	2.70E-09	9.05E-06	0
g__Mucispirillum	1.52E-05	7.62E-09	1.52E-05	0
g__Leucobacter	1.52E-05	2.02E-09	7.82E-06	0
g__Peptoanaerobacter	0	0	0	2.11E-05
g__Rikenella	1.43E-05	5.23E-09	1.26E-05	0
g__Sanguibacter	1.43E-05	1.91E-09	7.61E-06	0
g__Aurantimicrobium	1.25E-05	3.21E-09	9.86E-06	0
g__Vibrio	8.94E-07	2.64E-11	8.94E-07	2.02E-05
g__[Eubacterium]_saphenum_group	0	0	0	1.64E-05

g__Pseudoclostridium	1.16E-05	4.46E-09	1.16E-05	0
g__Promicromonospora	1.16E-05	1.25E-09	6.15E-06	0
g__Ileibacterium	1.34E-05	5.93E-09	1.34E-05	4.21E-07
g__Anaerostignum	3.58E-06	9.56E-11	1.70E-06	2.57E-05
g__Mycoplasma	0	0	0	1.56E-05
g__Tuzzerella	1.07E-05	1.84E-09	7.47E-06	0
g__Pelagibacterium	1.07E-05	3.20E-09	9.85E-06	0
g__Proteus	0	0	0	1.31E-05
g__Sphingobacterium	5.36E-06	2.97E-10	3.00E-06	2.61E-05
g__Enterorhabdus	1.97E-05	9.56E-09	1.70E-05	3.79E-06
g__Pygmaiobacter	8.05E-06	1.70E-09	7.18E-06	0
g__Cardiobacterium	3.58E-06	1.50E-10	2.13E-06	2.07E-05
g__Paenibacillus	2.24E-05	1.65E-08	2.24E-05	5.90E-06
g__Eikenella	2.41E-05	2.85E-09	9.30E-06	6.74E-06
g__Mailhella	4.47E-06	4.42E-10	3.66E-06	2.19E-05
g__Rheinheimera	0	0	0	1.10E-05
g__Synergistes	8.94E-06	2.64E-09	8.94E-06	4.21E-07
g__Lentimicrobium	8.94E-07	2.64E-11	8.94E-07	1.31E-05
g__Phocaeicola	8.94E-07	2.64E-11	8.94E-07	1.31E-05
g__Centipeda	3.58E-06	2.04E-10	2.49E-06	1.94E-05
g__Caldibacillus	7.15E-06	1.69E-09	7.15E-06	0
g__Lachnospiraceae_NK3A20_group	0	0	0	1.05E-05
g__CAG-352	2.86E-05	5.52E-09	1.29E-05	1.05E-05
g__UCG-004	8.94E-07	2.64E-11	8.94E-07	1.22E-05
g__Jeotgalicoccus	8.05E-06	1.70E-09	7.18E-06	4.21E-07
g__CHKCI002	8.05E-06	7.22E-10	4.68E-06	2.57E-05
g__Enhydrobacter	0	0	0	9.27E-06
g__[Bacteroides]_pectinophilus_group	6.26E-06	6.40E-10	4.40E-06	0
g__Pseudarcobacter	0	0	0	8.43E-06
g__Erysipelothrix	7.15E-06	8.72E-10	5.14E-06	4.21E-07
g__Selenomonas	7.15E-06	3.28E-10	3.15E-06	2.23E-05
g__Lachnospira	0.001705752	4.52E-06	0.000370279	0.018155535
g__Neisseria	9.83E-06	9.07E-10	5.24E-06	0.000441687
g__Lautropia	8.94E-07	2.64E-11	8.94E-07	0.000315671
g__Romboutsia	0.003540241	1.29E-05	0.000624259	0.022832024
g__Corynebacterium	1.70E-05	1.04E-09	5.60E-06	0.000152146
g__Leptotrichia	1.79E-06	5.11E-11	1.24E-06	0.000169004
g__Fusicatenibacter	0.001845216	3.42E-06	0.000322121	0.008341903
g__Peptostreptococcus	0.001432188	9.66E-06	0.000541078	5.69E-05
g__Cetobacterium	3.04E-05	2.36E-08	2.68E-05	0
g__Aerococcus	0.000159132	8.36E-07	0.000159132	0
g__Tannerella	8.94E-07	2.64E-11	8.94E-07	8.51E-05

g__Schlegelella	0	0	0	0.000191763
g__Prevotellaceae_Ga6A1_group	0.000905622	1.20E-05	0.00060216	0
g__Cellulomonas	3.31E-05	2.20E-08	2.58E-05	0
g__F0058	0	0	0	3.67E-05
g__Peptoclostridium	0.000498852	4.78E-06	0.000380491	0
g__Dubosiella	0.000193104	1.23E-06	0.000193104	0
g__Streptomyces	0.000347766	8.50E-07	0.000160462	0
g__Clostridium_sensu_stricto_18	0.000267306	1.41E-06	0.000207024	0
g__Family_XIII_AD3011_group	0.000774204	1.56E-06	0.000217114	0.000126437
g__Lachnospiraceae_UCG-001	0.000117114	3.09E-08	3.06E-05	0.000427779
g__Prevotellaceae_UCG-003	0.000361176	4.76E-07	0.000120133	0
g__Allobaculum	8.94E-05	2.38E-07	8.48E-05	0
g__Ammoniibacillus	7.24E-05	1.23E-07	6.10E-05	0
g__Lachnospiraceae_XPB1014_group	0.000225288	1.92E-07	7.62E-05	0
g__Succinivibrio	0.000140358	1.22E-07	6.07E-05	0
g__Nocardiopsis	4.92E-05	2.46E-08	2.73E-05	0
g__Morganella	8.94E-07	2.64E-11	8.94E-07	3.08E-05
g__Prevotellaceae_UCG-004	4.02E-05	9.07E-09	1.66E-05	0
g__Brachybacterium	5.90E-05	1.88E-08	2.38E-05	0
g__CAG-873	3.49E-05	5.85E-09	1.33E-05	0
g__dgA-11_gut_group	4.56E-05	8.49E-09	1.60E-05	0
g__Anaerovibrio	4.92E-05	1.26E-08	1.95E-05	0
g__Tepidimicrobium	5.36E-06	9.49E-10	5.36E-06	0
g__Pseudonocardia	5.36E-06	2.97E-10	3.00E-06	0
g__[Anaerorhabdus]_furcosa_group	5.36E-06	3.51E-10	3.26E-06	0
g__UCG-012	5.36E-06	6.78E-10	4.53E-06	0
g__Stenotrophomonas	9.83E-06	9.61E-10	5.40E-06	1.69E-06
g__Lachnospiraceae_UCG-006	9.83E-06	9.07E-10	5.24E-06	1.69E-06
g__UCG-008	1.07E-05	5.88E-10	4.22E-06	2.11E-06
g__Pedobacter	1.79E-06	5.11E-11	1.24E-06	1.22E-05
g__Lachnospiraceae_UCG-010	0.004414573	4.53E-05	0.001171773	0.000920463
g__Adlercreutzia	5.54E-05	1.05E-08	1.79E-05	0.000423143
g__Catabacter	0.000109068	7.51E-08	4.77E-05	3.79E-06
g__Ezakiella	1.79E-06	5.11E-11	1.24E-06	1.14E-05
g__W5053	0	0	0	6.74E-06
g__Christensenella	1.97E-05	1.67E-09	7.11E-06	7.59E-06
g__Ureaplasma	4.47E-06	6.59E-10	4.47E-06	0
g__Georgenia	4.47E-06	2.24E-10	2.61E-06	0
g__Sinibacillus	4.47E-06	6.59E-10	4.47E-06	0
g__Candidatus_Arthromitus	4.47E-06	6.59E-10	4.47E-06	0
g__Fibrobacter	4.47E-06	1.70E-10	2.27E-06	0
g__Pediococcus	4.47E-06	6.59E-10	4.47E-06	0
g__Arcticibacter	4.47E-06	6.59E-10	4.47E-06	0

g__Catenisphaera	4.47E-06	2.24E-10	2.61E-06	0
g__Egicoccus	4.47E-06	3.33E-10	3.18E-06	0
g__Anaerosporobacter	4.47E-06	2.79E-10	2.91E-06	0
g__GCA-900066755	1.79E-05	1.68E-09	7.14E-06	6.74E-06
g__Prevotellaceae_NK3B31_group	0.000707154	1.03E-06	0.00017673	5.35E-05
g__F0332	4.47E-06	4.42E-10	3.66E-06	1.56E-05
g__Psychrobacter	8.94E-07	2.64E-11	8.94E-07	7.84E-05
g__Vulcaniibacterium	8.94E-07	2.64E-11	8.94E-07	0.000225058
g__Frisingicoccus	9.92E-05	3.48E-08	3.25E-05	8.85E-06
g__Paracoccus	6.26E-06	3.68E-10	3.34E-06	1.81E-05
g__[Eubacterium]_eligens_group	0.002126826	1.73E-05	0.000724931	0.00739826
g__Gardnerella	2.68E-06	2.37E-10	2.68E-06	6.66E-05
g__Fretibacterium	8.94E-07	2.64E-11	8.94E-07	8.01E-06
g__Anaerofilum	1.16E-05	7.04E-10	4.62E-06	3.37E-06
g__UCG-009	0.000127842	2.69E-08	2.86E-05	2.99E-05
g__Phascolarctobacterium	0.004369873	5.25E-05	0.001261256	0.011453943
g__Turicibacter	0.000194892	7.33E-08	4.71E-05	0.00089939
g__Lachnoanaerobaculum	1.70E-05	2.34E-09	8.42E-06	0.000137395
g__Capnocytophaga	2.68E-06	1.29E-10	1.97E-06	6.07E-05
g__Delftia	3.58E-06	1.50E-10	2.13E-06	0
g__Trichococcus	3.58E-06	2.59E-10	2.80E-06	0
g__Thiopseudomonas	3.58E-06	2.59E-10	2.80E-06	0
g__Elusimicrobium	3.58E-06	2.59E-10	2.80E-06	0
g__Quinella	3.58E-06	4.22E-10	3.58E-06	0
g__unidentified_Ruminococcaceae	8.76E-05	1.32E-07	6.32E-05	1.26E-06
g__Candidatus_Soleaferrea	0.000126948	3.01E-08	3.02E-05	3.46E-05
g__Desulfobulbus	0	0	0	5.06E-06
g__Clostridium_sensu_stricto_13	9.48E-05	1.67E-07	7.11E-05	2.53E-06
g__Ilumatobacter	0	0	0	5.48E-06
g__Pseudopropionibacterium	4.47E-06	2.24E-10	2.61E-06	1.35E-05
g__Microbacterium	4.47E-06	2.24E-10	2.61E-06	4.21E-07
g__Anaerovorax	4.47E-06	1.70E-10	2.27E-06	4.21E-07
g__Pseudomonas	0.000328992	8.25E-07	0.000158099	0.001562342
g__UBA1819	0.00210537	1.60E-05	0.000697205	0.000435787
g__Filifactor	8.94E-06	1.17E-09	5.95E-06	7.63E-05
g__Mitsuokella	2.68E-06	1.29E-10	1.97E-06	0.000152146
g__Actinomyces	0.000118008	2.12E-08	2.53E-05	0.00029797
g__Agathobacter	0.015568119	0.000391885	0.003446058	0.031262854
g__Parvimonas	0.004964383	0.000566943	0.004144889	0.000106207
g__Massilia	5.36E-06	2.97E-10	3.00E-06	0.000107893
g__Coprococcus	0.003202309	8.85E-06	0.000517881	0.005426262
g__Stomatobaculum	8.94E-06	1.11E-09	5.81E-06	8.68E-05
g__Paludicola	5.90E-05	1.16E-08	1.88E-05	1.35E-05
g__Megamonas	0.005484691	0.000322879	0.003127973	0.02682196

g__Chryseobacterium	3.58E-06	2.59E-10	2.80E-06	0.000227587
g__Anaerotruncus	0.000112644	6.26E-08	4.36E-05	2.40E-05
g__Odoribacter	0.00082248	1.98E-06	0.000245014	0.000268468
g__Fusobacterium	0.043046109	0.008512949	0.016061387	0.006021359
g__Porphyromonas	0.015957903	0.003153966	0.009776232	0.000684447
g__Acinetobacter	0.0002682	6.17E-07	0.000136713	0.001223912
g__Shewanella	8.94E-07	2.64E-11	8.94E-07	6.32E-06
g__Asteroleplasma	8.94E-07	2.64E-11	8.94E-07	6.32E-06
g__Parasutterella	0.002722231	4.97E-05	0.001226888	0.008399643
g__Nocardioides	2.68E-06	7.42E-11	1.50E-06	0
g__Haliangium	2.68E-06	2.37E-10	2.68E-06	0
g__Litorimicrobium	2.68E-06	2.37E-10	2.68E-06	0
g__Rodentibacter	2.68E-06	2.37E-10	2.68E-06	0
g__Saccharopolyspora	2.68E-06	1.29E-10	1.97E-06	0
g__Acetobacter	2.68E-06	1.29E-10	1.97E-06	0
g__Harryflintia	2.68E-06	1.29E-10	1.97E-06	0
g__Brachyspira	2.68E-06	2.37E-10	2.68E-06	0
g__Faecalibacterium	0.080162315	0.00662749	0.014171557	0.123499191
g__Brevundimonas	7.15E-06	3.82E-10	3.40E-06	3.54E-05
g__Monoglobus	0.001055814	3.61E-06	0.0003307	0.001996864
g__Eubacterium	8.31E-05	3.67E-08	3.34E-05	8.01E-06
g__Dialister	0.01052864	0.000947961	0.005359674	0.028732425
g__Enterococcus	0.001866672	4.50E-05	0.001167538	0.000168161
g__Oxalobacter	2.24E-05	1.63E-09	7.04E-06	5.44E-05
g__Luteimonas	3.58E-06	2.59E-10	2.80E-06	4.21E-07
g__Catonella	1.70E-05	2.01E-09	7.81E-06	7.16E-05
g__Hungatella	0.000401406	5.89E-07	0.000133643	0.000103257
g__TM7x	2.95E-05	6.31E-09	1.38E-05	0.000191763
g__Helcococcus	3.58E-06	2.04E-10	2.49E-06	1.05E-05
g__Lawsonella	8.94E-07	2.64E-11	8.94E-07	5.48E-06
g__Sphingobium	8.94E-07	2.64E-11	8.94E-07	5.48E-06
g__[Eubacterium]_hallii_group	0.002463865	9.85E-06	0.000546202	0.003751812
g__Johnsonella	8.05E-06	6.68E-10	4.50E-06	4.64E-05
g__Bergeyella	8.94E-07	2.64E-11	8.94E-07	5.90E-06
g__Anoxybacillus	8.94E-07	2.64E-11	8.94E-07	5.90E-06
g__Cloacibacillus	5.36E-06	1.34E-10	2.01E-06	1.26E-05

**variance. group2.**	**standard. error. group2.**	**p.value**	**q.value**	**FC**	**log10FC**
**GC.Stool-CRC.Stool-Genus**					
0	0	0.0000	0.0000	0.9756	-0.0107
2.44E-08	2.23E-05	0.0000	0.0000	1.0283	0.0121
2.99E-08	2.47E-05	0.0000	0.0000	1.0265	0.0114
2.86E-08	2.42E-05	0.0000	0.0000	1.0265	0.0114

1.14E-08	1.52E-05	0.0000	0.0000	1.0265	0.0114
2.60E-08	2.30E-05	0.0000	0.0000	1.0259	0.0111
2.10E-08	2.07E-05	0.0000	0.0000	1.0259	0.0111
2.34E-08	2.18E-05	0.0000	0.0000	1.0274	0.0117
1.95E-08	2.00E-05	0.0000	0.0000	1.0247	0.0106
8.73E-11	1.34E-06	0.0000	0.0000	0.9756	-0.0107
1.80E-08	1.92E-05	0.0000	0.0000	1.0241	0.0103
7.11E-11	1.20E-06	0.0000	0.0000	0.9776	-0.0098
2.06E-08	2.05E-05	0.0000	0.0000	1.0229	0.0098
7.37E-09	1.23E-05	0.0000	0.0000	1.0229	0.0098
2.70E-08	2.35E-05	0.0000	0.0000	1.0235	0.0101
5.93E-09	1.10E-05	0.0000	0.0000	1.0223	0.0096
8.14E-09	1.29E-05	0.0000	0.0000	1.0223	0.0096
2.30E-08	2.17E-05	0.0000	0.0000	1.0217	0.0093
2.06E-08	2.05E-05	0.0000	0.0000	1.0217	0.0093
7.28E-09	1.22E-05	0.0000	0.0000	1.0217	0.0093
5.91E-09	1.10E-05	0.0000	0.0000	1.0211	0.0091
4.98E-09	1.01E-05	0.0000	0.0000	1.0205	0.0088
7.04E-09	1.20E-05	0.0000	0.0000	1.0205	0.0088
5.38E-09	1.05E-05	0.0000	0.0000	1.0199	0.0085
3.71E-09	8.71E-06	0.0000	0.0000	1.0199	0.0085
9.95E-09	1.43E-05	0.0000	0.0000	1.0199	0.0085
6.89E-09	1.19E-05	0.0000	0.0000	1.0235	0.0101
8.73E-11	1.34E-06	0.0000	0.0000	0.9799	-0.0088
8.14E-11	1.29E-06	0.0000	0.0000	0.9777	-0.0098
1.82E-08	1.93E-05	0.0000	0.0000	1.0193	0.0083
1.82E-08	1.93E-05	0.0000	0.0000	1.0208	0.0089
4.67E-09	9.76E-06	0.0000	0.0000	1.0187	0.0080
8.44E-09	1.31E-05	0.0000	0.0000	1.0187	0.0080
8.58E-09	1.32E-05	0.0000	0.0000	1.0187	0.0080
3.98E-09	9.01E-06	0.0000	0.0000	1.0187	0.0080
1.39E-08	1.69E-05	0.0000	0.0000	1.0175	0.0075
1.29E-08	1.63E-05	0.0000	0.0000	1.0175	0.0075
8.30E-09	1.30E-05	0.0000	0.0000	1.0175	0.0075
8.73E-09	1.34E-05	0.0000	0.0000	1.0181	0.0078
1.22E-08	1.58E-05	0.0000	0.0000	1.0225	0.0097
1.53E-08	1.77E-05	0.0000	0.0000	1.0228	0.0098
5.48E-09	1.06E-05	0.0000	0.0000	1.0163	0.0070
2.06E-10	2.05E-06	0.0000	0.0000	0.9775	-0.0099
2.29E-09	6.83E-06	0.0000	0.0001	1.0157	0.0067
3.74E-09	8.74E-06	0.0000	0.0001	1.0151	0.0065
7.43E-09	1.23E-05	0.0000	0.0002	1.0166	0.0071
4.28E-09	9.35E-06	0.0000	0.0002	1.0144	0.0062
1.02E-08	1.44E-05	0.0000	0.0002	1.0144	0.0062

1.02E-08	1.44E-05	0.0000	0.0002	1.0144	0.0062
3.70E-09	8.69E-06	0.0000	0.0002	1.0144	0.0062
0	0	0.0000	0.0002	0.9885	-0.0050
4.83E-09	9.93E-06	0.0000	0.0003	1.0138	0.0060
1.90E-10	1.97E-06	0.0000	0.0004	0.9837	-0.0072
1.46E-08	1.73E-05	0.0000	0.0004	1.0207	0.0089
0	0	0.0000	0.0005	0.9894	-0.0046
7.25E-10	3.85E-06	0.0000	0.0005	0.9806	-0.0085
6.42E-09	1.14E-05	0.0000	0.0005	1.0126	0.0055
7.83E-09	1.26E-05	0.0000	0.0005	1.0126	0.0055
6.46E-09	1.15E-05	0.0000	0.0005	1.0126	0.0055
2.54E-09	7.20E-06	0.0000	0.0005	1.0132	0.0057
2.32E-09	6.89E-06	0.0000	0.0005	1.0132	0.0057
3.70E-09	8.69E-06	0.0000	0.0005	1.0132	0.0057
2.64E-09	7.35E-06	0.0000	0.0009	1.0120	0.0052
7.11E-09	1.20E-05	0.0000	0.0009	1.0120	0.0052
1.32E-10	1.64E-06	0.0001	0.0011	0.9843	-0.0069
1.44E-09	5.43E-06	0.0001	0.0014	1.0114	0.0049
2.17E-09	6.65E-06	0.0001	0.0014	1.0114	0.0049
2.10E-09	6.54E-06	0.0001	0.0014	1.0114	0.0049
1.95E-09	6.31E-06	0.0001	0.0014	1.0114	0.0049
2.13E-09	6.59E-06	0.0001	0.0024	1.0108	0.0047
1.66E-09	5.82E-06	0.0001	0.0024	1.0108	0.0047
2.06E-09	6.48E-06	0.0001	0.0024	1.0108	0.0047
1.25E-09	5.06E-06	0.0002	0.0038	1.0102	0.0044
2.01E-09	6.41E-06	0.0002	0.0038	1.0102	0.0044
1.43E-09	5.41E-06	0.0002	0.0038	1.0102	0.0044
2.27E-09	6.80E-06	0.0002	0.0038	1.0102	0.0044
4.55E-09	9.64E-06	0.0002	0.0038	1.0102	0.0044
4.01E-09	9.05E-06	0.0002	0.0038	1.0102	0.0044
2.45E-09	7.07E-06	0.0002	0.0038	1.0102	0.0044
2.20E-09	6.69E-06	0.0002	0.0038	1.0102	0.0044
1.99E-09	6.38E-06	0.0003	0.0047	1.0117	0.0051
1.72E-09	5.92E-06	0.0004	0.0066	1.0096	0.0042
2.30E-09	6.85E-06	0.0004	0.0066	1.0096	0.0042
4.00E-09	9.04E-06	0.0004	0.0066	1.0096	0.0042
1.17E-09	4.90E-06	0.0004	0.0066	1.0096	0.0042
7.35E-09	1.22E-05	0.0006	0.0100	1.0147	0.0064
3.48E-11	8.43E-07	0.0007	0.0104	0.9906	-0.0041
0	0	0.0007	0.0109	0.9929	-0.0031
2.62E-09	7.31E-06	0.0007	0.0111	1.0090	0.0039
3.02E-09	7.85E-06	0.0007	0.0111	1.0090	0.0039
2.04E-09	6.45E-06	0.0007	0.0111	1.0090	0.0039
1.35E-09	5.25E-06	0.0007	0.0111	1.0090	0.0039

9.79E-10	4.47E-06	0.0007	0.0111	0.9838	-0.0071
1.01E-07	4.55E-05	0.0010	0.0131	1.1195	0.0490
2.03E-07	6.43E-05	0.0010	0.0131	0.2953	-0.5298
0	0	0.0010	0.0131	0.9705	-0.0130
1.47E-07	5.48E-05	0.0010	0.0131	1.0819	0.0342
1.76E-08	1.89E-05	0.0010	0.0131	1.0415	0.0177
1.04E-06	0.000145638	0.0010	0.0131	1.1788	0.0714
5.46E-07	0.000105528	0.0010	0.0131	1.1397	0.0568
4.55E-07	9.63E-05	0.0010	0.0131	1.0963	0.0399
1.13E-08	1.52E-05	0.0010	0.0131	1.0343	0.0147
1.29E-07	5.12E-05	0.0010	0.0131	1.0662	0.0279
9.71E-08	4.45E-05	0.0010	0.0131	1.0512	0.0217
9.87E-08	4.49E-05	0.0010	0.0131	1.0638	0.0269
8.29E-08	4.11E-05	0.0010	0.0131	1.0548	0.0232
1.21E-07	4.96E-05	0.0010	0.0131	1.1144	0.0470
1.84E-09	6.13E-06	0.0014	0.0169	1.0078	0.0034
1.04E-09	4.61E-06	0.0014	0.0169	1.0078	0.0034
7.90E-10	4.01E-06	0.0014	0.0169	1.0078	0.0034
1.92E-09	6.26E-06	0.0014	0.0169	1.0084	0.0036
1.27E-09	5.09E-06	0.0014	0.0169	1.0084	0.0036
2.21E-09	6.72E-06	0.0014	0.0169	1.0084	0.0036
1.31E-09	5.16E-06	0.0014	0.0169	1.0084	0.0036
9.07E-10	4.30E-06	0.0014	0.0169	1.0084	0.0036
6.66E-11	1.17E-06	0.0015	0.0177	0.9900	-0.0044
0	0	0.0017	0.0203	0.9938	-0.0027
2.51E-07	7.16E-05	0.0020	0.0231	1.1355	0.0552
1.20E-08	1.57E-05	0.0020	0.0231	1.0307	0.0131
5.83E-07	0.000109045	0.0020	0.0231	1.2325	0.0908
3.15E-10	2.53E-06	0.0021	0.0241	0.9878	-0.0053
1.07E-09	4.68E-06	0.0024	0.0256	1.0072	0.0031
2.16E-09	6.64E-06	0.0024	0.0256	1.0072	0.0031
1.29E-09	5.13E-06	0.0024	0.0256	1.0072	0.0031
1.29E-09	5.13E-06	0.0024	0.0256	1.0072	0.0031
1.58E-09	5.68E-06	0.0024	0.0256	1.0072	0.0031
9.98E-10	4.51E-06	0.0024	0.0256	1.0072	0.0031
2.16E-09	6.64E-06	0.0024	0.0256	1.0072	0.0031
2.16E-09	6.64E-06	0.0024	0.0256	1.0072	0.0031
1.29E-09	5.13E-06	0.0024	0.0256	1.0072	0.0031
1.29E-09	5.13E-06	0.0024	0.0256	1.0072	0.0031
3.48E-11	8.43E-07	0.0026	0.0278	0.9915	-0.0037
9.15E-06	0.000432065	0.0030	0.0309	1.5193	0.1817
1.53E-07	5.60E-05	0.0030	0.0309	1.0747	0.0313
2.85E-08	2.41E-05	0.0030	0.0309	1.0470	0.0199
6.45E-10	3.63E-06	0.0037	0.0381	0.9867	-0.0058

8.87E-09	1.35E-05	0.0039	0.0399	1.0141	0.0061
5.57E-05	0.001066285	0.0040	0.0401	2.1090	0.3241
4.40E-05	0.000947349	0.0040	0.0401	2.1445	0.3313
1.28E-09	5.11E-06	0.0042	0.0417	1.0066	0.0029
1.17E-09	4.89E-06	0.0042	0.0417	1.0066	0.0029
7.35E-10	3.87E-06	0.0042	0.0417	1.0066	0.0029
5.49E-07	0.000105804	0.0050	0.0447	1.2574	0.0995
9.69E-08	4.45E-05	0.0050	0.0447	1.0903	0.0376
5.76E-08	3.43E-05	0.0050	0.0447	1.0662	0.0279
1.48E-08	1.74E-05	0.0050	0.0447	1.0361	0.0154
3.91E-07	8.94E-05	0.0050	0.0447	1.1523	0.0616
1.93E-08	1.98E-05	0.0050	0.0447	1.0427	0.0182
5.54E-08	3.36E-05	0.0050	0.0447	1.0578	0.0244
5.16E-07	0.000102595	0.0050	0.0447	1.1258	0.0515
7.77E-08	3.98E-05	0.0050	0.0447	1.0897	0.0373
9.16E-09	1.37E-05	0.0050	0.0447	1.0301	0.0129
2.06E-07	6.49E-05	0.0050	0.0447	1.1048	0.0433
2.00E-07	6.39E-05	0.0050	0.0447	1.1313	0.0536
5.82E-08	3.45E-05	0.0050	0.0447	1.0731	0.0306
1.26E-07	5.07E-05	0.0050	0.0447	1.1042	0.0430
4.35E-10	2.98E-06	0.0052	0.0464	0.9901	-0.0043
0.000657446	0.003662959	0.0060	0.0524	2.8209	0.4504
4.89E-08	3.16E-05	0.0060	0.0524	1.0440	0.0187
3.34E-08	2.61E-05	0.0060	0.0524	1.0433	0.0184
9.43E-10	4.39E-06	0.0070	0.0589	1.0075	0.0033
1.69E-06	0.000185679	0.0070	0.0589	0.0949	-1.0226
1.99E-07	6.37E-05	0.0070	0.0589	1.0950	0.0394
4.90E-07	1.00E-04	0.0070	0.0589	1.2216	0.0869
1.67E-07	5.84E-05	0.0070	0.0589	1.0701	0.0294
3.06E-08	2.50E-05	0.0070	0.0589	1.0548	0.0232
4.34E-10	2.98E-06	0.0076	0.0613	1.0060	0.0026
1.01E-09	4.55E-06	0.0076	0.0613	1.0060	0.0026
7.97E-10	4.03E-06	0.0076	0.0613	1.0060	0.0026
7.25E-10	3.85E-06	0.0076	0.0613	1.0060	0.0026
1.78E-09	6.02E-06	0.0076	0.0613	1.0060	0.0026
1.16E-09	4.87E-06	0.0076	0.0613	1.0060	0.0026
8.70E-10	4.21E-06	0.0076	0.0613	1.0060	0.0026
2.57E-09	7.24E-06	0.0080	0.0636	0.7953	-0.0995
2.03E-07	6.44E-05	0.0080	0.0636	1.0785	0.0328
4.27E-08	2.95E-05	0.0080	0.0636	1.0506	0.0214
7.31E-05	0.001221569	0.0090	0.0711	1.9525	0.2906
0	0	0.0106	0.0817	0.9955	-0.0019
0	0	0.0106	0.0817	0.9955	-0.0019
0	0	0.0106	0.0817	0.9955	-0.0019

0	0	0.0106	0.0817	0.9955	-0.0019
1.14E-09	4.82E-06	0.0113	0.0871	0.9906	-0.0041
6.19E-08	3.55E-05	0.0120	0.0913	1.0686	0.0288
1.11E-08	1.50E-05	0.0120	0.0913	1.0313	0.0134
5.44E-10	3.33E-06	0.0130	0.0978	0.9093	-0.0413
4.81E-09	9.91E-06	0.0134	0.0980	1.0114	0.0049
3.15E-10	2.53E-06	0.0137	0.0980	1.0054	0.0023
6.77E-10	3.72E-06	0.0137	0.0980	1.0054	0.0023
3.87E-10	2.81E-06	0.0137	0.0980	1.0054	0.0023
3.15E-10	2.53E-06	0.0137	0.0980	1.0054	0.0023
1.44E-09	5.42E-06	0.0137	0.0980	1.0054	0.0023
1.44E-09	5.42E-06	0.0137	0.0980	1.0054	0.0023
1.15E-09	4.84E-06	0.0137	0.0980	1.0054	0.0023
5.32E-10	3.30E-06	0.0137	0.0980	1.0054	0.0023
4.96E-10	3.18E-06	0.0137	0.0980	1.0054	0.0023
1.26E-09	5.07E-06	0.0142	0.1012	1.0081	0.0035
8.24E-05	0.001297035	0.0150	0.1056	2.3400	0.3692
8.82E-08	4.24E-05	0.0150	0.1056	1.0675	0.0284
1.35E-08	1.66E-05	0.0160	0.1116	0.9264	-0.0332
2.64E-07	7.34E-05	0.0180	0.1249	0.5471	-0.2620
1.78E-11	6.02E-07	0.0195	0.1347	0.9953	-0.0021
2.99E-08	2.47E-05	0.0200	0.1368	1.0355	0.0152
7.07E-09	1.20E-05	0.0200	0.1368	0.9289	-0.0320
2.75E-09	7.50E-06	0.0207	0.1409	1.0084	0.0036
5.98E-08	3.49E-05	0.0210	0.1409	1.0391	0.0167
9.20E-08	4.33E-05	0.0210	0.1409	1.0470	0.0199
2.46E-09	7.09E-06	0.0210	0.1409	1.0198	0.0085
2.22E-07	6.73E-05	0.0220	0.1425	1.1174	0.0482
8.70E-07	0.000133252	0.0220	0.1425	1.2198	0.0863
4.45E-08	3.01E-05	0.0220	0.1425	1.0343	0.0147
4.14E-08	2.91E-05	0.0220	0.1425	1.0409	0.0174
2.93E-08	2.45E-05	0.0220	0.1425	1.0319	0.0136
1.69E-08	1.85E-05	0.0220	0.1425	1.0349	0.0149
4.48E-10	3.02E-06	0.0220	0.1425	0.9577	-0.0188
6.00E-10	3.50E-06	0.0220	0.1425	1.0075	0.0033
6.93E-08	3.76E-05	0.0230	0.1481	1.0733	0.0307
4.09E-09	9.13E-06	0.0235	0.1508	1.0105	0.0046
1.03E-10	1.45E-06	0.0240	0.1522	0.9686	-0.0138
1.01E-08	1.44E-05	0.0240	0.1522	0.8641	-0.0634
3.67E-08	2.74E-05	0.0250	0.1522	1.0622	0.0262
3.03E-10	2.49E-06	0.0251	0.1522	1.0048	0.0021
3.75E-10	2.77E-06	0.0251	0.1522	1.0048	0.0021
5.20E-10	3.26E-06	0.0251	0.1522	1.0048	0.0021
6.65E-10	3.68E-06	0.0251	0.1522	1.0048	0.0021

4.84E-10	3.14E-06	0.0251	0.1522	1.0048	0.0021
8.83E-10	4.24E-06	0.0251	0.1522	1.0048	0.0021
8.83E-10	4.24E-06	0.0251	0.1522	1.0048	0.0021
8.83E-10	4.24E-06	0.0251	0.1522	1.0048	0.0021
4.84E-10	3.14E-06	0.0251	0.1522	1.0048	0.0021
5.93E-10	3.48E-06	0.0251	0.1522	1.0048	0.0021
4.16E-08	2.91E-05	0.0260	0.1563	0.8500	-0.0706
1.75E-10	1.89E-06	0.0261	0.1563	0.9935	-0.0028
0	0	0.0262	0.1563	0.9964	-0.0016
0	0	0.0262	0.1563	0.9964	-0.0016
4.02E-09	9.06E-06	0.0270	0.1600	0.9070	-0.0424
1.77E-07	6.00E-05	0.0280	0.1646	1.0963	0.0399
5.56E-08	3.37E-05	0.0280	0.1646	1.0632	0.0266
3.05E-09	7.89E-06	0.0290	0.1691	0.9070	-0.0424
1.27E-08	1.61E-05	0.0290	0.1691	1.0290	0.0124
1.16E-05	0.000485575	0.0300	0.1742	0.4640	-0.3335
4.17E-08	2.92E-05	0.0310	0.1793	1.0470	0.0200
2.62E-09	7.32E-06	0.0330	0.1901	0.9429	-0.0255
1.13E-09	4.81E-06	0.0334	0.1915	0.9916	-0.0037
2.02E-07	6.43E-05	0.0340	0.1942	1.1073	0.0442
1.23E-07	5.01E-05	0.0380	0.2162	1.0979	0.0405
3.47E-09	8.42E-06	0.0383	0.2174	1.0108	0.0047
2.20E-07	6.69E-05	0.0390	0.2202	0.7391	-0.1313
6.36E-10	3.60E-06	0.0400	0.2249	0.9511	-0.0218
4.96E-08	3.18E-05	0.0420	0.2342	1.0664	0.0279
1.78E-11	6.02E-07	0.0421	0.2342	0.9961	-0.0017
2.76E-08	2.37E-05	0.0430	0.2371	1.0418	0.0178
1.83E-07	6.10E-05	0.0430	0.2371	0.7961	-0.0990
6.82E-08	3.73E-05	0.0440	0.2407	0.7851	-0.1051
1.75E-10	1.89E-06	0.0455	0.2435	0.9944	-0.0024
3.47E-09	8.41E-06	0.0458	0.2435	1.0114	0.0049
4.71E-10	3.10E-06	0.0467	0.2435	1.0042	0.0018
4.35E-10	2.98E-06	0.0467	0.2435	1.0042	0.0018
2.54E-10	2.28E-06	0.0467	0.2435	1.0042	0.0018
8.70E-10	4.21E-06	0.0467	0.2435	1.0042	0.0018
2.54E-10	2.28E-06	0.0467	0.2435	1.0042	0.0018
8.70E-10	4.21E-06	0.0467	0.2435	1.0042	0.0018
4.35E-10	2.98E-06	0.0467	0.2435	1.0042	0.0018
8.70E-10	4.21E-06	0.0467	0.2435	1.0042	0.0018
3.63E-10	2.72E-06	0.0467	0.2435	1.0042	0.0018
2.54E-10	2.28E-06	0.0467	0.2435	1.0042	0.0018
4.35E-10	2.98E-06	0.0467	0.2435	1.0042	0.0018
1.61E-07	5.73E-05	0.0470	0.2440	1.0690	0.0290
2.05E-08	2.05E-05	0.0500	0.2587	1.0313	0.0134

GC.Stool-N.Stool-Genus					
0	0	0.0000	0.0000	0.9725	-0.0121
0	0	0.0000	0.0000	0.9725	-0.0121
0	0	0.0000	0.0000	0.9725	-0.0121
0	0	0.0000	0.0000	0.9742	-0.0114
0	0	0.0000	0.0000	0.9742	-0.0114
0	0	0.0000	0.0000	0.9742	-0.0114
0	0	0.0000	0.0000	0.9742	-0.0114
0	0	0.0000	0.0000	0.9748	-0.0111
0	0	0.0000	0.0000	0.9748	-0.0111
0	0	0.0000	0.0000	0.9753	-0.0108
0	0	0.0000	0.0000	0.9759	-0.0106
0	0	0.0000	0.0000	0.9771	-0.0101
0	0	0.0000	0.0000	0.9776	-0.0098
0	0	0.0000	0.0000	0.9776	-0.0098
0	0	0.0000	0.0000	0.9782	-0.0096
2.09E-08	1.73E-05	0.0000	0.0000	1.0285	0.0122
0	0	0.0000	0.0000	0.9788	-0.0093
0	0	0.0000	0.0000	0.9788	-0.0093
0	0	0.0000	0.0000	0.9788	-0.0093
0	0	0.0000	0.0000	0.9794	-0.0091
0	0	0.0000	0.0000	0.9799	-0.0088
1.24E-11	4.21E-07	0.0000	0.0000	0.9786	-0.0094
0	0	0.0000	0.0000	0.9805	-0.0085
0	0	0.0000	0.0000	0.9805	-0.0085
0	0	0.0000	0.0000	0.9805	-0.0085
0	0	0.0000	0.0000	0.9811	-0.0083
0	0	0.0000	0.0000	0.9811	-0.0083
0	0	0.0000	0.0000	0.9811	-0.0083
0	0	0.0000	0.0000	0.9811	-0.0083
0	0	0.0000	0.0000	0.9817	-0.0080
0	0	0.0000	0.0000	0.9817	-0.0080
0	0	0.0000	0.0000	0.9817	-0.0080
0	0	0.0000	0.0000	0.9823	-0.0078
1.05E-08	1.22E-05	0.0000	0.0000	1.0211	0.0091
0	0	0.0000	0.0000	0.9828	-0.0075
0	0	0.0000	0.0000	0.9828	-0.0075
0	0	0.0000	0.0000	0.9828	-0.0075
2.39E-09	5.85E-06	0.0000	0.0000	1.0239	0.0102
0	0	0.0000	0.0000	0.9840	-0.0070
1.30E-10	1.36E-06	0.0000	0.0000	0.9782	-0.0096
0	0	0.0000	0.0000	0.9852	-0.0065
0	0	0.0000	0.0000	0.9852	-0.0065
0	0	0.0000	0.0000	0.9858	-0.0062

0	0	0.0000	0.0000	0.9858	-0.0062
0	0	0.0000	0.0000	0.9858	-0.0062
0	0	0.0000	0.0000	0.9858	-0.0062
3.62E-11	7.19E-07	0.0000	0.0000	0.9823	-0.0077
2.31E-10	1.82E-06	0.0000	0.0000	0.9794	-0.0091
0	0	0.0000	0.0000	0.9863	-0.0060
0	0	0.0000	0.0000	0.9869	-0.0057
0	0	0.0000	0.0000	0.9869	-0.0057
0	0	0.0000	0.0000	0.9869	-0.0057
1.24E-09	4.21E-06	0.0000	0.0000	0.9794	-0.0090
1.99E-10	1.69E-06	0.0000	0.0000	0.9833	-0.0073
0	0	0.0000	0.0000	0.9875	-0.0055
0	0	0.0000	0.0000	0.9875	-0.0055
0	0	0.0000	0.0000	0.9875	-0.0055
0	0	0.0000	0.0000	0.9881	-0.0052
0	0	0.0000	0.0000	0.9881	-0.0052
0	0	0.0000	0.0000	0.9881	-0.0052
0	0	0.0000	0.0000	0.9887	-0.0049
0	0	0.0000	0.0000	0.9887	-0.0049
0	0	0.0000	0.0000	0.9887	-0.0049
0	0	0.0000	0.0000	0.9887	-0.0049
1.12E-10	1.26E-06	0.0000	0.0000	0.9858	-0.0062
1.24E-11	4.21E-07	0.0000	0.0000	0.9879	-0.0053
0	0	0.0000	0.0000	0.9893	-0.0047
0	0	0.0000	0.0000	0.9893	-0.0047
0	0	0.0000	0.0000	0.9893	-0.0047
0	0	0.0000	0.0000	0.9893	-0.0047
0	0	0.0000	0.0000	0.9893	-0.0047
0	0	0.0000	0.0000	0.9899	-0.0044
0	0	0.0000	0.0000	0.9899	-0.0044
0	0	0.0000	0.0000	0.9899	-0.0044
0	0	0.0000	0.0000	0.9899	-0.0044
0	0	0.0000	0.0000	0.9899	-0.0044
0	0	0.0000	0.0000	0.9899	-0.0044
0	0	0.0000	0.0000	0.9899	-0.0044
0	0	0.0000	0.0000	0.9905	-0.0042
0	0	0.0000	0.0000	0.9905	-0.0042
0	0	0.0000	0.0000	0.9905	-0.0042
0	0	0.0000	0.0000	0.9905	-0.0042
0	0	0.0000	0.0000	0.9905	-0.0042
0	0	0.0000	0.0000	0.9910	-0.0039
0	0	0.0000	0.0000	0.9910	-0.0039
0	0	0.0000	0.0000	0.9910	-0.0039
0	0	0.0000	0.0000	0.9910	-0.0039

4.97E-11	8.43E-07	0.0000	0.0000	0.9889	-0.0048
1.24E-11	4.21E-07	0.0000	0.0000	0.9903	-0.0042
7.77E-09	1.05E-05	0.0000	0.0001	1.0105	0.0046
0	0	0.0000	0.0001	0.9916	-0.0036
0	0	0.0000	0.0001	0.9916	-0.0036
0	0	0.0000	0.0001	0.9916	-0.0036
0	0	0.0000	0.0001	0.9916	-0.0036
5.15E-09	8.58E-06	0.0000	0.0001	1.0153	0.0066
3.22E-09	6.78E-06	0.0000	0.0001	1.0140	0.0060
0	0	0.0000	0.0001	0.9922	-0.0034
0	0	0.0000	0.0001	0.9922	-0.0034
0	0	0.0000	0.0001	0.9922	-0.0034
0	0	0.0000	0.0001	0.9922	-0.0034
0	0	0.0000	0.0001	0.9922	-0.0034
2.00E-08	1.69E-05	0.0000	0.0002	1.0153	0.0066
2.62E-09	6.11E-06	0.0000	0.0002	1.0132	0.0057
1.80E-09	5.07E-06	0.0000	0.0003	0.9853	-0.0064
1.30E-09	4.32E-06	0.0000	0.0003	0.9839	-0.0071
0	0	0.0000	0.0003	0.9928	-0.0031
0	0	0.0000	0.0003	0.9928	-0.0031
0	0	0.0000	0.0003	0.9928	-0.0031
0	0	0.0000	0.0003	0.9928	-0.0031
0	0	0.0000	0.0003	0.9928	-0.0031
0	0	0.0000	0.0003	0.9928	-0.0031
0	0	0.0000	0.0003	0.9928	-0.0031
0	0	0.0000	0.0003	0.9928	-0.0031
1.24E-11	4.21E-07	0.0000	0.0005	0.9921	-0.0035
1.86E-09	5.15E-06	0.0000	0.0005	1.0117	0.0050
0	0	0.0001	0.0007	0.9934	-0.0029
0	0	0.0001	0.0007	0.9934	-0.0029
0	0	0.0001	0.0007	0.9934	-0.0029
1.99E-10	1.69E-06	0.0001	0.0007	0.9904	-0.0042
2.34E-10	1.83E-06	0.0001	0.0011	0.9871	-0.0057
0	0	0.0001	0.0015	0.9940	-0.0026
0	0	0.0001	0.0015	0.9940	-0.0026
0	0	0.0001	0.0015	0.9940	-0.0026
0	0	0.0001	0.0015	0.9940	-0.0026
0	0	0.0001	0.0015	0.9940	-0.0026
0	0	0.0001	0.0015	0.9940	-0.0026
0	0	0.0001	0.0015	0.9940	-0.0026
2.45E-11	5.92E-07	0.0002	0.0020	0.9925	-0.0033
1.24E-11	4.21E-07	0.0002	0.0020	0.9932	-0.0029
1.24E-11	4.21E-07	0.0002	0.0020	0.9932	-0.0029
3.76E-10	2.32E-06	0.0003	0.0033	0.9866	-0.0058

0	0	0.0003	0.0034	0.9946	-0.0023
0	0	0.0003	0.0034	0.9946	-0.0023
0	0	0.0003	0.0034	0.9946	-0.0023
0	0	0.0003	0.0034	0.9946	-0.0023
0	0	0.0003	0.0034	0.9946	-0.0023
0	0	0.0003	0.0034	0.9946	-0.0023
0	0	0.0003	0.0034	0.9946	-0.0023
0	0	0.0003	0.0034	0.9946	-0.0023
0	0	0.0003	0.0034	0.9946	-0.0023
0	0	0.0003	0.0034	0.9946	-0.0023
1.28E-09	4.28E-06	0.0004	0.0038	1.0063	0.0027
1.24E-11	4.21E-07	0.0004	0.0042	0.9938	-0.0027
7.30E-10	3.23E-06	0.0004	0.0043	1.0074	0.0032
1.07E-09	3.92E-06	0.0006	0.0054	0.9886	-0.0050
0	0	0.0008	0.0062	0.9952	-0.0021
0	0	0.0008	0.0062	0.9952	-0.0021
0	0	0.0008	0.0062	0.9952	-0.0021
0	0	0.0008	0.0062	0.9952	-0.0021
0	0	0.0008	0.0062	0.9952	-0.0021
0	0	0.0008	0.0062	0.9952	-0.0021
0	0	0.0008	0.0062	0.9952	-0.0021
0	0	0.0008	0.0062	0.9952	-0.0021
0	0	0.0008	0.0062	0.9952	-0.0021
0	0	0.0008	0.0062	0.9952	-0.0021
0	0	0.0008	0.0062	0.9952	-0.0021
0	0	0.0008	0.0062	0.9952	-0.0021
0	0	0.0008	0.0062	0.9952	-0.0021
0	0	0.0008	0.0062	0.9952	-0.0021
0	0	0.0008	0.0062	0.9952	-0.0021
1.24E-11	4.21E-07	0.0010	0.0062	0.9944	-0.0024
0.004658213	0.008157567	0.0010	0.0062	6.8334	0.8346
9.64E-06	0.000371096	0.0010	0.0062	0.1028	-0.9878
0.000241987	0.001859291	0.0010	0.0062	3.1896	0.5037
9.56E-05	0.001168611	0.0010	0.0062	1.9018	0.2792
0	0	0.0010	0.0062	0.9172	-0.0376
1.90E-08	1.65E-05	0.0010	0.0062	0.7943	-0.1000
0	0	0.0010	0.0062	0.9172	-0.0376
0	0	0.0010	0.0062	0.8807	-0.0552
0	0	0.0010	0.0062	0.9379	-0.0279
1.51E-06	0.000146997	0.0010	0.0062	1.1918	0.0762
0	0	0.0010	0.0062	0.8150	-0.0888
0	0	0.0010	0.0062	0.9384	-0.0276
2.71E-06	0.000196681	0.0010	0.0062	1.2243	0.0879
3.21E-10	2.14E-06	0.0010	0.0062	0.7077	-0.1502

0	0	0.0010	0.0062	0.9486	-0.0229
0	0	0.0010	0.0062	0.9243	-0.0342
0	0	0.0010	0.0062	0.9651	-0.0154
0	0	0.0010	0.0062	0.8920	-0.0496
0	0	0.0010	0.0062	0.9629	-0.0164
0	0	0.0010	0.0062	0.8063	-0.0935
0	0	0.0010	0.0062	0.8678	-0.0616
0	0	0.0010	0.0062	0.9601	-0.0177
0	0	0.0010	0.0062	0.9305	-0.0313
0	0	0.0010	0.0062	0.8483	-0.0714
0	0	0.0010	0.0062	0.9590	-0.0182
0	0	0.0010	0.0062	0.9679	-0.0141
0	0	0.0010	0.0062	0.8949	-0.0482
0	0	0.0010	0.0062	0.8973	-0.0470
0	0	0.0010	0.0062	0.9454	-0.0244
0	0	0.0010	0.0062	0.8198	-0.0863
0	0	0.0010	0.0062	0.8774	-0.0568
0	0	0.0010	0.0062	0.8389	-0.0763
0	0	0.0010	0.0062	0.8882	-0.0515
3.99E-09	7.55E-06	0.0010	0.0062	0.9154	-0.0384
0	0	0.0010	0.0062	0.9551	-0.0199
0	0	0.0010	0.0062	0.9358	-0.0288
1.24E-11	4.21E-07	0.0010	0.0062	0.9314	-0.0308
0	0	0.0010	0.0062	0.9708	-0.0129
0	0	0.0010	0.0062	0.8186	-0.0869
0	0	0.0010	0.0062	0.9228	-0.0349
0	0	0.0010	0.0062	0.8840	-0.0536
0	0	0.0010	0.0062	0.9121	-0.0399
0	0	0.0010	0.0062	0.9379	-0.0279
0	0	0.0010	0.0062	0.9513	-0.0217
0	0	0.0010	0.0062	0.9400	-0.0269
0	0	0.0010	0.0062	0.9551	-0.0199
0	0	0.0010	0.0062	0.9311	-0.0310
0	0	0.0010	0.0062	0.9057	-0.0430
0	0	0.0010	0.0062	0.9481	-0.0232
0	0	0.0010	0.0062	0.8973	-0.0470
0	0	0.0010	0.0062	0.9579	-0.0187
0	0	0.0010	0.0062	0.9601	-0.0177
0	0	0.0010	0.0062	0.9668	-0.0147
0	0	0.0010	0.0062	0.9708	-0.0129
0	0	0.0010	0.0062	0.9607	-0.0174
0	0	0.0010	0.0062	0.9519	-0.0214
0	0	0.0010	0.0062	0.9708	-0.0129
0	0	0.0010	0.0062	0.9405	-0.0266

0	0	0.0010	0.0062	0.9691	-0.0136
0	0	0.0010	0.0062	0.9585	-0.0184
0	0	0.0010	0.0062	0.9696	-0.0134
0	0	0.0010	0.0062	0.9696	-0.0134
0	0	0.0010	0.0062	0.9481	-0.0232
0	0	0.0010	0.0062	0.9663	-0.0149
6.40E-10	3.02E-06	0.0016	0.0097	0.9880	-0.0052
0.000144198	0.00143526	0.0020	0.0112	0.2293	-0.6396
8.80E-07	0.000112105	0.0020	0.0112	1.3930	0.1440
1.17E-07	4.09E-05	0.0020	0.0112	0.6720	-0.1726
1.38E-06	0.000140657	0.0020	0.0112	0.5812	-0.2357
9.87E-06	0.00037555	0.0020	0.0112	1.6016	0.2046
1.93E-08	1.66E-05	0.0020	0.0112	1.0354	0.0151
1.24E-11	4.21E-07	0.0020	0.0112	0.9181	-0.0371
2.43E-07	5.89E-05	0.0020	0.0112	1.0999	0.0413
6.54E-10	3.06E-06	0.0020	0.0112	0.9312	-0.0310
2.54E-08	1.90E-05	0.0020	0.0112	1.0289	0.0124
0	0	0.0020	0.0112	0.9958	-0.0018
0	0	0.0020	0.0112	0.9958	-0.0018
0	0	0.0020	0.0112	0.9958	-0.0018
0	0	0.0020	0.0112	0.9958	-0.0018
0	0	0.0020	0.0112	0.9958	-0.0018
0	0	0.0020	0.0112	0.9958	-0.0018
0	0	0.0020	0.0112	0.9958	-0.0018
0	0	0.0020	0.0112	0.9958	-0.0018
0	0	0.0020	0.0112	0.9958	-0.0018
0	0	0.0020	0.0112	0.9958	-0.0018
0	0	0.0020	0.0112	0.9958	-0.0018
6.35E-10	3.01E-06	0.0022	0.0122	1.0061	0.0027
6.60E-09	9.71E-06	0.0022	0.0124	1.0139	0.0060
3.27E-08	2.16E-05	0.0040	0.0217	1.0562	0.0238
9.15E-07	0.000114302	0.0040	0.0217	1.2422	0.0942
1.32E-05	0.000433508	0.0040	0.0217	1.7901	0.2529
5.29E-07	8.69E-05	0.0040	0.0217	1.2249	0.0881
1.24E-11	4.21E-07	0.0040	0.0217	0.9544	-0.0202
1.24E-11	4.21E-07	0.0047	0.0244	0.9956	-0.0019
0	0	0.0049	0.0244	0.9964	-0.0016
0	0	0.0049	0.0244	0.9964	-0.0016
0	0	0.0049	0.0244	0.9964	-0.0016
0	0	0.0049	0.0244	0.9964	-0.0016
0	0	0.0049	0.0244	0.9964	-0.0016
0	0	0.0049	0.0244	0.9964	-0.0016
0	0	0.0049	0.0244	0.9964	-0.0016
0	0	0.0049	0.0244	0.9964	-0.0016

0	0	0.0049	0.0244	0.9964	-0.0016
0	0	0.0049	0.0244	0.9964	-0.0016
0	0	0.0049	0.0244	0.9964	-0.0016
0	0	0.0049	0.0244	0.9964	-0.0016
0	0	0.0049	0.0244	0.9964	-0.0016
0	0	0.0049	0.0244	0.9964	-0.0016
0	0	0.0049	0.0244	0.9964	-0.0016
0	0	0.0049	0.0244	0.9964	-0.0016
0	0	0.0049	0.0244	0.9964	-0.0016
0	0	0.0049	0.0244	0.9964	-0.0016
0	0	0.0049	0.0244	0.9964	-0.0016
0	0	0.0049	0.0244	0.9964	-0.0016
0	0	0.0049	0.0244	0.9964	-0.0016
0	0	0.0049	0.0244	0.9964	-0.0016
0.01087793	0.012465914	0.0050	0.0248	1.6213	0.2099
1.24E-11	4.21E-07	0.0050	0.0248	0.9718	-0.0124
5.70E-08	2.85E-05	0.0060	0.0294	1.0726	0.0304
3.85E-08	2.34E-05	0.0060	0.0294	1.0692	0.0291
2.12E-08	1.74E-05	0.0060	0.0294	0.8590	-0.0660
3.76E-10	2.32E-06	0.0062	0.0301	0.9924	-0.0033
4.21E-07	7.75E-05	0.0080	0.0384	1.0953	0.0395
1.24E-11	4.21E-07	0.0080	0.0384	0.9268	-0.0330
6.12E-07	9.35E-05	0.0080	0.0384	1.2162	0.0850
2.99E-08	2.07E-05	0.0080	0.0384	1.0570	0.0241
3.83E-10	2.34E-06	0.0081	0.0388	1.0055	0.0024
1.00E-05	0.000378332	0.0100	0.0471	1.9555	0.2913
2.66E-07	6.17E-05	0.0100	0.0471	0.5098	-0.2926
1.24E-11	4.21E-07	0.0100	0.0471	0.9706	-0.0130
7.10E-07	0.000100733	0.0100	0.0471	1.2246	0.0880
3.46E-08	2.22E-05	0.0100	0.0471	1.0569	0.0240
**CRC.Stool-N.Stool-Genus**					
0	0	0.0000	0.0000	0.9739	-0.0115
0	0	0.0000	0.0000	0.9747	-0.0111
0	0	0.0000	0.0000	0.9756	-0.0107
0	0	0.0000	0.0000	0.9756	-0.0107
1.24E-11	4.21E-07	0.0000	0.0000	0.9743	-0.0113
0	0	0.0000	0.0000	0.9781	-0.0096
2.45E-11	5.92E-07	0.0000	0.0000	0.9756	-0.0108
0	0	0.0000	0.0000	0.9790	-0.0092
2.09E-08	1.73E-05	0.0000	0.0000	1.0291	0.0124
0	0	0.0000	0.0000	0.9807	-0.0085
0	0	0.0000	0.0000	0.9850	-0.0066
0	0	0.0000	0.0000	0.9850	-0.0066
1.05E-08	1.22E-05	0.0000	0.0000	1.0211	0.0091

0	0	0.0000	0.0000	0.9859	-0.0062
0	0	0.0000	0.0000	0.9859	-0.0062
0	0	0.0000	0.0000	0.9876	-0.0054
2.00E-08	1.69E-05	0.0000	0.0000	1.0193	0.0083
3.22E-09	6.78E-06	0.0000	0.0000	1.0164	0.0071
0	0	0.0000	0.0000	0.9885	-0.0050
0	0	0.0000	0.0000	0.9885	-0.0050
1.24E-11	4.21E-07	0.0000	0.0000	0.9872	-0.0056
2.39E-09	5.85E-06	0.0000	0.0000	1.0221	0.0095
2.62E-09	6.11E-06	0.0000	0.0001	1.0156	0.0067
0	0	0.0000	0.0001	0.9894	-0.0046
0	0	0.0000	0.0001	0.9894	-0.0046
2.41E-09	5.87E-06	0.0000	0.0004	1.0131	0.0056
6.60E-09	9.71E-06	0.0000	0.0005	1.0207	0.0089
3.76E-10	2.32E-06	0.0000	0.0008	0.9844	-0.0068
0	0	0.0000	0.0017	0.9920	-0.0035
2.88E-09	6.42E-06	0.0000	0.0020	1.0170	0.0073
1.07E-09	3.92E-06	0.0000	0.0020	0.9839	-0.0070
3.83E-10	2.34E-06	0.0000	0.0020	0.9830	-0.0074
2.11E-08	1.73E-05	0.0000	0.0021	1.0174	0.0075
2.60E-09	6.10E-06	0.0001	0.0026	1.0110	0.0047
1.24E-11	4.21E-07	0.0001	0.0034	0.9916	-0.0037
2.46E-09	5.93E-06	0.0001	0.0034	1.0122	0.0052
1.30E-09	4.31E-06	0.0001	0.0034	1.0122	0.0052
3.00E-09	6.55E-06	0.0001	0.0037	1.0158	0.0068
0	0	0.0001	0.0039	0.9929	-0.0031
1.36E-09	4.41E-06	0.0001	0.0039	1.0105	0.0046
2.25E-09	5.66E-06	0.0002	0.0061	0.9824	-0.0077
4.68E-09	8.18E-06	0.0002	0.0081	1.0113	0.0049
1.24E-11	4.21E-07	0.0003	0.0082	0.9924	-0.0033
1.85E-08	1.63E-05	0.0003	0.0092	1.0175	0.0075
2.33E-09	5.78E-06	0.0003	0.0097	1.0093	0.0040
0	0	0.0003	0.0103	0.9938	-0.0027
1.97E-09	5.31E-06	0.0005	0.0155	1.0084	0.0036
1.24E-11	4.21E-07	0.0007	0.0165	0.9933	-0.0029
5.08E-09	8.52E-06	0.0009	0.0165	1.0151	0.0065
0.004658213	0.008157567	0.0010	0.0165	7.0796	0.8500
8.80E-07	0.000112105	0.0010	0.0165	1.4276	0.1546
6.16E-07	9.38E-05	0.0010	0.0165	1.3145	0.1188
0.001859443	0.005153976	0.0010	0.0165	5.2491	0.7201
7.41E-08	3.25E-05	0.0010	0.0165	1.1329	0.0542
1.79E-07	5.06E-05	0.0010	0.0165	1.1669	0.0670
0.000146024	0.001444318	0.0010	0.0165	3.2834	0.5163
1.90E-08	1.65E-05	0.0010	0.0165	0.4345	-0.3620

0	0	0.0010	0.0165	0.9705	-0.0130
0	0	0.0010	0.0165	0.8627	-0.0641
4.43E-08	2.52E-05	0.0010	0.0165	1.0842	0.0351
1.51E-06	0.000146997	0.0010	0.0165	1.1918	0.0762
0	0	0.0010	0.0165	0.5248	-0.2800
0	0	0.0010	0.0165	0.9680	-0.0141
1.93E-08	1.66E-05	0.0010	0.0165	1.0367	0.0156
0	0	0.0010	0.0165	0.6672	-0.1758
0	0	0.0010	0.0165	0.8381	-0.0767
0	0	0.0010	0.0165	0.7420	-0.1296
0	0	0.0010	0.0165	0.7891	-0.1029
2.12E-08	1.74E-05	0.0010	0.0165	0.6349	-0.1973
6.12E-07	9.35E-05	0.0010	0.0165	1.2781	0.1066
0	0	0.0010	0.0165	0.7347	-0.1339
0	0	0.0010	0.0165	0.9179	-0.0372
0	0	0.0010	0.0165	0.9325	-0.0304
0	0	0.0010	0.0165	0.8161	-0.0882
0	0	0.0010	0.0165	0.8769	-0.0570
0	0	0.0010	0.0165	0.9531	-0.0208
2.54E-08	1.90E-05	0.0010	0.0165	1.0298	0.0128
0	0	0.0010	0.0165	0.9613	-0.0171
0	0	0.0010	0.0165	0.9443	-0.0249
0	0	0.0010	0.0165	0.9663	-0.0149
0	0	0.0010	0.0165	0.9564	-0.0194
0	0	0.0010	0.0165	0.9531	-0.0208
0	0	0.0011	0.0169	0.9947	-0.0023
0	0	0.0011	0.0169	0.9947	-0.0023
0	0	0.0011	0.0169	0.9947	-0.0023
0	0	0.0011	0.0169	0.9947	-0.0023
1.99E-10	1.69E-06	0.0012	0.0191	0.9919	-0.0035
1.23E-10	1.33E-06	0.0012	0.0191	0.9919	-0.0035
2.10E-10	1.73E-06	0.0013	0.0192	0.9915	-0.0037
3.14E-09	6.70E-06	0.0015	0.0227	1.0104	0.0045
1.38E-06	0.000140657	0.0020	0.0285	0.3547	-0.4502
3.45E-06	0.000221847	0.0020	0.0285	1.3484	0.1298
2.25E-10	1.79E-06	0.0020	0.0285	0.9051	-0.0433
2.13E-09	5.51E-06	0.0023	0.0321	1.0096	0.0041
6.35E-10	3.01E-06	0.0026	0.0363	1.0067	0.0029
6.23E-10	2.98E-06	0.0034	0.0425	0.9882	-0.0052
0	0	0.0034	0.0425	0.9955	-0.0019
0	0	0.0034	0.0425	0.9955	-0.0019
0	0	0.0034	0.0425	0.9955	-0.0019
0	0	0.0034	0.0425	0.9955	-0.0019
0	0	0.0034	0.0425	0.9955	-0.0019

0	0	0.0034	0.0425	0.9955	-0.0019
0	0	0.0034	0.0425	0.9955	-0.0019
0	0	0.0034	0.0425	0.9955	-0.0019
0	0	0.0034	0.0425	0.9955	-0.0019
0	0	0.0034	0.0425	0.9955	-0.0019
6.35E-10	3.01E-06	0.0038	0.0476	0.9891	-0.0048
1.40E-07	4.47E-05	0.0040	0.0495	0.6171	-0.2096
1.89E-09	5.19E-06	0.0043	0.0533	1.0111	0.0048
5.70E-08	2.85E-05	0.0050	0.0598	1.0774	0.0324
2.71E-06	0.000196681	0.0050	0.0598	1.2240	0.0878
6.40E-10	3.02E-06	0.0050	0.0598	0.9178	-0.0373
2.50E-09	5.98E-06	0.0058	0.0685	1.0118	0.0051
0.00022695	0.001800594	0.0070	0.0816	2.6859	0.4291
1.61E-07	4.80E-05	0.0070	0.0816	1.0637	0.0268
7.30E-10	3.23E-06	0.0071	0.0823	1.0071	0.0031
1.40E-10	1.41E-06	0.0079	0.0910	0.9918	-0.0036
5.87E-09	9.15E-06	0.0080	0.0910	0.9132	-0.0394
0.000379106	0.002327187	0.0090	0.1008	2.3192	0.3653
5.98E-06	0.000292344	0.0090	0.1008	1.5896	0.2013
1.47E-07	4.58E-05	0.0100	0.1094	1.1184	0.0486
3.27E-08	2.16E-05	0.0100	0.1094	1.0579	0.0244
0	0	0.0105	0.1111	0.9964	-0.0016
0	0	0.0105	0.1111	0.9964	-0.0016
0	0	0.0105	0.1111	0.9964	-0.0016
0	0	0.0105	0.1111	0.9964	-0.0016
0	0	0.0105	0.1111	0.9964	-0.0016
1.12E-10	1.26E-06	0.0110	0.1151	0.9206	-0.0359
5.98E-09	9.24E-06	0.0120	0.1246	0.9180	-0.0371
6.05E-10	2.94E-06	0.0125	0.1294	1.0051	0.0022
1.70E-10	1.56E-06	0.0130	0.1330	0.9157	-0.0382
1.80E-09	5.07E-06	0.0132	0.1341	1.0055	0.0024
1.86E-09	5.15E-06	0.0135	0.1364	1.0090	0.0039
1.24E-11	4.21E-07	0.0148	0.1478	0.9960	-0.0018
1.24E-11	4.21E-07	0.0148	0.1478	0.9960	-0.0018
1.31E-05	0.000432831	0.0150	0.1482	1.9280	0.2851
1.72E-06	0.00015694	0.0160	0.1548	0.4624	-0.3350
3.85E-08	2.34E-05	0.0160	0.1548	1.0667	0.0281
1.20E-06	0.000130947	0.0160	0.1548	1.1491	0.0603
3.38E-07	6.95E-05	0.0190	0.1790	1.1610	0.0648
0.001870585	0.005169395	0.0190	0.1790	1.9473	0.2894
9.48E-08	3.68E-05	0.0190	0.1790	0.1855	-0.7317
9.09E-08	3.60E-05	0.0190	0.1790	1.1020	0.0422
3.57E-05	0.000714105	0.0200	0.1848	1.5292	0.1845
6.73E-08	3.10E-05	0.0200	0.1848	1.0772	0.0323

9.26E-10	3.64E-06	0.0200	0.1848	0.9570	-0.0191
0.003882217	0.007447163	0.0210	0.1927	4.2904	0.6325
7.10E-07	0.000100733	0.0220	0.1993	1.2232	0.0875
3.99E-09	7.55E-06	0.0220	0.1993	0.9204	-0.0360
1.17E-07	4.09E-05	0.0230	0.2071	0.6960	-0.1574
0.000148904	0.001458494	0.0240	0.2134	0.1594	-0.7975
1.67E-06	0.000154639	0.0240	0.2134	0.0993	-1.0029
1.00E-05	0.000378332	0.0280	0.2474	1.7536	0.2439
7.54E-10	3.28E-06	0.0295	0.2579	1.0054	0.0023
1.28E-09	4.28E-06	0.0295	0.2579	1.0054	0.0023
0.000312495	0.002112868	0.0300	0.2602	2.5253	0.4023
0	0	0.0329	0.2698	0.9973	-0.0012
0	0	0.0329	0.2698	0.9973	-0.0012
0	0	0.0329	0.2698	0.9973	-0.0012
0	0	0.0329	0.2698	0.9973	-0.0012
0	0	0.0329	0.2698	0.9973	-0.0012
0	0	0.0329	0.2698	0.9973	-0.0012
0	0	0.0329	0.2698	0.9973	-0.0012
0	0	0.0329	0.2698	0.9973	-0.0012
0.01087793	0.012465914	0.0330	0.2698	1.5340	0.1858
9.58E-09	1.17E-05	0.0330	0.2698	1.0280	0.0120
5.70E-06	0.000285457	0.0360	0.2910	1.4578	0.1637
6.54E-10	3.06E-06	0.0360	0.2910	0.9306	-0.0312
0.002179532	0.005579979	0.0380	0.3020	2.5790	0.4115
2.63E-07	6.13E-05	0.0380	0.3020	0.4075	-0.3899
1.03E-08	1.21E-05	0.0380	0.3020	1.0313	0.0134
1.24E-11	4.21E-07	0.0392	0.3082	0.9969	-0.0014
3.20E-08	2.14E-05	0.0410	0.3187	1.0537	0.0227
1.02E-07	3.81E-05	0.0410	0.3187	0.7873	-0.1039
1.10E-06	0.000125524	0.0430	0.3325	1.1576	0.0636
7.77E-09	1.05E-05	0.0444	0.3420	1.0069	0.0030
1.80E-09	5.07E-06	0.0473	0.3602	1.0046	0.0020
1.09E-09	3.95E-06	0.0473	0.3602	1.0046	0.0020
1.00E-05	0.000378506	0.0480	0.3605	1.3718	0.1373
1.90E-08	1.65E-05	0.0480	0.3605	1.0380	0.0162
3.18E-10	2.13E-06	0.0483	0.3605	1.0050	0.0022
3.68E-10	2.29E-06	0.0483	0.3605	1.0050	0.0022
1.73E-09	4.97E-06	0.0500	0.3705	1.0072	0.0031
