# Supplementary material for: Combined Non-Invasive Prediction and New Biomarkers of Oral and Fecal Microbiota in Patients With Gastric and Colorectal Cancer
Source: Front Cell Infect Microbiol. 2022 May 19;12:830684. doi: 10.3389/fcimb.2022.830684 (PMC9161364; doi:10.3389/fcimb.2022.830684)
Supplement: Supplementary file 1 [file DataSheet_1.zip › Supplementary Table 5.pdf]

Table S5. Metastases analysis of phylum and genus levels of oral samples in tissue of GC and CRC

Phylum	mean. group1.	variance. group1.	standard. error. group1.	mean. group2.
**GC.Tumor-CRC.Tumor-Phylum**				
p__Armatimonadota	1.23E-06	3.63E-11	1.23E-06	2.59E-05
p__Margulisbacteria	0	0	0	1.88E-05
p__Campilobacterota	0.010137627	0.000176719	0.002713541	0.183772466
p__Gracilibacteria	0	0	0	4.20E-05
p__Deinococcota	2.46E-06	1.45E-10	2.46E-06	0.000507792
p__Sva0485	3.69E-06	1.75E-10	2.70E-06	2.06E-05
p__Fusobacteriota	0.132137027	0.052475701	0.046759892	0.012455211
p__Planctomycetes	6.15E-06	4.53E-10	4.34E-06	2.24E-05
p__Spirochaetota	0.000208973	4.07E-07	0.00013024	0.007072435
p__Hydrogenedentes	1.11E-05	1.88E-09	8.85E-06	1.79E-06
p__Bdellovibrionota	6.27E-05	1.90E-08	2.82E-05	0.000243168
p__Proteobacteria	0.274664906	0.063372848	0.051386139	0.118564093
p__Thermoplasmatota	1.97E-05	5.65E-09	1.53E-05	7.15E-06
p__Kapabacteria	4.92E-06	2.02E-10	2.90E-06	0.000121584
p__Elusimicrobia	0	0	0	6.26E-06
p__Halanaerobiaeota	0	0	0	0.000327204
p__Acetothermia	0	0	0	5.36E-06
p__WOR-1	0	0	0	5.36E-06
p__Desulfobacterota	0.002264279	1.49E-05	0.000789179	0.00061239
**GC.Para-CRC.Para -Phylum**				
p__Fibrobacterota	2.46E-06	6.94E-11	1.70E-06	2.46E-05
p__Nitrospinota	0	0	0	1.48E-05
p__Euryarchaeota	4.92E-06	2.02E-10	2.90E-06	2.70E-05
p__Campilobacterota	0.009188646	0.000181092	0.002746909	0.351904027
p__Firmicutes	0.225400244	0.016941291	0.026568536	0.098708796
p__Fusobacteriota	0.12108115	0.029504325	0.035062043	0.009160373
p__Bacteroidota	0.186295335	0.016281668	0.026046168	0.047806362
p__Thermotogae	1.23E-06	3.63E-11	1.23E-06	1.48E-05
p__Sumerlaeota	0	0	0	3.03E-05
p__Crenarchaeota	9.83E-06	1.19E-09	7.03E-06	0.000784262
p__Thermoplasmatota	0	0	0	9.01E-06
p__Halobacterota	0	0	0	6.39E-05
p__Proteobacteria	0.250366317	0.037840163	0.039707348	0.127267557
p__Kryptonia	7.38E-06	1.31E-09	7.38E-06	2.21E-05
p__RCP2-54	1.35E-05	4.39E-09	1.35E-05	0.000270435
p__Elusimicrobiota	1.11E-05	2.94E-09	1.11E-05	0.000109813
p__Myxococcota	0.000666254	2.53E-06	0.000324803	0.003130491
p__Bdellovibrionota	7.99E-05	4.47E-08	4.32E-05	0.000397458

p__Dadabacteria	3.69E-06	3.26E-10	3.69E-06	0.000203236
p__Planctomycetota	2.58E-05	3.20E-09	1.16E-05	0.000204875
p__Desulfobacterota	0.001811915	6.86E-06	0.000534611	0.000549885
p__Chloroflexi	0.001708658	4.78E-05	0.001410754	0.008594098
p__Entotheonellaeota	9.59E-05	2.21E-07	9.59E-05	0.001510339
p__Acidobacteriota	0.004323273	0.000361092	0.003878852	0.037625711
p__NB1-j	5.29E-05	6.71E-08	5.29E-05	0.000464657
p__Cyanobacteria	0.000701902	3.43E-06	0.00037806	0.002431457
p__GAL15	2.46E-06	1.45E-10	2.46E-06	1.07E-05
p__Latescibacteria	3.69E-06	3.26E-10	3.69E-06	5.74E-05
p__MBNT15	4.79E-05	2.66E-08	3.33E-05	0.000218807
p__Hydrogenedentes	7.38E-06	7.00E-10	5.40E-06	5.08E-05
**GC.Tumor-GC.Para-Phylum**				
p__Margulisbacteria	0	0	0	1.88E-05
p__Hydrogenedentes	5.08E-05	1.39E-08	1.96E-05	1.79E-06
p__Acetothermia	0	0	0	5.36E-06
p__Thermotogae	1.48E-05	6.24E-09	1.32E-05	4.47E-06
p__Chloroflexi	0.008594098	0.0002723	0.002750254	0.002133978
p__Myxococcota	0.003130491	2.87E-05	0.000892153	0.000984294
p__Dadabacteria	0.000203236	2.13E-07	7.70E-05	1.61E-05
p__RCP2-54	0.000270435	2.95E-07	9.05E-05	6.17E-05
p__Entotheonellaeota	0.001510339	1.24E-05	0.000587548	0.000195786
p__Campilobacterota	0.351904027	0.125623116	0.059072252	0.183772466
p__Acidobacteriota	0.037625711	0.007305396	0.014245268	0.005732329
p__Latescibacterota	0.000487603	9.78E-07	0.000164783	0.000136782
**CRC.Tumor-CRC.Para-Phylum**				
p__Thermoplasmatota	0	0	0	1.97E-05
p__Halanaerobiaeota	1.72E-05	7.11E-09	1.72E-05	0
p__Halobacterota	0	0	0	4.30E-05
p__Parcubacteria	4.06E-05	3.27E-08	3.69E-05	0
p__Armatimonadota	1.48E-05	3.71E-09	1.24E-05	1.23E-06
p__Latescibacteria	3.69E-06	3.26E-10	3.69E-06	1.48E-05
p__Crenarchaeota	9.83E-06	1.19E-09	7.03E-06	0.000159803

variance. group2.	standard. error. group2.	p.value	q.value	FC	log10FC
**GC.Tumor-CRC.Tumor-Phylum**					
7.33E-09	1.49E-05	0.0000	0.0002	1.0247	0.0106
8.58E-09	1.61E-05	0.0000	0.0007	1.0188	0.0081
0.065926191	0.044696346	0.0010	0.0166	16.5899	1.2198
1.65E-08	2.24E-05	0.0010	0.0166	1.0420	0.0179
7.13E-06	0.000464754	0.0010	0.0166	1.5041	0.1773
9.60E-09	1.71E-05	0.0011	0.0166	1.0168	0.0072
0.000626722	0.004357934	0.0040	0.0440	0.1011	-0.9954

4.68E-09	1.19E-05	0.0048	0.0469	1.0161	0.0069
0.001207468	0.006048962	0.0090	0.0791	6.6771	0.8246
1.05E-10	1.79E-06	0.0113	0.0904	0.9908	-0.0040
1.75E-07	7.29E-05	0.0150	0.1099	1.1698	0.0681
0.028364331	0.02931766	0.0180	0.1217	0.4337	-0.3628
6.54E-10	4.45E-06	0.0212	0.1289	0.9877	-0.0054
2.35E-07	8.43E-05	0.0220	0.1289	1.1161	0.0477
5.31E-10	4.01E-06	0.0241	0.1328	1.0063	0.0027
1.44E-06	0.000209117	0.0360	0.1862	1.3272	0.1229
9.49E-10	5.36E-06	0.0432	0.1978	1.0054	0.0023
5.14E-10	3.95E-06	0.0432	0.1978	1.0054	0.0023
7.48E-07	0.00015053	0.0450	0.1978	0.4939	-0.3063
**GC.Para-CRC.Para -Phylum**					
3.80E-09	1.03E-05	0.0000	0.0028	1.0221	0.0095
3.80E-09	1.03E-05	0.0001	0.0043	1.0148	0.0064
8.52E-09	1.54E-05	0.0001	0.0043	1.0220	0.0095
0.125623116	0.059072252	0.0010	0.0126	34.6370	1.5395
0.010610255	0.017167682	0.0010	0.0126	0.4404	-0.3561
0.000503436	0.003739562	0.0010	0.0126	0.0832	-1.0797
0.005806317	0.012699866	0.0010	0.0126	0.2606	-0.5841
6.24E-09	1.32E-05	0.0015	0.0162	1.0135	0.0058
6.49E-09	1.34E-05	0.0020	0.0195	1.0303	0.0130
9.56E-06	0.000515441	0.0040	0.0320	1.7669	0.2472
2.43E-09	8.21E-06	0.0044	0.0320	1.0090	0.0039
7.00E-08	4.41E-05	0.0060	0.0406	1.0639	0.0269
0.015383512	0.020671714	0.0110	0.0672	0.5103	-0.2922
2.65E-09	8.59E-06	0.0118	0.0672	1.0146	0.0063
2.95E-07	9.05E-05	0.0130	0.0672	1.2535	0.0981
5.31E-08	3.84E-05	0.0130	0.0672	1.0977	0.0405
2.87E-05	0.000892153	0.0140	0.0684	2.4789	0.3943
5.40E-07	0.00012248	0.0180	0.0791	1.2941	0.1120
2.13E-07	7.70E-05	0.0180	0.0791	1.1988	0.0788
1.70E-07	6.88E-05	0.0190	0.0795	1.1746	0.0699
5.55E-07	0.00012417	0.0220	0.0879	0.5512	-0.2587
0.0002723	0.002750254	0.0260	0.0949	3.5420	0.5493
1.24E-05	0.000587548	0.0270	0.0949	2.2907	0.3600
0.007305396	0.014245268	0.0300	0.1009	7.2560	0.8607
1.18E-06	0.000181023	0.0310	0.1009	1.3911	0.1434
1.65E-05	0.000677418	0.0360	0.1106	2.0162	0.3045
8.53E-10	4.87E-06	0.0365	0.1106	1.0082	0.0035
1.94E-08	2.32E-05	0.0380	0.1114	1.0535	0.0226
1.94E-07	7.33E-05	0.0470	0.1291	1.1630	0.0656
1.39E-08	1.96E-05	0.0470	0.1291	1.0431	0.0183
**GC.Tumor-GC.Para-Phylum**					

8.58E-09	1.61E-05	0.0000	0.0000	1.0188	0.0081
1.05E-10	1.79E-06	0.0080	0.2161	0.9533	-0.0207
9.49E-10	5.36E-06	0.0120	0.2161	1.0054	0.0023
2.79E-10	2.91E-06	0.0123	0.2161	0.9899	-0.0044
9.77E-06	0.000544146	0.0240	0.2711	0.3267	-0.4859
3.66E-06	0.000333035	0.0280	0.2711	0.4804	-0.3184
2.18E-09	8.13E-06	0.0300	0.2711	0.8445	-0.0734
1.96E-08	2.44E-05	0.0320	0.2711	0.8357	-0.0780
2.06E-07	7.91E-05	0.0330	0.2711	0.4763	-0.3221
0.065926191	0.044696346	0.0340	0.2711	0.5236	-0.2810
8.91E-05	0.001643523	0.0370	0.2711	0.1743	-0.7587
1.04E-07	5.62E-05	0.0470	0.2741	0.7642	-0.1168
**CRC.Tumor-CRC.Para-Phylum**					
5.65E-09	1.53E-05	0.0000	0.0027	1.0197	0.0085
0	0	0.0001	0.0054	0.9831	-0.0074
1.01E-08	2.05E-05	0.0010	0.0176	1.0430	0.0183
0	0	0.0010	0.0176	0.9610	-0.0173
3.63E-11	1.23E-06	0.0034	0.0501	0.9867	-0.0058
2.04E-09	9.23E-06	0.0352	0.3437	1.0110	0.0048
2.17E-07	9.51E-05	0.0490	0.4076	1.1485	0.0601

Genus	mean. group1.	variance. group1.	standard. error. group1.	mean. group2.
**GC.Tumor-CRC.Tumor-Phylum**				
g__Sporolactobacillus	2.83E-05	6.47E-09	1.64E-05	0
g__Merdibacter	2.58E-05	8.12E-09	1.84E-05	0
g__Dysgonomonas	0	0	0	2.77E-05
g__Litoreibacter	0	0	0	2.77E-05
g__Fournierella	2.34E-05	3.63E-09	1.23E-05	0
g__Vulcaniibacterium	0	0	0	2.68E-05
g__Shuttleworthia	0	0	0	2.59E-05
g__Tychonema_CCAP_1459-11B	0	0	0	2.59E-05
g__unidentified_Rikenellaceae	2.09E-05	1.05E-08	2.09E-05	0
g__Friedmanniella	1.23E-06	3.63E-11	1.23E-06	2.86E-05
g__Marinobacter	0	0	0	2.41E-05
g__Tepidanaerobacter	0	0	0	2.41E-05
g__Arcticibacter	0	0	0	2.32E-05
g__Helcococcus	1.97E-05	9.28E-09	1.97E-05	0
g__Thermanaerovibrio	1.23E-06	3.63E-11	1.23E-06	2.68E-05
g__Williamsia	0	0	0	2.24E-05
g__Franconibacter	0	0	0	2.24E-05
g__Clostridium_sensu_stricto_14	0	0	0	2.24E-05
g__Stenotrophobacter	0	0	0	2.15E-05

g__Blastocatella	0	0	0	2.06E-05
g__Auricoccus-Abyssicoccus	0	0	0	2.06E-05
g__Papillibacter	2.46E-06	6.94E-11	1.70E-06	2.77E-05
g__Erysipelothrix	0	0	0	1.97E-05
g__CL500-3	0	0	0	1.97E-05
g__Nevskia	0	0	0	1.97E-05
g__C39	0	0	0	1.97E-05
g__Mobiluncus	1.97E-05	5.27E-09	1.48E-05	8.94E-07
g__Marinicella	0	0	0	1.88E-05
g__NS5_marine_group	2.46E-06	1.45E-10	2.46E-06	2.50E-05
g__Coprobacter	2.09E-05	5.41E-09	1.50E-05	1.79E-06
g__Methanobacterium	2.09E-05	7.30E-09	1.74E-05	1.79E-06
g__Longispora	0	0	0	1.79E-05
g__RS62_marine_group	0	0	0	1.79E-05
g__Crocinitomix	0	0	0	1.79E-05
g__Iodobacter	1.48E-05	5.22E-09	1.48E-05	0
g__Geobacter	1.23E-06	3.63E-11	1.23E-06	2.06E-05
g__Senegalimassilia	1.72E-05	3.10E-09	1.14E-05	8.94E-07
g__Coriobacteriaceae_UCG-002	1.72E-05	2.11E-09	9.38E-06	8.94E-07
g__Pyramidobacter	1.23E-06	3.63E-11	1.23E-06	1.97E-05
g__Candidatus_Nitrocosmicus	1.23E-06	3.63E-11	1.23E-06	1.97E-05
g__Oligoflexus	3.69E-06	3.26E-10	3.69E-06	2.59E-05
g__Desulfurivibrio	0	0	0	1.61E-05
g__Sulfuritalea	1.23E-06	3.63E-11	1.23E-06	1.88E-05
g__Fastidiosipila	2.34E-05	2.42E-09	1.00E-05	3.58E-06
g__Fermentimonas	3.69E-06	1.75E-10	2.70E-06	2.50E-05
g__Thermoanaerobaculum	0	0	0	1.43E-05
g__Propioniciclava	0	0	0	1.43E-05
g__NS2b_marine_group	2.46E-06	1.45E-10	2.46E-06	2.15E-05
g__Oligella	1.84E-05	8.16E-09	1.84E-05	1.79E-06
g__Buchnera	1.23E-06	3.63E-11	1.23E-06	1.79E-05
g__Planosporangium	1.48E-05	5.22E-09	1.48E-05	8.94E-07
g__Motilibacter	0	0	0	1.34E-05
g__Zoogloea	0	0	0	1.34E-05
g__Tahibacter	0	0	0	1.34E-05
g__Sulfitobacter	0	0	0	1.34E-05
g__Ohtaekwangia	2.83E-05	9.65E-09	2.01E-05	7.15E-06
g__Sulfuriferula	1.11E-05	1.12E-09	6.83E-06	0
g__Caldalkalibacillus	2.70E-05	6.73E-09	1.67E-05	6.26E-06
g__Ignatzschineria	0	0	0	1.25E-05
g__Rhizocola	0	0	0	1.25E-05
g__Formosa	0	0	0	1.25E-05
g__966-1	0	0	0	1.25E-05
g__Candidatus_Megaira	0	0	0	1.25E-05

g__Chromohalobacter	0	0	0	1.25E-05
g__Polyangium	4.92E-06	2.78E-10	3.40E-06	2.41E-05
g__Anaerovibrio	1.60E-05	6.13E-09	1.60E-05	1.79E-06
g__Dolosigranulum	1.72E-05	7.11E-09	1.72E-05	2.68E-06
g__Frisingicoccus	9.83E-06	8.83E-10	6.07E-06	0
g__Pelagibacterium	9.83E-06	2.32E-09	9.83E-06	0
g__Filimonas	9.83E-06	5.80E-10	4.92E-06	0
g__Pseudokineococcus	2.46E-06	6.94E-11	1.70E-06	1.88E-05
g__Helicobacter	0.008047901	0.000179194	0.002732473	0.18315203
g__Bacteroides	0.066748289	0.005631663	0.015318375	0.003468721
g__Streptococcus	0.003825427	5.08E-05	0.001455465	0.023605181
g__Faecalibacterium	0.015814305	0.000557332	0.00481894	0.001458114
g__Capnocytophaga	6.15E-06	4.53E-10	4.34E-06	0.00011175
g__Ureaplasma	0.004608459	0.000402411	0.004094769	0
g__[Ruminococcus]_torques_group	0.004726467	4.43E-05	0.001358297	0.000336144
g__TM7x	1.23E-06	3.63E-11	1.23E-06	5.36E-05
g__Dorea	0.003171466	2.21E-05	0.000960214	0.00019668
g__Lachnospiraceae_UCG-010	0.00273877	3.56E-05	0.00121828	7.06E-05
g__Centipeda	2.83E-05	6.70E-09	1.67E-05	0.00153768
g__Catonella	2.95E-05	7.19E-09	1.73E-05	0.001872036
g__Salinisphaera	0.00095021	2.09E-05	0.000933577	0
g__Stomatobaculum	2.09E-05	6.32E-09	1.62E-05	0.001222992
g__Stenotrophomonas	5.41E-05	5.88E-09	1.56E-05	0.001097832
g__SD04E11	0.000588811	8.14E-06	0.000582419	0
g__Peptoclostridium	1.23E-06	3.63E-11	1.23E-06	6.97E-05
g__Clostridium_sensu_stricto_18	0.000228641	4.63E-07	0.000138903	0
g__Oribacterium	1.84E-05	3.24E-09	1.16E-05	0.000684804
g__Chelatococcus	0.000282728	1.78E-06	0.000272522	0
g__Planomicrobium	0	0	0	4.20E-05
g__Parvibaculum	0.000108174	2.81E-07	0.000108174	0
g__Taeseokella	0	0	0	4.47E-05
g__Nubsella	6.02E-05	8.71E-08	6.02E-05	0
g__Oceanobacillus	0	0	0	7.87E-05
g__Clostridium_sensu_stricto_15	6.76E-05	7.84E-08	5.71E-05	0
g__[Bacteroides]_pectinophilus_group	3.81E-05	1.71E-08	2.67E-05	0
g__Rhodobacter	0	0	0	4.56E-05
g__Methylomonas	0	0	0	1.16E-05
g__Aridibacter	0	0	0	1.16E-05
g__UCG-012	0	0	0	1.16E-05
g__Lachnospiraceae_UCG-004	1.23E-05	5.23E-10	4.67E-06	8.94E-07
g__Paludibaculum	6.15E-06	3.77E-10	3.96E-06	2.50E-05
g__Anaerolinea	1.48E-05	2.57E-09	1.04E-05	1.79E-06
g__Azospira	2.70E-05	1.44E-08	2.45E-05	8.05E-06

g__Holdemania	1.60E-05	2.12E-09	9.39E-06	2.68E-06
g__Cohnella	1.60E-05	2.50E-09	1.02E-05	2.68E-06
g__Actinospica	3.69E-06	1.75E-10	2.70E-06	1.97E-05
g__Caenimonas	3.69E-06	3.26E-10	3.69E-06	1.97E-05
g__Defluviitaleaceae_UCG-011	7.38E-06	9.27E-10	6.22E-06	2.68E-05
g__Marinobacterium	0	0	0	1.07E-05
g__Halarcobacter	0	0	0	1.07E-05
g__Sporanaerobacter	0	0	0	1.07E-05
g__[Anaerorhabdus]_furcosa_group	0	0	0	1.07E-05
g__Pseudophaeobacter	0	0	0	1.07E-05
g__Lachnoclostridium	0.005199729	5.10E-05	0.00145773	0.000951216
g__Delftia	9.34E-05	1.06E-07	6.63E-05	0.001182762
g__Lentimicrobium	1.84E-05	5.28E-09	1.48E-05	0.000852876
g__Haloplasma	0	0	0	0.00016539
g__Asticcacaulis	1.23E-06	3.63E-11	1.23E-06	1.43E-05
g__Dyella	2.83E-05	6.85E-09	1.69E-05	8.94E-06
g__Xanthobacter	8.60E-06	8.69E-10	6.02E-06	0
g__Lachnospiraceae_NC2004_group	8.60E-06	5.66E-10	4.86E-06	0
g__Planifilum	8.60E-06	1.32E-09	7.42E-06	0
g__Antarcticibacterium	8.60E-06	1.32E-09	7.42E-06	0
g__Truepera	2.46E-06	1.45E-10	2.46E-06	1.61E-05
g__Actinoallomurus	2.46E-06	6.94E-11	1.70E-06	1.61E-05
g__W5053	1.35E-05	4.39E-09	1.35E-05	1.79E-06
g__Subdoligranulum	0.00926363	0.000817079	0.005834806	0.000571266
g__Actinomyces	0.000167178	8.40E-08	5.92E-05	0.001106772
g__Coprococcus	0.001858626	9.08E-06	0.000615095	0.00015198
g__Deinococcus	0	0	0	0.000489018
g__Johnsonella	2.46E-06	1.45E-10	2.46E-06	0.00029502
g__[Eubacterium]_yurii_group	8.60E-06	7.17E-10	5.47E-06	0.000425544
g__Weissella	1.72E-05	1.43E-09	7.72E-06	0.000121584
g__Solitalea	7.38E-06	1.31E-09	7.38E-06	2.59E-05
g__Polycyclovorans	4.92E-06	3.53E-10	3.84E-06	2.06E-05
g__Adlercreutzia	2.70E-05	6.81E-09	1.68E-05	8.94E-06
g__Dermabacter	0	0	0	9.83E-06
g__Demequina	0	0	0	9.83E-06
g__Methylonatrum	0	0	0	9.83E-06
g__DNF00809	0	0	0	9.83E-06
g__Candidatus_Finniella	0	0	0	9.83E-06
g__Kutzneria	0	0	0	9.83E-06
g__Aurantivirga	0	0	0	9.83E-06
g__Ruminiclostridium	2.21E-05	5.47E-09	1.51E-05	6.26E-06
g__Escherichia-Shigella	0.105469672	0.040137861	0.040895121	0.010687772
g__Klebsiella	0.084786307	0.027704315	0.033975675	0.005327347
g__Parabacteroides	0.007556201	0.00053329	0.004713855	0.000600768

g__Erysipelatoclostridium	0.002499066	4.76E-05	0.001408615	9.83E-05
g__UCG-003	0.000356483	7.87E-07	0.000181071	3.13E-05
g__[Eubacterium]_nodatum_group	4.30E-05	4.99E-09	1.44E-05	0.000204726
g__Peptoanaerobacter	4.92E-06	2.78E-10	3.40E-06	0.000181482
g__Thermicanus	7.38E-06	1.31E-09	7.38E-06	0
g__Xylanibacillus	7.38E-06	6.24E-10	5.10E-06	0
g__Anaerostipes	0.000481866	4.28E-07	0.00013356	0.000112644
g__Rahnella1	2.46E-06	6.94E-11	1.70E-06	5.01E-05
g__TM7	2.09E-05	2.84E-09	1.09E-05	6.26E-06
g__CAG-352	1.23E-06	3.63E-11	1.23E-06	1.16E-05
g__Sandaracinus	1.23E-06	3.63E-11	1.23E-06	1.25E-05
g__Propionibacterium	0	0	0	8.94E-06
g__Microcoleus_SAG_1449-1a	0	0	0	8.94E-06
g__marine_group	0	0	0	8.94E-06
g__possible_genus_04	0	0	0	8.94E-06
g__Blautia	0.004781783	2.72E-05	0.001064705	0.001349046
g__Phascolarctobacterium	0.00070436	2.75E-06	0.000338763	3.75E-05
g__Mycoplasma	0	0	0	0.000154662
g__Anaerovorax	2.46E-06	6.94E-11	1.70E-06	1.43E-05
g__Rhodoluna	4.92E-06	5.80E-10	4.92E-06	1.88E-05
g__Fusobacterium	0.107504081	0.043936236	0.042786406	0.0102962
g__Anoxybacillus	0	0	0	0.000313794
g__Succinivibrionaceae_UCG-002	3.69E-06	3.26E-10	3.69E-06	1.70E-05
g__Fictibacillus	3.69E-06	9.93E-11	2.03E-06	1.70E-05
g__Succiniclasticum	9.83E-06	1.41E-09	7.67E-06	2.68E-05
g__Saccharofermentans	2.46E-06	1.45E-10	2.46E-06	5.99E-05
g__Providencia	2.46E-06	6.94E-11	1.70E-06	3.13E-05
g__UCG-002	0.002653951	4.04E-05	0.001297955	0.000235122
g__Hungatella	0.000264289	3.04E-07	0.000112571	3.31E-05
g__Alsobacter	0	0	0	5.72E-05
g__Acetobacter	1.48E-05	2.04E-09	9.23E-06	3.58E-06
g__Idiomarina	7.38E-06	9.27E-10	6.22E-06	2.24E-05
g__Labrys	1.23E-06	3.63E-11	1.23E-06	1.07E-05
g__Trueperella	1.23E-06	3.63E-11	1.23E-06	1.07E-05
g__Erysipelotrichaceae_UCG-003	0.000175783	1.68E-07	8.38E-05	2.32E-05
g__Wolinella	2.46E-06	1.45E-10	2.46E-06	9.21E-05
g__Sharpea	8.60E-06	9.44E-10	6.27E-06	8.94E-07
g__Herbaspirillum	7.38E-06	1.31E-09	7.38E-06	0.00919211
g__unidentified_P5D1-392	2.46E-06	1.45E-10	2.46E-06	1.34E-05
g__Pseudogracilibacillus	0	0	0	8.05E-06
g__Actinomadura	0	0	0	8.05E-06
g__Paraclostridium	0	0	0	0.000142146
g__Legionella	0	0	0	3.31E-05
g__Hydrogenoanaerobacterium	6.15E-06	9.07E-10	6.15E-06	0

g__Rummeliibacillus	6.15E-06	3.01E-10	3.54E-06	0
g__FCPS473	6.15E-06	4.53E-10	4.34E-06	0
g__UTBCD1	6.15E-06	9.07E-10	6.15E-06	0
g__Oceanisphaera	6.15E-06	9.07E-10	6.15E-06	0
g__Leptolinea	6.15E-06	9.07E-10	6.15E-06	0
g__unidentified_11-24	6.15E-06	4.53E-10	4.34E-06	0
g__Alcaligenes	7.74E-05	1.41E-08	2.42E-05	0.000526566
g__Treponema	0.000201597	3.87E-07	0.000126926	0.007057237
g__Eggerthella	0.000259372	2.14E-07	9.43E-05	5.54E-05
g__Parafilimonas	1.35E-05	2.57E-09	1.04E-05	3.58E-06
g__Petrimonas	1.23E-06	3.63E-11	1.23E-06	9.83E-06
g__Aminobacter	4.92E-06	5.80E-10	4.92E-06	1.70E-05
g__[Clostridium]_innocuum_group	0.000478178	1.44E-06	0.000244978	2.15E-05
g__Atopostipes	8.60E-06	4.90E-10	4.52E-06	4.29E-05
g__DTU089	2.46E-06	1.45E-10	2.46E-06	1.25E-05
g__Rubellimicrobium	8.60E-06	1.32E-09	7.42E-06	2.24E-05
g__Oxalobacter	3.69E-06	1.75E-10	2.70E-06	1.43E-05
g__Prevotellaceae_UCG-004	1.23E-05	3.63E-09	1.23E-05	2.68E-06
g__Roseburia	0.002572821	2.45E-05	0.001010356	0.000508686
g__Puia	2.21E-05	3.05E-09	1.13E-05	8.94E-06
g__SM1A02	2.21E-05	8.34E-09	1.86E-05	8.94E-06
g__Syntrophorhabdus	9.83E-06	1.19E-09	7.03E-06	1.79E-06
g__Kurthia	0.001057155	7.59E-06	0.000562497	2.41E-05
g__Rheinheimera	3.69E-06	3.26E-10	3.69E-06	4.47E-05
g__Vulgatibacter	0	0	0	6.26E-06
g__Desulfohalotomaculum	0	0	0	6.26E-06
g__Rickettsia	0	0	0	6.26E-06
g__Colwellia	0	0	0	6.26E-06
g__Chromobacterium	0	0	0	6.26E-06
g__F0058	0	0	0	7.15E-06
g__unidentified_F082	0	0	0	7.15E-06
g__IMCC26207	0	0	0	7.15E-06
g__Agaricicola	0	0	0	7.15E-06
g__Panacagrimonas	0	0	0	7.15E-06
g__Geminicoccus	0	0	0	7.15E-06
g__Sedimentibacter	0	0	0	7.15E-06
g__Gemmata	0	0	0	7.15E-06
g__Methyloparacoccus	0	0	0	7.15E-06
g__AAP99	0	0	0	7.15E-06
g__Methylobacter	0	0	0	7.15E-06
g__Trichloromonas	0	0	0	7.15E-06
g__Planktotalea	0	0	0	7.15E-06
g__Acidipila	0	0	0	7.15E-06
g__SH3-11	1.48E-05	2.12E-09	9.40E-06	4.47E-06

g__Maricaulis	1.48E-05	5.22E-09	1.48E-05	4.47E-06
g__Acetoanaerobium	2.46E-06	1.45E-10	2.46E-06	5.45E-05
g__Sellimonas	0.000222494	2.96E-07	0.000110984	2.06E-05
g__Collinsella	0.003749213	8.06E-05	0.00183271	0.000341508
g__Exiguobacterium	8.24E-05	3.07E-08	3.57E-05	0.000439848
g__Ellin516	6.15E-06	4.53E-10	4.34E-06	1.79E-05
g__UTCFX1	2.09E-05	2.91E-09	1.10E-05	8.05E-06
g__Filifactor	1.11E-05	1.80E-09	8.67E-06	0.000154662
g__Leuconostoc	0.000283957	1.01E-06	0.00020531	0.002323506
g__Mitsuokella	1.23E-06	3.63E-11	1.23E-06	4.02E-05
g__Flaviflexus	0	0	0	5.36E-05
g__Halocella	0	0	0	0.000327204
g__Caldicoprobacter	0	0	0	3.93E-05
g__Luteibacter	0	0	0	5.01E-05
g__Dyadobacter	2.46E-05	8.38E-09	1.87E-05	1.07E-05
g__Megamonas	0.000347878	6.04E-07	0.000158654	5.45E-05
g__Butyricicoccus	0.000486783	6.07E-07	0.000159091	0.000161814
g__Microlunatus	0	0	0	0.000292338
g__Sphingobacterium	1.23E-06	3.63E-11	1.23E-06	8.94E-06
g__Ileibacterium	1.23E-06	3.63E-11	1.23E-06	8.94E-06
g__Gordonibacter	5.90E-05	2.26E-08	3.07E-05	4.47E-06
g__Ezakiella	1.23E-05	1.89E-09	8.86E-06	3.58E-06
g__Methanosphaera	4.92E-06	3.53E-10	3.84E-06	0
g__CHKCI002	4.92E-06	2.78E-10	3.40E-06	0
g__Candidatus_Nitrososphaera	4.92E-06	5.80E-10	4.92E-06	0
g__Azoarcus	4.92E-06	5.80E-10	4.92E-06	0
g__SEEP-SRB1	4.92E-06	5.80E-10	4.92E-06	0
g__Thermoflavimicrobium	4.92E-06	2.02E-10	2.90E-06	0
g__Chroococcidiopsis_SAG_2023	4.92E-06	5.80E-10	4.92E-06	0
g__Desulfomonile	4.92E-06	3.53E-10	3.84E-06	0
g__Veillonellaceae_UCG-001	4.92E-06	2.02E-10	2.90E-06	0
g__Microbacterium	2.46E-06	6.94E-11	1.70E-06	8.94E-05
g__Aurantimicrobium	1.35E-05	9.07E-10	6.15E-06	0.000112644
g__Allobaculum	2.46E-06	6.94E-11	1.70E-06	1.16E-05
g__Cyanobium_PCC-6307	2.46E-06	6.94E-11	1.70E-06	0.000329886
g__Solibacillus	1.23E-06	3.63E-11	1.23E-06	4.56E-05
g__Sinibacillus	6.15E-06	9.07E-10	6.15E-06	0.000520308
g__Marmoricola	2.09E-05	2.16E-09	9.48E-06	0.000202938
g__Paenibacillus	0.000414257	6.92E-07	0.00016975	4.29E-05
g__Mogibacterium	2.95E-05	9.23E-09	1.96E-05	0.000150192
g__Georgenia	2.46E-06	1.45E-10	2.46E-06	4.11E-05
g__Hyphomicrobium	4.92E-06	3.53E-10	3.84E-06	4.47E-05
g__Bergeyella	6.15E-06	4.53E-10	4.34E-06	0.001657476
g__Gordonia	0	0	0	5.36E-06

g__Proteus	0	0	0	5.36E-06
g__Parviterribacter	0	0	0	5.36E-06
g__Tepidimonas	0	0	0	5.36E-06
g__Limnochorda	0	0	0	5.36E-06
g__Haliea	0	0	0	5.36E-06
g__Promicromonospora	0	0	0	5.36E-06
g__Leucobacter	0	0	0	5.36E-06
g__Sva0081_sediment_group	0	0	0	5.36E-06
g__Denitromonas	0	0	0	5.36E-06
g__CK06-06-Mud-MAS4B-21	0	0	0	5.36E-06
g__CENA518	0	0	0	5.36E-06
g__Litoricola	0	0	0	5.36E-06
g__Oceanimonas	0	0	0	5.36E-06
g__Sporichthya	0	0	0	5.36E-06
g__Aliidiomarina	0	0	0	5.36E-06
g__Prevotellaceae_YAB2003_group	0	0	0	5.36E-06
g__Balneola	0	0	0	5.36E-06
g__Prochlorothrix_PCC-9006	0	0	0	5.36E-06
g__DMER64	0	0	0	5.36E-06
g__Thermobifida	0	0	0	5.36E-06
g__Tuberibacillus	0	0	0	5.36E-06
g__Candidatus_Jidaibacter	0	0	0	5.36E-06
g__Thermophagus	0	0	0	5.36E-06
g__Sphingopyxis	4.92E-06	3.53E-10	3.84E-06	1.52E-05
g__unidentified_Sandaracinaceae	4.92E-06	3.53E-10	3.84E-06	1.52E-05
g__Virgisporangium	2.70E-05	6.43E-09	1.64E-05	1.34E-05
g__Clostridium_sensu_stricto_12	7.74E-05	2.46E-08	3.20E-05	1.16E-05
g__Dubosiella	0.000891206	5.11E-06	0.000461626	0.000133206
g__FukuN57	3.69E-06	3.26E-10	3.69E-06	0.000206514
g__Emticicia	3.69E-06	3.26E-10	3.69E-06	1.25E-05
g__Aerococcus	2.21E-05	4.03E-09	1.30E-05	0.001257858
g__Phreatobacter	0.000515056	9.88E-07	0.000202847	8.05E-05
g__Tannerella	7.38E-06	9.27E-10	6.22E-06	1.88E-05
**GC.Para-CRC.Para-Genus**				
g__Tepidiphilus	0	0	0	2.87E-05
g__Rhizocola	0	0	0	2.79E-05
g__Aurantisolimonas	0	0	0	2.79E-05
g__Geobacter	0	0	0	2.70E-05
g__Psychroglaciecola	2.83E-05	1.18E-08	2.22E-05	1.64E-06
g__Gelria	0	0	0	2.62E-05
g__Litoribrevibacter	0	0	0	2.62E-05
g__Erysipelotrichaceae_UCG-002	2.46E-05	4.14E-09	1.31E-05	8.20E-07
g__Frankia	1.23E-06	3.63E-11	1.23E-06	2.87E-05
g__Actinoallomurus	0	0	0	2.46E-05

g__Paraeggerthella	1.97E-05	9.28E-09	1.97E-05	0
g__ASF356	0	0	0	2.29E-05
g__Myroides	0	0	0	2.29E-05
g__Arsenicicoccus	0	0	0	2.29E-05
g__Franconibacter	0	0	0	2.38E-05
g__A2	0	0	0	2.38E-05
g__Marinobacterium	0	0	0	2.38E-05
g__Friedmanniella	2.70E-05	1.04E-08	2.09E-05	2.46E-06
g__Allobaculum	1.23E-06	3.63E-11	1.23E-06	2.70E-05
g__Chroococcidiopsis_PCC_7203	0	0	0	2.21E-05
g__Pseudogulbenkiania	0	0	0	2.21E-05
g__Alkalibacillus	0	0	0	2.21E-05
g__Haliea	0	0	0	2.21E-05
g__Rahnella1	1.23E-06	3.63E-11	1.23E-06	2.54E-05
g__GOUTA6	0	0	0	2.13E-05
g__IMCC26207	0	0	0	2.13E-05
g__Truepera	2.34E-05	1.31E-08	2.34E-05	1.64E-06
g__Halanaerobium	1.72E-05	7.11E-09	1.72E-05	0
g__Limnobacter	1.23E-06	3.63E-11	1.23E-06	2.46E-05
g__Pseudokineococcus	1.23E-06	3.63E-11	1.23E-06	2.46E-05
g__Polycyclovorans	1.23E-06	3.63E-11	1.23E-06	2.46E-05
g__Azohydromonas	1.23E-06	3.63E-11	1.23E-06	2.46E-05
g__Candidatus_Nitrosopumilus	0	0	0	2.05E-05
g__Cerasibacillus	0	0	0	2.05E-05
g__Agarivorans	0	0	0	2.05E-05
g__Oikopleura	2.46E-06	1.45E-10	2.46E-06	2.70E-05
g__Coxiella	2.46E-06	1.45E-10	2.46E-06	2.70E-05
g__Tsukamurella	1.60E-05	2.65E-09	1.05E-05	0
g__Peptoclostridium	0	0	0	1.97E-05
g__Sphaerotilus	0	0	0	1.97E-05
g__Defluviimonas	0	0	0	1.97E-05
g__Ignavigranum	0	0	0	1.97E-05
g__Acetatifactor	0	0	0	1.97E-05
g__Amphibacillus	0	0	0	1.97E-05
g__Sulfurifustis	0	0	0	1.97E-05
g__Lewinella	0	0	0	1.97E-05
g__Vogesella	0	0	0	1.88E-05
g__Holdemania	2.70E-05	4.08E-09	1.30E-05	4.10E-06
g__Lachnospiraceae_UCG-003	1.23E-06	3.63E-11	1.23E-06	2.21E-05
g__Ahniella	1.23E-06	3.63E-11	1.23E-06	2.21E-05
g__Prevotellaceae_NK3B31_group	0	0	0	1.80E-05
g__Sphingopyxis	3.69E-06	1.75E-10	2.70E-06	2.79E-05
g__Smithella	0	0	0	1.64E-05
g__Desulfuromonas	0	0	0	1.64E-05

g__Muricauda	0	0	0	1.64E-05
g__Tepidanaerobacter	0	0	0	1.64E-05
g__Carnobacterium	0	0	0	1.72E-05
g__Leptospirillum	0	0	0	1.72E-05
g__Thermoclostridium	0	0	0	1.56E-05
g__Rosenbergiella	0	0	0	1.56E-05
g__Methylonatrum	0	0	0	1.56E-05
g__Geminicoccus	0	0	0	1.56E-05
g__AKYG587	3.69E-06	3.26E-10	3.69E-06	2.62E-05
g__Fibrobacter	1.23E-06	3.63E-11	1.23E-06	1.88E-05
g__Jannaschia	0	0	0	1.48E-05
g__Clostridium_sensu_stricto_13	0	0	0	1.48E-05
g__Tissierella	0	0	0	1.48E-05
g__Proteocatella	0	0	0	1.48E-05
g__Kibdelosporangium	0	0	0	1.48E-05
g__Candidatus_Soleaferrea	1.97E-05	2.55E-09	1.03E-05	2.46E-06
g__Williamsia	0	0	0	1.39E-05
g__Limnochorda	0	0	0	1.39E-05
g__Kaistia	0	0	0	1.39E-05
g__Cerasicoccus	0	0	0	1.39E-05
g__Halioglobus	0	0	0	1.39E-05
g__Anaeroplasma	1.23E-06	3.63E-11	1.23E-06	1.72E-05
g__SM1A02	1.23E-06	3.63E-11	1.23E-06	1.72E-05
g__Methylotenera	1.23E-06	3.63E-11	1.23E-06	1.72E-05
g__Syntrophococcus	1.35E-05	2.12E-09	9.39E-06	8.20E-07
g__Cognatishimia	0	0	0	1.31E-05
g__Ileibacterium	0	0	0	1.31E-05
g__JTB215	0	0	0	1.31E-05
g__Sulfuriferula	0	0	0	1.31E-05
g__Arenibacter	0	0	0	1.31E-05
g__Shuttleworthia	2.46E-06	1.45E-10	2.46E-06	1.97E-05
g__Actinoplanes	6.15E-06	9.07E-10	6.15E-06	2.62E-05
g__Erysipelothrix	2.46E-06	1.45E-10	2.46E-06	1.88E-05
g__CL500-3	0	0	0	1.23E-05
g__Ekhidna	0	0	0	1.23E-05
g__Helicobacter	0.005794686	9.60E-05	0.00200005	0.351432814
g__Subdoligranulum	0.014806319	0.000691851	0.005369089	0.000608069
g__Fusobacterium	0.116170295	0.029237879	0.034903366	0.005815173
g__Bacteroides	0.126503373	0.012597974	0.022911036	0.005204646
g__Faecalibacterium	0.040732436	0.0031585	0.011471886	0.001924186
g__Parabacteroides	0.004779325	3.42E-05	0.001194526	0.000621181
g__Erysipelotrichaceae_UCG-003	0.000735092	1.43E-06	0.000243694	1.56E-05
g__Phascolarctobacterium	0.001193602	2.79E-06	0.000340701	4.02E-05
g__Collinsella	0.009568484	0.000397435	0.004069372	0.000267157

g__Ureaplasma	0.001127222	2.74E-05	0.001069396	0
g__[Ruminococcus]_torques_group	0.013606571	0.000581926	0.004924114	0.000438433
g__Tyzzerella	0.001585733	1.58E-05	0.000810551	3.85E-05
g__Acidaminococcus	0.000631835	4.71E-06	0.000443007	1.64E-06
g__Dorea	0.004462178	2.82E-05	0.001084452	0.000272894
g__Lachnospiraceae_UCG-010	0.00414872	3.85E-05	0.001266388	0.000172915
g__Coprococcus	0.003261201	1.65E-05	0.000828363	0.000252406
g__Acidithiobacillus	0	0	0	8.69E-05
g__Exiguobacterium	3.69E-06	9.93E-11	2.03E-06	0.000241753
g__Eggerthella	0.001322673	7.65E-06	0.00056473	4.59E-05
g__Sinibacillus	0	0	0	0.000404014
g__Bilophila	0.001264899	4.82E-06	0.000448351	9.18E-05
g__UCG-003	0.000469574	6.24E-07	0.000161286	3.44E-05
g__Faecalibaculum	0	0	0	8.36E-05
g__Johnsonella	0	0	0	8.60E-05
g__Enterorhabdus	1.60E-05	2.95E-09	1.11E-05	0.000170456
g__Muribaculum	0	0	0	5.41E-05
g__Candidatus_Saccharimonas	1.23E-06	3.63E-11	1.23E-06	6.39E-05
g__Pseudogracilibacillus	0	0	0	0.000218807
g__Epulopiscium	0.000333127	1.14E-06	0.000218371	0
g__Mobiluncus	0.0001807	7.62E-07	0.000178151	0
g__Clostridium_sensu_stricto_2	0.000116779	3.06E-07	0.000112991	0
g__SP3-e08	0.000109403	2.81E-07	0.000108128	0
g__Candidatus_Nitrosopelagicus	0	0	0	6.72E-05
g__Microbacterium	0	0	0	9.51E-05
g__Caldicoprobacter	0	0	0	7.70E-05
g__Ezakiella	9.10E-05	1.82E-07	8.72E-05	0
g__Coprobacillus	3.93E-05	2.46E-08	3.20E-05	0
g__SUP05_cluster	0	0	0	3.03E-05
g__Merdibacter	7.38E-05	8.54E-08	5.96E-05	0
g__Oceanobacillus	0	0	0	7.46E-05
g__Lachnospiraceae_NC2004_group	3.56E-05	5.52E-09	1.52E-05	0
g__Dielma	5.29E-05	4.09E-08	4.13E-05	0
g__JTB255_marine_benthic_group	0	0	0	0.000118828
g__unidentified_Nitrospiraceae	0	0	0	7.21E-05
g__Spirosoma	4.06E-05	3.71E-08	3.93E-05	0
g__Defluviicoccus	3.07E-05	2.08E-08	2.95E-05	0
g__Pleionea	3.07E-05	1.75E-08	2.70E-05	0
g__Flavitalea	0	0	0	5.33E-05
g__Marinobacter	0	0	0	4.02E-05
g__Marivita	0	0	0	4.59E-05
g__Jeotgalicoccus	2.46E-06	1.45E-10	2.46E-06	1.80E-05
g__Aminobacter	0	0	0	1.15E-05
g__Maribacter	0	0	0	1.15E-05

g__Actinomadura	1.23E-06	3.63E-11	1.23E-06	1.48E-05
g__Methanothermobacter	1.23E-06	3.63E-11	1.23E-06	1.48E-05
g__Butyrivibrio	4.92E-06	5.80E-10	4.92E-06	2.21E-05
g__Jonquetella	8.60E-06	1.32E-09	7.42E-06	0
g__Tepidimicrobium	1.23E-06	3.63E-11	1.23E-06	0.000518744
g__Coprobacter	0.000334356	1.10E-06	0.000214173	8.20E-07
g__Erythrobacter	1.23E-06	3.63E-11	1.23E-06	5.49E-05
g__Paraclostridium	0	0	0	9.75E-05
g__Luteitalea	1.23E-06	3.63E-11	1.23E-06	0.000149969
g__IS-44	1.23E-06	3.63E-11	1.23E-06	0.000143413
g__Sandaracinus	0	0	0	7.13E-05
g__Cellulosilyticum	0	0	0	9.83E-06
g__Proteiniclasticum	0	0	0	9.83E-06
g__Thermus	0	0	0	9.83E-06
g__Pseudarcobacter	0	0	0	9.83E-06
g__Garciella	0	0	0	9.83E-06
g__Anaerosalibacter	0	0	0	9.83E-06
g__Ureibacillus	0	0	0	9.83E-06
g__Vicingus	0	0	0	9.83E-06
g__Psychrobacillus	0	0	0	9.83E-06
g__Nocardiopsis	1.23E-06	3.63E-11	1.23E-06	1.31E-05
g__Caenimonas	1.23E-06	3.63E-11	1.23E-06	1.31E-05
g__Methylomonas	1.23E-06	3.63E-11	1.23E-06	1.31E-05
g__Chujaibacter	1.23E-06	3.63E-11	1.23E-06	1.39E-05
g__Pseudoramibacter	1.23E-06	3.63E-11	1.23E-06	1.39E-05
g__Lachnospiraceae_XPB1014_group	0	0	0	1.07E-05
g__Cytophaga	0	0	0	1.07E-05
g__Anaerovorax	0	0	0	1.07E-05
g__Neptuniibacter	0	0	0	1.07E-05
g__Fusicatenibacter	0.001355863	8.09E-06	0.000580621	8.85E-05
g__Haloplasma	0	0	0	0.00011473
g__Pseudoalteromonas	0	0	0	0.000167178
g__Sva0996_marine_group	0	0	0	6.15E-05
g__Bdellovibrio	1.23E-06	3.63E-11	1.23E-06	0.000108174
g__Sumerlaea	0	0	0	3.03E-05
g__Peredibacter	6.15E-06	6.04E-10	5.02E-06	6.56E-05
g__Nocardia	0	0	0	4.10E-05
g__Nesterenkonia	2.46E-06	6.94E-11	1.70E-06	1.56E-05
g__MWH-Ta3	6.15E-06	9.07E-10	6.15E-06	2.29E-05
g__Promicromonospora	9.83E-06	2.32E-09	9.83E-06	8.20E-07
g__Escherichia-Shigella	0.070395474	0.013322549	0.023560692	0.011049321
g__[Eubacterium]_siraeum_group	4.92E-06	3.53E-10	3.84E-06	6.47E-05
g__UCG-002	0.002399496	1.05E-05	0.000661794	0.000403194

g__Streptobacillus	0.001223104	3.24E-05	0.00116172	2.46E-06
g__Sorangium	0	0	0	9.01E-06
g__[Bacteroides]_pectinophilus_group	0	0	0	9.01E-06
g__Murdochiella	0	0	0	9.01E-06
g__Negativicoccus	0	0	0	9.01E-06
g__Hellea	0	0	0	9.01E-06
g__Reinekea	0	0	0	9.01E-06
g__Oligoflexus	9.83E-06	2.32E-09	9.83E-06	2.87E-05
g__1959-1	1.35E-05	4.39E-09	1.35E-05	2.46E-06
g__Lachnoclostridium	0.005309132	6.77E-05	0.001678921	0.000634293
g__Butyricicoccus	0.001253835	1.01E-05	0.000647407	0.000154066
g__Ellin516	1.23E-06	3.63E-11	1.23E-06	5.24E-05
g__Tannerella	7.38E-06	3.22E-10	3.66E-06	2.38E-05
g__Hyphomonas	6.15E-06	9.07E-10	6.15E-06	2.21E-05
g__[Eubacterium]_hallii_group	0.000864163	1.68E-06	0.000264807	0.000158164
g__Nitrosomonas	0	0	0	0.000276172
g__Rhodanobacter	2.46E-06	1.45E-10	2.46E-06	5.08E-05
g__Iamia	4.92E-06	5.80E-10	4.92E-06	0.000107355
g__Rhizobacter	6.15E-06	6.04E-10	5.02E-06	7.46E-05
g__Thermomonas	9.83E-06	1.79E-09	8.64E-06	2.70E-05
g__Thermosporothrix	2.09E-05	1.05E-08	2.09E-05	6.56E-06
g__Ilumatobacter	2.95E-05	1.21E-08	2.25E-05	0.000272894
g__Steroidobacter	3.32E-05	2.64E-08	3.32E-05	0.001325132
g__Candidatus_Entotheonella	1.23E-06	3.63E-11	1.23E-06	7.87E-05
g__Acidibacillus	0	0	0	8.20E-06
g__Duganella	0	0	0	8.20E-06
g__BIyi10	0	0	0	8.20E-06
g__Sva0081_sediment_group	0	0	0	8.20E-06
g__Bhargavaea	0	0	0	8.20E-06
g__Marimicrobium	0	0	0	8.20E-06
g__Colwellia	0	0	0	8.20E-06
g__Nitrospina	0	0	0	8.20E-06
g__Blautia	0.013639761	0.00070001	0.005400657	0.001912713
g__Megamonas	0.000464657	7.04E-07	0.000171265	5.41E-05
g__Citrobacter	0.002780564	1.90E-05	0.000889732	0.000441711
g__Halocella	0	0	0	0.000243392
g__Altererythrobacter	6.15E-06	6.04E-10	5.02E-06	7.79E-05
g__Pseudopropionibacterium	2.46E-06	6.94E-11	1.70E-06	1.39E-05
g__Acinetobacter	0.008228601	0.000164045	0.002614422	0.001734882
g__Rhodoplanes	2.34E-05	8.25E-09	1.85E-05	0.000283547
g__Bordetella	1.11E-05	2.94E-09	1.11E-05	1.64E-06
g__Leuconostoc	6.15E-06	3.01E-10	3.54E-06	0.000957996
g__Sutterella	0.002891197	6.74E-05	0.001675308	0.000136037

g__Ruegeria	0	0	0	0.000122925
g__Ignatzschineria	0	0	0	4.18E-05
g__SD04E11	6.15E-06	4.53E-10	4.34E-06	0
g__Catenulispora	6.15E-06	9.07E-10	6.15E-06	0
g__Cnuella	6.15E-06	9.07E-10	6.15E-06	0
g__Lactivibrio	6.15E-06	6.04E-10	5.02E-06	0
g__Klebsiella	0.103345527	0.016130247	0.025924769	0.029057018
g__Butyricimonas	0.000658878	1.91E-06	0.000282263	3.77E-05
g__Agathobacter	0.004802681	8.54E-05	0.001885831	0.000720341
g__Roseburia	0.004110613	5.78E-05	0.001551805	0.000634293
g__Haliangium	0.000126613	1.65E-07	8.28E-05	0.000657239
g__Mycobacterium	3.93E-05	3.06E-08	3.57E-05	0.000447447
g__Chryseolinea	2.46E-05	1.45E-08	2.46E-05	0.000998151
g__Bryobacter	0.000113091	2.17E-07	9.52E-05	0.000769511
g__Erysipelatoclostridium	0.001212041	3.26E-06	0.000368552	0.000177832
g__Centipeda	4.92E-05	1.25E-08	2.29E-05	0.000832612
g__Terrimonas	1.84E-05	6.19E-09	1.61E-05	0.00021389
g__SH3-11	3.69E-06	3.26E-10	3.69E-06	0.000127842
g__Streptosporangium	2.46E-06	1.45E-10	2.46E-06	4.84E-05
g__Chryseomicrobium	4.92E-06	5.80E-10	4.92E-06	1.72E-05
g__Rikenella	0	0	0	7.38E-06
g__Desulfobulbus	0	0	0	7.38E-06
g__Herbinix	0	0	0	7.38E-06
g__AUTHM297	0	0	0	7.38E-06
g__unidentified_HOC36	0	0	0	7.38E-06
g__HSB_OF53-F07	0	0	0	7.38E-06
g__Aliidiomarina	0	0	0	7.38E-06
g__Trichloromonas	0	0	0	7.38E-06
g__Savagea	0	0	0	7.38E-06
g__Piscibacillus	0	0	0	7.38E-06
g__Flexithrix	0	0	0	7.38E-06
g__Catonella	6.02E-05	1.56E-08	2.55E-05	0.000828515
g__Woeseia	0	0	0	0.000472032
g__Nakamurella	7.38E-06	6.24E-10	5.10E-06	0.000127023
g__Adhaeribacter	7.38E-06	7.00E-10	5.40E-06	6.64E-05
g__Pediococcus	3.69E-06	3.26E-10	3.69E-06	1.48E-05
g__[Eubacterium]_saphenum_group	3.69E-06	1.75E-10	2.70E-06	1.56E-05
g__Candidatus_Actinomarina	3.69E-06	3.26E-10	3.69E-06	1.56E-05
g__Inquilinus	3.69E-06	3.26E-10	3.69E-06	1.56E-05
g__Dinghuibacter	3.93E-05	1.97E-08	2.86E-05	0.000417126
g__Phaselicystis	7.38E-06	1.31E-09	7.38E-06	0.000204056
g__Phenylobacterium	3.32E-05	8.80E-09	1.92E-05	0.000231099
g__ADurb.Bin063-1	7.38E-06	1.31E-09	7.38E-06	0.000122106
g__Jatrophihabitans	9.83E-06	8.83E-10	6.07E-06	0.000124564

g__Ellin6055	2.46E-05	1.05E-08	2.09E-05	0.000204875
g__TM7x	3.69E-06	3.26E-10	3.69E-06	4.67E-05
g__Filifactor	3.69E-06	9.93E-11	2.03E-06	3.44E-05
g__P3OB-42	2.21E-05	1.05E-08	2.09E-05	0.000347468
g__Mycoplasma	2.46E-06	1.45E-10	2.46E-06	9.10E-05
g__Hirschia	2.58E-05	9.94E-09	2.03E-05	0.000547426
g__Lachnospiraceae_UCG-006	3.69E-06	1.75E-10	2.70E-06	8.03E-05
g__Lachnospiraceae_UCG-004	9.83E-06	7.32E-10	5.52E-06	1.64E-06
g__unidentified_Marinococcaceae	6.15E-06	9.07E-10	6.15E-06	1.88E-05
g__Quadrisphaera	8.60E-06	6.42E-10	5.17E-06	0.000473671
g__Pseudoxanthomonas	7.38E-06	7.00E-10	5.40E-06	5.41E-05
g__Agromyces	2.46E-06	6.94E-11	1.70E-06	6.80E-05
g__Clostridium_sensu_stricto_7	8.60E-06	4.90E-10	4.52E-06	7.79E-05
g__unidentified_P5D1-392	1.23E-06	3.63E-11	1.23E-06	9.83E-06
g__Lacunisphaera	1.23E-06	3.63E-11	1.23E-06	9.83E-06
g__Aridibacter	1.23E-06	3.63E-11	1.23E-06	9.83E-06
g__KD3-10	1.23E-06	3.63E-11	1.23E-06	9.83E-06
g__Pasteuria	1.23E-06	3.63E-11	1.23E-06	9.83E-06
g__Bacillus	0.000660107	6.96E-06	0.000538633	0.005879094
g__MND1	0.000113091	2.43E-07	0.000100575	0.000860475
g__Acanthopleuribacter	0	0	0	8.52E-05
g__Thalassotalea	0	0	0	8.03E-05
g__Motilimonas	0	0	0	4.51E-05
g__Kiloniella	0	0	0	4.51E-05
g__Parvularcula	0	0	0	4.59E-05
g__Aliikangiella	0	0	0	3.11E-05
g__Taibaiella	1.35E-05	4.39E-09	1.35E-05	4.10E-06
g__Parasutterella	0.002105706	1.35E-05	0.000750414	0.00024667
g__Prevotellaceae_UCG-001	1.72E-05	1.89E-09	8.86E-06	0.000145871
g__Methanosaeta	0	0	0	6.15E-05
g__[Ruminococcus]_gnavus_group	0.005123515	0.00010259	0.00206751	0.000481866
g__Anaerococcus	0.000614625	1.20E-06	0.000223355	8.52E-05
g__Synechococcus_CC9902	0	0	0	6.56E-06
g__Longispora	0	0	0	6.56E-06
g__Sulfitobacter	0	0	0	6.56E-06
g__Chiayiivirga	0	0	0	6.56E-06
g__Saccharomonospora	0	0	0	6.56E-06
g__Dethiobacter;	0	0	0	6.56E-06
g__Candidatus_Microthrix	4.92E-06	5.80E-10	4.92E-06	0
g__Fimbriiglobus	4.92E-06	3.53E-10	3.84E-06	0
g__Blastomonas	0.000109403	2.59E-08	3.29E-05	0.000500715
g__Family_XIII_AD3011_group	0.000381068	5.73E-07	0.000154576	5.00E-05
g__SWB02	4.67E-05	5.24E-08	4.67E-05	0.000676088
g__Cloacibacterium	0	0	0	0.000121286

g__Lachnospiraceae_FCS020_group	0.000145052	1.08E-07	6.72E-05	1.23E-05
g__Bauldia	5.90E-05	8.00E-08	5.77E-05	0.000397458
g__Alteromonas	0	0	0	0.000143413
g__Thermobacillus	1.23E-06	3.63E-11	1.23E-06	3.85E-05
g__Roseimicrobium	4.92E-06	5.80E-10	4.92E-06	0.000107355
g__Hymenobacter	2.46E-06	1.45E-10	2.46E-06	8.52E-05
g__JGI_0001001-H03	3.44E-05	2.28E-08	3.08E-05	0.000546607
g__Clostridium_sensu_stricto_1	0.009358282	0.001239671	0.007186999	0.000533495
g__Candidatus_Xiphinematobacter	6.02E-05	8.71E-08	6.02E-05	0.00157426
g__Crossiella	1.72E-05	7.11E-09	1.72E-05	0.0001639
g__Ellin517	4.92E-06	5.80E-10	4.92E-06	3.93E-05
g__Blastocatella	1.48E-05	5.22E-09	1.48E-05	4.92E-06
g__Ralstonia	0.000451135	1.33E-06	0.000235297	0.008767013
g__Edaphobaculum	1.48E-05	5.22E-09	1.48E-05	0.000121286
g__Eikenella	0.000191763	6.73E-07	0.000167483	8.20E-06
g__Pedomicrobium	0.000192992	8.94E-07	0.000192992	0.001634083
g__Litorimicrobium	0	0	0	0.000219626
g__Labrys	4.92E-06	5.80E-10	4.92E-06	3.44E-05
g__UCG-009	2.34E-05	3.03E-09	1.12E-05	1.07E-05
g__Alkanindiges	2.34E-05	5.45E-09	1.51E-05	1.07E-05
g__TM7a	1.11E-05	2.94E-09	1.11E-05	0.000106535
g__Luedemannella	4.92E-06	2.02E-10	2.90E-06	1.56E-05
g__[Ruminococcus]_gauvreauii_group	0.000226182	2.34E-07	9.87E-05	3.36E-05
g__Roseisolibacter	0	0	0	7.62E-05
g__Polyangium	3.69E-06	3.26E-10	3.69E-06	2.95E-05
g__[Eubacterium]_brachy_group	1.11E-05	5.91E-10	4.96E-06	2.46E-05
g__mle1-7	4.67E-05	3.15E-08	3.62E-05	0.00032862
g__EBM-39	8.60E-06	1.78E-09	8.60E-06	1.64E-06
g__Methylobacterium-Methylorubrum	9.96E-05	8.05E-08	5.79E-05	0.000461379
g__Arcobacter	2.46E-06	1.45E-10	2.46E-06	1.07E-05
g__Providencia	2.46E-06	1.45E-10	2.46E-06	1.07E-05
g__Citrifermentans	2.46E-06	1.45E-10	2.46E-06	1.07E-05
g__Amphiplicatus	2.46E-06	1.45E-10	2.46E-06	3.03E-05
g__Paenarthrobacter	2.46E-06	1.45E-10	2.46E-06	1.15E-05
g__Sulfuricurvum	2.46E-06	1.45E-10	2.46E-06	1.15E-05
g__CAG-352	6.15E-05	6.98E-08	5.39E-05	8.20E-07
g__[Clostridium]_innocuum_group	0.001188685	1.27E-05	0.00072871	4.92E-05
g__Alcaligenes	1.72E-05	4.08E-09	1.30E-05	0.000146691
g__Brochothrix	0.007508261	0.00127833	0.007298201	5.65E-05
g__Anaerostipes	0.000910874	5.97E-06	0.000498663	0.000120467
g__Candidatus_Solibacter	0.000138905	4.46E-07	0.00013635	0.00080311
g__Defluviitaleaceae_UCG-011	4.92E-06	2.78E-10	3.40E-06	3.61E-05

g__Eubacterium	1.11E-05	1.20E-09	7.06E-06	3.28E-06
g__Moryella	0.001696365	4.85E-05	0.001421826	3.11E-05
g__Hydrogenoanaerobacterium	0	0	0	5.74E-06
g__Candidatus_Stoquefichus	0	0	0	5.74E-06
g__Mailhella	0	0	0	5.74E-06
g__RS62_marine_group	0	0	0	5.74E-06
g__Pelagibacterium	0	0	0	5.74E-06
g__Roseiarcus	0	0	0	5.74E-06
g__Aneurinibacillus	0	0	0	5.74E-06
g__Candidatus_Nitrososphaera	0	0	0	5.74E-06
g__Desulfohalotomaculum	0	0	0	5.74E-06
g__Fervidobacterium	0	0	0	5.74E-06
g__Denitromonas	0	0	0	5.74E-06
g__MSB-1D1	0	0	0	5.74E-06
g__Cm1-21	0	0	0	5.74E-06
g__LS-NOB	0	0	0	5.74E-06
g__Aetherobacter	0	0	0	5.74E-06
g__Desulfobacca	0	0	0	5.74E-06
g__Pedosphaera	0	0	0	5.74E-06
g__Novosphingobium	0	0	0	5.74E-06
g__Paenalcaligenes	0	0	0	5.74E-06
g__Pirellula	0	0	0	5.74E-06
g__Acidipila	0	0	0	5.74E-06
g__Litorilinea	2.09E-05	1.05E-08	2.09E-05	0.000140135
g__Lentimicrobium	2.46E-05	4.59E-09	1.38E-05	0.000504812
g__Proteus	0.000215119	5.16E-07	0.00014669	4.10E-06
**GC.Tumor-GC.Para-Genus**				
g__Litoreibacter	0	0	0	2.77E-05
g__Clostridium_sensu_stricto_18	2.87E-05	1.01E-08	1.67E-05	0
g__Tepidiphilus	2.87E-05	2.96E-08	2.87E-05	0
g__Gelria	2.62E-05	2.48E-08	2.62E-05	0
g__Litoribrevibacter	2.62E-05	5.91E-09	1.28E-05	0
g__Arcticibacter	0	0	0	2.32E-05
g__Clostridium_sensu_stricto_14	0	0	0	2.24E-05
g__Myroides	2.29E-05	1.90E-08	2.29E-05	0
g__Arsenicicoccus	2.29E-05	1.90E-08	2.29E-05	0
g__Alkalibacillus	2.21E-05	8.72E-09	1.56E-05	0
g__Papillibacter	1.64E-06	4.70E-11	1.14E-06	2.77E-05
g__Friedmanniella	2.46E-06	2.18E-10	2.46E-06	2.86E-05
g__Thermanaerovibrio	1.64E-06	9.67E-11	1.64E-06	2.68E-05
g__Cerasibacillus	2.05E-05	7.45E-09	1.44E-05	0
g__Agarivorans	2.05E-05	3.47E-09	9.82E-06	0
g__Oikopleura	2.70E-05	7.53E-09	1.45E-05	1.79E-06
g__Ignavigranum	1.97E-05	1.39E-08	1.97E-05	0

g__Succiniclasticum	2.46E-06	1.18E-10	1.81E-06	2.68E-05
g__Pseudogulbenkiania	2.21E-05	8.22E-09	1.51E-05	8.94E-07
g__Succinivibrionaceae_UCG-002	0	0	0	1.70E-05
g__Desulfurivibrio	0	0	0	1.61E-05
g__NS2b_marine_group	1.64E-06	9.67E-11	1.64E-06	2.15E-05
g__Fermentimonas	3.28E-06	2.38E-10	2.57E-06	2.50E-05
g__Thermoclostridium	1.56E-05	8.73E-09	1.56E-05	0
g__Rosenbergiella	1.56E-05	7.83E-09	1.48E-05	0
g__Auricoccus-Abyssicoccus	1.64E-06	9.67E-11	1.64E-06	2.06E-05
g__Propioniciclava	0	0	0	1.43E-05
g__Tissierella	1.48E-05	3.26E-09	9.51E-06	0
g__Proteocatella	1.48E-05	2.26E-09	7.93E-06	0
g__NS5_marine_group	4.10E-06	3.06E-10	2.92E-06	2.50E-05
g__Cerasicoccus	1.39E-05	1.76E-09	7.00E-06	0
g__Tychonema_CCAP_1459-11B	4.92E-06	4.72E-10	3.62E-06	2.59E-05
g__Rodentibacter	0	0	0	1.25E-05
g__Formosa	0	0	0	1.25E-05
g__Thermomonas	2.70E-05	1.78E-08	2.23E-05	5.36E-06
g__Fournierella	1.31E-05	2.46E-09	8.26E-06	0
g__JTB215	1.31E-05	4.15E-09	1.07E-05	0
g__Sulfuriferula	1.31E-05	2.16E-09	7.75E-06	0
g__Azohydromonas	2.46E-05	3.06E-09	9.22E-06	4.47E-06
g__Amphibacillus	1.97E-05	3.43E-09	9.76E-06	2.68E-06
g__Solitalea	5.74E-06	4.39E-10	3.49E-06	2.59E-05
g__Fastidiosipila	2.21E-05	1.28E-08	1.89E-05	3.58E-06
g__Thermoanaerobaculum	8.20E-07	2.42E-11	8.20E-07	1.43E-05
g__Kibdelosporangium	1.48E-05	1.72E-09	6.90E-06	8.94E-07
g__Vulcaniibacterium	6.56E-06	6.02E-10	4.09E-06	2.68E-05
g__Truepera	1.64E-06	9.67E-11	1.64E-06	1.61E-05
g__[Anaerorhabdus]_furcosa_group	0	0	0	1.07E-05
g__Muricauda	1.64E-05	4.45E-09	1.11E-05	1.79E-06
g__Pseudopropionibacterium	1.39E-05	2.21E-09	7.84E-06	8.94E-07
g__Halioglobus	1.39E-05	1.72E-09	6.90E-06	8.94E-07
g__Chryseomicrobium	1.72E-05	1.61E-09	6.69E-06	2.68E-06
g__AKYG587	2.62E-05	5.11E-09	1.19E-05	7.15E-06
g__Nevskia	4.10E-06	3.06E-10	2.92E-06	1.97E-05
g__Actinospica	4.10E-06	4.05E-10	3.36E-06	1.97E-05
g__GOUTA6	2.13E-05	1.40E-08	1.97E-05	4.47E-06
g__Garciella	9.83E-06	2.49E-09	8.31E-06	0
g__Ureibacillus	9.83E-06	1.34E-09	6.11E-06	0
g__Ekhidna	1.23E-05	1.81E-09	7.09E-06	8.94E-07
g__Propionibacterium	0	0	0	8.94E-06
g__Microcoleus_SAG_1449-1a	0	0	0	8.94E-06
g__CAG-352	8.20E-07	2.42E-11	8.20E-07	1.16E-05

g__Blastocatella	4.92E-06	8.70E-10	4.92E-06	2.06E-05
g__Buchnera	3.28E-06	2.38E-10	2.57E-06	1.79E-05
g__Crocinitomix	3.28E-06	2.38E-10	2.57E-06	1.79E-05
g__Haliea	2.21E-05	5.89E-09	1.28E-05	5.36E-06
g__Paludicola	1.64E-06	4.70E-11	1.14E-06	1.34E-05
g__Zoogloea	1.64E-06	4.70E-11	1.14E-06	1.34E-05
g__NS4_marine_group	0	0	0	5.90E-05
g__Clostridium_sensu_stricto_11	0	0	0	3.93E-05
g__[Bacteroides]_pectinophilus_group	9.01E-06	2.93E-09	9.01E-06	0
g__Reinekea	9.01E-06	9.36E-10	5.10E-06	0
g__Pyramidobacter	4.92E-06	6.22E-10	4.16E-06	1.97E-05
g__C39	4.92E-06	8.70E-10	4.92E-06	1.97E-05
g__Acetatifactor	1.97E-05	7.21E-09	1.42E-05	4.47E-06
g__Carnobacterium	1.72E-05	2.70E-09	8.67E-06	3.58E-06
g__Sporanaerobacter	8.20E-07	2.42E-11	8.20E-07	1.07E-05
g__Pseudophaeobacter	8.20E-07	2.42E-11	8.20E-07	1.07E-05
g__Sphaerochaeta	0	0	0	8.05E-06
g__Bulleidia	0	0	0	8.05E-06
g__Halobacillus	0	0	0	8.05E-06
g__Streptobacillus	2.46E-06	1.18E-10	1.81E-06	1.43E-05
g__Asticcacaulis	2.46E-06	2.18E-10	2.46E-06	1.43E-05
g__Atopostipes	4.10E-06	2.56E-10	2.67E-06	4.29E-05
g__Citrifermentans	1.07E-05	2.49E-09	8.32E-06	8.94E-07
g__Neptuniibacter	1.07E-05	1.25E-09	5.89E-06	8.94E-07
g__Sporolactobacillus	8.20E-06	8.76E-10	4.93E-06	0
g__Bhargavaea	8.20E-06	1.27E-09	5.95E-06	0
g__Marimicrobium	8.20E-06	1.07E-09	5.46E-06	0
g__Nitrospina	8.20E-06	9.75E-10	5.21E-06	0
g__Verticiella	8.20E-07	2.42E-11	8.20E-07	9.83E-06
g__UCG-009	1.07E-05	1.10E-09	5.53E-06	2.86E-05
g__Dyella	2.54E-05	5.03E-09	1.18E-05	8.94E-06
g__Epulopiscium	0	0	0	7.15E-06
g__Agaricicola	0	0	0	7.15E-06
g__Leptospira	0	0	0	7.15E-06
g__Sedimentibacter	0	0	0	7.15E-06
g__AAP99	0	0	0	7.15E-06
g__Acetomicrobium	0	0	0	7.15E-06
g__Lewinella	1.97E-05	3.28E-09	9.55E-06	5.36E-06
g__Hydrogenispora	1.39E-05	2.16E-09	7.75E-06	2.68E-06
g__Rubellimicrobium	7.38E-06	7.65E-10	4.61E-06	2.24E-05
g__[Eubacterium]_saphenum_group	1.56E-05	4.90E-09	1.17E-05	3.58E-06
g__Motilibacter	2.46E-06	1.18E-10	1.81E-06	1.34E-05
g__Hyphomonas	2.21E-05	3.75E-09	1.02E-05	7.15E-06

g__Proteiniclasticum	9.83E-06	1.14E-09	5.64E-06	8.94E-07
g__Anaerosalibacter	9.83E-06	1.74E-09	6.95E-06	8.94E-07
g__KD3-10	9.83E-06	1.14E-09	5.64E-06	8.94E-07
g__Vicingus	9.83E-06	1.09E-09	5.51E-06	8.94E-07
g__Pasteuria	9.83E-06	5.97E-10	4.07E-06	8.94E-07
g__Savagea	7.38E-06	9.64E-10	5.17E-06	0
g__Piscibacillus	7.38E-06	8.14E-10	4.76E-06	0
g__Sulfuritalea	5.74E-06	4.88E-10	3.68E-06	1.88E-05
g__Maribacter	1.15E-05	1.41E-09	6.25E-06	1.79E-06
g__Ornithinimicrobium	7.38E-06	6.65E-10	4.30E-06	2.15E-05
g__Cohnella	5.33E-05	1.34E-08	1.93E-05	2.68E-06
g__Arenibacter	1.31E-05	1.86E-09	7.19E-06	2.68E-06
g__Methanothermobacter	1.48E-05	7.83E-09	1.48E-05	3.58E-06
g__IMCC26207	2.13E-05	5.05E-09	1.18E-05	7.15E-06
g__Dielma	0	0	0	6.26E-06
g__Vulgatibacter	0	0	0	6.26E-06
g__HN-HF0106	0	0	0	6.26E-06
g__Rickettsia	0	0	0	6.26E-06
g__Tundrisphaera	0	0	0	6.26E-06
g__966-1	2.46E-06	1.18E-10	1.81E-06	1.25E-05
g__Puia	2.38E-05	4.17E-09	1.08E-05	8.94E-06
g__Pseudogracilibacillus	0.000218807	6.18E-07	0.000130977	8.05E-06
g__RS62_marine_group	5.74E-06	1.18E-09	5.74E-06	1.79E-05
g__Minicystis	9.01E-06	1.63E-09	6.73E-06	8.94E-07
g__ADurb.Bin063-1	0.000122106	8.28E-08	4.80E-05	1.43E-05
g__SH3-11	0.000127842	9.88E-08	5.24E-05	4.47E-06
g__Demequina	2.46E-05	3.85E-09	1.03E-05	9.83E-06
g__Allobaculum	2.70E-05	9.07E-09	1.59E-05	1.16E-05
g__Desulfuromonas	1.64E-05	8.73E-09	1.56E-05	4.47E-06
g__Aeromonas	8.77E-05	2.69E-08	2.73E-05	0.000990552
g__Anaerocolumna	6.56E-06	6.02E-10	4.09E-06	0
g__Solimonas	6.56E-06	3.54E-10	3.13E-06	0
g__Pseudoramibacter	1.39E-05	5.50E-09	1.24E-05	3.58E-06
g__Negativibacillus	2.29E-05	4.58E-09	1.13E-05	8.94E-06
g__Rhodoluna	6.56E-06	5.03E-10	3.74E-06	1.88E-05
g__Stenotrophobacter	8.20E-06	5.28E-10	3.83E-06	2.15E-05
g__Tahibacter	3.28E-06	2.38E-10	2.57E-06	1.34E-05
g__Quadrisphaera	0.000473671	6.76E-06	0.000433425	7.15E-06
g__Prevotellaceae_NK3B31_group	1.80E-05	3.69E-09	1.01E-05	0.000158238
g__Petrimonas	1.64E-06	4.70E-11	1.14E-06	9.83E-06
g__Kutzneria	1.64E-06	4.70E-11	1.14E-06	9.83E-06
g__Chryseobacterium	2.46E-06	1.18E-10	1.81E-06	1.16E-05
g__UCG-012	2.46E-06	2.18E-10	2.46E-06	1.16E-05
g__Kurthia	0.001100589	1.52E-05	0.000650084	2.41E-05

g__Prevotellaceae_Ga6A1_group	1.64E-06	9.67E-11	1.64E-06	3.13E-05
g__Chryseolinea	0.000998151	5.20E-06	0.000380206	4.83E-05
g__Phaselicystis	0.000204056	9.03E-07	0.000158405	1.16E-05
g__Dysgonomonas	1.23E-05	1.81E-09	7.09E-06	2.77E-05
g__Leucobacter	0	0	0	5.36E-06
g__CK06-06-Mud-MAS4B-21	0	0	0	5.36E-06
g__CENA518	0	0	0	5.36E-06
g__Sporichthya	0	0	0	5.36E-06
g__Prochlorothrix_PCC-9006	0	0	0	5.36E-06
g__DMER64	0	0	0	5.36E-06
g__Thermobifida	0	0	0	5.36E-06
g__Candidatus_Jidaibacter	0	0	0	5.36E-06
g__Thermophagus	0	0	0	5.36E-06
g__Marivita	4.59E-05	1.40E-08	1.97E-05	3.58E-06
g__Sulfurifustis	1.97E-05	2.69E-09	8.64E-06	7.15E-06
g__Rhizocola	2.79E-05	1.30E-08	1.90E-05	1.25E-05
g__Longispora	6.56E-06	5.53E-10	3.92E-06	1.79E-05
g__Oxalobacter	4.10E-06	3.06E-10	2.92E-06	1.43E-05
g__TM7	6.88E-05	2.72E-08	2.75E-05	6.26E-06
g__Aureimonas	1.64E-06	4.70E-11	1.14E-06	5.27E-05
g__Hydrogenoanaerobacterium	5.74E-06	1.18E-09	5.74E-06	0
g__Pelagibacterium	5.74E-06	1.18E-09	5.74E-06	0
g__Tabrizicola	5.74E-06	8.86E-10	4.96E-06	0
g__Candidatus_Nitrososphaera	5.74E-06	1.18E-09	5.74E-06	0
g__Fervidobacterium	5.74E-06	1.18E-09	5.74E-06	0
g__Desulfobacca	5.74E-06	8.86E-10	4.96E-06	0
g__Paenalcaligenes	5.74E-06	6.87E-10	4.37E-06	0
g__Klebsiella	0.029057018	0.004163649	0.010754391	0.005327347
g__Harryflintia	2.46E-06	1.18E-10	1.81E-06	1.07E-05
g__Paenibacillus	0.000222085	2.03E-07	7.50E-05	4.29E-05
g__Cupriavidus	0.000173734	1.60E-07	6.67E-05	1.43E-05
g__Marinobacterium	2.38E-05	8.20E-09	1.51E-05	1.07E-05
g__Helicobacter	0.351432814	0.12569853	0.059089981	0.18315203
g__Brevibacillus	0.000208973	1.31E-06	0.000190577	1.79E-06
g__P3OB-42	0.000347468	5.59E-07	0.000124558	5.27E-05
g__Steroidobacter	0.001325132	7.82E-06	0.000466022	0.000248532
g__Hirschia	0.000547426	1.45E-06	0.000200797	7.51E-05
g__mle1-7	0.00032862	4.94E-07	0.000117139	5.36E-05
g__Luteitalea	0.000149969	1.21E-07	5.79E-05	2.41E-05
g__Hymenobacter	8.52E-05	1.29E-07	6.00E-05	1.79E-06
g__JGI_0001001-H03	0.000546607	1.65E-06	0.000213816	5.19E-05
g__JTB255_marine_benthic_group	0.000118828	6.84E-08	4.36E-05	2.15E-05
g__RB41	0.00519727	0.000138612	0.001962224	0.000605238
g__Roseimicrobium	0.000107355	6.97E-08	4.40E-05	1.07E-05

g__Coriobacteriaceae_UCG-002	7.38E-06	1.96E-09	7.38E-06	8.94E-07
g__AUTHM297	7.38E-06	1.96E-09	7.38E-06	8.94E-07
g__Flexithrix	7.38E-06	6.15E-10	4.13E-06	8.94E-07
g__Pedomicrobium	0.001634083	1.35E-05	0.000611923	0.000242274
g__Candidatus_Udaeobacter	0.000444989	1.04E-06	0.000169665	4.92E-05
g__Reyranella	0.000620362	1.48E-06	0.000202421	0.000152874
g__Bauldia	0.000397458	5.80E-07	0.000126934	0.000108174
g__Clostridium_sensu_stricto_7	7.79E-05	4.77E-08	3.64E-05	1.07E-05
g__Hellea	9.01E-06	1.63E-09	6.73E-06	1.79E-06
g__Paraprevotella	9.01E-06	6.38E-10	4.21E-06	4.65E-05
g__Ammoniibacillus	0	0	0	4.47E-06
g__Larkinella	0	0	0	4.47E-06
g__Planktosalinus	0	0	0	4.47E-06
g__Catenisphaera	0	0	0	4.47E-06
g__Glycomyces	0	0	0	4.47E-06
g__Planctomicrobium	0	0	0	4.47E-06
g__Candidatus_Xiphinematobacter	0.00157426	1.48E-05	0.000641605	9.92E-05
g__Dongia	0.001239904	7.47E-06	0.000455578	0.000219924
g__Lacihabitans	3.28E-06	3.87E-10	3.28E-06	1.16E-05
g__Brachybacterium	1.39E-05	2.46E-09	8.27E-06	8.14E-05
g__Acidibacter	0.000576928	1.05E-06	0.000170721	0.00016986
g__Coxiella	2.70E-05	5.39E-09	1.22E-05	1.34E-05
g__Candidatus_Soleaferrea	2.46E-06	1.18E-10	1.81E-06	9.83E-06
g__Pseudanabaena_PCC-7429	2.46E-06	2.18E-10	2.46E-06	9.83E-06
g__SWB02	0.000676088	2.26E-06	0.00025069	0.000133206
g__Eikenella	8.20E-06	5.28E-10	3.83E-06	1.88E-05
g__Acidaminococcus	1.64E-06	9.67E-11	1.64E-06	8.05E-06
g__Hamadaea	1.64E-06	9.67E-11	1.64E-06	8.05E-06
g__NS10_marine_group	4.92E-06	8.70E-10	4.92E-06	0
g__Lentibacillus	4.92E-06	4.72E-10	3.62E-06	0
g__Alloiococcus	4.92E-06	3.23E-10	3.00E-06	0
g__Lacunisphaera	9.83E-06	7.46E-10	4.55E-06	2.68E-06
g__Thermus	9.83E-06	1.74E-09	6.95E-06	2.68E-06
g__Fictibacillus	7.38E-06	5.16E-10	3.79E-06	1.70E-05
g__Gracilibacter	7.38E-06	4.17E-10	3.40E-06	1.70E-05
g__Limnochorda	1.39E-05	5.50E-09	1.24E-05	5.36E-06
g__Clostridium_sensu_stricto_13	1.48E-05	1.87E-09	7.20E-06	5.36E-06
g__TM7a	0.000106535	7.52E-08	4.57E-05	1.70E-05
g__Pseudoxanthomonas	5.41E-05	1.03E-08	1.69E-05	1.52E-05
g__IS-44	0.000143413	9.01E-08	5.00E-05	3.49E-05
g__Dethiobacter	6.56E-06	1.20E-09	5.77E-06	8.94E-07
g__Bryobacter	0.000769511	2.04E-06	0.00023822	0.000216348
g__Litorilinea	0.000140135	1.03E-07	5.35E-05	2.32E-05
g__Halarcobacter	3.28E-06	3.87E-10	3.28E-06	1.07E-05

g__Trueperella	3.28E-06	2.38E-10	2.57E-06	1.07E-05
g__Anaerolinea	0.000290923	2.66E-06	0.000271731	1.79E-06
g__Candidatus_Solibacter	0.00080311	2.98E-06	0.000287778	0.000234228
g__Pseudoalteromonas	0.000167178	3.56E-07	9.95E-05	1.70E-05
g__Rhodoplanes	0.000283547	3.32E-07	9.60E-05	8.31E-05
g__Sphingopyxis	2.79E-05	1.32E-08	1.92E-05	1.52E-05
g__Parvibacter	2.46E-06	1.18E-10	1.81E-06	8.94E-06
g__marine_group	2.46E-06	2.18E-10	2.46E-06	8.94E-06
g__Marvinbryantia	1.64E-06	4.70E-11	1.14E-06	0.000101022
g__Vicinamibacter	0.000170456	1.71E-07	6.89E-05	2.41E-05
**CRC.Tumor-CRC.Para-Genus**				
g__Psychroglaciecola	2.83E-05	1.18E-08	2.22E-05	0
g__Aurantisolimonas	0	0	0	2.83E-05
g__Caldalkalibacillus	0	0	0	2.70E-05
g__Friedmanniella	2.70E-05	1.04E-08	2.09E-05	1.23E-06
g__Jannaschia	0	0	0	2.21E-05
g__Methanobacterium	0	0	0	2.09E-05
g__Candidatus_Entotheonella	1.23E-06	3.63E-11	1.23E-06	2.46E-05
g__Anoxybacillus	1.97E-05	4.52E-09	1.37E-05	0
g__A2	0	0	0	1.97E-05
g__Paraeggerthella	1.97E-05	9.28E-09	1.97E-05	0
g__Pseudarcobacter	0	0	0	1.97E-05
g__Luminiphilus	0	0	0	1.84E-05
g__Acetoanaerobium	2.58E-05	1.31E-08	2.34E-05	2.46E-06
g__Erythrobacter	1.23E-06	3.63E-11	1.23E-06	2.21E-05
g__SM1A02	1.23E-06	3.63E-11	1.23E-06	2.21E-05
g__OM60(NOR5) _clade	3.69E-06	3.26E-10	3.69E-06	2.83E-05
g__Flaviflexus	1.72E-05	3.40E-09	1.19E-05	0
g__Faecalibaculum	0	0	0	1.72E-05
g__Halanaerobium	1.72E-05	7.11E-09	1.72E-05	0
g__Truepera	2.34E-05	1.31E-08	2.34E-05	2.46E-06
g__Nitrosomonas	0	0	0	1.60E-05
g__Litorimicrobium	0	0	0	1.60E-05
g__Candidatus_Nitrosopumilus	0	0	0	1.60E-05
g__Leptospirillum	0	0	0	1.60E-05
g__Anaerovibrio	0	0	0	1.60E-05
g__Tsukamurella	1.60E-05	2.65E-09	1.05E-05	0
g__Limnobacter	1.23E-06	3.63E-11	1.23E-06	1.97E-05
g__Ohtaekwangia	4.92E-06	2.78E-10	3.40E-06	2.83E-05
g__Vulcaniibacterium	1.48E-05	4.39E-09	1.35E-05	0
g__Dysgonomonas	1.48E-05	2.57E-09	1.04E-05	0
g__Thermovirga	0	0	0	1.48E-05
g__Blastocatella	1.48E-05	5.22E-09	1.48E-05	0
g__Iodobacter	0	0	0	1.48E-05

g__Maricaulis	0	0	0	1.48E-05
g__Acetobacter	0	0	0	1.48E-05
g__Oligella	1.23E-06	3.63E-11	1.23E-06	1.84E-05
g__unidentified_Rikenellaceae	2.46E-06	1.45E-10	2.46E-06	2.09E-05
g__Thiobacillus	6.15E-06	3.01E-10	3.54E-06	2.83E-05
g__Ruegeria	0	0	0	1.35E-05
g__Negativicoccus	0	0	0	1.35E-05
g__F0332	2.95E-05	1.32E-08	2.34E-05	0
g__Prevotellaceae_Ga6A1_group	0	0	0	4.55E-05
g__Gordonia	0.000674858	1.08E-05	0.000671016	0
g__Deinococcus	8.24E-05	1.58E-07	8.11E-05	0
g__Acidithiobacillus	0	0	0	0.000296249
g__Prevotellaceae_NK3B31_group	0	0	0	5.29E-05
g__Muribaculum	0	0	0	0.000346649
g__Candidatus_Saccharimonas	1.23E-06	3.63E-11	1.23E-06	0.000244621
g__Clade_Ia	0	0	0	9.96E-05
g__Pseudoalteromonas	0	0	0	4.43E-05
g__PMMR1	0.000136447	4.47E-07	0.000136447	0
g__Proteus	0.000215119	5.16E-07	0.00014669	0
g__Rikenella	0	0	0	0.000161032
g__Sva0996_marine_group	0	0	0	5.78E-05
g__Clostridium_sensu_stricto_2	0.000116779	3.06E-07	0.000112991	0
g__Parvibaculum	0	0	0	0.000108174
g__SP3-e08	0.000109403	2.81E-07	0.000108128	0
g__Candidatus_Nitrosopelagicus	0	0	0	5.41E-05
g__ASF356	0	0	0	4.55E-05
g__Sporocytophaga	8.36E-05	1.68E-07	8.36E-05	0
g__Aeribacillus	8.24E-05	1.39E-07	7.60E-05	0
g__Coprobacillus	3.93E-05	2.46E-08	3.20E-05	0
g__Demequina	7.25E-05	9.36E-08	6.25E-05	0
g__Methanosaeta	0	0	0	3.56E-05
g__SUP05_cluster	0	0	0	7.01E-05
g__Nubsella	0	0	0	6.02E-05
g__Smithella	0	0	0	4.06E-05
g__JTB255_marine_benthic_group	0	0	0	3.69E-05
g__Succinivibrionaceae_UCG-001	0	0	0	4.18E-05
g__Spirosoma	4.06E-05	3.71E-08	3.93E-05	0
g__Defluviicoccus	3.07E-05	2.08E-08	2.95E-05	0
g__Pleionea	3.07E-05	1.75E-08	2.70E-05	0
g__[Bacteroides]_pectinophilus_group	0	0	0	3.81E-05
g__Parvibacter	0	0	0	3.56E-05
g__Bdellovibrio	1.23E-06	3.63E-11	1.23E-06	1.60E-05
g__Synechococcus_CC9902	0	0	0	1.23E-05

g__Prevotellaceae_UCG-004	0	0	0	1.23E-05
g__Flavitalea	0	0	0	1.23E-05
g__Harryflintia	0	0	0	1.23E-05
g__Sporolactobacillus	7.38E-06	1.31E-09	7.38E-06	2.83E-05
g__Planosporangium	1.23E-06	3.63E-11	1.23E-06	1.48E-05
g__Azospira	7.38E-06	7.00E-10	5.40E-06	2.70E-05
g__Virgisporangium	7.38E-06	1.31E-09	7.38E-06	2.70E-05
g__Cognatishimia	0	0	0	1.11E-05
g__Carnobacterium	0	0	0	1.11E-05
g__Bordetella	1.11E-05	2.94E-09	1.11E-05	0
g__Sulfuriferula	0	0	0	1.11E-05
g__Dolosigranulum	2.46E-06	1.45E-10	2.46E-06	1.72E-05
g__Photobacterium	0.001396428	4.04E-05	0.00129809	9.83E-06
g__Iamia	4.92E-06	5.80E-10	4.92E-06	2.09E-05
g__Rhodanobacter	2.46E-06	1.45E-10	2.46E-06	1.60E-05
g__Methanobrevibacter	3.69E-06	1.75E-10	2.70E-06	1.84E-05
g__Defluviimonas	0	0	0	9.83E-06
g__Paludibacter	0	0	0	9.83E-06
g__Succiniclasticum	0	0	0	9.83E-06
g__Promicromonospora	9.83E-06	2.32E-09	9.83E-06	0
g__Pelagibacterium	0	0	0	9.83E-06
g__unidentified_HOC36	0	0	0	9.83E-06
g__Candidatus_Paracaedibacter	9.83E-06	2.32E-09	9.83E-06	0
g__Micropruina	0	0	0	9.83E-06
g__Syntrophorhabdus	0	0	0	9.83E-06
g__Dactylosporangium	7.38E-06	7.00E-10	5.40E-06	2.46E-05
g__Gardnerella	0.000724028	7.75E-06	0.000568282	1.23E-05
g__[Eubacterium]_siraeum_group	4.92E-06	3.53E-10	3.84E-06	0.000542099
g__Chujaibacter	1.23E-06	3.63E-11	1.23E-06	1.23E-05
g__Thauera	1.23E-06	3.63E-11	1.23E-06	1.23E-05
g__Exiguobacterium	3.69E-06	9.93E-11	2.03E-06	8.24E-05
g__Oribacterium	0.000313459	1.20E-06	0.000223934	1.84E-05
g__Solibacillus	7.01E-05	5.07E-08	4.60E-05	1.23E-06
g__Alteromonas	0	0	0	8.60E-06
g__Rhodobacter	8.60E-06	1.78E-09	8.60E-06	0
g__EBM-39	8.60E-06	1.78E-09	8.60E-06	0
g__Novibacillus	8.60E-06	1.78E-09	8.60E-06	0
g__Jonquetella	8.60E-06	1.32E-09	7.42E-06	0
g__Antarcticibacterium	0	0	0	8.60E-06
g__Sharpea	0	0	0	8.60E-06
g__Lachnospiraceae_UCG-006	3.69E-06	1.75E-10	2.70E-06	0.000110633
g__NK4A214_group	9.10E-05	2.42E-08	3.18E-05	0.000363858
g__Eggerthella	0.001322673	7.65E-06	0.00056473	0.000259372
g__Roseimicrobium	4.92E-06	5.80E-10	4.92E-06	1.84E-05

g__CAG-352	6.15E-05	6.98E-08	5.39E-05	1.23E-06
g__Inquilinus	3.69E-06	3.26E-10	3.69E-06	1.60E-05
g__Eubacterium	1.11E-05	1.20E-09	7.06E-06	1.23E-06
g__Methylotenera	1.23E-06	3.63E-11	1.23E-06	1.11E-05
g__1959-1	1.35E-05	4.39E-09	1.35E-05	2.46E-06
g__Kroppenstedtia	2.46E-06	1.45E-10	2.46E-06	1.35E-05
g__Ruminiclostridium	7.38E-06	1.31E-09	7.38E-06	2.21E-05
g__Enterorhabdus	1.60E-05	2.95E-09	1.11E-05	0.00045851
g__Prevotellaceae_UCG-001	1.72E-05	1.89E-09	8.86E-06	0.000248309
g__Gordonibacter	3.69E-06	1.75E-10	2.70E-06	5.90E-05
g__Alkanindiges	2.34E-05	5.45E-09	1.51E-05	8.60E-06
g__Herbaspirillum	0.000169637	4.34E-07	0.000134532	7.38E-06
g__Paludicola	4.92E-06	5.80E-10	4.92E-06	1.72E-05
g__Erysipelotrichaceae_UCG-003	0.000735092	1.43E-06	0.000243694	0.000175783
g__Fusicatenibacter	0.001355863	8.09E-06	0.000580621	0.000168407
g__Marvinbryantia	0	0	0	7.38E-06
g__Idiomarina	0	0	0	7.38E-06
g__Lewinella	0	0	0	7.38E-06
g__Thermicanus	0	0	0	7.38E-06
g__Acidiphilium	7.38E-06	7.00E-10	5.40E-06	0
g__Xylanibacillus	0	0	0	7.38E-06
g__Alcaligenes	1.72E-05	4.08E-09	1.30E-05	7.74E-05
g__UTCFX1	7.38E-06	1.31E-09	7.38E-06	2.09E-05
g__SH3-11	3.69E-06	3.26E-10	3.69E-06	1.48E-05
g__Bacteroides	0.126503373	0.012597974	0.022911036	0.066748289
g__Agromyces	2.46E-06	6.94E-11	1.70E-06	1.23E-05
g__Lachnospiraceae_UCG-003	1.23E-06	3.63E-11	1.23E-06	9.83E-06
g__Frisingicoccus	1.23E-06	3.63E-11	1.23E-06	9.83E-06
g__Methanothermobacter	1.23E-06	3.63E-11	1.23E-06	9.83E-06
g__Anaeromyxobacter	6.15E-06	6.04E-10	5.02E-06	1.84E-05
g__Paraprevotella	4.92E-06	1.26E-10	2.29E-06	3.20E-05
g__Butyrivibrio	4.92E-06	5.80E-10	4.92E-06	7.13E-05
g__Clostridium_sensu_stricto_12	1.11E-05	2.33E-09	9.86E-06	7.74E-05
g__Acholeplasma	2.58E-05	5.62E-09	1.53E-05	1.11E-05
g__Cardiobacterium	2.21E-05	4.03E-09	1.30E-05	8.60E-06
g__Clostridium_sensu_stricto_7	8.60E-06	4.90E-10	4.52E-06	2.21E-05
g__Rikenellaceae_RC9_gut_group	9.22E-05	7.03E-08	5.41E-05	0.000351566
g__Anaeroplasma	1.23E-06	3.63E-11	1.23E-06	0.000224953
g__Faecalibacterium	0.040732436	0.0031585	0.011471886	0.015814305
g__Lachnospiraceae_NK4A136_group	0.001080511	3.16E-06	0.000362691	0.004801451
g__Hymenobacter;	2.46E-06	1.45E-10	2.46E-06	0.000758447

variance. group2.	standard. error. group2.	p.value	q.value	FC	log10FC
**GC.Tumor-CRC.Tumor-Genus**					
0	0	0.0000	0.0000	0.9725	-0.0121
0	0	0.0000	0.0000	0.9748	-0.0111
7.45E-09	1.50E-05	0.0000	0.0000	1.0277	0.0119
1.25E-08	1.94E-05	0.0000	0.0000	1.0277	0.0119
0	0	0.0000	0.0000	0.9772	-0.0100
5.41E-09	1.28E-05	0.0000	0.0000	1.0268	0.0115
3.41E-09	1.02E-05	0.0000	0.0000	1.0259	0.0111
1.65E-08	2.23E-05	0.0000	0.0000	1.0259	0.0111
0	0	0.0000	0.0001	0.9795	-0.0090
3.56E-09	1.04E-05	0.0000	0.0001	1.0273	0.0117
4.81E-09	1.21E-05	0.0000	0.0001	1.0241	0.0104
6.17E-09	1.37E-05	0.0000	0.0001	1.0241	0.0104
3.63E-09	1.05E-05	0.0000	0.0001	1.0232	0.0100
0	0	0.0000	0.0001	0.9807	-0.0085
1.06E-08	1.79E-05	0.0000	0.0001	1.0256	0.0110
1.65E-08	2.24E-05	0.0000	0.0001	1.0224	0.0096
3.37E-09	1.01E-05	0.0000	0.0001	1.0224	0.0096
1.29E-08	1.98E-05	0.0000	0.0001	1.0224	0.0096
3.22E-09	9.88E-06	0.0000	0.0002	1.0215	0.0092
5.30E-09	1.27E-05	0.0000	0.0003	1.0206	0.0088
4.54E-09	1.17E-05	0.0000	0.0003	1.0206	0.0088
1.02E-08	1.76E-05	0.0000	0.0005	1.0252	0.0108
4.22E-09	1.13E-05	0.0000	0.0005	1.0197	0.0085
1.28E-08	1.97E-05	0.0000	0.0005	1.0197	0.0085
1.16E-08	1.88E-05	0.0000	0.0005	1.0197	0.0085
1.06E-08	1.79E-05	0.0000	0.0005	1.0197	0.0085
2.64E-11	8.94E-07	0.0000	0.0005	0.9816	-0.0081
1.16E-08	1.88E-05	0.0000	0.0008	1.0188	0.0081
1.78E-08	2.32E-05	0.0000	0.0011	1.0225	0.0097
5.11E-11	1.24E-06	0.0000	0.0011	0.9813	-0.0082
1.05E-10	1.79E-06	0.0000	0.0011	0.9813	-0.0082
2.72E-09	9.07E-06	0.0000	0.0012	1.0179	0.0077
9.52E-09	1.70E-05	0.0000	0.0012	1.0179	0.0077
3.10E-09	9.69E-06	0.0000	0.0012	1.0179	0.0077
0	0	0.0000	0.0012	0.9855	-0.0064
3.02E-09	9.56E-06	0.0000	0.0016	1.0193	0.0083
2.64E-11	8.94E-07	0.0001	0.0018	0.9840	-0.0070
2.64E-11	8.94E-07	0.0001	0.0018	0.9840	-0.0070
4.33E-09	1.15E-05	0.0001	0.0025	1.0184	0.0079
1.16E-08	1.88E-05	0.0001	0.0025	1.0184	0.0079
4.07E-09	1.11E-05	0.0001	0.0031	1.0222	0.0095

2.29E-09	8.33E-06	0.0001	0.0036	1.0161	0.0069
1.05E-08	1.79E-05	0.0001	0.0041	1.0175	0.0075
2.59E-10	2.80E-06	0.0001	0.0046	0.9807	-0.0085
9.15E-09	1.66E-05	0.0002	0.0048	1.0213	0.0091
1.48E-09	6.69E-06	0.0002	0.0053	1.0143	0.0062
2.84E-09	9.27E-06	0.0002	0.0053	1.0143	0.0062
1.52E-08	2.15E-05	0.0002	0.0056	1.0190	0.0082
1.05E-10	1.79E-06	0.0002	0.0058	0.9837	-0.0072
9.52E-09	1.70E-05	0.0002	0.0060	1.0166	0.0072
2.64E-11	8.94E-07	0.0002	0.0066	0.9863	-0.0060
5.17E-09	1.25E-05	0.0003	0.0080	1.0134	0.0058
5.17E-09	1.25E-05	0.0003	0.0080	1.0134	0.0058
2.72E-09	9.09E-06	0.0003	0.0080	1.0134	0.0058
2.40E-09	8.53E-06	0.0003	0.0080	1.0134	0.0058
7.63E-10	4.81E-06	0.0004	0.0093	0.9795	-0.0090
0	0	0.0004	0.0099	0.9891	-0.0048
5.31E-10	4.01E-06	0.0004	0.0105	0.9798	-0.0089
1.96E-09	7.71E-06	0.0006	0.0125	1.0125	0.0054
2.34E-09	8.42E-06	0.0006	0.0125	1.0125	0.0054
5.17E-09	1.25E-05	0.0006	0.0125	1.0125	0.0054
3.86E-09	1.08E-05	0.0006	0.0125	1.0125	0.0054
2.29E-09	8.32E-06	0.0006	0.0125	1.0125	0.0054
1.36E-09	6.42E-06	0.0006	0.0125	1.0125	0.0054
2.53E-09	8.75E-06	0.0008	0.0137	1.0191	0.0082
1.05E-10	1.79E-06	0.0008	0.0137	0.9860	-0.0061
1.29E-10	1.97E-06	0.0009	0.0137	0.9857	-0.0062
0	0	0.0010	0.0137	0.9903	-0.0042
0	0	0.0010	0.0137	0.9903	-0.0042
0	0	0.0010	0.0137	0.9903	-0.0042
1.05E-08	1.79E-05	0.0010	0.0137	1.0163	0.0070
0.065975064	0.04471291	0.0010	0.0137	20.3530	1.3086
1.46E-05	0.000665712	0.0010	0.0137	0.0660	-1.1807
0.001242847	0.00613694	0.0010	0.0137	5.0991	0.7075
5.64E-06	0.000413311	0.0010	0.0137	0.1462	-0.8351
3.46E-08	3.24E-05	0.0010	0.0137	1.1050	0.0433
0	0	0.0010	0.0137	0.1783	-0.7488
5.04E-07	0.000123562	0.0010	0.0137	0.2333	-0.6320
1.77E-08	2.32E-05	0.0010	0.0137	1.0523	0.0222
9.90E-08	5.48E-05	0.0010	0.0137	0.2869	-0.5423
1.05E-08	1.78E-05	0.0010	0.0137	0.2864	-0.5431
2.23E-05	0.000822441	0.0010	0.0137	2.4679	0.3923
2.09E-05	0.000795108	0.0010	0.0137	2.7897	0.4456
0	0	0.0010	0.0137	0.5128	-0.2901
1.39E-05	0.000648695	0.0010	0.0137	2.1775	0.3380

1.44E-05	0.000661337	0.0010	0.0137	1.9902	0.2989
0	0	0.0010	0.0137	0.6294	-0.2011
1.83E-08	2.35E-05	0.0010	0.0137	1.0684	0.0287
0	0	0.0010	0.0137	0.8139	-0.0894
3.15E-06	0.000308993	0.0010	0.0137	1.6543	0.2186
0	0	0.0010	0.0137	0.7796	-0.1081
1.47E-08	2.11E-05	0.0010	0.0137	1.0420	0.0179
0	0	0.0010	0.0137	0.9024	-0.0446
6.59E-08	4.47E-05	0.0010	0.0137	1.0447	0.0190
0	0	0.0010	0.0137	0.9432	-0.0254
7.14E-08	4.65E-05	0.0010	0.0137	1.0787	0.0329
0	0	0.0010	0.0137	0.9367	-0.0284
0	0	0.0010	0.0137	0.9633	-0.0162
9.91E-09	1.73E-05	0.0010	0.0137	1.0456	0.0194
3.21E-09	9.86E-06	0.0011	0.0142	1.0116	0.0050
1.63E-09	7.03E-06	0.0011	0.0142	1.0116	0.0050
2.17E-09	8.11E-06	0.0011	0.0142	1.0116	0.0050
2.64E-11	8.94E-07	0.0012	0.0157	0.9887	-0.0049
1.40E-08	2.06E-05	0.0013	0.0167	1.0188	0.0081
5.11E-11	1.24E-06	0.0015	0.0199	0.9872	-0.0056
1.05E-09	5.64E-06	0.0016	0.0202	0.9815	-0.0081
1.29E-10	1.97E-06	0.0018	0.0228	0.9869	-0.0057
7.42E-11	1.50E-06	0.0018	0.0228	0.9869	-0.0057
9.50E-09	1.70E-05	0.0018	0.0228	1.0159	0.0069
6.46E-09	1.40E-05	0.0018	0.0228	1.0159	0.0069
3.45E-09	1.02E-05	0.0019	0.0228	1.0193	0.0083
3.80E-09	1.07E-05	0.0020	0.0228	1.0107	0.0046
3.80E-09	1.07E-05	0.0020	0.0228	1.0107	0.0046
3.80E-09	1.07E-05	0.0020	0.0228	1.0107	0.0046
3.20E-09	9.85E-06	0.0020	0.0228	1.0107	0.0046
2.17E-09	8.10E-06	0.0020	0.0228	1.0107	0.0046
1.65E-06	0.000223544	0.0020	0.0228	0.3147	-0.5021
1.31E-05	0.000629729	0.0020	0.0228	1.9963	0.3002
5.35E-06	0.000402813	0.0020	0.0228	1.8193	0.2599
3.83E-07	0.000107756	0.0020	0.0228	1.1654	0.0665
4.52E-09	1.17E-05	0.0021	0.0234	1.0131	0.0056
5.70E-10	4.16E-06	0.0022	0.0243	0.9812	-0.0082
0	0	0.0023	0.0255	0.9915	-0.0037
0	0	0.0023	0.0255	0.9915	-0.0037
0	0	0.0023	0.0255	0.9915	-0.0037
0	0	0.0023	0.0255	0.9915	-0.0037
1.64E-09	7.04E-06	0.0027	0.0290	1.0136	0.0059
3.60E-09	1.04E-05	0.0027	0.0290	1.0136	0.0059
1.05E-10	1.79E-06	0.0030	0.0302	0.9884	-0.0051

7.93E-07	0.000155007	0.0030	0.0302	0.1531	-0.8151
1.36E-05	0.000642064	0.0030	0.0302	1.8050	0.2565
6.59E-08	4.47E-05	0.0030	0.0302	0.4030	-0.3947
7.14E-06	0.000465307	0.0030	0.0302	1.4890	0.1729
4.79E-07	0.00012053	0.0030	0.0302	1.2918	0.1112
9.38E-07	0.000168569	0.0030	0.0302	1.4134	0.1503
3.73E-08	3.36E-05	0.0030	0.0302	1.1026	0.0424
7.87E-09	1.54E-05	0.0030	0.0302	1.0184	0.0079
1.40E-08	2.06E-05	0.0032	0.0319	1.0156	0.0067
1.06E-09	5.67E-06	0.0035	0.0343	0.9824	-0.0077
2.16E-09	8.09E-06	0.0036	0.0343	1.0098	0.0042
1.12E-09	5.84E-06	0.0036	0.0343	1.0098	0.0042
9.61E-10	5.40E-06	0.0036	0.0343	1.0098	0.0042
3.19E-09	9.83E-06	0.0036	0.0343	1.0098	0.0042
2.21E-09	8.19E-06	0.0036	0.0343	1.0098	0.0042
1.56E-09	6.87E-06	0.0036	0.0343	1.0098	0.0042
1.18E-09	5.98E-06	0.0036	0.0343	1.0098	0.0042
6.40E-10	4.40E-06	0.0037	0.0343	0.9845	-0.0068
0.000449902	0.003692344	0.0040	0.0356	0.1098	-0.9595
5.25E-05	0.001261488	0.0040	0.0356	0.0738	-1.1322
1.24E-06	0.000194035	0.0040	0.0356	0.1871	-0.7280
7.95E-08	4.91E-05	0.0040	0.0356	0.3139	-0.5032
2.50E-09	8.70E-06	0.0040	0.0356	0.7603	-0.1190
1.80E-07	7.38E-05	0.0040	0.0356	1.1550	0.0626
1.37E-07	6.44E-05	0.0040	0.0356	1.1757	0.0703
0	0	0.0056	0.0484	0.9927	-0.0032
0	0	0.0056	0.0484	0.9927	-0.0032
3.22E-08	3.13E-05	0.0060	0.0514	0.7508	-0.1245
1.19E-08	1.90E-05	0.0060	0.0514	1.0475	0.0201
2.59E-10	2.80E-06	0.0061	0.0522	0.9857	-0.0063
1.25E-09	6.15E-06	0.0064	0.0540	1.0104	0.0045
1.74E-09	7.27E-06	0.0065	0.0550	1.0113	0.0049
8.42E-10	5.05E-06	0.0068	0.0561	1.0089	0.0039
2.64E-09	8.94E-06	0.0068	0.0561	1.0089	0.0039
2.15E-09	8.07E-06	0.0068	0.0561	1.0089	0.0039
1.71E-09	7.20E-06	0.0068	0.0561	1.0089	0.0039
4.93E-06	0.000386492	0.0070	0.0566	0.4063	-0.3912
3.39E-09	1.01E-05	0.0070	0.0566	0.6088	-0.2156
1.97E-07	7.73E-05	0.0070	0.0566	1.1547	0.0625
5.17E-09	1.25E-05	0.0074	0.0594	1.0118	0.0051
3.69E-09	1.06E-05	0.0078	0.0623	1.0138	0.0059
0.000589427	0.004226277	0.0080	0.0626	0.1041	-0.9825
2.85E-06	0.000293844	0.0080	0.0626	1.3138	0.1185
6.04E-09	1.35E-05	0.0080	0.0626	1.0132	0.0057

6.26E-09	1.38E-05	0.0080	0.0626	1.0132	0.0057
7.42E-09	1.50E-05	0.0082	0.0640	1.0168	0.0072
4.90E-08	3.85E-05	0.0090	0.0692	1.0573	0.0242
9.14E-09	1.66E-05	0.0090	0.0692	1.0288	0.0123
1.51E-07	6.77E-05	0.0100	0.0755	0.3380	-0.4711
5.32E-09	1.27E-05	0.0100	0.0755	0.8171	-0.0877
8.27E-08	5.01E-05	0.0100	0.0755	1.0572	0.0242
2.04E-10	2.49E-06	0.0100	0.0755	0.9890	-0.0048
9.68E-09	1.71E-05	0.0103	0.0771	1.0149	0.0064
5.34E-10	4.02E-06	0.0108	0.0798	1.0095	0.0041
8.60E-10	5.11E-06	0.0108	0.0798	1.0095	0.0041
2.00E-09	7.78E-06	0.0110	0.0807	0.8703	-0.0603
1.12E-07	5.83E-05	0.0110	0.0807	1.0894	0.0372
2.64E-11	8.94E-07	0.0119	0.0866	0.9924	-0.0033
0.002321473	0.008387352	0.0120	0.0871	10.1175	1.0051
1.64E-09	7.04E-06	0.0122	0.0882	1.0109	0.0047
8.31E-10	5.02E-06	0.0129	0.0906	1.0080	0.0035
7.22E-10	4.68E-06	0.0129	0.0906	1.0080	0.0035
1.15E-07	5.91E-05	0.0130	0.0906	1.1421	0.0577
1.02E-08	1.75E-05	0.0130	0.0906	1.0331	0.0141
0	0	0.0132	0.0906	0.9939	-0.0027
0	0	0.0132	0.0906	0.9939	-0.0027
0	0	0.0132	0.0906	0.9939	-0.0027
0	0	0.0132	0.0906	0.9939	-0.0027
0	0	0.0132	0.0906	0.9939	-0.0027
0	0	0.0132	0.0906	0.9939	-0.0027
0	0	0.0132	0.0906	0.9939	-0.0027
1.86E-06	0.000237718	0.0140	0.0953	1.4168	0.1513
0.00120763	0.006049367	0.0160	0.1084	6.7054	0.8264
1.83E-08	2.36E-05	0.0170	0.1146	0.8381	-0.0767
2.04E-10	2.49E-06	0.0178	0.1198	0.9902	-0.0043
2.65E-09	8.96E-06	0.0182	0.1217	1.0086	0.0037
4.95E-09	1.22E-05	0.0187	0.1247	1.0120	0.0052
3.01E-09	9.55E-06	0.0190	0.1251	0.6910	-0.1605
6.53E-09	1.41E-05	0.0190	0.1251	1.0340	0.0145
9.81E-10	5.45E-06	0.0201	0.1311	1.0100	0.0043
7.40E-09	1.50E-05	0.0201	0.1311	1.0136	0.0059
6.75E-09	1.43E-05	0.0202	0.1311	1.0106	0.0046
1.29E-10	1.97E-06	0.0205	0.1325	0.9905	-0.0041
1.59E-06	0.00021953	0.0210	0.1345	0.4223	-0.3744
1.39E-09	6.48E-06	0.0211	0.1345	0.9871	-0.0056
1.11E-09	5.81E-06	0.0211	0.1345	0.9871	-0.0056
1.05E-10	1.79E-06	0.0217	0.1380	0.9920	-0.0035
1.13E-08	1.85E-05	0.0220	0.1385	0.4978	-0.3029

1.00E-08	1.74E-05	0.0220	0.1385	1.0409	0.0174
6.40E-10	4.40E-06	0.0241	0.1400	1.0063	0.0027
1.29E-09	6.26E-06	0.0241	0.1400	1.0063	0.0027
1.29E-09	6.26E-06	0.0241	0.1400	1.0063	0.0027
6.40E-10	4.40E-06	0.0241	0.1400	1.0063	0.0027
3.68E-10	3.34E-06	0.0241	0.1400	1.0063	0.0027
7.63E-10	4.81E-06	0.0245	0.1400	1.0072	0.0031
4.37E-10	3.64E-06	0.0245	0.1400	1.0072	0.0031
6.54E-10	4.45E-06	0.0245	0.1400	1.0072	0.0031
1.69E-09	7.15E-06	0.0245	0.1400	1.0072	0.0031
1.69E-09	7.15E-06	0.0245	0.1400	1.0072	0.0031
7.63E-10	4.81E-06	0.0245	0.1400	1.0072	0.0031
9.81E-10	5.45E-06	0.0245	0.1400	1.0072	0.0031
1.04E-09	5.60E-06	0.0245	0.1400	1.0072	0.0031
5.46E-10	4.07E-06	0.0245	0.1400	1.0072	0.0031
9.81E-10	5.45E-06	0.0245	0.1400	1.0072	0.0031
7.09E-10	4.63E-06	0.0245	0.1400	1.0072	0.0031
2.74E-10	2.88E-06	0.0245	0.1400	1.0072	0.0031
8.18E-10	4.98E-06	0.0245	0.1400	1.0072	0.0031
5.46E-10	4.07E-06	0.0245	0.1400	1.0072	0.0031
2.79E-10	2.91E-06	0.0247	0.1401	0.9899	-0.0044
3.33E-10	3.18E-06	0.0247	0.1401	0.9899	-0.0044
5.00E-08	3.89E-05	0.0250	0.1406	1.0519	0.0220
3.02E-09	9.56E-06	0.0250	0.1406	0.8348	-0.0784
4.97E-07	0.00012276	0.0260	0.1445	0.2825	-0.5490
7.82E-07	0.000153953	0.0260	0.1445	1.3303	0.1239
2.34E-09	8.41E-06	0.0260	0.1445	1.0117	0.0050
5.59E-10	4.12E-06	0.0268	0.1485	0.9874	-0.0055
4.41E-07	0.000115645	0.0270	0.1489	1.1420	0.0577
2.47E-05	0.000864821	0.0280	0.1532	2.5885	0.4130
2.07E-08	2.51E-05	0.0280	0.1532	1.0390	0.0166
5.28E-08	4.00E-05	0.0290	0.1563	1.0536	0.0227
1.44E-06	0.000209117	0.0290	0.1563	1.3272	0.1229
2.17E-08	2.57E-05	0.0290	0.1563	1.0393	0.0168
2.76E-08	2.89E-05	0.0290	0.1563	1.0501	0.0212
1.57E-09	6.89E-06	0.0300	0.1592	0.9865	-0.0059
2.45E-08	2.72E-05	0.0300	0.1592	0.7824	-0.1066
5.62E-08	4.13E-05	0.0300	0.1592	0.7814	-0.1071
1.77E-06	0.000231782	0.0300	0.1592	1.2923	0.1114
8.42E-10	5.05E-06	0.0309	0.1593	1.0077	0.0033
8.97E-10	5.21E-06	0.0309	0.1593	1.0077	0.0033
2.24E-10	2.61E-06	0.0310	0.1593	0.9485	-0.0230
4.22E-10	3.58E-06	0.0313	0.1593	0.9914	-0.0038
0	0	0.0314	0.1593	0.9951	-0.0021

0	0	0.0314	0.1593	0.9951	-0.0021
0	0	0.0314	0.1593	0.9951	-0.0021
0	0	0.0314	0.1593	0.9951	-0.0021
0	0	0.0314	0.1593	0.9951	-0.0021
0	0	0.0314	0.1593	0.9951	-0.0021
0	0	0.0314	0.1593	0.9951	-0.0021
0	0	0.0314	0.1593	0.9951	-0.0021
0	0	0.0314	0.1593	0.9951	-0.0021
9.90E-08	5.48E-05	0.0330	0.1659	1.0867	0.0361
6.40E-08	4.40E-05	0.0330	0.1659	1.0978	0.0405
7.58E-10	4.79E-06	0.0331	0.1661	1.0091	0.0040
1.57E-06	0.00021787	0.0350	0.1741	1.3266	0.1227
2.46E-08	2.73E-05	0.0350	0.1741	1.0443	0.0188
4.39E-06	0.000364601	0.0370	0.1834	1.5110	0.1793
4.05E-07	0.000110742	0.0380	0.1864	1.1783	0.0713
9.58E-09	1.70E-05	0.0380	0.1864	0.7374	-0.1323
1.16E-07	5.94E-05	0.0380	0.1864	1.1172	0.0481
1.45E-08	2.09E-05	0.0390	0.1900	1.0386	0.0164
1.42E-08	2.07E-05	0.0390	0.1900	1.0396	0.0169
8.59E-05	0.001613477	0.0400	0.1930	2.6412	0.4218
2.42E-10	2.71E-06	0.0432	0.1930	1.0054	0.0023
5.14E-10	3.95E-06	0.0432	0.1930	1.0054	0.0023
9.49E-10	5.36E-06	0.0432	0.1930	1.0054	0.0023
5.14E-10	3.95E-06	0.0432	0.1930	1.0054	0.0023
9.49E-10	5.36E-06	0.0432	0.1930	1.0054	0.0023
9.49E-10	5.36E-06	0.0432	0.1930	1.0054	0.0023
9.49E-10	5.36E-06	0.0432	0.1930	1.0054	0.0023
2.42E-10	2.71E-06	0.0432	0.1930	1.0054	0.0023
2.97E-10	3.00E-06	0.0432	0.1930	1.0054	0.0023
9.49E-10	5.36E-06	0.0432	0.1930	1.0054	0.0023
9.49E-10	5.36E-06	0.0432	0.1930	1.0054	0.0023
9.49E-10	5.36E-06	0.0432	0.1930	1.0054	0.0023
9.49E-10	5.36E-06	0.0432	0.1930	1.0054	0.0023
9.49E-10	5.36E-06	0.0432	0.1930	1.0054	0.0023
9.49E-10	5.36E-06	0.0432	0.1930	1.0054	0.0023
9.49E-10	5.36E-06	0.0432	0.1930	1.0054	0.0023
9.49E-10	5.36E-06	0.0432	0.1930	1.0054	0.0023
6.78E-10	4.53E-06	0.0432	0.1930	1.0054	0.0023
5.14E-10	3.95E-06	0.0432	0.1930	1.0054	0.0023
3.51E-10	3.26E-06	0.0432	0.1930	1.0054	0.0023
2.97E-10	3.00E-06	0.0432	0.1930	1.0054	0.0023
4.60E-10	3.73E-06	0.0432	0.1930	1.0054	0.0023
4.60E-10	3.73E-06	0.0432	0.1930	1.0054	0.0023
2.42E-10	2.71E-06	0.0432	0.1930	1.0054	0.0023

1.69E-09	7.16E-06	0.0443	0.1959	1.0102	0.0044
1.53E-09	6.81E-06	0.0443	0.1959	1.0102	0.0044
1.96E-09	7.71E-06	0.0443	0.1959	0.9867	-0.0058
1.74E-09	7.26E-06	0.0450	0.1982	0.9389	-0.0274
6.81E-08	4.54E-05	0.0470	0.2057	0.5992	-0.2224
1.15E-06	0.000186494	0.0470	0.2057	1.2021	0.0799
8.72E-10	5.14E-06	0.0488	0.2125	1.0088	0.0038
4.74E-05	0.001198083	0.0490	0.2125	2.2090	0.3442
6.37E-08	4.39E-05	0.0490	0.2125	0.7131	-0.1468
1.84E-09	7.47E-06	0.0493	0.2135	1.0113	0.0049
**GC.Para-CRC.Para-Genus**					
2.96E-08	2.87E-05	0.0000	0.0000	1.0287	0.0123
1.30E-08	1.90E-05	0.0000	0.0000	1.0279	0.0119
3.68E-09	1.01E-05	0.0000	0.0000	1.0279	0.0119
1.21E-08	1.83E-05	0.0000	0.0000	1.0270	0.0116
9.67E-11	1.64E-06	0.0000	0.0000	0.9741	-0.0114
2.48E-08	2.62E-05	0.0000	0.0000	1.0262	0.0112
5.91E-09	1.28E-05	0.0000	0.0000	1.0262	0.0112
2.42E-11	8.20E-07	0.0000	0.0000	0.9768	-0.0102
8.83E-09	1.57E-05	0.0000	0.0000	1.0274	0.0117
8.53E-09	1.54E-05	0.0000	0.0000	1.0246	0.0105
0	0	0.0000	0.0000	0.9807	-0.0085
7.22E-09	1.42E-05	0.0000	0.0001	1.0229	0.0099
1.90E-08	2.29E-05	0.0000	0.0001	1.0229	0.0099
1.90E-08	2.29E-05	0.0000	0.0001	1.0229	0.0099
1.35E-08	1.93E-05	0.0000	0.0001	1.0238	0.0102
6.46E-09	1.34E-05	0.0000	0.0001	1.0238	0.0102
8.20E-09	1.51E-05	0.0000	0.0001	1.0238	0.0102
2.18E-10	2.46E-06	0.0000	0.0001	0.9761	-0.0105
9.07E-09	1.59E-05	0.0000	0.0001	1.0258	0.0111
1.08E-08	1.73E-05	0.0000	0.0001	1.0221	0.0095
8.22E-09	1.51E-05	0.0000	0.0001	1.0221	0.0095
8.72E-09	1.56E-05	0.0000	0.0001	1.0221	0.0095
5.89E-09	1.28E-05	0.0000	0.0001	1.0221	0.0095
5.23E-09	1.21E-05	0.0000	0.0001	1.0241	0.0104
1.40E-08	1.97E-05	0.0000	0.0001	1.0213	0.0092
5.05E-09	1.18E-05	0.0000	0.0001	1.0213	0.0092
9.67E-11	1.64E-06	0.0000	0.0001	0.9788	-0.0093
0	0	0.0000	0.0001	0.9831	-0.0074
1.01E-08	1.68E-05	0.0000	0.0001	1.0233	0.0100
1.52E-08	2.06E-05	0.0000	0.0001	1.0233	0.0100
2.56E-09	8.43E-06	0.0000	0.0001	1.0233	0.0100
3.06E-09	9.22E-06	0.0000	0.0001	1.0233	0.0100
1.51E-08	2.05E-05	0.0000	0.0001	1.0205	0.0088

7.45E-09	1.44E-05	0.0000	0.0001	1.0205	0.0088
3.47E-09	9.82E-06	0.0000	0.0001	1.0205	0.0088
7.53E-09	1.45E-05	0.0000	0.0002	1.0245	0.0105
5.39E-09	1.22E-05	0.0000	0.0002	1.0245	0.0105
0	0	0.0000	0.0002	0.9843	-0.0069
9.80E-09	1.65E-05	0.0000	0.0002	1.0197	0.0085
1.28E-08	1.88E-05	0.0000	0.0002	1.0197	0.0085
1.74E-09	6.95E-06	0.0000	0.0002	1.0197	0.0085
1.39E-08	1.97E-05	0.0000	0.0002	1.0197	0.0085
7.21E-09	1.42E-05	0.0000	0.0002	1.0197	0.0085
3.43E-09	9.76E-06	0.0000	0.0002	1.0197	0.0085
2.69E-09	8.64E-06	0.0000	0.0002	1.0197	0.0085
3.28E-09	9.55E-06	0.0000	0.0002	1.0197	0.0085
1.17E-08	1.80E-05	0.0000	0.0003	1.0188	0.0081
2.07E-10	2.40E-06	0.0000	0.0004	0.9777	-0.0098
6.48E-09	1.34E-05	0.0000	0.0005	1.0209	0.0090
3.70E-09	1.01E-05	0.0000	0.0005	1.0209	0.0090
3.69E-09	1.01E-05	0.0000	0.0006	1.0180	0.0078
1.32E-08	1.92E-05	0.0000	0.0007	1.0241	0.0103
1.76E-09	7.00E-06	0.0000	0.0009	1.0164	0.0071
8.73E-09	1.56E-05	0.0000	0.0009	1.0164	0.0071
4.45E-09	1.11E-05	0.0000	0.0009	1.0164	0.0071
3.16E-09	9.36E-06	0.0000	0.0009	1.0164	0.0071
2.70E-09	8.67E-06	0.0000	0.0009	1.0172	0.0074
1.81E-09	7.09E-06	0.0000	0.0009	1.0172	0.0074
8.73E-09	1.56E-05	0.0001	0.0015	1.0156	0.0067
7.83E-09	1.48E-05	0.0001	0.0015	1.0156	0.0067
5.25E-09	1.21E-05	0.0001	0.0015	1.0156	0.0067
2.21E-09	7.84E-06	0.0001	0.0015	1.0156	0.0067
5.11E-09	1.19E-05	0.0001	0.0016	1.0225	0.0096
3.39E-09	9.70E-06	0.0001	0.0019	1.0176	0.0076
1.82E-09	7.10E-06	0.0001	0.0023	1.0148	0.0064
1.87E-09	7.20E-06	0.0001	0.0023	1.0148	0.0064
3.26E-09	9.51E-06	0.0001	0.0023	1.0148	0.0064
2.26E-09	7.93E-06	0.0001	0.0023	1.0148	0.0064
1.72E-09	6.90E-06	0.0001	0.0023	1.0148	0.0064
1.18E-10	1.81E-06	0.0002	0.0029	0.9831	-0.0074
6.99E-09	1.39E-05	0.0002	0.0038	1.0139	0.0060
5.50E-09	1.24E-05	0.0002	0.0038	1.0139	0.0060
4.00E-09	1.05E-05	0.0002	0.0038	1.0139	0.0060
1.76E-09	7.00E-06	0.0002	0.0038	1.0139	0.0060
1.72E-09	6.90E-06	0.0002	0.0038	1.0139	0.0060
1.41E-09	6.26E-06	0.0003	0.0046	1.0160	0.0069
2.75E-09	8.75E-06	0.0003	0.0046	1.0160	0.0069

2.85E-09	8.90E-06	0.0003	0.0046	1.0160	0.0069
2.42E-11	8.20E-07	0.0003	0.0052	0.9875	-0.0055
2.96E-09	9.06E-06	0.0004	0.0063	1.0131	0.0057
2.31E-09	8.01E-06	0.0004	0.0063	1.0131	0.0057
4.15E-09	1.07E-05	0.0004	0.0063	1.0131	0.0057
2.16E-09	7.75E-06	0.0004	0.0063	1.0131	0.0057
1.86E-09	7.19E-06	0.0004	0.0063	1.0131	0.0057
4.92E-09	1.17E-05	0.0004	0.0064	1.0172	0.0074
1.64E-08	2.13E-05	0.0006	0.0095	1.0200	0.0086
3.04E-09	9.19E-06	0.0007	0.0095	1.0163	0.0070
1.86E-09	7.19E-06	0.0007	0.0095	1.0123	0.0053
1.81E-09	7.09E-06	0.0007	0.0095	1.0123	0.0053
0.12569853	0.059089981	0.0010	0.0095	51.8689	1.7149
8.55E-07	0.000154094	0.0010	0.0095	0.1017	-0.9925
0.000163325	0.00212998	0.0010	0.0095	0.0582	-1.2353
0.000198344	0.002347246	0.0010	0.0095	0.0487	-1.3128
2.21E-05	0.000783505	0.0010	0.0095	0.0701	-1.1545
3.16E-06	0.000296482	0.0010	0.0095	0.2805	-0.5520
1.37E-09	6.16E-06	0.0010	0.0095	0.5853	-0.2326
8.96E-09	1.58E-05	0.0010	0.0095	0.4742	-0.3241
1.14E-06	0.000177779	0.0010	0.0095	0.1199	-0.9212
0	0	0.0010	0.0095	0.4701	-0.3278
1.62E-06	0.000212177	0.0010	0.0095	0.0985	-1.0067
1.23E-08	1.85E-05	0.0010	0.0095	0.4016	-0.3962
9.67E-11	1.64E-06	0.0010	0.0095	0.6138	-0.2120
4.80E-07	0.000115424	0.0010	0.0095	0.2330	-0.6326
1.39E-07	6.22E-05	0.0010	0.0095	0.2278	-0.6424
4.10E-07	0.000106685	0.0010	0.0095	0.2939	-0.5318
2.72E-07	8.69E-05	0.0010	0.0095	1.0869	0.0362
3.48E-07	9.83E-05	0.0010	0.0095	1.2372	0.0924
9.82E-09	1.65E-05	0.0010	0.0095	0.4503	-0.3465
1.35E-06	0.000193742	0.0010	0.0095	1.4040	0.1474
4.17E-08	3.40E-05	0.0010	0.0095	0.4820	-0.3169
5.35E-09	1.22E-05	0.0010	0.0095	0.7039	-0.1525
7.69E-08	4.62E-05	0.0010	0.0095	1.0836	0.0349
6.40E-08	4.22E-05	0.0010	0.0095	1.0860	0.0358
1.01E-07	5.30E-05	0.0010	0.0095	1.1520	0.0615
2.69E-08	2.73E-05	0.0010	0.0095	1.0541	0.0229
1.46E-08	2.02E-05	0.0010	0.0095	1.0626	0.0264
6.18E-07	0.000130977	0.0010	0.0095	1.2188	0.0859
0	0	0.0010	0.0095	0.7501	-0.1249
0	0	0.0010	0.0095	0.8470	-0.0721
0	0	0.0010	0.0095	0.8954	-0.0480
0	0	0.0010	0.0095	0.9014	-0.0451

1.63E-07	6.72E-05	0.0010	0.0095	1.0672	0.0282
1.41E-07	6.26E-05	0.0010	0.0095	1.0951	0.0394
1.26E-07	5.93E-05	0.0010	0.0095	1.0770	0.0322
0	0	0.0010	0.0095	0.9166	-0.0378
0	0	0.0010	0.0095	0.9622	-0.0168
3.31E-08	3.03E-05	0.0010	0.0095	1.0303	0.0130
0	0	0.0010	0.0095	0.9313	-0.0309
6.65E-08	4.30E-05	0.0010	0.0095	1.0746	0.0312
0	0	0.0010	0.0095	0.9656	-0.0152
0	0	0.0010	0.0095	0.9498	-0.0224
6.84E-08	4.36E-05	0.0010	0.0095	1.1188	0.0488
3.61E-08	3.17E-05	0.0010	0.0095	1.0721	0.0302
0	0	0.0010	0.0095	0.9610	-0.0173
0	0	0.0010	0.0095	0.9702	-0.0131
0	0	0.0010	0.0095	0.9702	-0.0131
1.56E-08	2.08E-05	0.0010	0.0095	1.0533	0.0225
1.17E-08	1.80E-05	0.0010	0.0095	1.0402	0.0171
1.40E-08	1.97E-05	0.0010	0.0095	1.0459	0.0195
2.85E-09	8.90E-06	0.0012	0.0113	1.0155	0.0067
3.55E-09	9.92E-06	0.0014	0.0130	1.0115	0.0050
1.41E-09	6.25E-06	0.0014	0.0130	1.0115	0.0050
2.51E-09	8.35E-06	0.0015	0.0135	1.0135	0.0058
7.83E-09	1.48E-05	0.0015	0.0135	1.0135	0.0058
4.79E-09	1.15E-05	0.0015	0.0138	1.0171	0.0074
0	0	0.0016	0.0149	0.9915	-0.0037
2.25E-06	0.000249734	0.0020	0.0172	1.5169	0.1810
2.42E-11	8.20E-07	0.0020	0.0172	0.7500	-0.1249
2.25E-08	2.50E-05	0.0020	0.0172	1.0536	0.0227
2.26E-07	7.92E-05	0.0020	0.0172	1.0975	0.0404
1.21E-07	5.79E-05	0.0020	0.0172	1.1486	0.0602
9.01E-08	5.00E-05	0.0020	0.0172	1.1420	0.0577
3.88E-08	3.28E-05	0.0020	0.0172	1.0713	0.0299
1.64E-09	6.75E-06	0.0025	0.0202	1.0098	0.0042
1.14E-09	5.64E-06	0.0025	0.0202	1.0098	0.0042
1.74E-09	6.95E-06	0.0025	0.0202	1.0098	0.0042
2.49E-09	8.31E-06	0.0025	0.0202	1.0098	0.0042
2.49E-09	8.31E-06	0.0025	0.0202	1.0098	0.0042
1.74E-09	6.95E-06	0.0025	0.0202	1.0098	0.0042
1.34E-09	6.11E-06	0.0025	0.0202	1.0098	0.0042
1.09E-09	5.51E-06	0.0025	0.0202	1.0098	0.0042
6.47E-10	4.24E-06	0.0025	0.0202	1.0098	0.0042
1.51E-09	6.49E-06	0.0025	0.0202	1.0119	0.0051
4.80E-09	1.15E-05	0.0025	0.0202	1.0119	0.0051
4.15E-09	1.07E-05	0.0025	0.0202	1.0119	0.0051

1.27E-09	5.93E-06	0.0026	0.0202	1.0127	0.0055
5.50E-09	1.24E-05	0.0026	0.0202	1.0127	0.0055
1.15E-09	5.66E-06	0.0026	0.0202	1.0107	0.0046
1.10E-09	5.53E-06	0.0026	0.0202	1.0107	0.0046
2.49E-09	8.32E-06	0.0026	0.0202	1.0107	0.0046
1.25E-09	5.89E-06	0.0026	0.0202	1.0107	0.0046
2.92E-08	2.85E-05	0.0030	0.0220	0.4620	-0.3353
1.52E-07	6.50E-05	0.0030	0.0220	1.1147	0.0472
3.56E-07	9.95E-05	0.0030	0.0220	1.1672	0.0671
2.70E-08	2.74E-05	0.0030	0.0220	1.0615	0.0259
4.25E-08	3.44E-05	0.0030	0.0220	1.1068	0.0441
6.49E-09	1.34E-05	0.0030	0.0220	1.0303	0.0130
1.48E-08	2.03E-05	0.0030	0.0220	1.0591	0.0249
1.07E-08	1.72E-05	0.0030	0.0220	1.0410	0.0174
2.21E-09	7.84E-06	0.0032	0.0234	1.0131	0.0056
1.42E-08	1.98E-05	0.0037	0.0272	1.0167	0.0072
2.42E-11	8.20E-07	0.0038	0.0275	0.9911	-0.0039
0.000390951	0.003295413	0.0040	0.0283	0.1688	-0.7727
1.72E-08	2.19E-05	0.0040	0.0283	1.0595	0.0251
8.11E-07	0.00015005	0.0040	0.0283	0.4128	-0.3843
1.18E-10	1.81E-06	0.0040	0.0283	0.4509	-0.3459
2.03E-09	7.51E-06	0.0044	0.0299	1.0090	0.0039
2.93E-09	9.01E-06	0.0044	0.0299	1.0090	0.0039
2.93E-09	9.01E-06	0.0044	0.0299	1.0090	0.0039
2.03E-09	7.51E-06	0.0044	0.0299	1.0090	0.0039
1.63E-09	6.73E-06	0.0044	0.0299	1.0090	0.0039
9.36E-10	5.10E-06	0.0044	0.0299	1.0090	0.0039
3.46E-09	9.80E-06	0.0045	0.0306	1.0187	0.0080
2.18E-10	2.46E-06	0.0047	0.0318	0.9891	-0.0048
2.38E-06	0.000256936	0.0050	0.0334	0.2590	-0.5866
6.83E-08	4.36E-05	0.0050	0.0334	0.5120	-0.2907
1.92E-08	2.31E-05	0.0050	0.0334	1.0512	0.0217
6.41E-09	1.33E-05	0.0053	0.0351	1.0163	0.0070
3.75E-09	1.02E-05	0.0058	0.0385	1.0159	0.0068
1.33E-07	6.07E-05	0.0060	0.0388	0.6213	-0.2067
6.22E-07	0.000131497	0.0060	0.0388	1.2762	0.1059
1.23E-08	1.85E-05	0.0060	0.0388	1.0482	0.0205
5.36E-08	3.86E-05	0.0060	0.0388	1.1019	0.0422
2.85E-08	2.81E-05	0.0060	0.0388	1.0680	0.0286
1.78E-08	2.23E-05	0.0065	0.0421	1.0170	0.0073
1.20E-09	5.77E-06	0.0067	0.0429	0.9860	-0.0061
5.60E-07	0.000124754	0.0070	0.0443	1.2364	0.0922
7.82E-06	0.000466022	0.0070	0.0443	2.2504	0.3523
3.62E-08	3.17E-05	0.0070	0.0443	1.0773	0.0324

2.42E-09	8.20E-06	0.0077	0.0470	1.0082	0.0035
1.97E-09	7.40E-06	0.0077	0.0470	1.0082	0.0035
1.62E-09	6.71E-06	0.0077	0.0470	1.0082	0.0035
7.76E-10	4.64E-06	0.0077	0.0470	1.0082	0.0035
1.27E-09	5.95E-06	0.0077	0.0470	1.0082	0.0035
1.07E-09	5.46E-06	0.0077	0.0470	1.0082	0.0035
1.22E-09	5.83E-06	0.0077	0.0470	1.0082	0.0035
9.75E-10	5.21E-06	0.0077	0.0470	1.0082	0.0035
2.19E-05	0.000780064	0.0080	0.0476	0.1990	-0.7012
1.34E-08	1.93E-05	0.0080	0.0476	0.7197	-0.1429
1.12E-06	0.000176439	0.0080	0.0476	0.3813	-0.4187
7.88E-07	0.000147979	0.0080	0.0476	1.2434	0.0946
2.15E-08	2.45E-05	0.0080	0.0476	1.0713	0.0299
2.21E-09	7.84E-06	0.0085	0.0504	1.0114	0.0049
9.10E-06	0.000502821	0.0090	0.0527	0.2963	-0.5282
3.32E-07	9.60E-05	0.0090	0.0527	1.2543	0.0984
9.67E-11	1.64E-06	0.0096	0.0557	0.9907	-0.0041
8.86E-06	0.000496154	0.0100	0.0574	1.9460	0.2892
7.28E-08	4.50E-05	0.0100	0.0574	0.2920	-0.5347
1.29E-07	5.98E-05	0.0100	0.0574	1.1229	0.0504
2.86E-08	2.82E-05	0.0100	0.0574	1.0418	0.0178
0	0	0.0102	0.0579	0.9939	-0.0027
0	0	0.0102	0.0579	0.9939	-0.0027
0	0	0.0102	0.0579	0.9939	-0.0027
0	0	0.0102	0.0579	0.9939	-0.0027
0.004163649	0.010754391	0.0110	0.0614	0.2881	-0.5405
2.20E-08	2.47E-05	0.0110	0.0614	0.6255	-0.2037
3.56E-06	0.000314627	0.0120	0.0654	0.2965	-0.5280
1.19E-06	0.000181557	0.0120	0.0654	0.3198	-0.4951
1.27E-06	0.000187745	0.0120	0.0654	1.4710	0.1676
1.88E-06	0.00022849	0.0120	0.0654	1.3927	0.1438
5.20E-06	0.000380206	0.0120	0.0654	1.9502	0.2901
2.04E-06	0.00023822	0.0120	0.0654	1.5897	0.2013
5.91E-07	0.000128141	0.0130	0.0695	0.5325	-0.2737
4.77E-06	0.000364194	0.0130	0.0695	1.7467	0.2422
2.12E-07	7.68E-05	0.0130	0.0695	1.1919	0.0762
9.88E-08	5.24E-05	0.0130	0.0695	1.1237	0.0506
1.88E-08	2.28E-05	0.0130	0.0695	1.0458	0.0194
1.61E-09	6.69E-06	0.0138	0.0706	1.0122	0.0053
4.17E-10	3.40E-06	0.0139	0.0706	1.0074	0.0032
9.64E-10	5.17E-06	0.0139	0.0706	1.0074	0.0032
1.56E-09	6.58E-06	0.0139	0.0706	1.0074	0.0032
1.96E-09	7.38E-06	0.0139	0.0706	1.0074	0.0032
1.96E-09	7.38E-06	0.0139	0.0706	1.0074	0.0032

1.96E-09	7.38E-06	0.0139	0.0706	1.0074	0.0032
6.65E-10	4.30E-06	0.0139	0.0706	1.0074	0.0032
9.64E-10	5.17E-06	0.0139	0.0706	1.0074	0.0032
9.64E-10	5.17E-06	0.0139	0.0706	1.0074	0.0032
8.14E-10	4.76E-06	0.0139	0.0706	1.0074	0.0032
6.15E-10	4.13E-06	0.0139	0.0706	1.0074	0.0032
6.83E-06	0.000435648	0.0140	0.0706	1.7246	0.2367
1.91E-06	0.000230421	0.0140	0.0706	1.4720	0.1679
2.58E-07	8.46E-05	0.0140	0.0706	1.1188	0.0487
3.43E-08	3.09E-05	0.0140	0.0706	1.0586	0.0247
1.17E-09	5.70E-06	0.0146	0.0727	1.0110	0.0048
4.90E-09	1.17E-05	0.0146	0.0727	1.0118	0.0051
4.55E-09	1.12E-05	0.0146	0.0727	1.0118	0.0051
2.06E-09	7.57E-06	0.0146	0.0727	1.0118	0.0051
7.49E-07	0.000144282	0.0150	0.0736	1.3635	0.1347
9.03E-07	0.000158405	0.0150	0.0736	1.1952	0.0775
5.75E-07	0.000126421	0.0150	0.0736	1.1916	0.0761
8.28E-08	4.80E-05	0.0150	0.0736	1.1139	0.0468
1.61E-07	6.69E-05	0.0160	0.0777	1.1136	0.0467
1.73E-07	6.94E-05	0.0160	0.0777	1.1760	0.0704
2.42E-08	2.59E-05	0.0170	0.0820	1.0429	0.0182
7.04E-09	1.40E-05	0.0170	0.0820	1.0306	0.0131
5.59E-07	0.000124558	0.0180	0.0856	1.3183	0.1200
8.97E-08	4.99E-05	0.0180	0.0856	1.0883	0.0367
1.45E-06	0.000200797	0.0180	0.0856	1.5085	0.1785
6.75E-08	4.33E-05	0.0180	0.0856	1.0763	0.0320
4.70E-11	1.14E-06	0.0183	0.0871	0.9919	-0.0035
4.14E-09	1.07E-05	0.0193	0.0911	1.0126	0.0054
6.76E-06	0.000433425	0.0200	0.0930	1.4611	0.1647
1.03E-08	1.69E-05	0.0200	0.0930	1.0464	0.0197
6.36E-08	4.20E-05	0.0200	0.0930	1.0654	0.0275
4.77E-08	3.64E-05	0.0200	0.0930	1.0687	0.0288
8.95E-10	4.99E-06	0.0204	0.0935	1.0086	0.0037
7.46E-10	4.55E-06	0.0204	0.0935	1.0086	0.0037
2.49E-09	8.31E-06	0.0204	0.0935	1.0086	0.0037
1.14E-09	5.64E-06	0.0204	0.0935	1.0086	0.0037
5.97E-10	4.07E-06	0.0204	0.0935	1.0086	0.0037
0.000209499	0.002412345	0.0210	0.0958	4.1438	0.6174
2.99E-06	0.000288158	0.0220	0.0981	1.6714	0.2231
7.25E-08	4.49E-05	0.0220	0.0981	1.0852	0.0355
5.68E-08	3.97E-05	0.0220	0.0981	1.0803	0.0335
3.07E-08	2.92E-05	0.0220	0.0981	1.0451	0.0191
2.01E-08	2.36E-05	0.0220	0.0981	1.0451	0.0191
1.83E-08	2.25E-05	0.0220	0.0981	1.0459	0.0195

9.70E-09	1.64E-05	0.0220	0.0981	1.0311	0.0133
2.56E-10	2.67E-06	0.0224	0.0998	0.9907	-0.0041
4.64E-07	0.000113522	0.0230	0.1019	0.4014	-0.3964
1.25E-07	5.89E-05	0.0240	0.1057	1.1265	0.0517
7.01E-08	4.41E-05	0.0240	0.1057	1.0615	0.0259
2.01E-06	0.000236233	0.0250	0.1081	0.2420	-0.6162
2.70E-08	2.74E-05	0.0250	0.1081	0.6721	-0.1726
1.20E-09	5.77E-06	0.0253	0.1081	1.0066	0.0028
5.53E-10	3.92E-06	0.0253	0.1081	1.0066	0.0028
6.02E-10	4.09E-06	0.0253	0.1081	1.0066	0.0028
1.55E-09	6.56E-06	0.0253	0.1081	1.0066	0.0028
1.55E-09	6.56E-06	0.0253	0.1081	1.0066	0.0028
1.20E-09	5.77E-06	0.0253	0.1081	1.0066	0.0028
0	0	0.0256	0.1081	0.9951	-0.0021
0	0	0.0256	0.1081	0.9951	-0.0021
1.89E-06	0.000229395	0.0260	0.1081	1.3527	0.1312
1.50E-08	2.04E-05	0.0260	0.1081	0.7603	-0.1190
2.26E-06	0.00025069	0.0260	0.1081	1.6013	0.2045
4.66E-07	0.000113793	0.0260	0.1081	1.1213	0.0497
2.56E-09	8.42E-06	0.0260	0.1081	0.8841	-0.0535
5.80E-07	0.000126934	0.0260	0.1081	1.3196	0.1204
2.58E-07	8.47E-05	0.0260	0.1081	1.1434	0.0582
1.92E-08	2.31E-05	0.0260	0.1081	1.0372	0.0159
6.97E-08	4.40E-05	0.0260	0.1081	1.1019	0.0422
1.29E-07	6.00E-05	0.0270	0.1117	1.0826	0.0345
1.65E-06	0.000213816	0.0270	0.1117	1.4951	0.1747
5.46E-07	0.000123121	0.0280	0.1145	0.1480	-0.8296
1.48E-05	0.000641605	0.0280	0.1145	2.4280	0.3853
1.34E-07	6.10E-05	0.0280	0.1145	1.1442	0.0585
7.86E-09	1.48E-05	0.0280	0.1145	1.0343	0.0146
8.70E-10	4.92E-06	0.0285	0.1163	0.9903	-0.0042
0.000630603	0.004185303	0.0290	0.1175	6.7306	0.8281
7.34E-08	4.52E-05	0.0290	0.1175	1.1050	0.0434
5.28E-10	3.83E-06	0.0300	0.1198	0.8460	-0.0726
1.35E-05	0.000611923	0.0300	0.1198	2.2080	0.3440
5.53E-07	0.000123993	0.0300	0.1198	1.2196	0.0862
5.35E-09	1.22E-05	0.0300	0.1198	1.0294	0.0126
1.10E-09	5.53E-06	0.0300	0.1198	0.9876	-0.0054
9.53E-10	5.14E-06	0.0300	0.1198	0.9876	-0.0054
7.52E-08	4.57E-05	0.0310	0.1232	1.0944	0.0392
1.17E-09	5.70E-06	0.0318	0.1258	1.0106	0.0046
1.59E-08	2.10E-05	0.0320	0.1258	0.8429	-0.0742
9.09E-08	5.03E-05	0.0320	0.1258	1.0762	0.0319
4.72E-09	1.15E-05	0.0320	0.1258	1.0257	0.0110

4.40E-09	1.11E-05	0.0333	0.1300	1.0134	0.0058
4.94E-07	0.000117139	0.0340	0.1322	1.2693	0.1036
9.67E-11	1.64E-06	0.0351	0.1362	0.9931	-0.0030
9.79E-07	0.00016489	0.0360	0.1392	1.3290	0.1235
1.40E-09	6.24E-06	0.0365	0.1399	1.0082	0.0035
6.54E-10	4.26E-06	0.0365	0.1399	1.0082	0.0035
2.49E-09	8.32E-06	0.0365	0.1399	1.0082	0.0035
6.04E-09	1.30E-05	0.0370	0.1415	1.0278	0.0119
1.61E-09	6.68E-06	0.0375	0.1427	1.0090	0.0039
1.31E-09	6.03E-06	0.0375	0.1427	1.0090	0.0039
2.42E-11	8.20E-07	0.0380	0.1442	0.9429	-0.0255
2.99E-08	2.88E-05	0.0390	0.1476	0.4794	-0.3193
1.24E-07	5.88E-05	0.0400	0.1501	1.1273	0.0520
3.41E-08	3.08E-05	0.0410	0.1527	0.1242	-0.9060
6.17E-08	4.14E-05	0.0410	0.1527	0.5864	-0.2318
2.98E-06	0.000287778	0.0410	0.1527	1.5832	0.1995
8.56E-09	1.54E-05	0.0430	0.1597	1.0310	0.0133
3.87E-10	3.28E-06	0.0447	0.1631	0.9923	-0.0034
3.13E-08	2.95E-05	0.0450	0.1631	0.3824	-0.4175
1.18E-09	5.74E-06	0.0468	0.1631	1.0057	0.0025
3.39E-10	3.07E-06	0.0468	0.1631	1.0057	0.0025
5.88E-10	4.04E-06	0.0468	0.1631	1.0057	0.0025
1.18E-09	5.74E-06	0.0468	0.1631	1.0057	0.0025
1.18E-09	5.74E-06	0.0468	0.1631	1.0057	0.0025
5.88E-10	4.04E-06	0.0468	0.1631	1.0057	0.0025
1.18E-09	5.74E-06	0.0468	0.1631	1.0057	0.0025
1.18E-09	5.74E-06	0.0468	0.1631	1.0057	0.0025
5.88E-10	4.04E-06	0.0468	0.1631	1.0057	0.0025
1.18E-09	5.74E-06	0.0468	0.1631	1.0057	0.0025
1.18E-09	5.74E-06	0.0468	0.1631	1.0057	0.0025
1.18E-09	5.74E-06	0.0468	0.1631	1.0057	0.0025
1.18E-09	5.74E-06	0.0468	0.1631	1.0057	0.0025
1.18E-09	5.74E-06	0.0468	0.1631	1.0057	0.0025
8.86E-10	4.96E-06	0.0468	0.1631	1.0057	0.0025
8.86E-10	4.96E-06	0.0468	0.1631	1.0057	0.0025
8.86E-10	4.96E-06	0.0468	0.1631	1.0057	0.0025
6.87E-10	4.37E-06	0.0468	0.1631	1.0057	0.0025
6.87E-10	4.37E-06	0.0468	0.1631	1.0057	0.0025
6.38E-10	4.21E-06	0.0468	0.1631	1.0057	0.0025
3.39E-10	3.07E-06	0.0468	0.1631	1.0057	0.0025
1.03E-07	5.35E-05	0.0470	0.1631	1.1168	0.0480
2.34E-06	0.000254899	0.0480	0.1657	1.4687	0.1669
2.56E-10	2.67E-06	0.0480	0.1657	0.8263	-0.0828
**GC.Tumor-GC.Para-Genus**					

1.25E-08	1.94E-05	0.0000	0.0000	1.0277	0.0119
0	0	0.0000	0.0000	0.9721	-0.0123
0	0	0.0000	0.0000	0.9721	-0.0123
0	0	0.0000	0.0000	0.9744	-0.0112
0	0	0.0000	0.0000	0.9744	-0.0112
3.63E-09	1.05E-05	0.0000	0.0000	1.0232	0.0100
1.29E-08	1.98E-05	0.0000	0.0000	1.0224	0.0096
0	0	0.0000	0.0000	0.9776	-0.0099
0	0	0.0000	0.0000	0.9776	-0.0099
0	0	0.0000	0.0000	0.9784	-0.0095
1.02E-08	1.76E-05	0.0000	0.0000	1.0260	0.0112
3.56E-09	1.04E-05	0.0000	0.0000	1.0261	0.0112
1.06E-08	1.79E-05	0.0000	0.0000	1.0251	0.0108
0	0	0.0000	0.0000	0.9799	-0.0088
0	0	0.0000	0.0000	0.9799	-0.0088
5.11E-11	1.24E-06	0.0000	0.0000	0.9754	-0.0108
0	0	0.0000	0.0000	0.9807	-0.0085
7.42E-09	1.50E-05	0.0000	0.0000	1.0243	0.0104
2.64E-11	8.94E-07	0.0000	0.0000	0.9792	-0.0091
6.04E-09	1.35E-05	0.0000	0.0001	1.0170	0.0073
2.29E-09	8.33E-06	0.0000	0.0001	1.0161	0.0069
1.52E-08	2.15E-05	0.0000	0.0002	1.0198	0.0085
9.15E-09	1.66E-05	0.0000	0.0002	1.0217	0.0093
0	0	0.0000	0.0003	0.9847	-0.0067
0	0	0.0000	0.0003	0.9847	-0.0067
4.54E-09	1.17E-05	0.0000	0.0003	1.0189	0.0081
2.84E-09	9.27E-06	0.0000	0.0004	1.0143	0.0062
0	0	0.0000	0.0005	0.9855	-0.0064
0	0	0.0000	0.0005	0.9855	-0.0064
1.78E-08	2.32E-05	0.0000	0.0006	1.0208	0.0090
0	0	0.0000	0.0009	0.9863	-0.0060
1.65E-08	2.23E-05	0.0000	0.0011	1.0209	0.0090
1.96E-09	7.71E-06	0.0000	0.0013	1.0125	0.0054
5.17E-09	1.25E-05	0.0000	0.0013	1.0125	0.0054
3.51E-10	3.26E-06	0.0000	0.0013	0.9789	-0.0093
0	0	0.0000	0.0014	0.9871	-0.0057
0	0	0.0000	0.0014	0.9871	-0.0057
0	0	0.0000	0.0014	0.9871	-0.0057
2.79E-10	2.91E-06	0.0001	0.0019	0.9804	-0.0086
2.37E-10	2.68E-06	0.0001	0.0024	0.9833	-0.0073
7.87E-09	1.54E-05	0.0001	0.0026	1.0201	0.0086
2.59E-10	2.80E-06	0.0001	0.0027	0.9819	-0.0080
1.48E-09	6.69E-06	0.0001	0.0028	1.0135	0.0058
2.64E-11	8.94E-07	0.0001	0.0031	0.9863	-0.0060

5.41E-09	1.28E-05	0.0001	0.0037	1.0201	0.0087
1.64E-09	7.04E-06	0.0001	0.0042	1.0144	0.0062
3.20E-09	9.85E-06	0.0001	0.0042	1.0107	0.0046
1.05E-10	1.79E-06	0.0002	0.0045	0.9856	-0.0063
2.64E-11	8.94E-07	0.0002	0.0049	0.9871	-0.0056
2.64E-11	8.94E-07	0.0002	0.0049	0.9871	-0.0056
7.42E-11	1.50E-06	0.0004	0.0097	0.9857	-0.0062
7.09E-10	4.63E-06	0.0004	0.0100	0.9814	-0.0081
1.16E-08	1.88E-05	0.0004	0.0103	1.0155	0.0067
9.50E-09	1.70E-05	0.0004	0.0103	1.0155	0.0067
6.59E-10	4.47E-06	0.0004	0.0105	0.9835	-0.0072
0	0	0.0006	0.0133	0.9903	-0.0042
0	0	0.0006	0.0133	0.9903	-0.0042
2.64E-11	8.94E-07	0.0006	0.0143	0.9887	-0.0049
8.42E-10	5.05E-06	0.0006	0.0143	1.0089	0.0039
2.64E-09	8.94E-06	0.0006	0.0143	1.0089	0.0039
1.25E-09	6.15E-06	0.0006	0.0143	1.0108	0.0047
5.30E-09	1.27E-05	0.0007	0.0143	1.0156	0.0067
9.52E-09	1.70E-05	0.0007	0.0143	1.0146	0.0063
3.10E-09	9.69E-06	0.0007	0.0143	1.0146	0.0063
9.49E-10	5.36E-06	0.0007	0.0143	0.9836	-0.0072
1.96E-09	7.71E-06	0.0009	0.0188	1.0118	0.0051
5.17E-09	1.25E-05	0.0009	0.0188	1.0118	0.0051
8.85E-08	5.18E-05	0.0010	0.0200	1.0590	0.0249
4.44E-08	3.67E-05	0.0010	0.0200	1.0393	0.0168
0	0	0.0011	0.0208	0.9911	-0.0039
0	0	0.0011	0.0208	0.9911	-0.0039
4.33E-09	1.15E-05	0.0011	0.0208	1.0147	0.0063
1.06E-08	1.79E-05	0.0011	0.0208	1.0147	0.0063
2.79E-10	2.91E-06	0.0011	0.0214	0.9851	-0.0065
1.50E-10	2.13E-06	0.0012	0.0214	0.9866	-0.0059
3.80E-09	1.07E-05	0.0013	0.0226	1.0099	0.0043
2.17E-09	8.10E-06	0.0013	0.0226	1.0099	0.0043
6.68E-10	4.50E-06	0.0013	0.0227	1.0080	0.0035
7.76E-10	4.85E-06	0.0013	0.0227	1.0080	0.0035
1.32E-09	6.33E-06	0.0013	0.0227	1.0080	0.0035
3.54E-09	1.04E-05	0.0019	0.0324	1.0118	0.0051
4.52E-09	1.17E-05	0.0019	0.0324	1.0118	0.0051
6.53E-09	1.41E-05	0.0020	0.0332	1.0387	0.0165
2.64E-11	8.94E-07	0.0021	0.0332	0.9903	-0.0042
2.64E-11	8.94E-07	0.0021	0.0332	0.9903	-0.0042
0	0	0.0021	0.0332	0.9919	-0.0035
0	0	0.0021	0.0332	0.9919	-0.0035
0	0	0.0021	0.0332	0.9919	-0.0035

0	0	0.0021	0.0332	0.9919	-0.0035
1.45E-09	6.63E-06	0.0024	0.0373	1.0090	0.0039
4.32E-09	1.14E-05	0.0024	0.0373	1.0178	0.0076
5.70E-10	4.16E-06	0.0027	0.0390	0.9839	-0.0070
7.63E-10	4.81E-06	0.0027	0.0390	1.0072	0.0031
1.69E-09	7.15E-06	0.0027	0.0390	1.0072	0.0031
1.31E-09	6.29E-06	0.0027	0.0390	1.0072	0.0031
9.81E-10	5.45E-06	0.0027	0.0390	1.0072	0.0031
9.81E-10	5.45E-06	0.0027	0.0390	1.0072	0.0031
9.81E-10	5.45E-06	0.0027	0.0390	1.0072	0.0031
9.49E-10	5.36E-06	0.0028	0.0393	0.9860	-0.0061
7.42E-11	1.50E-06	0.0030	0.0424	0.9889	-0.0048
7.40E-09	1.50E-05	0.0031	0.0426	1.0149	0.0064
9.56E-11	1.70E-06	0.0031	0.0430	0.9882	-0.0052
5.17E-09	1.25E-05	0.0034	0.0456	1.0109	0.0047
3.82E-10	3.40E-06	0.0035	0.0467	0.9853	-0.0064
2.64E-11	8.94E-07	0.0038	0.0481	0.9911	-0.0039
2.64E-11	8.94E-07	0.0038	0.0481	0.9911	-0.0039
2.64E-11	8.94E-07	0.0038	0.0481	0.9911	-0.0039
2.64E-11	8.94E-07	0.0038	0.0481	0.9911	-0.0039
2.64E-11	8.94E-07	0.0038	0.0481	0.9911	-0.0039
0	0	0.0042	0.0517	0.9927	-0.0032
0	0	0.0042	0.0517	0.9927	-0.0032
1.05E-08	1.79E-05	0.0042	0.0517	1.0130	0.0056
1.05E-10	1.79E-06	0.0047	0.0575	0.9904	-0.0042
1.37E-09	6.45E-06	0.0048	0.0578	1.0140	0.0060
7.42E-11	1.50E-06	0.0050	0.0598	0.9520	-0.0214
2.37E-10	2.68E-06	0.0051	0.0603	0.9897	-0.0045
4.22E-10	3.58E-06	0.0051	0.0603	0.9890	-0.0048
6.54E-10	4.45E-06	0.0053	0.0621	0.9861	-0.0061
1.29E-09	6.26E-06	0.0057	0.0637	1.0063	0.0027
6.40E-10	4.40E-06	0.0057	0.0637	1.0063	0.0027
6.40E-10	4.40E-06	0.0057	0.0637	1.0063	0.0027
1.29E-09	6.26E-06	0.0057	0.0637	1.0063	0.0027
6.40E-10	4.40E-06	0.0057	0.0637	1.0063	0.0027
3.86E-09	1.08E-05	0.0059	0.0648	1.0100	0.0043
1.39E-09	6.48E-06	0.0060	0.0652	0.9855	-0.0063
8.31E-10	5.02E-06	0.0060	0.0652	0.8271	-0.0825
9.52E-09	1.70E-05	0.0067	0.0724	1.0121	0.0052
2.64E-11	8.94E-07	0.0069	0.0738	0.9920	-0.0035
2.35E-09	8.43E-06	0.0070	0.0738	0.9039	-0.0439
2.79E-10	2.91E-06	0.0070	0.0738	0.8906	-0.0503
1.12E-09	5.84E-06	0.0075	0.0784	0.9856	-0.0063
7.58E-10	4.79E-06	0.0078	0.0811	0.9850	-0.0066

6.59E-10	4.47E-06	0.0079	0.0811	0.9883	-0.0051
1.29E-05	0.000625424	0.0080	0.0819	1.8301	0.2625
0	0	0.0082	0.0831	0.9935	-0.0028
0	0	0.0082	0.0831	0.9935	-0.0028
2.59E-10	2.80E-06	0.0083	0.0834	0.9898	-0.0045
5.70E-10	4.16E-06	0.0088	0.0874	0.9863	-0.0060
3.69E-09	1.06E-05	0.0088	0.0874	1.0121	0.0052
3.22E-09	9.88E-06	0.0093	0.0917	1.0132	0.0057
2.72E-09	9.09E-06	0.0096	0.0937	1.0101	0.0044
3.28E-10	3.15E-06	0.0100	0.0953	0.6834	-0.1653
1.19E-07	6.01E-05	0.0100	0.0953	1.1377	0.0560
2.65E-09	8.96E-06	0.0101	0.0953	1.0082	0.0035
1.56E-09	6.87E-06	0.0101	0.0953	1.0082	0.0035
1.41E-09	6.54E-06	0.0102	0.0953	1.0091	0.0040
2.17E-09	8.11E-06	0.0102	0.0953	1.0091	0.0040
1.13E-08	1.85E-05	0.0110	0.1003	0.4875	-0.3120
5.60E-09	1.30E-05	0.0110	0.1003	1.0296	0.0127
8.69E-09	1.62E-05	0.0110	0.1003	0.5246	-0.2802
1.14E-09	5.88E-06	0.0110	0.1003	0.8402	-0.0756
7.45E-09	1.50E-05	0.0113	0.1016	1.0152	0.0066
2.42E-10	2.71E-06	0.0120	0.1016	1.0054	0.0023
9.49E-10	5.36E-06	0.0120	0.1016	1.0054	0.0023
9.49E-10	5.36E-06	0.0120	0.1016	1.0054	0.0023
9.49E-10	5.36E-06	0.0120	0.1016	1.0054	0.0023
5.14E-10	3.95E-06	0.0120	0.1016	1.0054	0.0023
3.51E-10	3.26E-06	0.0120	0.1016	1.0054	0.0023
2.97E-10	3.00E-06	0.0120	0.1016	1.0054	0.0023
4.60E-10	3.73E-06	0.0120	0.1016	1.0054	0.0023
2.42E-10	2.71E-06	0.0120	0.1016	1.0054	0.0023
1.50E-10	2.13E-06	0.0120	0.1016	0.9595	-0.0179
5.46E-10	4.07E-06	0.0121	0.1024	0.9877	-0.0054
2.34E-09	8.42E-06	0.0132	0.1105	0.9851	-0.0065
2.72E-09	9.07E-06	0.0136	0.1136	1.0113	0.0049
6.75E-09	1.43E-05	0.0142	0.1176	1.0102	0.0044
2.59E-10	2.80E-06	0.0150	0.1233	0.9415	-0.0262
3.77E-08	3.38E-05	0.0160	0.1271	1.0510	0.0216
0	0	0.0162	0.1271	0.9943	-0.0025
0	0	0.0162	0.1271	0.9943	-0.0025
0	0	0.0162	0.1271	0.9943	-0.0025
0	0	0.0162	0.1271	0.9943	-0.0025
0	0	0.0162	0.1271	0.9943	-0.0025
0	0	0.0162	0.1271	0.9943	-0.0025
0	0	0.0162	0.1271	0.9943	-0.0025
5.25E-05	0.001261488	0.0170	0.1322	0.2105	-0.6767

8.60E-10	5.11E-06	0.0174	0.1349	1.0082	0.0036
9.58E-09	1.70E-05	0.0180	0.1377	0.8534	-0.0689
2.02E-09	7.82E-06	0.0180	0.1377	0.8642	-0.0634
3.80E-09	1.07E-05	0.0187	0.1427	0.9873	-0.0056
0.065975064	0.04471291	0.0190	0.1430	0.5225	-0.2819
1.05E-10	1.79E-06	0.0190	0.1430	0.8286	-0.0816
2.10E-08	2.52E-05	0.0200	0.1467	0.7813	-0.1072
3.00E-07	9.53E-05	0.0200	0.1467	0.5370	-0.2700
3.79E-08	3.39E-05	0.0200	0.1467	0.6948	-0.1582
1.05E-08	1.79E-05	0.0200	0.1467	0.7930	-0.1007
3.02E-09	9.56E-06	0.0200	0.1467	0.8906	-0.0503
5.11E-11	1.24E-06	0.0210	0.1516	0.9231	-0.0347
1.12E-08	1.84E-05	0.0210	0.1516	0.6801	-0.1674
3.99E-09	1.10E-05	0.0210	0.1516	0.9130	-0.0395
1.73E-06	0.000228677	0.0220	0.1573	0.2590	-0.5867
1.40E-09	6.52E-06	0.0220	0.1573	0.9127	-0.0397
2.64E-11	8.94E-07	0.0227	0.1596	0.9936	-0.0028
2.64E-11	8.94E-07	0.0227	0.1596	0.9936	-0.0028
2.64E-11	8.94E-07	0.0227	0.1596	0.9936	-0.0028
2.56E-07	8.81E-05	0.0230	0.1596	0.4716	-0.3264
9.56E-09	1.70E-05	0.0230	0.1596	0.7261	-0.1390
9.90E-08	5.48E-05	0.0240	0.1639	0.7115	-0.1478
3.80E-08	3.39E-05	0.0240	0.1639	0.7930	-0.1007
1.30E-09	6.27E-06	0.0240	0.1639	0.9377	-0.0279
1.05E-10	1.79E-06	0.0241	0.1639	0.9928	-0.0031
8.11E-09	1.57E-05	0.0250	0.1642	1.0371	0.0158
6.59E-10	4.47E-06	0.0250	0.1642	1.0045	0.0019
6.59E-10	4.47E-06	0.0250	0.1642	1.0045	0.0019
6.59E-10	4.47E-06	0.0250	0.1642	1.0045	0.0019
4.42E-10	3.66E-06	0.0250	0.1642	1.0045	0.0019
3.33E-10	3.18E-06	0.0250	0.1642	1.0045	0.0019
3.33E-10	3.18E-06	0.0250	0.1642	1.0045	0.0019
4.83E-08	3.83E-05	0.0260	0.1689	0.4270	-0.3696
2.38E-07	8.48E-05	0.0260	0.1689	0.5446	-0.2639
1.96E-09	7.70E-06	0.0262	0.1695	1.0083	0.0036
2.79E-08	2.91E-05	0.0270	0.1738	1.0665	0.0280
1.47E-07	6.67E-05	0.0290	0.1858	0.7419	-0.1297
3.87E-09	1.08E-05	0.0292	0.1861	0.9867	-0.0058
1.12E-09	5.84E-06	0.0296	0.1871	1.0074	0.0032
1.18E-09	5.98E-06	0.0296	0.1871	1.0074	0.0032
8.36E-08	5.03E-05	0.0300	0.1888	0.6761	-0.1700
1.51E-09	6.77E-06	0.0306	0.1917	1.0105	0.0045
1.32E-09	6.33E-06	0.0321	0.1973	1.0064	0.0028
1.05E-09	5.64E-06	0.0321	0.1973	1.0064	0.0028

0	0	0.0321	0.1973	0.9951	-0.0021
0	0	0.0321	0.1973	0.9951	-0.0021
0	0	0.0321	0.1973	0.9951	-0.0021
2.37E-10	2.68E-06	0.0377	0.2278	0.9929	-0.0031
1.29E-10	1.97E-06	0.0377	0.2278	0.9929	-0.0031
6.26E-09	1.38E-05	0.0378	0.2278	1.0095	0.0041
3.05E-09	9.61E-06	0.0378	0.2278	1.0095	0.0041
9.49E-10	5.36E-06	0.0386	0.2318	0.9916	-0.0037
1.88E-10	2.39E-06	0.0387	0.2318	0.9907	-0.0040
1.91E-09	7.60E-06	0.0390	0.2321	0.9191	-0.0367
1.69E-09	7.16E-06	0.0400	0.2361	0.9631	-0.0163
1.34E-08	2.02E-05	0.0400	0.2361	0.9051	-0.0433
2.64E-11	8.94E-07	0.0407	0.2390	0.9944	-0.0024
2.87E-07	9.32E-05	0.0410	0.2390	0.6874	-0.1628
3.36E-09	1.01E-05	0.0410	0.2390	0.8975	-0.0470
3.80E-09	1.07E-05	0.0425	0.2459	1.0074	0.0032
8.60E-10	5.11E-06	0.0425	0.2459	1.0074	0.0032
5.11E-11	1.24E-06	0.0450	0.2581	0.7760	-0.1101
1.69E-07	7.16E-05	0.0450	0.2581	0.6845	-0.1646
3.16E-09	9.78E-06	0.0460	0.2628	0.8713	-0.0598
4.86E-08	3.84E-05	0.0480	0.2719	0.8439	-0.0737
1.69E-09	7.16E-06	0.0487	0.2719	0.9877	-0.0054
2.15E-09	8.07E-06	0.0495	0.2719	1.0065	0.0028
2.15E-09	8.07E-06	0.0495	0.2719	1.0065	0.0028
2.84E-07	9.28E-05	0.0500	0.2719	1.0992	0.0411
2.85E-09	9.30E-06	0.0500	0.2719	0.8750	-0.0580
**CRC.Tumor-CRC.Para-Genus**					
0	0	0.0000	0.0002	0.9725	-0.0121
1.60E-08	2.58E-05	0.0000	0.0002	1.0283	0.0121
6.73E-09	1.67E-05	0.0000	0.0002	1.0270	0.0116
3.63E-11	1.23E-06	0.0000	0.0020	0.9749	-0.0111
8.12E-09	1.84E-05	0.0000	0.0022	1.0221	0.0095
7.30E-09	1.74E-05	0.0000	0.0031	1.0209	0.0090
6.33E-09	1.62E-05	0.0000	0.0033	1.0233	0.0100
0	0	0.0000	0.0033	0.9807	-0.0085
5.27E-09	1.48E-05	0.0000	0.0033	1.0197	0.0085
0	0	0.0000	0.0033	0.9807	-0.0085
8.15E-09	1.84E-05	0.0000	0.0033	1.0197	0.0085
6.19E-09	1.61E-05	0.0001	0.0058	1.0184	0.0079
1.45E-10	2.46E-06	0.0001	0.0059	0.9772	-0.0100
3.50E-09	1.21E-05	0.0001	0.0060	1.0209	0.0090
8.34E-09	1.86E-05	0.0001	0.0060	1.0209	0.0090
1.24E-08	2.27E-05	0.0001	0.0066	1.0245	0.0105
0	0	0.0001	0.0079	0.9831	-0.0074

1.96E-09	9.04E-06	0.0001	0.0079	1.0172	0.0074
0	0	0.0001	0.0079	0.9831	-0.0074
1.45E-10	2.46E-06	0.0002	0.0120	0.9796	-0.0090
2.12E-09	9.39E-06	0.0002	0.0120	1.0160	0.0069
4.46E-09	1.36E-05	0.0002	0.0120	1.0160	0.0069
5.22E-09	1.47E-05	0.0002	0.0120	1.0160	0.0069
3.18E-09	1.15E-05	0.0002	0.0120	1.0160	0.0069
6.13E-09	1.60E-05	0.0002	0.0120	1.0160	0.0069
0	0	0.0002	0.0120	0.9843	-0.0069
3.91E-09	1.28E-05	0.0003	0.0130	1.0184	0.0079
9.65E-09	2.01E-05	0.0003	0.0143	1.0232	0.0100
0	0	0.0005	0.0171	0.9855	-0.0064
0	0	0.0005	0.0171	0.9855	-0.0064
5.22E-09	1.48E-05	0.0005	0.0171	1.0148	0.0064
0	0	0.0005	0.0171	0.9855	-0.0064
5.22E-09	1.48E-05	0.0005	0.0171	1.0148	0.0064
5.22E-09	1.48E-05	0.0005	0.0171	1.0148	0.0064
2.04E-09	9.23E-06	0.0005	0.0171	1.0148	0.0064
8.16E-09	1.84E-05	0.0005	0.0171	1.0172	0.0074
1.05E-08	2.09E-05	0.0007	0.0171	1.0184	0.0079
7.76E-09	1.80E-05	0.0009	0.0171	1.0220	0.0094
1.06E-09	6.64E-06	0.0010	0.0171	1.0135	0.0058
1.66E-09	8.33E-06	0.0010	0.0171	1.0135	0.0058
0	0	0.0010	0.0171	0.9713	-0.0126
3.71E-08	3.93E-05	0.0010	0.0171	1.0455	0.0193
0	0	0.0010	0.0171	0.5971	-0.2240
0	0	0.0010	0.0171	0.9239	-0.0344
1.52E-06	0.000251868	0.0010	0.0171	1.2962	0.1127
4.96E-08	4.54E-05	0.0010	0.0171	1.0529	0.0224
1.19E-06	0.000222354	0.0010	0.0171	1.3466	0.1293
3.05E-07	0.000112795	0.0010	0.0171	1.2431	0.0945
2.20E-07	9.58E-05	0.0010	0.0171	1.0996	0.0412
3.12E-08	3.60E-05	0.0010	0.0171	1.0443	0.0188
0	0	0.0010	0.0171	0.8799	-0.0555
0	0	0.0010	0.0171	0.8230	-0.0846
3.43E-07	0.000119614	0.0010	0.0171	1.1610	0.0648
5.76E-08	4.90E-05	0.0010	0.0171	1.0578	0.0244
0	0	0.0010	0.0171	0.8954	-0.0480
2.81E-07	0.000108174	0.0010	0.0171	1.1082	0.0446
0	0	0.0010	0.0171	0.9014	-0.0451
6.70E-08	5.28E-05	0.0010	0.0171	1.0541	0.0229
4.69E-08	4.42E-05	0.0010	0.0171	1.0455	0.0193
0	0	0.0010	0.0171	0.9229	-0.0349
0	0	0.0010	0.0171	0.9239	-0.0344

0	0	0.0010	0.0171	0.9622	-0.0168
0	0	0.0010	0.0171	0.9324	-0.0304
7.26E-09	1.74E-05	0.0010	0.0171	1.0356	0.0152
9.13E-08	6.17E-05	0.0010	0.0171	1.0701	0.0294
8.71E-08	6.02E-05	0.0010	0.0171	1.0602	0.0254
1.23E-08	2.27E-05	0.0010	0.0171	1.0406	0.0173
8.65E-09	1.90E-05	0.0010	0.0171	1.0369	0.0157
2.53E-08	3.25E-05	0.0010	0.0171	1.0418	0.0178
0	0	0.0010	0.0171	0.9610	-0.0173
0	0	0.0010	0.0171	0.9702	-0.0131
0	0	0.0010	0.0171	0.9702	-0.0131
1.71E-08	2.67E-05	0.0010	0.0171	1.0381	0.0162
7.87E-09	1.81E-05	0.0010	0.0171	1.0356	0.0152
2.42E-09	1.00E-05	0.0018	0.0310	1.0147	0.0064
3.63E-09	1.23E-05	0.0020	0.0316	1.0123	0.0053
3.63E-09	1.23E-05	0.0020	0.0316	1.0123	0.0053
2.95E-09	1.11E-05	0.0020	0.0316	1.0123	0.0053
1.43E-09	7.72E-06	0.0020	0.0316	1.0123	0.0053
6.47E-09	1.64E-05	0.0023	0.0370	1.0207	0.0089
5.22E-09	1.48E-05	0.0034	0.0541	1.0135	0.0058
1.44E-08	2.45E-05	0.0037	0.0576	1.0195	0.0084
6.43E-09	1.64E-05	0.0037	0.0576	1.0195	0.0084
2.33E-09	9.86E-06	0.0039	0.0579	1.0111	0.0048
1.42E-09	7.70E-06	0.0039	0.0579	1.0111	0.0048
0	0	0.0039	0.0579	0.9891	-0.0048
1.12E-09	6.83E-06	0.0039	0.0579	1.0111	0.0048
7.11E-09	1.72E-05	0.0042	0.0614	1.0147	0.0063
2.32E-09	9.83E-06	0.0060	0.0871	0.4214	-0.3753
1.78E-09	8.60E-06	0.0072	0.1002	1.0159	0.0069
1.36E-09	7.53E-06	0.0074	0.1002	1.0135	0.0058
6.19E-09	1.61E-05	0.0075	0.1002	1.0147	0.0063
1.41E-09	7.67E-06	0.0078	0.1002	1.0098	0.0042
1.19E-09	7.03E-06	0.0078	0.1002	1.0098	0.0042
1.41E-09	7.67E-06	0.0078	0.1002	1.0098	0.0042
0	0	0.0078	0.1002	0.9903	-0.0042
2.32E-09	9.83E-06	0.0078	0.1002	1.0098	0.0042
1.79E-09	8.64E-06	0.0078	0.1002	1.0098	0.0042
0	0	0.0078	0.1002	0.9903	-0.0042
2.32E-09	9.83E-06	0.0078	0.1002	1.0098	0.0042
1.19E-09	7.03E-06	0.0078	0.1002	1.0098	0.0042
5.65E-09	1.53E-05	0.0094	0.1189	1.0171	0.0074
1.58E-09	8.12E-06	0.0100	0.1259	0.5872	-0.2312
1.92E-06	0.000283145	0.0110	0.1373	1.5346	0.1860
1.20E-09	7.08E-06	0.0117	0.1439	1.0110	0.0048

3.63E-09	1.23E-05	0.0117	0.1439	1.0110	0.0048
3.07E-08	3.57E-05	0.0120	0.1459	1.0784	0.0328
3.24E-09	1.16E-05	0.0130	0.1567	0.7754	-0.1105
3.63E-11	1.23E-06	0.0140	0.1674	0.9357	-0.0289
8.69E-10	6.02E-06	0.0156	0.1752	1.0086	0.0037
0	0	0.0156	0.1752	0.9915	-0.0037
0	0	0.0156	0.1752	0.9915	-0.0037
0	0	0.0156	0.1752	0.9915	-0.0037
0	0	0.0156	0.1752	0.9915	-0.0037
1.32E-09	7.42E-06	0.0156	0.1752	1.0086	0.0037
9.44E-10	6.27E-06	0.0156	0.1752	1.0086	0.0037
7.71E-08	5.67E-05	0.0160	0.1778	1.1066	0.0440
2.88E-07	0.000109622	0.0180	0.1985	1.2501	0.0970
2.14E-07	9.43E-05	0.0190	0.2079	0.5422	-0.2658
3.01E-09	1.12E-05	0.0192	0.2088	1.0135	0.0058
3.63E-11	1.23E-06	0.0210	0.2263	0.9433	-0.0254
1.66E-09	8.33E-06	0.0213	0.2266	1.0122	0.0053
3.63E-11	1.23E-06	0.0215	0.2266	0.9903	-0.0042
1.20E-09	7.06E-06	0.0215	0.2266	1.0098	0.0042
6.94E-11	1.70E-06	0.0225	0.2335	0.9891	-0.0048
2.57E-09	1.04E-05	0.0225	0.2335	1.0110	0.0048
5.47E-09	1.51E-05	0.0227	0.2338	1.0146	0.0063
1.57E-06	0.00025563	0.0230	0.2354	1.4356	0.1570
3.59E-07	0.000122285	0.0240	0.2439	1.2272	0.0889
2.26E-08	3.07E-05	0.0270	0.2705	1.0551	0.0233
7.17E-10	5.47E-06	0.0290	0.2865	0.9856	-0.0063
1.31E-09	7.38E-06	0.0290	0.2865	0.8613	-0.0649
4.46E-09	1.36E-05	0.0309	0.2890	1.0122	0.0053
1.68E-07	8.38E-05	0.0310	0.2890	0.6776	-0.1690
7.96E-08	5.76E-05	0.0310	0.2890	0.4960	-0.3046
7.00E-10	5.40E-06	0.0312	0.2890	1.0074	0.0032
9.27E-10	6.22E-06	0.0312	0.2890	1.0074	0.0032
1.31E-09	7.38E-06	0.0312	0.2890	1.0074	0.0032
1.31E-09	7.38E-06	0.0312	0.2890	1.0074	0.0032
0	0	0.0312	0.2890	0.9927	-0.0032
6.24E-10	5.10E-06	0.0312	0.2890	1.0074	0.0032
1.41E-08	2.42E-05	0.0340	0.3120	1.0592	0.0250
2.91E-09	1.10E-05	0.0347	0.3166	1.0134	0.0058
2.12E-09	9.40E-06	0.0352	0.3168	1.0110	0.0048
0.005631663	0.015318375	0.0360	0.3221	0.5313	-0.2746
2.95E-09	1.11E-05	0.0386	0.3413	1.0098	0.0042
1.11E-09	6.80E-06	0.0391	0.3413	1.0086	0.0037
8.83E-10	6.07E-06	0.0391	0.3413	1.0086	0.0037
2.32E-09	9.83E-06	0.0391	0.3413	1.0086	0.0037

2.71E-09	1.06E-05	0.0414	0.3578	1.0122	0.0053
4.84E-09	1.42E-05	0.0420	0.3578	1.0269	0.0115
3.44E-08	3.79E-05	0.0420	0.3578	1.0661	0.0278
2.46E-08	3.20E-05	0.0420	0.3578	1.0657	0.0276
1.42E-09	7.70E-06	0.0428	0.3625	0.9856	-0.0063
8.69E-10	6.02E-06	0.0433	0.3626	0.9868	-0.0058
2.52E-09	1.02E-05	0.0433	0.3626	1.0134	0.0058
3.15E-07	0.000114522	0.0450	0.3722	1.2375	0.0925
5.41E-07	0.000150136	0.0450	0.3722	1.2234	0.0876
0.000557332	0.00481894	0.0460	0.3783	0.4029	-0.3948
8.01E-05	0.001827265	0.0470	0.3821	2.7885	0.4454
1.35E-05	0.00074948	0.0470	0.3821	1.7541	0.2441
