# Supplementary material for: Combined Non-Invasive Prediction and New Biomarkers of Oral and Fecal Microbiota in Patients With Gastric and Colorectal Cancer
Source: Front Cell Infect Microbiol. 2022 May 19;12:830684. doi: 10.3389/fcimb.2022.830684 (PMC9161364; doi:10.3389/fcimb.2022.830684)
Supplement: Supplementary file 1 [file DataSheet_1.zip › Supplementary Table 6.pdf]

Table S6. Network analysis of microbiota shared by tumor, paracancerous tissue, oral cavity, and stool in HC, GC and CRC.

Taxonomy
**Oral: Tissue**
k__Bacteria;p__Firmicutes;c__Bacilli;o__Lactobacillales;f__Streptococcaceae;g__Streptococcus
k__Bacteria;p__Proteobacteria;c__Gammaproteobacteria;o__Burkholderiales;f__Neisseriaceae;g__Neisseria;s__Neisseria_mucosa
k__Bacteria;p__Proteobacteria;c__Gammaproteobacteria;o__Pasteurellales;f__Pasteurellaceae;g__Haemophilus;s__Haemophilus_parainfluenzae
k__Bacteria;p__Actinobacteriota;c__Actinobacteria;o__Micrococcales;f__Micrococcaceae;g__Rothia;s__
k__Bacteria;p__Firmicutes;c__Bacilli;o__Staphylococcales;f__Gemellaceae;g__Gemella;s__
k__Bacteria;p__Proteobacteria;c__Gammaproteobacteria;o__Burkholderiales;f__Burkholderiaceae;g__Lautropia;s__
k__Bacteria;p__Proteobacteria;c__Gammaproteobacteria;o__Pasteurellales;f__Pasteurellaceae;g__Haemophilus
k__Bacteria;p__Fusobacteriota;c__Fusobacteriia;o__Fusobacteriales;f__Fusobacteriaceae;g__Fusobacterium;s__Fusobacterium_nucleatum
k__Bacteria;p__Proteobacteria;c__Gammaproteobacteria;o__Burkholderiales;f__Neisseriaceae;g__Neisseria
k__Bacteria;p__Bacteroidota;c__Bacteroidia;o__Bacteroidales;f__Prevotellaceae;g__Prevotella;s__Prevotella_melaninogenica
k__Bacteria;p__Firmicutes;c__Negativicutes;o__Veillonellales-Selenomonadales;f__Veillonellaceae;g__Veillonella
k__Bacteria;p__Proteobacteria;c__Gammaproteobacteria;o__Pasteurellales;f__Pasteurellaceae;g__Aggregatibacter;s__
k__Bacteria;p__Proteobacteria;c__Gammaproteobacteria;o__Pasteurellales;f__Pasteurellaceae;g__Actinobacillus;s__Haemophilus_parahaemolyticus
k__Bacteria;p__Actinobacteriota;c__Actinobacteria;o__Actinomycetales;f__Actinomycetaceae;g__Actinomyces
k__Bacteria;p__Proteobacteria;c__Gammaproteobacteria;o__Burkholderiales;f__Neisseriaceae;g__Neisseria
k__Bacteria;p__Fusobacteriota;c__Fusobacteriia;o__Fusobacteriales;f__Fusobacteriaceae;g__Fusobacterium;s__Fusobacterium_periodonticum
**Stool: Tissue**
k__Bacteria;p__Firmicutes;c__Bacilli;o__Lactobacillales;f__Streptococcaceae;g__Streptococcus
k__Bacteria;p__Proteobacteria;c__Gammaproteobacteria;o__Enterobacterales;f__Enterobacteriaceae;g__Escherichia-Shigella;s__Escherichia_coli
k__Bacteria;p__Firmicutes;c__Clostridia;o__Oscillospirales;f__Ruminococcaceae;g__Faecalibacterium;s__Faecalibacterium_prausnitzii
k__Bacteria;p__Bacteroidota;c__Bacteroidia;o__Bacteroidales;f__Bacteroidaceae;g__Bacteroides;s__Bacteroides_vulgatus
k__Bacteria;p__Proteobacteria;c__Gammaproteobacteria;o__Enterobacterales;f__Enterobacteriaceae;g__Klebsiella
k__Bacteria;p__Firmicutes;c__Clostridia;o__Lachnospirales;f__Lachnospiraceae;g__Agathobacter;s__
k__Bacteria;p__Firmicutes;c__Clostridia;o__Lachnospirales;f__Lachnospiraceae;g__Blautia

k__Bacteria;p__Bacteroidota;c__Bacteroidia;o__Bacteroidales;f__Bacteroidaceae;g__Bacteroides;s__Bacteroides_plebeius
k__Bacteria;p__Bacteroidota;c__Bacteroidia;o__Bacteroidales;f__Bacteroidaceae;g__Bacteroides;s__Bacteroides_uniformis
k__Bacteria;p__Firmicutes;c__Clostridia;o__Oscillospirales;f__Ruminococcaceae;g__Subdoligranulum;s__
k__Bacteria;p__Bacteroidota;c__Bacteroidia;o__Bacteroidales;f__Prevotellaceae;g__Prevotella
k__Bacteria;p__Firmicutes;c__Clostridia;o__Lachnospirales;f__Lachnospiraceae;g__Roseburia
k__Bacteria;p__Bacteroidota;c__Bacteroidia;o__Bacteroidales;f__Tannerellaceae;g__Parabacteroides;s__Parabacteroides_sp_CT06
k__Bacteria;p__Bacteroidota;c__Bacteroidia;o__Bacteroidales;f__Bacteroidaceae;g__Bacteroides;s__Bacteroides_dorei
k__Bacteria;p__Bacteroidota;c__Bacteroidia;o__Bacteroidales;f__Bacteroidaceae;g__Bacteroides
k__Bacteria;p__Firmicutes;c__Clostridia;o__Oscillospirales;f__Ruminococcaceae;g__Faecalibacterium

OTU	CAG	logFC	logCPM	PValue	FDR.BH	FDR	FC
**Oral: Tissue**							
OTU_1	Biofilm CAG	0.0882	17.1325	0.774959	0.881382	> 0.1	<= 1
OTU_4	Biofilm CAG	-0.901	15.0271	0.0645676	0.1561605	> 0.1	<= 1
OTU_6	Biofilm CAG	0.3777	15.1516	0.262742	0.4266992	> 0.1	<= 1
OTU_9	Biofilm CAG	0.6779	14.5681	0.1580621	0.2963441	> 0.1	<= 1
OTU_10	Biofilm CAG	-0.005	14.4009	0.9981335	1	> 0.1	<= 1
OTU_17	Biofilm CAG	-0.387	13.8648	0.4566631	0.6274158	> 0.1	<= 1
OTU_19	Biofilm CAG	0.8655	14.2011	0.0273081	0.08266	<= 0.1	<= 1
OTU_24	Oral Pathogen CAG	0.198	13.4912	0.5046256	0.6545915	> 0.1	<= 1
OTU_30	Biofilm CAG	0.7344	12.2073	0.0591181	0.1461693	> 0.1	<= 1
OTU_38	Oral Pathogen CAG	-1.079	13.3804	0.0061029	0.0261817	<= 0.1	<= 1
OTU_45	Oral Pathogen CAG	-0.368	13.5291	0.3153148	0.4848082	> 0.1	<= 1
OTU_47	Biofilm CAG	-0.41	11.0195	0.3341497	0.4954846	> 0.1	<= 1
OTU_49	Biofilm CAG	-0.794	13.263	0.0662899	0.1592474	> 0.1	<= 1
OTU_50	Biofilm CAG	-0.841	13.4162	0.1236779	0.2497891	> 0.1	<= 1
OTU_4521	Biofilm CAG	0.0786	12.8396	0.8271699	0.9196306	> 0.1	<= 1
OTU_5051	Oral Pathogen CAG	-0.135	12.785	0.6903394	0.8455748	> 0.1	<= 1
**Stool: Tissue**							
OTU_1	cluster 3	-2.769	14.0717	4.38E-14	5.65E-11	<= 0.1	<= 1
OTU_5	cluster 3	2.5699	16.0497	4.44E-12	2.18E-09	<= 0.1	> 1
OTU_7	cluster 1	0.7864	14.2281	0.013625	0.039244	<= 0.1	<= 1
OTU_8	cluster 2	1.4483	14.5395	5.79E-05	0.0005422	<= 0.1	> 1
OTU_13	cluster 3	2.0669	15.6972	5.34E-06	8.94E-05	<= 0.1	> 1
OTU_14	cluster 1	-0.119	12.3801	0.7309761	0.821906	> 0.1	<= 1
OTU_16	cluster 1	-0.173	12.6591	0.6357951	0.7580271	> 0.1	<= 1

OTU_18	cluster 1	0.1602	12.3117	0.7448194	0.8342846	> 0.1	<= 1
OTU_22	cluster 2	0.3723	12.5205	0.3145321	0.4325183	> 0.1	<= 1
OTU_23	cluster 1	1.6895	12.408	7.39E-07	1.97E-05	<= 0.1	> 1
OTU_28	cluster 1	-0.741	12.9015	0.1335268	0.2292776	> 0.1	<= 1
OTU_29	cluster 1	0.2997	11.6992	0.3901829	0.5114403	> 0.1	<= 1
OTU_60	cluster 2	0.8197	13.0688	0.0349944	0.0811329	<= 0.1	<= 1
OTU_802	cluster 2	1.0846	12.7487	0.0099087	0.0306601	<= 0.1	> 1
OTU_11972	cluster 2	0.8554	12.3914	0.0212137	0.0550995	<= 0.1	<= 1
OTU_27940	cluster 1	0.7777	12.6295	0.0245473	0.0619364	<= 0.1	<= 1

**Phylum**		
**Firmicutes**		
k__Bacteria;p__Firmicutes;c__Bacilli;o__Lactobacillales;f__Streptococcaceae;g__Streptococcus	OTU_1	Biofilm CAG/ cluster 3
k__Bacteria;p__Firmicutes;c__Clostridia;o__Oscillospirales;f__Ruminococcaceae;g__Faecalibacterium;s__Faecalibacterium_prausnitzii	OTU_7	cluster 1
k__Bacteria;p__Firmicutes;c__Bacilli;o__Staphylococcales;f__Gemellaceae;g__Gemella;s__	OTU_10	Biofilm CAG
k__Bacteria;p__Firmicutes;c__Clostridia;o__Lachnospirales;f__Lachnospiraceae;g__Agathobacter;s__	OTU_14	cluster 1
k__Bacteria;p__Firmicutes;c__Clostridia;o__Lachnospirales;f__Lachnospiraceae;g__Blautia	OTU_16	cluster 1
k__Bacteria;p__Firmicutes;c__Clostridia;o__Oscillospirales;f__Ruminococcaceae;g__Subdoligranulum;s__	OTU_23	cluster 1
k__Bacteria;p__Firmicutes;c__Clostridia;o__Lachnospirales;f__Lachnospiraceae;g__Roseburia	OTU_29	cluster 1
k__Bacteria;p__Firmicutes;c__Negativicutes;o__Veillonellales-Selenomonadales;f__Veillonellaceae;g__Veillonella	OTU_45	Oral Pathogen CAG
k__Bacteria;p__Firmicutes;c__Clostridia;o__Oscillospirales;f__Ruminococcaceae;g__Faecalibacterium	OTU_27940	cluster 1
**Proteobacteria**		
k__Bacteria;p__Proteobacteria;c__Gammaproteobacteria;o__Burkholderiales;f__Neisseriaceae;g__Neisseria;s__Neisseria_mucosa	OTU_4	cluster 2
k__Bacteria;p__Proteobacteria;c__Gammaproteobacteria;o__Enterobacterales;f__Enterobacteriaceae;g__Escherichia-Shigella;s__Escherichia_coli	OTU_5	cluster 3
k__Bacteria;p__Proteobacteria;c__Gammaproteobacteria;o__Pasteurellales;f__Pasteurellaceae;g__Haemophilus;s__Haemophilus_parainfluenzae	OTU_6	Biofilm CAG
k__Bacteria;p__Proteobacteria;c__Gammaproteobacteria;o__Enterobacterales;f__Enterobacteriaceae;g__Klebsiella	OTU_13	cluster 3
k__Bacteria;p__Proteobacteria;c__Gammaproteobacteria;o__Burkholderiales;f__Burkholderiaceae;g__Lautropia;s__	OTU_17	Biofilm CAG
k__Bacteria;p__Proteobacteria;c__Gammaproteobacteria;o__Pasteurellales;f__Pasteurellaceae;g__Haemophilus	OTU_19	Biofilm CAG

k__Bacteria;p__Proteobacteria;c__Gammaproteobacteria;o__Burkholderiales;f__Neisseriaceae;g__Neisseria	OTU_30	Biofilm CAG
k__Bacteria;p__Proteobacteria;c__Gammaproteobacteria;o__Pasteurellales;f__Pasteurellaceae;g__Aggregatibacter;s__	OTU_47	Biofilm CAG
k__Bacteria;p__Proteobacteria;c__Gammaproteobacteria;o__Pasteurellales;f__Pasteurellaceae;g__Actinobacillus;s__Haemophilus_parahaemolyticus	OTU_49	Biofilm CAG
k__Bacteria;p__Proteobacteria;c__Gammaproteobacteria;o__Burkholderiales;f__Neisseriaceae;g__Neisseria	OTU_4521	Biofilm CAG
**Bacteroidota**		
k__Bacteria;p__Bacteroidota;c__Bacteroidia;o__Bacteroidales;f__Bacteroidaceae;g__Bacteroides;s__Bacteroides_vulgatus	OTU_8	cluster 2
k__Bacteria;p__Bacteroidota;c__Bacteroidia;o__Bacteroidales;f__Bacteroidaceae;g__Bacteroides;s__Bacteroides_plebeius	OTU_18	cluster 1
k__Bacteria;p__Bacteroidota;c__Bacteroidia;o__Bacteroidales;f__Bacteroidaceae;g__Bacteroides;s__Bacteroides_uniformis	OTU_22	cluster 2
k__Bacteria;p__Bacteroidota;c__Bacteroidia;o__Bacteroidales;f__Prevotellaceae;g__Prevotella	OTU_28	cluster 1
k__Bacteria;p__Bacteroidota;c__Bacteroidia;o__Bacteroidales;f__Prevotellaceae;g__Prevotella;s__Prevotella_melaninogenica	OTU_38	Oral Pathogen CAG
k__Bacteria;p__Bacteroidota;c__Bacteroidia;o__Bacteroidales;f__Tannerellaceae;g__Parabacteroides;s__Parabacteroides_sp_CT06	OTU_60	cluster 2
k__Bacteria;p__Bacteroidota;c__Bacteroidia;o__Bacteroidales;f__Bacteroidaceae;g__Bacteroides;s__Bacteroides_dorei	OTU_802	cluster 2
k__Bacteria;p__Bacteroidota;c__Bacteroidia;o__Bacteroidales;f__Bacteroidaceae;g__Bacteroides	OTU_11972	cluster 2
**Actinobacteriota**		
k__Bacteria;p__Actinobacteriota;c__Actinobacteria;o__Micrococcales;f__Micrococcaceae;g__Rothia;s__	OTU_9	Biofilm CAG
k__Bacteria;p__Actinobacteriota;c__Actinobacteria;o__Actinomycetales;f__Actinomycetaceae;g__Actinomyces	OTU_50	Biofilm CAG
**Fusobacteriota**		
k__Bacteria;p__Fusobacteriota;c__Fusobacteriia;o__Fusobacteriales;f__Fusobacteriaceae;g__Fusobacterium;s__Fusobacterium_nucleatum	OTU_24	Oral Pathogen CAG
k__Bacteria;p__Fusobacteriota;c__Fusobacteriia;o__Fusobacteriales;f__Fusobacteriaceae;g__Fusobacterium;s__Fusobacterium_periodonticum	OTU_5051	Oral Pathogen CAG
