# Supplementary material for: Combined Non-Invasive Prediction and New Biomarkers of Oral and Fecal Microbiota in Patients With Gastric and Colorectal Cancer
Source: Front Cell Infect Microbiol. 2022 May 19;12:830684. doi: 10.3389/fcimb.2022.830684 (PMC9161364; doi:10.3389/fcimb.2022.830684)
Supplement: Supplementary file 1 [file DataSheet_1.zip › Supplementary Table 7.pdf]

Table S7. Identification and validation of GC, CRC and GI markers based on oral and fecal microbial OTU.

GC.Oral: N.Oral	GC.Stool: N.Stool	CRC.Oral: N.Oral	CRC.Stool: N.Stool	GI.Oral: N.Oral	GI.Stool: N.Stool
OTU_62	OTU_42	OTU_30	OTU_79	OTU_30	OTU_15
OTU_80	OTU_48	OTU_78	OTU_127	OTU_47	OTU_27
OTU_121	OTU_132	OTU_160	OTU_197	OTU_49	OTU_43
OTU_132	OTU_138	OTU_409	OTU_467	OTU_115	OTU_48
OTU_288	OTU_163	OTU_599	OTU_5550	OTU_281	OTU_57
OTU_300	OTU_430	OTU_5284	OTU_8667	OTU_311	OTU_58
OTU_337	OTU_1121	OTU_9805		OTU_356	OTU_82
OTU_371	OTU_10429	OTU_22277		OTU_364	OTU_86
OTU_647	OTU_14528	OTU_23699		OTU_409	OTU_132
OTU_649				OTU_464	OTU_159
OTU_6130				OTU_721	OTU_163
OTU_9805				OTU_757	OTU_197
OTU_23699				OTU_2903	OTU_233
				OTU_4903	OTU_239
				OTU_5284	OTU_489
				OTU_6130	OTU_1626
				OTU_7128	OTU_7407
				OTU_9805	OTU_7854
				OTU_22277	OTU_10429
				OTU_23699	OTU_11040

OTU_15	k__Bacteria;p__Firmicutes;c__Negativicutes;o__Veillonellales-Selenomonadales;f__Veillonellaceae;g__Dialister;s__
OTU_27	k__Bacteria;p__Firmicutes;c__Negativicutes;o__Veillonellales-Selenomonadales;f__Veillonellaceae;g__Dialister;s__Dialister_sp_Marseille-P5638
OTU_30	k__Bacteria;p__Proteobacteria;c__Gammaproteobacteria;o__Burkholderiales;f__Neisseriaceae;g__Neisseria
OTU_42	k__Bacteria;p__Firmicutes;c__Clostridia;o__Lachnospirales;f__Lachnospiraceae;g__Lachnospira;s__
OTU_43	k__Bacteria;p__Firmicutes;c__Negativicutes;o__Acidaminococcales;f__Acidaminococcaceae;g__Phascolarctobacterium;s__Phascolarctobacterium_faecium
OTU_47	k__Bacteria;p__Proteobacteria;c__Gammaproteobacteria;o__Pasteurellales;f__Pasteurellaceae;g__Aggregatibacter;s__
OTU_48	k__Bacteria;p__Firmicutes;c__Clostridia;o__Clostridiales;f__Clostridiaceae;g__Clostridium_sensu_stricto_1;s__Clostridium_perfringens
OTU_49	k__Bacteria;p__Proteobacteria;c__Gammaproteobacteria;o__Pasteurellales;f__Pasteurellaceae;g__Actinobacillus;s__Haemophilus_parahaemolyticus
OTU_57	k__Bacteria;p__Firmicutes;c__Negativicutes;o__Veillonellales-Selenomonadales;f__Selenomonadaceae;g__Megamonas;s__

OTU_58	k__Bacteria;p__Firmicutes;c__Bacilli;o__Lactobacillales;f__Lactobacillaceae;g__Lactobacillus;s__Lactobacillus_salivarius
OTU_62	k__Bacteria;p__Bacteroidota;c__Bacteroidia;o__Bacteroidales;f__Prevotellaceae;g__Alloprevotella
OTU_78	k__Bacteria;p__Proteobacteria;c__Gammaproteobacteria;o__Burkholderiales;f__Neisseriaceae;g__Neisseria;s__Neisseria_elongata
OTU_79	k__Bacteria;p__Firmicutes;c__Clostridia;o__Peptostreptococcales-Tissierellales;f__unidentified_Peptostreptococcales-Tissierellales;g__Parvimonas;s__Parvimonas_micra
OTU_80	k__Bacteria;p__Bacteroidota;c__Bacteroidia;o__Bacteroidales;f__Prevotellaceae;g__Alloprevotella;s__Prevotella_sp
OTU_82	k__Bacteria;p__Firmicutes;c__Clostridia;o__Peptostreptococcales-Tissierellales;f__Peptostreptococcaceae;g__Peptostreptococcus;s__
OTU_86	k__Bacteria;p__Proteobacteria;c__Gammaproteobacteria;o__Burkholderiales;f__Sutterellaceae;g__Parasutterella;s__
OTU_115	k__Bacteria;p__Bacteroidota;c__Bacteroidia;o__Bacteroidales;f__Prevotellaceae;g__Prevotella;s__Prevotella_nanceiensis
OTU_121	k__Bacteria;p__Actinobacteriota;c__Actinobacteria;o__Actinomycetales;f__Actinomycetaceae;g__F0332;s__
OTU_127	k__Bacteria;p__Firmicutes;c__Clostridia;o__Oscillospirales;f__Ruminococcaceae;g__UBA1819;s__
OTU_132	k__Bacteria;p__Firmicutes;c__Bacilli;o__Lactobacillales;f__Streptococcaceae;g__Streptococcus;s__Streptococcus_anginosus
OTU_138	k__Bacteria;p__Firmicutes;c__Bacilli;o__Lactobacillales;f__Lactobacillaceae;g__Lactobacillus
OTU_159	k__Bacteria;p__Firmicutes;c__Clostridia;o__Oscillospirales;f__Oscillospiraceae;g__NK4A214_group;s__
OTU_160	k__Bacteria;p__Proteobacteria;c__Gammaproteobacteria;o__Pasteurellales;f__Pasteurellaceae;g__Aggregatibacter
OTU_163	k__Bacteria;p__Firmicutes;c__Bacilli;o__Lactobacillales;f__Lactobacillaceae;g__Lactobacillus;s__Lactobacillus_mucosae
OTU_197	k__Bacteria;p__Bacteroidota;c__Bacteroidia;o__Bacteroidales;f__Prevotellaceae;g__Paraprevotella;s__
OTU_233	k__Bacteria;p__Desulfobacterota;c__Desulfovibrionia;o__Desulfovibrionales;f__Desulfovibrionaceae;g__Desulfovibrio;s__Desulfovibrio_piger
OTU_239	k__Bacteria;p__Firmicutes;c__Bacilli;o__Lactobacillales;f__Lactobacillaceae;g__Lactobacillus
OTU_281	k__Bacteria;p__Gracilibacteria;c__unidentified_Gracilibacteria;o__Absconditabacteriales_(SR1);f__unidentified_Absconditabacteriales_(SR1);g__unidentified_Absconditabacteriales_(SR1);s__SR1_bacterium_oral_taxon_875
OTU_288	k__Bacteria;p__Proteobacteria;c__Gammaproteobacteria;o__Burkholderiales;f__Neisseriaceae;g__Kingella;s__
OTU_300	k__Bacteria;p__Bacteroidota;c__Bacteroidia;o__Bacteroidales;f__Prevotellaceae;g__Prevotella;s__Prevotella_pallens

OTU_311	k__Bacteria;p__Firmicutes;c__Bacilli;o__Erysipelotrichales;f__Erysipelotrichaceae;g__Solobacterium;s__Solobacterium_moorei
OTU_336	k__Bacteria;p__Bacteroidota;c__Bacteroidia;o__Bacteroidales;f__Prevotellaceae;g__Prevotella;s__Prevotella_buccae
OTU_337	k__Bacteria;p__Proteobacteria;c__Gammaproteobacteria;o__Burkholderiales;f__Neisseriaceae;g__Eikenella;s__Eikenella_corrodens
OTU_356	k__Bacteria;p__Bacteroidota;c__Bacteroidia;o__Bacteroidales;f__Prevotellaceae;g__Prevotella;s__Prevotella_aurantiaca
OTU_364	k__Bacteria;p__Firmicutes;c__Clostridia;o__Lachnospirales;f__Lachnospiraceae;g__Johnsonella;s__
OTU_371	k__Bacteria;p__Proteobacteria;c__Gammaproteobacteria;o__Burkholderiales;f__Comamonadaceae;g__Comamonas;s__Ottowia_sp_oral_taxon_894
OTU_409	k__Bacteria;p__Firmicutes;c__Clostridia;o__Peptostreptococcales-Tissierellales;f__Anaerovoracaceae;g__[Eubacterium]_nodatum_group
OTU_430	k__Bacteria;p__Proteobacteria;c__Gammaproteobacteria;o__Burkholderiales;f__Sutterellaceae;g__Parasutterella;s__Parasutterella_secunda
OTU_464	k__Bacteria;p__Firmicutes;c__Negativicutes;o__Veillonellales-Selenomonadales;f__Veillonellaceae;g__Megasphaera;s__Megasphaera_micronuciformis
OTU_467	k__Bacteria;p__Firmicutes;c__Clostridia;o__Clostridiales;f__Clostridiaceae;g__Clostridium_sensu_stricto_18
OTU_489	k__Bacteria;p__Firmicutes;c__Clostridia;o__Lachnospirales;f__Lachnospiraceae
OTU_599	k__Bacteria;p__Actinobacteriota;c__Actinobacteria;o__Propionibacteriales;f__Propionibacteriaceae;g__Cutibacterium;s__Cutibacterium_granulosum
OTU_647	k__Bacteria;p__Firmicutes;c__Clostridia;o__Lachnospirales;f__Lachnospiraceae;g__Butyrivibrio;s__Firmicutes_oral_clone_BB124
OTU_649	k__Bacteria;p__unidentified_Bacteria;c__Negativicutes;o__Veillonellales-Selenomonadales;f__Selenomonadaceae;g__unidentified_Selenomonadaceae;s__Mitsuokella_sp_oral_taxon_G68
OTU_721	k__Bacteria;p__Firmicutes;c__Negativicutes;o__Veillonellales-Selenomonadales;f__Veillonellaceae;g__Veillonella;s__Veillonella_atypica
OTU_757	k__Bacteria;p__Firmicutes;c__Clostridia;o__Peptostreptococcales-Tissierellales;f__Anaerovoracaceae;g__Amnipila;s__
OTU_1121	k__Bacteria;p__Firmicutes;c__Clostridia;o__Peptococcales;f__Peptococcaceae;g__Peptococcus;s__
OTU_1626	k__Bacteria;p__Firmicutes;c__Clostridia;o__Lachnospirales;f__Lachnospiraceae;g__Lachnoclostridium
OTU_2903	k__Bacteria;p__Spirochaetota;c__Spirochaetia;o__Spirochaetales;f__Spirochaetaceae;g__Treponema;s__Treponema_sp_oral_taxon_246
OTU_4903	k__Bacteria;p__Firmicutes;c__Bacilli;o__Lactobacillales;f__Streptococcaceae;g__Streptococcus
OTU_5284	k__Bacteria;p__Proteobacteria;c__Gammaproteobacteria;o__Pasteurellales;f__Pasteurellaceae

OTU_5550	k__Bacteria;p__Firmicutes;c__Clostridia;o__Oscillospirales;f__Oscillospiraceae;g__Colidextribacter
OTU_6130	k__Bacteria;p__Firmicutes;c__Negativicutes;o__Veillonellales-Selenomonadales;f__Veillonellaceae;g__Veillonella
OTU_7128	k__Bacteria;p__Fusobacteriota;c__Fusobacteriia;o__Fusobacteriales;f__Fusobacteriaceae;g__Fusobacterium
OTU_7407	k__Bacteria;p__Firmicutes;c__Clostridia;o__Oscillospirales;f__Ruminococcaceae;g__Faecalibacterium
OTU_7854	k__Bacteria;p__Firmicutes;c__Bacilli;o__Erysipelotrichales;f__Erysipelotrichaceae;g__Holdemanella;s__
OTU_8667	k__Bacteria;p__Firmicutes;c__Clostridia;o__Lachnospirales;f__Lachnospiraceae;g__Fusicatenibacter;s__
OTU_9805	k__Bacteria;p__Proteobacteria;c__Gammaproteobacteria;o__Burkholderiales;f__Neisseriaceae;g__Neisseria
OTU_10429	k__Bacteria;p__Bacteroidota;c__Bacteroidia;o__Bacteroidales;f__Prevotellaceae;g__Paraprevotella
OTU_11040	Unknown
OTU_11626	k__Bacteria;p__Bacteroidota;c__Bacteroidia;o__Bacteroidales;f__Prevotellaceae;g__Prevotella;s__Prevotellaceae_bacterium_DJF_CR21k6
OTU_14528	k__Bacteria;p__Firmicutes;c__Bacilli;o__Lactobacillales;f__Streptococcaceae;g__Streptococcus
OTU_22277	k__Bacteria;p__Actinobacteriota;c__Actinobacteria;o__Micrococcales;f__Micrococcaceae;g__Rothia;s__Rothia_mucilaginosa
OTU_23699	k__Bacteria;p__Proteobacteria;c__Gammaproteobacteria;o__Burkholderiales;f__Burkholderiaceae;g__Lautropia;s__
